# Landscaping the Nitazene Scaffold to Identify Novel Mu-Opioid Receptor Modulators: From Molecular Design and Chemical Synthesis to Pharmacological Profiling

**DOI:** 10.1021/acs.jmedchem.6c00283

**Published:** 2026-07-06

**Authors:** Neha Upadhyay, Logan T. Neel, Ennian Li, Abeje A. Silte, Rui Lyu, Dana E. Selley, William L. Dewey, Yan Zhang, Piyusha P. Pagare

**Affiliations:** a Department of Medicinal Chemistry, School of Pharmacy, 6889 Virginia Commonwealth University, Richmond, Virginia 23298, United States; b Department of Pharmacology and Toxicology, 6889 Virginia Commonwealth University, Richmond, Virginia 23298, United States; c Center for Drug Discovery, 6889 Virginia Commonwealth University, Richmond, Virginia 23298, United States

## Abstract

The surge in ultrapotent synthetic opioids has intensified the need for new chemical strategies for modulating the mu-opioid receptor (MOR). In this study, nitazene scaffold, a chemically distinct and underexplored chemotype, was utilized as an underexplored framework for MOR modulation. Structure–activity relationship studies were conducted through modifications at three key positions of the nitazene core and evaluated for MOR binding, in vitro functional, and in vivo behavioral activities. Among them, compound **26** significantly blocked the effects of synthetic opioids, including fentanyl and etonitazene. It also demonstrated affinity and selectivity for MOR and exhibited favorable metabolic stability and CNS permeability. Molecular modeling studies provided structural insights into potential binding modes of these ligands. This work expands the chemical space for opioid receptor ligands and provides a proof-of-concept that the nitazene core is chemically tunable for MOR functional modulation, highlighting nitazenes as a promising platform for future therapeutic exploration.

## Graphical abstract

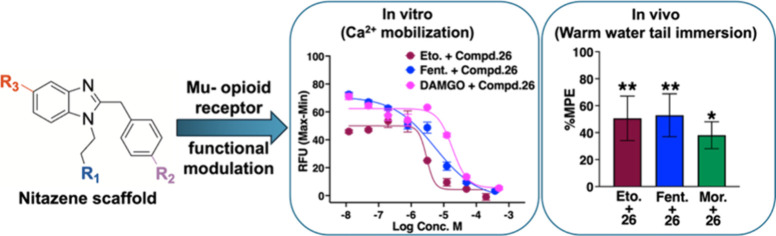

## Introduction

The opioid epidemic continues to pose a severe public health threat, with opioid use disorder (OUD) and overdose-related deaths affecting millions globally.^1^ In the United States, 2023 saw an estimated 107,543 drug overdose deaths, approximately 81,083 of which involved opioids.^2^ While this represents a modest 3% decline from 2022—the first annual decrease since 2018 due to public health interventions—opioid-related mortality remains alarmingly high.^3–5^ Among these deaths, synthetic opioids, particularly illicitly manufactured fentanyl (IMF), remain the dominant drivers, while emerging nonfentanyl novel synthetic opioids (NSOs) continue to appear in the recreational drug supply.^6–9^ Synthetic opioids are a structurally diverse class of mu-opioid receptor (MOR) agonists that include both fentanyl analogs as well as newly emerging nonfentanyl substances.^10^ Advances in analytical detection have unveiled the extent and complexity of NSO involvement in overdose related cases,^11–13^ underscoring the need to understand how structural features influence MOR activity across distinct synthetic opioid chemotypes.^2,5^ IMFs fuel the current overdose crisis due to their extreme potency (50–10,000 times that of morphine), rapid onset, and high lipophilicity, all of which contribute to an increased risk of fatal respiratory depression.^14^

More recently, the 2-benzylbenzimidazole (nitazene) scaffold has garnered attention due to its extreme potency, with several derivatives exceeding that of fentanyl.^15–18^ Etonitazene, first identified in the 1950s by CIBA for its analgesic potential,^19,20^ was nearly 1,000-fold more potent than morphine^21,22^ and 10-fold more potent than fentanyl.^23,24^ Many nitazene derivatives (Figure 1) are ultrapotent MOR agonists, associated with respiratory depression, seizures and overdose.^13,25–33^ Despite Drug Enforcement Administration (DEA) scheduling efforts,^34^ new analogs continue to emerge,^20^ and online drug forums discussions indicate increasing availability of these drugs^6,25,35^ posing a significant public health threat.^15,36^ While naloxone (NLX), a potent and neutral MOR antagonist, has been reported to reverse nitazene-induced overdose, their high potency may require significantly higher or multiple doses of NLX to achieve the same or similar reversal effects.^37–40^

**Figure 1:**
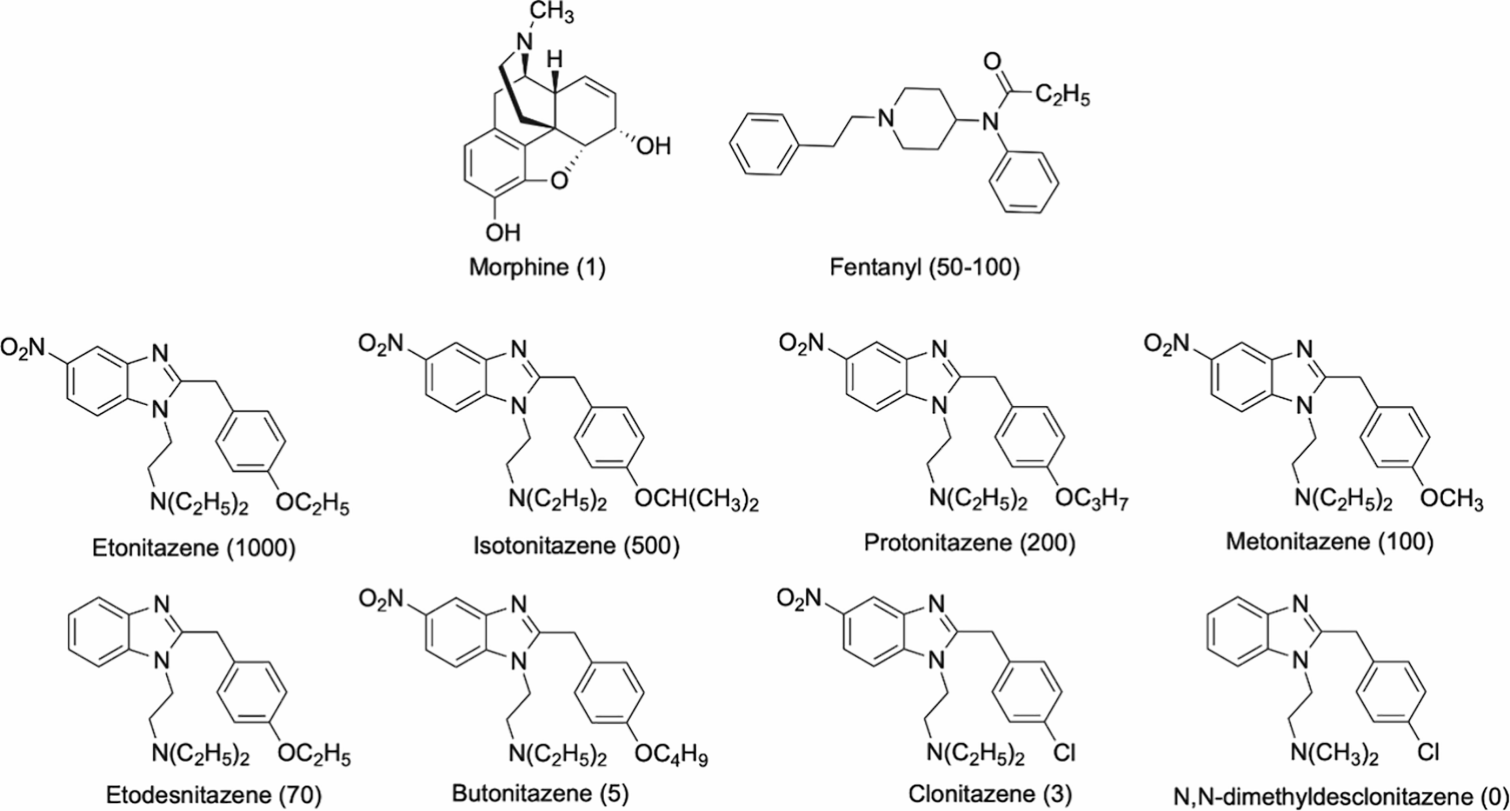
Chemical structures of morphine, fentanyl, and representative nitazene analogs. Antinociceptive activity relative to morphine in radiant heat tail-flick studies in mice are shown in brackets.^22,41^

Renewed interest in nitazenes has grown in recent years as the availability of these substances continues to rise. Despite this, detailed structure–activity relationship (SAR) studies have remained largely unexplored until recently. Notable contributions by researchers have begun to illuminate the pharmacological properties of these compounds,^6,12,13,21,42,43^ yet comparatively little attention has been given to their potential functional diversity or to the structural features that may enable MOR modulation rather than activation. In this context, recent work by Gomez et al. demonstrates that modest modifications within the nitazene scaffold can markedly alter MOR signaling profile, reinforcing the need for systematic SAR interrogation for MOR modulation.^44^ In particular, it remains unclear whether systematic modification of the nitazene scaffold can yield compounds capable of attenuating MOR signaling across multiple classes of synthetic opioids, an insight that could inform both overdose reversal strategies and broader opioid pharmacology.

In this study, we conducted the SAR analysis of nitazene analogs using integrated in vitro, in vivo, and computational approaches to define structural determinants of MOR modulatory activity. Using etonitazene as a reference, a series of nitazene analogs were designed and evaluated for their functional effects on MOR signaling and opioid induced antinociception. Our findings reveal that specific substitutions within the nitazene scaffold can confer selective, centrally active MOR modulation, including cross-class functional antagonism of epoxymorphinan-, phenylpiperidine-, and benzimidazole-based opioids. Collectively, this work reveals that the nitazene core is chemically versatile and may serve as a novel framework for the development of MOR modulators, highlighting the previously underappreciated potential of this scaffold for the rational design of mechanistically distinct MOR modulators.

## Results and Discussion

### Molecular Design

Previous studies have identified three key pharmacophoric features of nitazenes that significantly influence their potency and efficacy at the MOR: (1) the presence of a nitro substituent at the 5-position of the heteroaromatic benzimidazole ring, which appears optimal for high antinociceptive activity;^21,32,42,45^ (2) a tertiary amine side chain (such as ethane-1-amine) at the 1-position of the benzimidazole core, which is critical for MOR agonist potency;^21^ and (3) substitution at 4-position of the benzyl ring (preferably alkoxy) contributes to the MOR agonist potency^6,21,24,45–48^ while compounds with substituents at 2-position or 3-position of the benzyl ring were found to have reduced or no pronounced antinociceptive activity.^16^

To explore the SAR of the nitazene scaffold, a series of 2-benzylbenzimidazole analogs were designed with targeted modifications at all three of these key positions (Figure 2). These modifications included (1) 5-nitro group of the benzimidazole ring was eliminated to validate its critical role in the MOR function; (2) a range of tertiary amine chains including dimethylamine, diethylamine, pyrrolidine, and piperidine were introduced to explore the steric bulkiness of the tertiary amine on the compound’s pharmacological activity at the MOR; and (3) various substituents at 4-position of the benzyl ring were incorporated to gain insights into how substituent effects modulate the pharmacological profile of the nitazene scaffold. These included small alkyl groups (methyl, ethyl, isopropyl) to probe steric and hydrophobic influences; polar and electron-withdrawing/donating groups (cyano, fluoro, chloro, trifluoromethyl, ethoxy) to evaluate electronic and lipophilic contributions.

**Figure 2:**
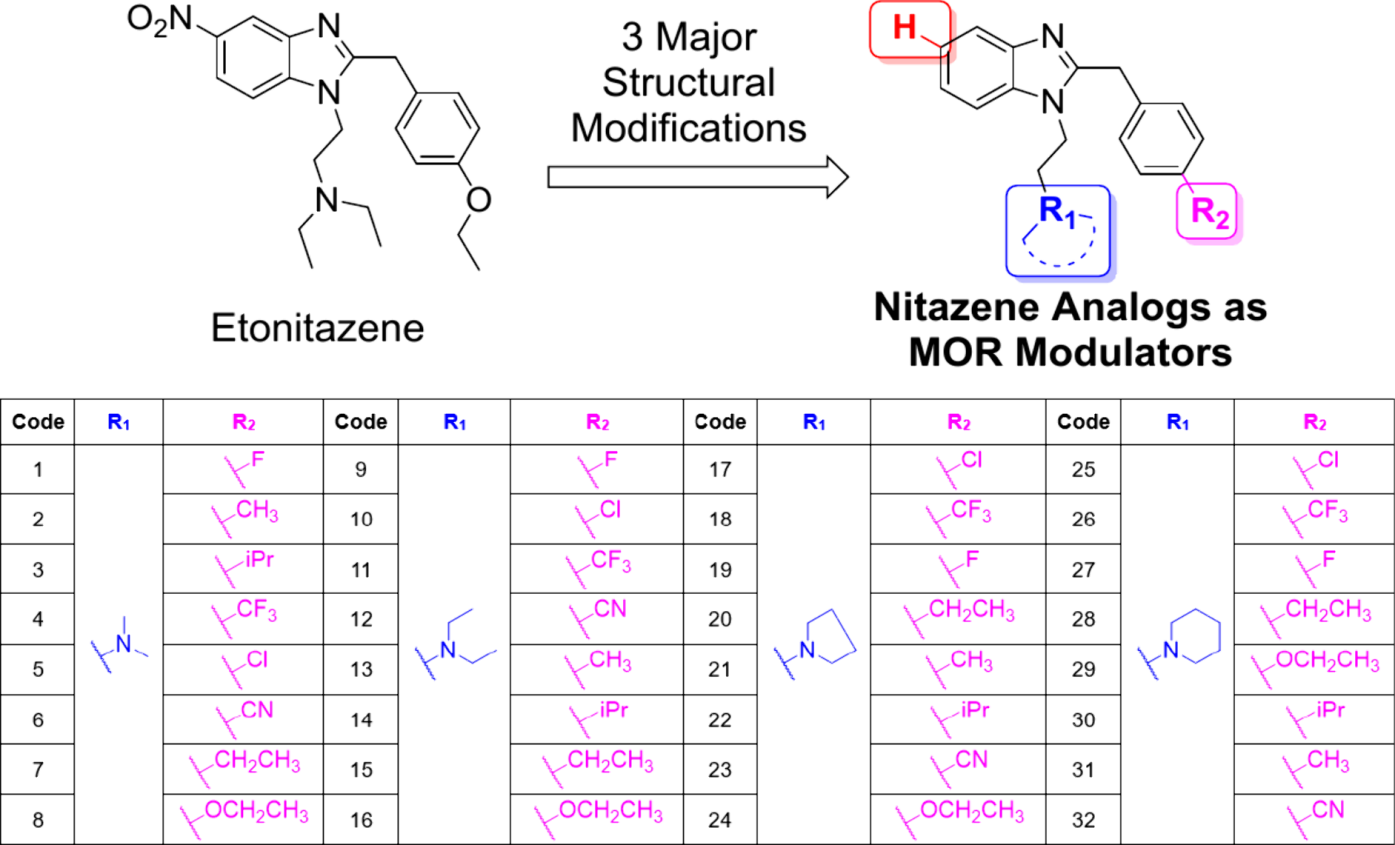
Structural architecture of nitazene analogs.

### Chemical Synthesis

Etonitazene and thirty-two nitazene derivatives (Figure 2) were synthesized by following previously reported synthetic procedures with modifications.^49^ Briefly, etonitazene was synthesized by coupling 2,4-dinitrobromobenzene with N,N’-diethylaminodiamine under basic conditions to obtain intermediate **1**, followed by selective reduction of the nitro group in the presence of aqueous ammonium sulfide to obtain intermediate **2**. This was finally coupled with 4-ethoxyphenylacetic acid to obtain etonitazene as a free base which was further converted to its hydrochloride salt (Scheme 1).

**Scheme 1:**
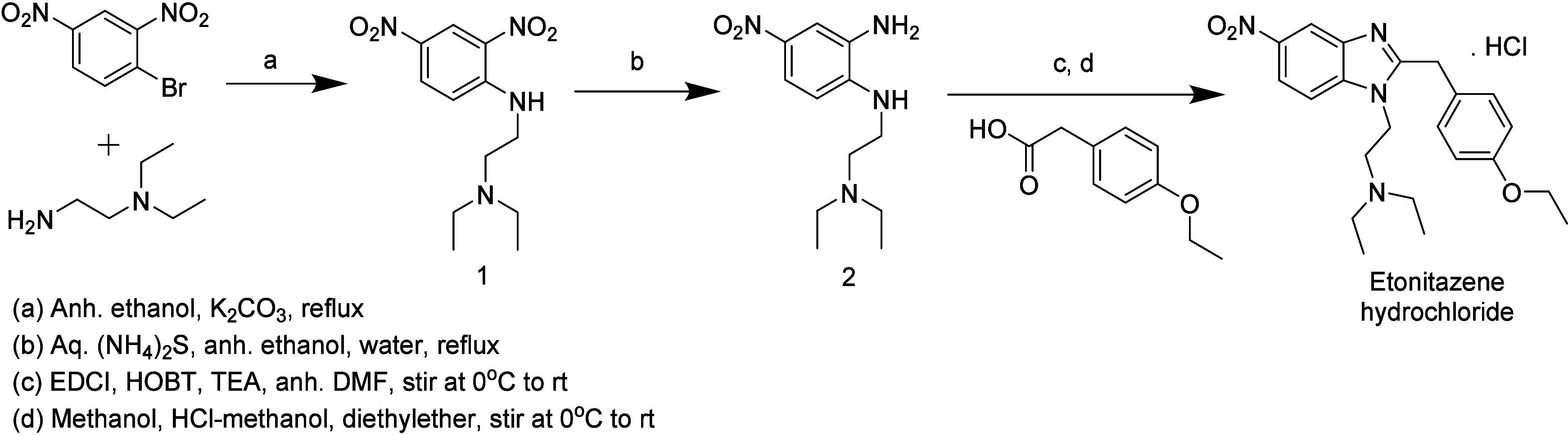
Synthetic Scheme of Etonitazene Hydrochloride

Thirty-two nitazene derivatives were synthesized by following a multistep synthetic route (Scheme 2). First, differently substituted N,N’-diethylamino side chains (1a-1d) were coupled with 2-fluoronitrobenzene under basic conditions to get intermediate **1** (2a-2d). In the second step, intermediate **1** was reduced by hydrogenation to get respective intermediate **2** (3a-3d). This intermediate was then coupled with differently substituted phenyl acetic acids (4a-4h) to obtain intermediates 5a-8h. In the last step, these intermediates were cyclized under acidic conditions to obtain the final compounds as free bases which were further converted to their hydrochloride salts (**1–32**). It should be noted all compounds are novel except compounds **5**, **8**, **10** (clodesnitazene), **13**, **16** (etodesnitazene), **24**, **25**, and **29** which have been previously reported.^16,22,41,42,50^ All target compounds were fully characterized before advancing to pharmacological assessments.

**Scheme 2:**
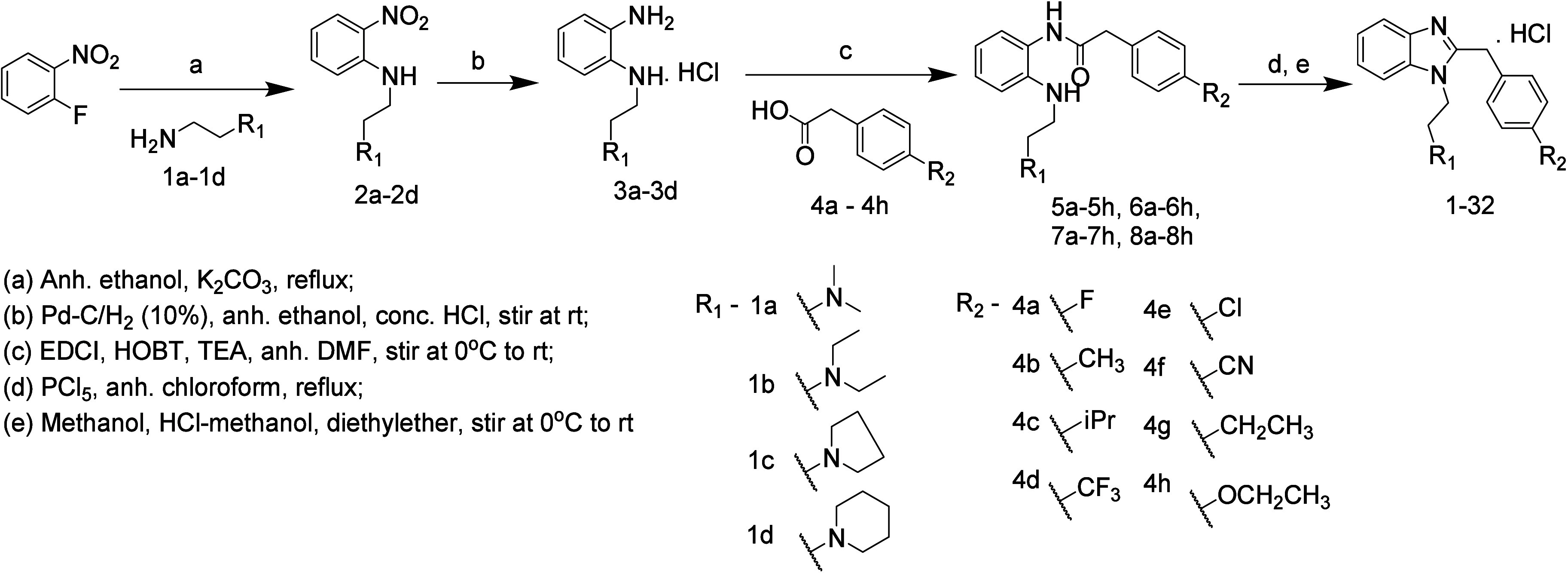
Synthetic Route of Nitazene Derivatives

### In Vitro MOR Functional Characterization

Calcium mobilization assays are widely used for the functional characterization of G-protein coupled receptors (GPCRs), as they enable real-time monitoring of intracellular calcium flux in response to receptor activation. This assay provides a dynamic and quantifiable readout of receptor-mediated signaling, making it particularly valuable for investigating ligand–receptor interactions.^51,52^ Hence, it was utilized as the primary assay for functional characterization of the novel nitazene derivatives at the MOR. In this study, to facilitate calcium-dependent signaling at MOR, which canonically couples to Gi/o proteins, a chimeric Gqi4 protein was coexpressed in monoclonal Chinese hamster ovary (CHO) cells stably expressing MOR.^53^ This redirection of signaling allowed the detection of both agonist-induced calcium flux and antagonist-mediated inhibition.

All thirty-two derivatives were first screened at a single concentration of 10 μM to evaluate adequate receptor engagement and facilitate the detection of agonistic activity of these derivatives at the MOR (Figure 3). The two MOR agonists, DAMGO and etonitazene were used as positive controls at 10 μM concentrations and data of the derivatives were represented % relative to 10 μM DAMGO (Table S1). DAMGO was used as a reference of a full agonist to characterize compounds for their MOR agonist functional activity and prioritize compounds with reduced agonist efficacy for further studies. Overall, all compounds showed a diverse range of response in inducing the Ca^2+^ flux. Similar to etonitazene, four out of the thirty-two compounds viz. compound **8, 20, 22,** and **24** elicited Ca^2+^ flux comparable to the full MOR agonist DAMGO (Figure 3), suggesting their potential agonistic activity at the MOR. Remaining twenty-eight compounds induced varying degrees of Ca^2+^ flux relative to DAMGO (Figure 3), indicating their potential to modulate the MOR signaling at different extent. Among the twenty-eight, 14 compounds viz. **1, 2, 4, 5, 6, 9, 13, 17, 19, 21, 25, 26, 27,** and **31** that demonstrated the most significant reductions in induction of Ca^2+^ flux (****P < 0.0001, compared to 10 μM DAMGO) were studied further for their potential to modulate the effect of MOR agonists.

**Figure 3:**
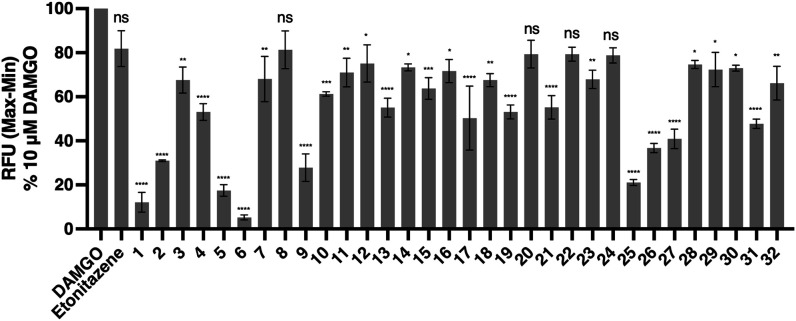
In vitro calcium mobilization results of nitazene analogs at single concentration of 10 μM at the MOR. Etonitazene (10 μM) and DAMGO (10 μM) were used as positive controls. Data are presented as mean values ± SEM; *n* = 3. *P < 0.03, **P < 0.002, ***P < 0.0002, ****P < 0.0001. ns – not significant.

All 14 compounds were further assessed for their potential to antagonize the MOR full agonist DAMGO at a concentration of 10 μM. DAMGO was used at its EC_80_ concentration of 500 nM. Naltrexone (NTX), a well-established MOR antagonist, was used as a positive control at the same concentration of 10 μM and the data were represented as % relative to DAMGO. As expected, NTX significantly attenuated DAMGO-induced calcium mobilization (12.25% of DAMGO). Six out of 14 nitazene derivatives viz. **4, 9, 13, 17, 21,** and **26** significantly reduced DAMGO-induced calcium responses, as reflected by decrease in the calcium mobilization (Figure 4). These findings suggest that these six compounds show potential to antagonize DAMGO-mediated calcium flux.

**Figure 4:**
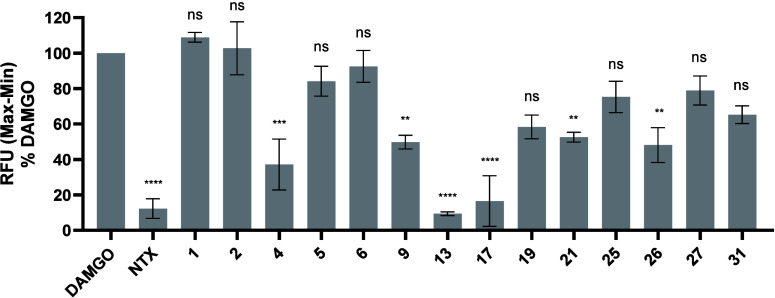
In vitro calcium mobilization results of nitazene analogs at a single concentration of 10 μM against DAMGO (500 nM) in the MOR cells. NTX (10 μM) was used as positive control. Data are presented as mean values ± SEM; *n* = 3; **P < 0.002, ***P < 0.0002; ****P < 0.0001, ns – not significant.

First, agonist-mode concentration response curves were obtained for the identified six compounds (Figure S1) to evaluate their intrinsic functional efficacy. The efficacy values presented in Figure S1 were determined from independently generated full concentration–response curve analyses. The data points were normalized to the maximal DAMGO response obtained under the same experiment conditions. All compounds (**4, 9, 13, 17, 21,** and **26**) demonstrated submaximal responses relative to that of maximal DAMGO [*E*_max_ ≈ 17–33% relative to maximal DAMGO], thereby indicating that these ligands may function as low-efficacy partial agonists. Following this, full concentration–response curves were generated for these six compounds to determine their potency in antagonizing DAMGO, fentanyl and etonitazene (Figure S2). NTX was incorporated as a positive control while the MOR agonists were applied at their respective EC_80_ concentrations. Throughout the experimental procedures, all tested compounds remained fully soluble, with no evidence of precipitation or turbidity observed at concentrations up to 500 μM. As shown in Table 1, all six nitazene derivatives significantly antagonized the effects of three distinct classes of MOR agonists, viz. opioid peptide (DAMGO), phenylpiperidine (fentanyl), and 2-benzylbenzimidazole (etonitazene). Among the tested analogs, compounds **4**, **13**, **17**, and **21** showed highest potency in antagonizing DAMGO-induced calcium signaling, with IC_50_ values in the single-digit micromolar range. In contrast, compounds **9** and **26** exhibited lower potency, showing IC_50_ values in the two-digit micromolar range. However, all compounds demonstrated stronger antagonism potential with single digit micromolar IC_50_ values against fentanyl induced Ca^2+^ flux. Notably, compounds **9** and **26** exhibited a 4-fold increase in potency against fentanyl compared to DAMGO.

**Table 1:**
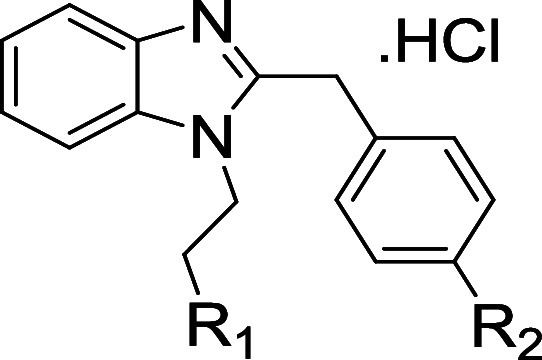

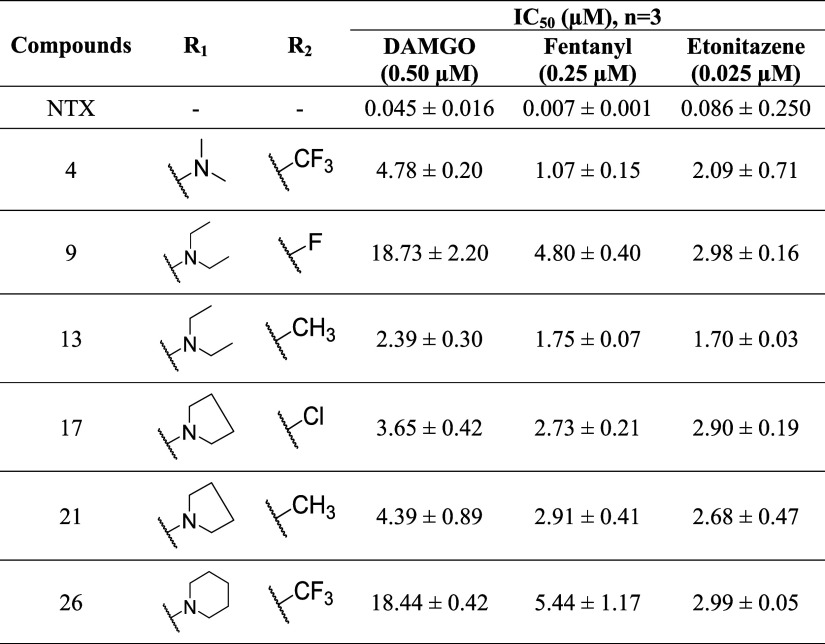
Calcium Mobilization Assay Results of Nitazene Analogs at the MOR

When evaluated against etonitazene, all six compounds showed single digit micromolar potency, confirming their potential to attenuate etonitazene-mediated calcium mobilization. Interestingly, compounds **9** and **26** were found to be 6-fold more potent in blocking etonitazene-induced responses compared to DAMGO. Thus, taken together, compounds **4**, **13**, **17**, and **21** displayed consistent, broad-spectrum of activity across all three MOR agonists. Compounds **9** and **26** showed preferential potency toward fentanyl and etonitazene as compared to DAMGO. These findings provided proof-of-concept that the nitazene scaffold is chemically tunable and may be further optimized to achieve selective modulation of functional activity against distinct classes of MOR agonists.

Collectively, these results reveal distinct SAR trends and demonstrate the potential of the nitazene scaffold to modulate the MOR function. It was observed that substitution at 4-position of the benzyl ring (Figure 2, R_2_) was particularly impactful, where compounds with electron-donating substituents like -^i^Pr, −CH_2_CH_3_, and -OCH_2_CH_3_ (e.g., **8, 20, 22,** and **24**) demonstrated statistically similar agonist activity as DAMGO at a concentration of 10 μM. Although the 5-nitro group on the benzimidazole ring was eliminated, several compounds with electron-donating substituents at the R_2_ position still exhibited MOR agonistic activity, suggesting that substituents at this position may play a critical role in modulating functional activity at the MOR. Meanwhile, the six identified hits (**4, 9, 13, 17, 21,** and **26**) incorporate either electron-withdrawing groups like −CF_3_, -F, -Cl or smaller electron donating group such as −CH_3_ at the R_2_ position. At the tertiary amine side chain (Figure 2, R_1_), no clear structure–activity trend was observed, as compounds bearing either sterically smaller groups such as −N­(CH_3_)_2_ and −N­(C_2_H_5_)_2_, or bulkier cyclic amines like pyrrolidine and piperidine, elicited similar inhibition in the functional response. Overall, these findings highlight the functional tunability of the nitazene scaffold, where strategic modifications at R_2_ may influence functional profiles spanning from agonism to antagonist-like behavior.

### In Vivo Characterization

In parallel to the in vitro Ca-mobilization studies, in vivo warm water tail immersion (WWTI) assays were conducted using Swiss Webster mice to assess their pharmacological activity in a more complex biological system and evaluate their SAR in a physiologically relevant system. This dual approach enabled assessment of SAR at both the cellular level and within the context of whole-body physiology, helping to ensure that no pharmacologically relevant analogs were overlooked. The WWTI assay has been used for the assessment of thermal pain associated with the antinociceptive properties of opioids. This pain sensitivity assay involves immersing the mouse’s tail in warm water, and measuring the tail withdrawal latency (i.e., the amount of time taken before the tail is flicked in response to pain).^54,55^ The longer duration corresponds to a larger percentage of maximum potential effect (%MPE), indicating stronger antinociception effects. A cutoff time of 10 s is applied to prevent the possibility of tissue damage.

First, the antinociception potential of the thirty-two compounds was examined to identify any potential opioid receptor agonists. A single dose of 10 mg/kg of each test compound was administered subcutaneously (s.c.), and the withdrawal latency was measured after 20 min. It must be noted that, no solubility issues or evidence of precipitation in vehicle were observed at the tested dose for any of the tested compounds. Three positive controls were included in this assay: etonitazene (0.1 mg/kg), fentanyl (10 mg/kg), and morphine (10 mg/kg). Etonitazene, a MOR agonist with potency far exceeding that of morphine, was administered at a significantly lower dose to minimize the risk of toxicity while still achieving robust pharmacological effects. All positive controls produced 100% MPE, confirming full antinociceptive efficacy under the assay conditions. Figure 5 demonstrates that majority of the synthesized analogs did not display significant antinociceptive effects compared to vehicle. Statistically significant antinociception was displayed by six compounds viz. **8, 16, 20, 22, 24,** and **29**, as evident from their high % MPE (comparable to morphine and etonitazene). Notably, compound **16** (etodesnitazene) produced antinociception effects (high %MPE) at 10 mg/kg similar to morphine and etonitazene, consistent with its classification as a highly potent opioid.^16^ Importantly, these results were also in line with those observed from the in vitro studies (Figure 3). Structurally these six compounds incorporated electron donating groups (such as -^i^Pr, -CH_2_CH_3_, and -OCH_2_CH_3_) at the R_2_ position (Figure 2) suggesting that these substituents may play a significant role in mediating the observed in vivo antinociceptive effects. The remaining twenty-six analogs exhibited no significant antinociceptive activity and were therefore evaluated for their potential to block the antinociceptive effects of opioid agonists.

**Figure 5:**
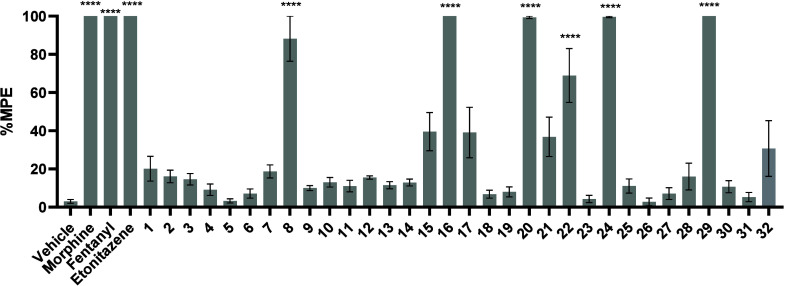
WWTI assay results of nitazene analogs at a single dose of 10 mg/kg (s.c.). Morphine (10 mg/kg, s.c.), fentanyl (10 mg/kg, s.c.), and etonitazene (0.1 mg/kg, s.c.) were used as positive controls and vehicle as the negative control. Data are presented as mean values ± SEM, *n* = 6. ****P < 0.0001, compared to vehicles.

The potential of these analogs at a single dose of 10 mg/kg s.c. to counteract the antinociceptive effects of morphine (10 mg/kg, s.c.) was investigated first. Figure 6 shows that among the tested derivatives, five compounds **10, 25, 26, 27,** and **30** significantly antagonized the antinociceptive effects of morphine, demonstrating their potential to modulate central nervous system mediated antinociception. These five identified hits were further investigated for their ability to counteract the antinociceptive effects of two synthetic opioids, fentanyl (0.1 mg/kg) and etonitazene (0.025 mg/kg) in vivo (Figure 7A,B). The specific doses of both agonists were determined based on their respective ED_50_ values [fentanyl (0.056 mg/kg) and etonitazene (0.007 mg/kg)].^26,56^ Notably, among the tested derivatives, compounds **26** and **30** demonstrated significant antagonism of both fentanyl- and etonitazene-induced antinociception, underscoring their potential to produce cross-class functional antagonism in vivo.

**Figure 6:**
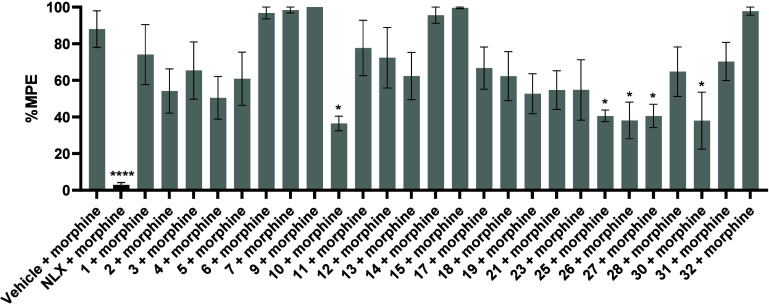
WWTI antagonism assay results of analogs at a single dose of 10 mg/kg (s.c.) in the presence of morphine (10 mg/kg, s.c.). NLX and vehicle were used as controls. Data are presented as mean values ± SD, *n* = 6. *P < 0.05 compared to vehicle.

**Figure 7:**
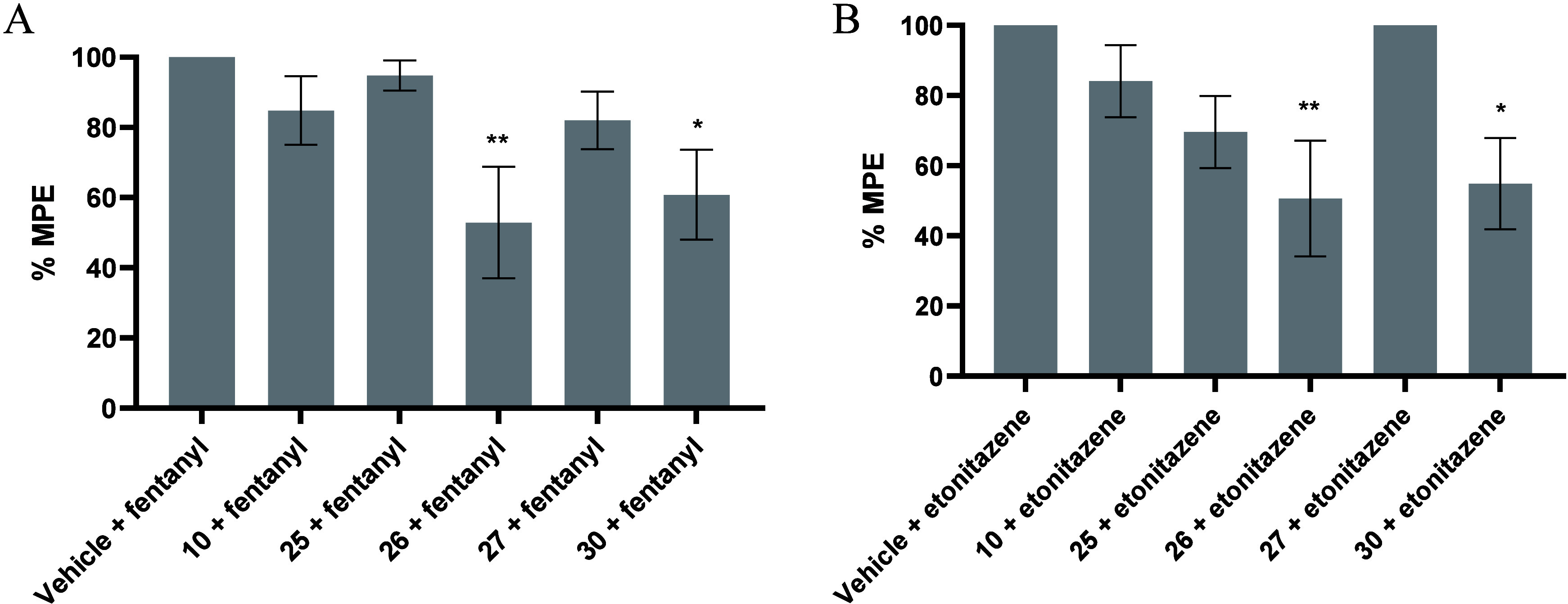
WWTI antagonism assay results of analogs at a single dose of 10 mg/kg (s.c.) in the presence of (A) fentanyl (0.1 mg/kg, s.c.) and (B) etonitazene (0.025 mg/kg, s.c.). Fentanyl and etonitazene were used as positive controls and saline as the negative control. Data are presented as mean values ± SD, *n* = 6. *P < 0.05 and **P < 0.01 compared to vehicles.

While in vitro calcium flux studies identified several nitazene derivatives, including compounds **4, 9, 13, 17, 21,** and **26**, that antagonized MOR agonists, only compound **26** exhibited consistent antagonistic activity in vivo. The reduced in vivo efficacy observed for other derivatives (compounds **4, 9, 13, 17,** and **21**) may be attributed to common challenges such as limited bioavailability, rapid metabolism, or restricted blood-brain barrier (BBB) permeability. Although many nitazene analogs are generally lipophilic and known to cross the BBB—often through the formation of active metabolites—even subtle structural modifications may lead to divergent pharmacokinetic outcomes. Recent in vitro studies in human hepatocytes have shown that nitazenes undergo extensive phase I and phase II metabolism, including N dealkylation and glucuronidation, with distinct clearance patterns across analogs, potentially affecting both bioavailability and CNS penetration.^57^ Furthermore, Jadhav et al. have demonstrated that several nitazenes undergo extensive metabolism via cytochrome P450 enzymes, potentially limiting systemic exposure and CNS activity.^58^ Although dedicated ADME studies were not performed in the present work, the known metabolic liabilities associated with this class of compounds offer a plausible explanation for their reduced in vivo efficacy and opens a window for investigation in future studies.

Interestingly, compound **30** demonstrated significant attenuation of the antinociceptive effects of morphine, fentanyl, and etonitazene in vivo (Figures 6 and 7), while exhibiting partial agonist activity in the in vitro functional assays (% *E*_max_ 55.88 ± 3.56, Figure S1), indicating potential differences in efficacy under physiological and cellular assay conditions. This observation may stem from factors not captured in the cellular assay system, such as favorable pharmacokinetic properties, the formation of active metabolites, or indirect mechanisms and off-target effects that become evident only in a complex physiological environment. These findings underscore the importance of complementary in vitro and in vivo approaches to capture the full pharmacological potential of novel scaffolds. Further explorations are underway to investigate the mechanism of action of compound **30** and other similar compounds, including possible alternative receptor interactions or metabolic contributions, to better define its unique activity profile.

Further, following the single dose screening, compound **26,** was selected for detailed dose response studies against three different classes of MOR agonists, viz. epoxymorphinan (morphine), phenylpiperidine (fentanyl), 2-benzylbenzimidazole (etonitazene). The compound was evaluated at doses up to 64 mg/kg and remained fully soluble under the experimental conditions, with no evidence of precipitation or formulation instability. As shown in Figure 8, compound **26** effectively blocked the antinociceptive effects of three pharmacologically distinct MOR agonists—morphine, fentanyl, and etonitazene—in a dose-dependent manner, as reflected by their AD_50_ (anti-antinociception) values. The compound exhibited the greatest potency against morphine (AD_50_: 2.88 mg/kg), followed by fentanyl (AD_50_: 6.41 mg/kg), and etonitazene (AD_50_: 16.77 mg/kg). Compound **26** did not antagonize any of the three agonists—morphine, fentanyl, or etonitazene—at a dose of 1 mg/kg (Figure 8A–C). However, significant antagonism was observed against all three agonists at higher doses (≥3.2 mg/kg). To our knowledge, this is the first demonstration of a behaviorally active MOR modulator based on the nitazene (2-benzylbenzimidazole) scaffold capable of attenuating the effects of three structurally and pharmacologically diverse opioid agonists. These results not only validate the functional potential of compound **26** but also establish a compelling proof-of-concept that the nitazene core is chemically tunable for functional modulation of the MOR. Taken together, these findings expand the accessible chemical space for opioid receptor ligands and position nitazenes as a promising scaffold for future therapeutic exploration targeting opioid-related disorders.

**Figure 8:**
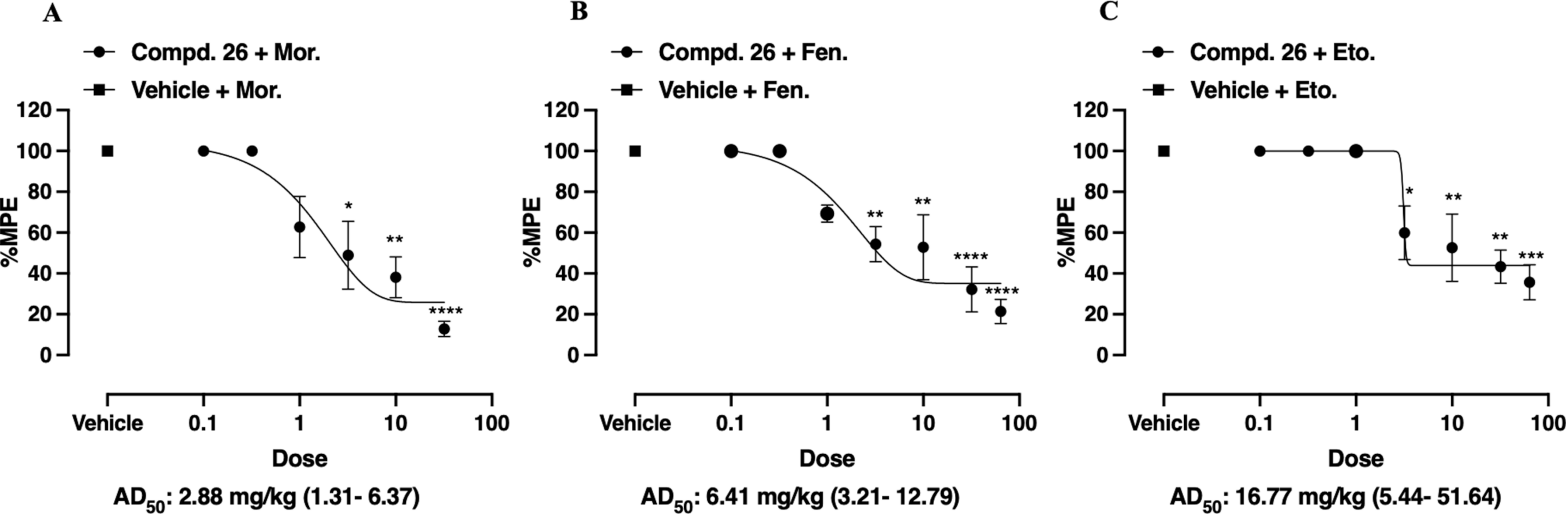
Dose response antagonism studies of compound **26** in mice against (A) morphine (10 mg/kg, s.c.), (B) fentanyl (0.1 mg/kg, s.c.), **(C**) etonitazene (0.025 mg/kg, s.c.). Saline (vehicle) was used as negative control. Data are presented as mean ± SEM *n* = 6. *P < 0.05, **P < 0.01, ***P < 0.001, ****P < 0.0001 compared to vehicle.

### In Vitro Radioligand Binding Data

To confirm MOR engagement and to assess receptor level selectivity, radioligand binding assays were conducted for the ten nitazene analogs identified as hits from the in vitro functional and in vivo behavioral studies. Binding affinities were determined using CHO cell membrane preparations stably expressing the classical opioid receptors MOR, KOR, or DOR, according to previously established protocols.^59–61^ [^3^H]­Naloxone was used for labeling the MOR, whereas [^3^H]­diprenorphine was employed for KOR and DOR.

As summarized in Table 2 all analogs exhibited submicromolar binding affinity at the MOR, confirming MOR engagement across the series, with several-fold selectivity for MOR over KOR and DOR. Notably selectivity over DOR was generally greater than selectivity over KOR. Compounds **4**, **9**, **10**, **13**, and **17** displayed comparable selectivity ratios for KOR/MOR and DOR/MOR, whereas compounds **21**, **26**, and **27** demonstrated a consistent trend toward MOR selectivity across other two receptors. Interestingly, compounds **25** and **30** exhibited similar selectivity for MOR over KOR but differed substantially in their affinities toward DOR, suggesting their marked differences in receptor recognition. The distinct binding profiles of these analogs align with their corresponding lower potencies in the in vitro functional assays (Table 1) and reduced efficacies in the in vivo behavioral studies (Figure 7), reinforcing the connection between receptor affinity and pharmacological response.

**Table 2:**
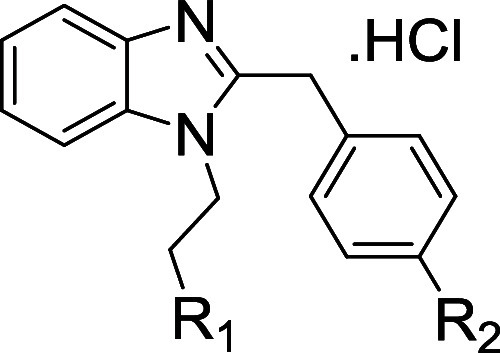

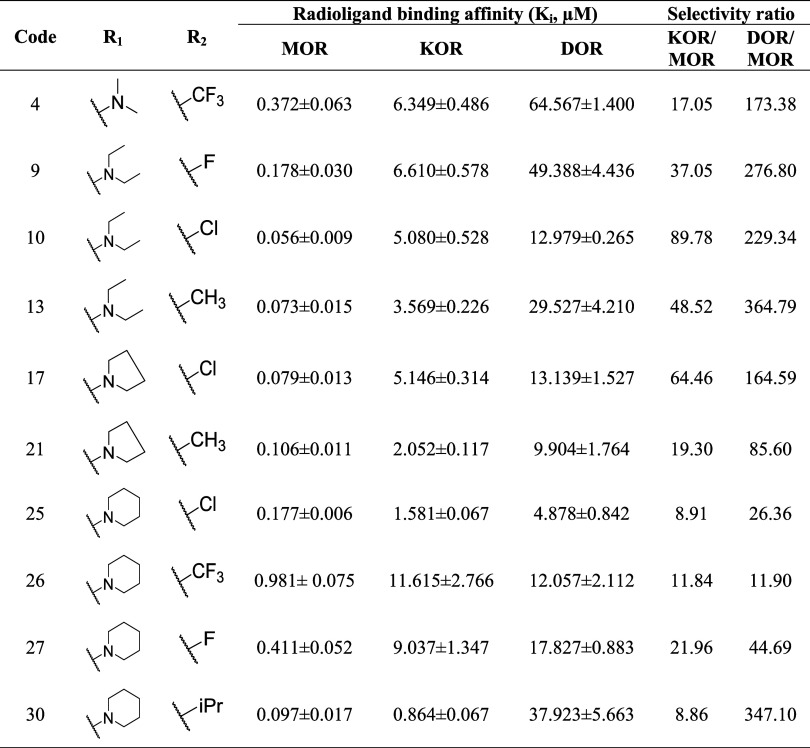
Binding Affinity and Selectivity Results of Nitazene Analogs

### In Vitro ADME Characterization

In order to provide additional insights into the pharmacokinetic properties of the nitazene scaffold that may influence in vivo activity and CNS penetration, in vitro ADME studies were conducted to evaluate the metabolic stability and permeability of compound **26**. First, 1 μM of compound **26** was incubated in human and mouse liver S9 fractions to study the clearance mechanism by the Phase II glucuronidation reactions mediated via UDP-glucuronosyltransferase (UGT) enzymes and assess for rapid evaluation of overall liver metabolism in these two species. In human liver S9, compound **26** demonstrated moderate metabolic stability, with a half-life of 25.6 min and intrinsic clearance (Cl_int_) of 27.2 μL/min/mg, suggesting a manageable rate of hepatic metabolism compatible with systemic exposure (Table 3). These values compare favorably to those reported for nitazene agonists like butonitazene, isotonitazene and protonitazene (Table 3); however, cross-study comparisons should be interpreted with caution due to potential differences in the experimental conditions.^58^ In contrast, mouse liver S9 fractions showed significantly faster metabolism of compound **26**, with a half-life of 5.2 min and a Cl_int_ of 132.8 μL/min/mg, indicating species-dependent differences in hepatic clearance that may contribute to interspecies variability in pharmacokinetics and systemic exposure.

**Table 3:** Intrinsic Clearance of Compound **26** in Human and Mouse Liver Microsomes with Cofactors[Table-fn t3fn3]

	Human (liver, S9)		Mouse, CD-1 (liver, S9)	
Compound	*t*_1/2_ (min)	CL_int_(μL/min/mg)	*t*_1/2_ (min)	CL_int_(μL/min/mg)
Compd. **26**	25.6 ± 1.9	27.2	5.2 ± 0.1	132.8
Butonitazene^b^	8	217	ND	ND
Isotonitazene^b^	10	139	ND	ND
Protonitazene^b^	9	150	ND	ND
Estrone	61.4 ± 2.1	11.3	22.3 ± 2.8	31.3
Terfenadine	77 ± 8.1	9.0	38.6 ± 5.2	18.1

a Data was reported in ref 58.

b ND: not determined.

To further evaluate the potential involvement of active efflux mechanisms that could limit absorption or CNS penetration of compound **26**, bidirectional Caco-2 permeability studies were performed in the presence of selective transporter inhibitors using digoxin (P-glycoprotein, P-gp substrate)^62^ and estrone sulfate (breast cancer resistance protein, BCRP substrate)^63^ as positive controls (Table 4). Under baseline conditions, compound **26** exhibited moderate passive permeability (A→B: 6.1 × 10^–6^ cm/s) with an efflux ratio (E_R_) of 1.1­(B→A/A→B), suggesting it might not be a significant substrate for efflux transporters. In contrast, digoxin (E_R_= 35) and estrone sulfate (E_R_= 36) showed strong efflux at baseline, as expected for classical P-gp and BCRP substrates, respectively.

**Table 4:** Caco-2 Permeability and Transporter Interaction of Compound **26** [Table-fn t4fn2]

	Permeability (× 10^–6^ cm/s)		
	Compd. 26	Digoxin	Estrone sulfate
A → B	6.1 ± 0.2	0.3 ± 0.04	0.4 ± 0.02
B → A	6.6 ± 0.2	10.6 ± 1.3	14.5 ± 2.1
A → B + verapamil	12.7 ± 0.5	1.3 ± 0.2	NA
B → A + verapamil	6.4 ± 0.02	2.0 ± 0.2	NA
A → B + KO143	10.8 ± 0.2	NA	1.5 ± 0.01
B → A + KO143	3.7 ± 0.03	NA	3.7 ± 0.6

a A → B (apical to basolateral) and B → A (basolateral to apical) flux. Verapamil (P-gp inhibitor) and KO143 (BCRP inhibitor) were used to assess transporter contributions. NA: not applicable.

It was observed that coincubation with the P-gp inhibitor verapamil^64^ increased the A→B permeability of compound **26** from 6.1 to 12.7 × 10^–6^ cm/s while retaining B→A permeability thus lowering the P-gp efflux ratio to 0.5. These findings suggest a minor interaction with P-gp likely insufficient to significantly impair permeability. Similarly, incubation with the selective BCRP inhibitor KO143 ^65^ increased the A→B permeability to 10.8 × 10^–6^ cm/s and reduced B→A permeability to 3.7 × 10^–6^ cm/s, resulting in a BCRP efflux ratio of 0.34. Collectively, these data indicated that compound **26** exhibits low susceptibility to both P-gp and BCRP-mediated efflux, compared to the strong inhibition effects observed with the positive control substrates digoxin and estrone sulfate. Collectively, the ADME profile of compound **26** suggested favorable metabolic and permeability properties supportive of systemic exposure and CNS penetration, which are critical parameters for centrally acting agents. Importantly, these data may help explain, in part, the differential in vivo activity observed across the series and provide a foundation for further chemical optimization to balance receptor potency, metabolic stability, and pharmacokinetic properties.

### Molecular Modeling Studies of the Nitazene Binding at the Mu-Opioid Receptor

To gain insights into how nitazene-based ligands bind to the MOR in the context of their differentiated functions on the MOR, molecular docking studies were conducted for the ultrapotent MOR agonist etonitazene and its functionally distinct analog compound **26**.

Docking of etonitazene in the active MOR resulted in two plausible, high scoring binding poses (Figure 9) that occupied the orthosteric binding pocket but differed by an ∼180° flipped orientation. In both conformations, etonitazene interacted with the canonical orthosteric binding pocket composed of residues D147, Y148, M151, W293, I296, H297, W318 and I322 similar to that seen for morphine (PDB ID 8EF6) and fentanyl (PDB ID 8EF5) (pink surface, Figure 9).^66^ In binding pose 1 (Figure 9A), the 5-nitro group of etonitazene engaged in hydrogen bonding interactions (2.8 Å) with H297, located in transmembrane (TM) 6. This residue has been reported to play a ligand-dependent role in stabilizing MOR-ligand interactions and influencing receptor activation, particularly through its ability to participate in polar contacts and modulate receptor conformational states.^67,68^ In binding pose 2 (Figure 9B), due to the flip in conformation, the hydrogen binding interaction was replaced by a π–π stacking interaction observed between the aromatic ring of the 2-(4’-ethoxybenzyl) group and W318 in TM7. W318 is a critical determinant of MOR binding affinity; mutation of this residue has been reported to abolish opioid binding almost entirely.^69^

**Figure 9:**
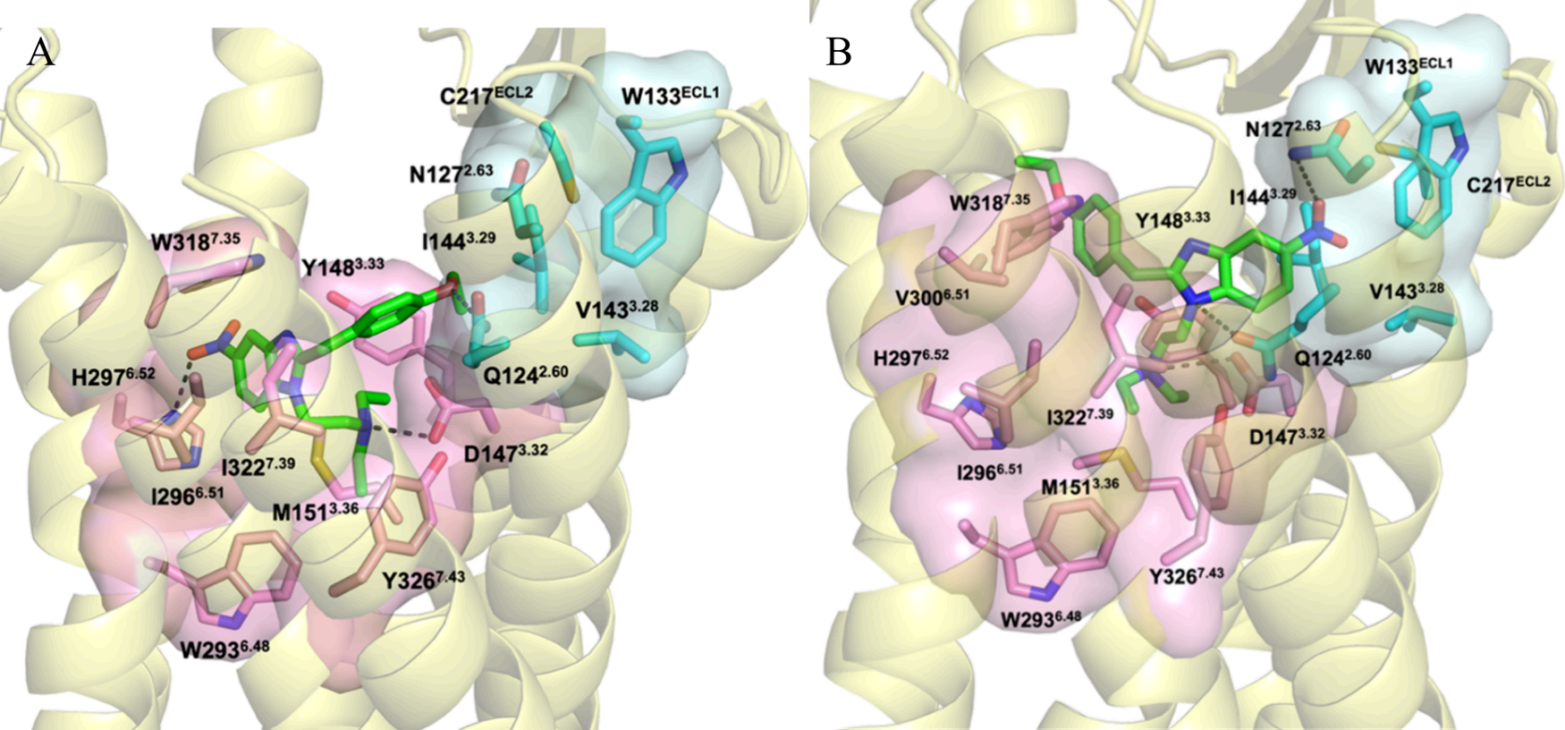
Two highest scored docking poses: (A) binding pose 1 and (B) binding pose 2 of etonitazene (green sticks) in the active MOR (PDB ID 8EF5; pale yellow cartoon). The orthosteric binding site is shown as pink surface while the novel secondary binding site is shown as cyan surface with key amino acids as sticks.

Interestingly, distinct from morphine, etonitazene extended into a secondary hydrophobic pocket formed among TM2, TM3, extracellular loop (ECL) 1 and ECL2 and comprising of residues Q124, N127, W133, V143, I144 and C217 (cyan surface, Figure 9). In pose 1, the 2-(4’-ethoxybenzyl) group of etonitazene showed potential hydrogen bonding interactions (3.7 Å) with Q124 in TM2 while in pose 2, the 5-nitro group and the benzimidazole nitrogen formed hydrogen bonds with N127 (3.7 Å) and Q124 (3.7 Å), respectively. These residues have been implicated in ligand recognition and selectivity.^70,71^ Additionally, it is worth noting that the interactions identified in this study align with those proposed by Clayton et al. in their recent molecular modeling investigations of nitazene agonists.^72^ During the submission and revision of this manuscript, a cryo-EM structure of a nitazene-bound MOR complex was reported, revealing a comparable active-state binding mode, including the conserved interaction with D147 and extension of the nitazene scaffold toward the TM2/ECL region (Q124/N127), thereby providing structural validation of the docking poses predicted here.^44^

While molecular docking studies cannot unambiguously define a single bound conformation, pose 2 was highlighted for further discussion because it positioned both the 5-nitro group and the benzimidazole nitrogen toward the Q124/N127-containing secondary pocket, enabling additional polar contacts relative to pose 1. In conjunction with prior SAR studies showing marked losses in activity upon modification or removal of the 5-nitro or 2-(4′-alkoxybenzyl) substituents,^16,73^ these observations suggest that interactions in this region may contribute to high MOR affinity; however, this interpretation remains hypothesis-generating and will require further experimental validation.

To enable a direct comparison with etonitazene, compound **26** was docked into the active MOR conformation using identical parameters. In addition, given that compound **26** antagonized MOR agonist responses in vitro and in vivo, docking into the inactive MOR conformation was also explored.

In the active MOR, compound **26** adopted an orientation similar to binding pose 2 of etonitazene (Figure 9B), engaging the orthosteric site and forming contacts with residues D147, M151, W293, I296, H297, I322, and Y326 (Figure 10A). The −CF_3_ substituent was positioned within a hydrophobic region formed by I296, H297, and I322. However, in contrast to etonitazene, compound **26** did not extend into the secondary pocket adjacent to TM2 and ECL regions, suggesting that substitution at the benzyl moiety as well as at the 5-position of the benzimidazole core may influence access to auxiliary cavities as well as MOR binding. When docked into the inactive MOR, compound **26** displayed a distinct binding orientation while maintaining contacts within the orthosteric site (Figure 10B). In this conformation, the 2-benzyl substituent projected toward a different hydrophobic cavity formed by residues K233, V236, F237, V300, and I301. Within this pocket, the −CF_3_ group engaged hydrophobic contacts with V300, V236, and F237. This region has been implicated in inactive-state MOR structures, and residue K233 has been reported to form a covalent interaction with the irreversible antagonist β-FNA in the inactive receptor conformation.^68^

**Figure 10:**
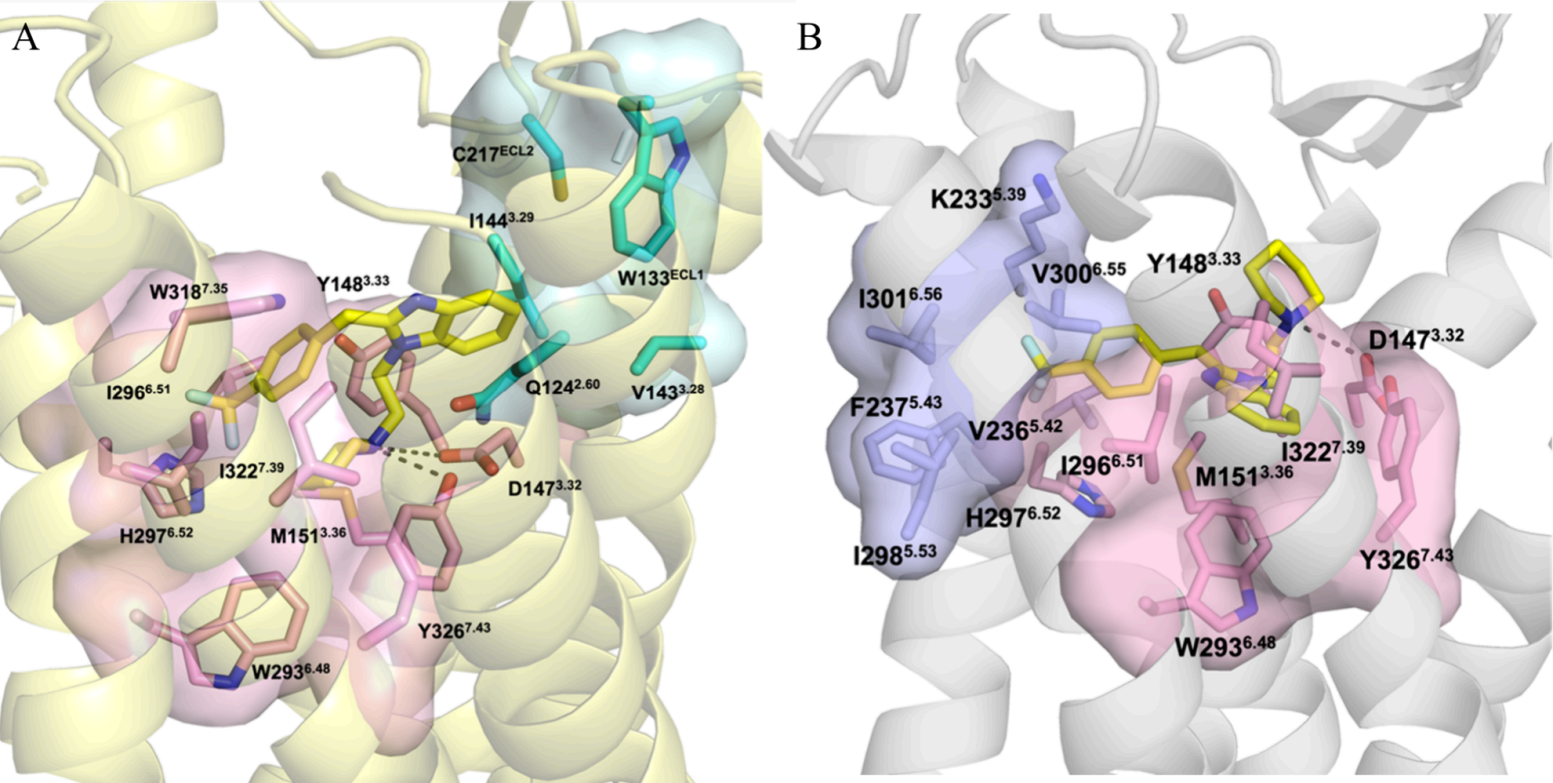
Highest scored docking pose of compound **26** (yellow sticks) in the (A) active MOR (PDB ID 8EF5; pale yellow cartoon) and (B) inactive MOR (PDB ID 9BJK; gray cartoon). The orthosteric binding site is shown as pink surface while the secondary binding sites are shown as cyan and blue surfaces with key amino acids as sticks.

Direct comparison of the representative poses of compound **26** in the active and inactive MOR conformations (Figure 10A,B) indicated that while the benzimidazole core occupied a similar position within the orthosteric pocket in both states, the orientation of the benzyl substituent differed substantially, resulting in engagement of distinct peripheral cavities. These observations suggest that compound **26** may be capable of adopting receptor-state-dependent binding modes, which could be consistent with its mixed functional profile observed experimentally. Taken together, these molecular modeling results are best interpreted as hypothesis-generating and provide a structural framework for considering how modest scaffold modifications within the nitazene series may influence receptor-state-dependent binding orientations and engagement of peripheral cavities. Such effects could help rationalize broader SAR trends observed for closely related analogs in this series, including compounds that display divergent pharmacological profiles despite minimal chemical changes, although dedicated experimental studies will be required to rigorously test these possibilities.

## Conclusions

Overall, this study presents a SAR analysis of 2-benzylbenzimidazole (nitazene) analogs, demonstrating that a scaffold associated with ultrapotent MOR agonism can be systematically modified to yield selective MOR modulators capable of spanning a broad functional activity spectrum, from agonism to low-efficacy partial agonism and in vivo functional antagonism. In vitro assays identified six compounds that effectively reduced the responses induced by MOR agonists, DAMGO, fentanyl, and etonitazene, with differences in potency suggesting potential selectivity among opioid classes. In vivo, five compounds significantly attenuated morphine (an epoxymorphinan)-induced antinociception, while two analogs (compounds **26** and **30**) effectively blocked the antinociceptive effects of both fentanyl (a phenylpiperidine) and etonitazene (a 2-benzylbenzimidazole), demonstrating cross-class antagonism. The SAR analysis of these identified MOR modulators suggests that specific structural features of the nitazene scaffold may contribute to their modulatory activity. In particular, para-substitution on the 2-benzyl ring (R_2_) with electron-withdrawing groups appears to correlate with functional activity, while the contribution of R_1_ substituents was assay dependent, with increased steric bulk correlating with behavioral efficacy in vivo. All identified hits exhibited submicromolar binding affinities and substantially higher selectivity for the MOR over the KOR and DOR. Molecular modeling studies of etonitazene and compound **26** provide a hypothesis for how subtle scaffold modifications may alter binding orientation, potentially extending toward secondary pockets beyond the canonical orthosteric site. These models are intended to guide future experimental interrogation rather than to define mechanism. Lastly, compound **26** exhibited an ADME profile indicative of good metabolic stability and permeability, supporting its potential for systemic availability and CNS penetration, key attributes for centrally acting therapeutics. Overall, these findings have shifted the perception of nitazenes from ultrapotent MOR agonists toward a privileged opioid ligand chemotype and advanced structure–activity understanding of this opioid family, opened up a wide landscape for further investigation aiming to validate the hypothesized novel binding mode, and underscored the potential of the nitazene scaffold as a foundation for designing next-generation MOR modulators.

## Experimental Details

### Chemistry

All commercial reagents and chemicals were procured from Sigma-Aldrich, Bepharm Scientific Inc., and Combi-Blocks and used without any further purification. Regular Thin layer chromatography was performed by using silica gel GHIF plates, 250 μm, 2.5 × 10 cm (Analtech Uniplate). Proton (^1^H, 400 MHz) and Carbon (^13^C, 100 MHz) Nuclear Magnetic Resonance (NMR) spectra were acquired at ambient temperatures on a Bruker Ultrashield 400 Plus spectrometer. Chemical shift values (δ) are described in parts per million (ppm) and coupling constant values (J) are specified in hertz (Hz). Abbreviations used in the NMR interpretation are broad singlet (bs), singlet (s), doublet (d), triplet (t), quartet (q), doublet of doublet (dd), and multiplet (m). Mass spectra were recorded with an Applied BioSystems 3200 Q trap with a turbo V source for TurbolonSpray. The reverse-phase High-Performance Liquid Chromatography (HPLC) was performed on the system Waters Arc HPLC. The conditions for the HPLC are column: XBridge C_18_ 3.5 μm (4.6 × 50 mm); sample concentration: 0.125 mg/mL; injection solvent: acetonitrile; injection volume: 5 μL; isocratic mobile phase: 20% mobile phase A – 0.1% trifluoroacetic acid in water, 80% mobile phase B – acetonitrile; flow rate: 0.2 mL/min; single wavelength: 210 nm; run time: 10 min. The purity of all synthesized final compounds was found to be >95% (Table S2).

#### Procedure for the Synthesis of *N* ^1^-(2,4-Dinitrophenyl)-*N* ^2^,*N* ^2^-diethylethane-1,2-diamine (**1**)

Potassium carbonate (K_2_CO_3_, 2 equiv) was added in anhydrous ethanol in a predried round-bottom flask purged with nitrogen. To this mixture, N,N’-diethylethylenediamine (1.2 equiv) was added dropwise under stirring followed by addition of 1-bromo-2,4-dinitrobenzene (1 equiv). The resulting mixture was stirred at reflux in an oil bath for 48 h and then cooled to room temperature. This solution was evaporated under reduced pressure and partitioned between basic water (pH 10 to 12) and dichloromethane (DCM). The aqueous layer was extracted multiple times with DCM. Organic layer was combined together, washed with distilled water and brine, dried over sodium sulfate, and concentrated to obtain viscous liquid which was purified by column chromatography (dichloromethane:0.1% NH_4_OH in methanol). ^1^H NMR (400 MHz, δ ppm, CDCl_3_) 9.15 (d, *J =* 2.66 Hz, 1H), 9.12 (bs, 1H), 8.26 (dd, *J =* 9.48 Hz, 2.63 Hz, 1H), 6.87 (d, *J =* 9.51 Hz, 1H), 3.39 (q, *J =* 5.67 Hz, 2H), 2.80 (t, *J =* 6.00 Hz, 2H), 2.60 (q, *J =* 7.10 Hz, 2H), 1.07 (t, *J =* 7.12 Hz, 6H). ^13^C NMR (100 MHz, DMSO-*d*_6_) 148.4, 135.2, 130.4, 129.9, 124.0, 116.2, 79.6, 50.4, 46.4, 41.0, 39.8, 12.3. HRMS (*m*/*z*): calc. 282.1328; obs. 283.1407 (M+H)^+^.

#### Procedure for the Synthesis of *N* ^1^-(2-(Diethylamino)­ethyl)-4-nitrobenzene-1,2-diamine (**2**)

In a two neck round-bottom flask fitted with a dropping funnel and a condenser was added anhydrous ethanol and intermediate 1 (1 equiv) and heated to 65 °C in an oil bath. In the dropping funnel, distilled water (12 mL), anhydrous ethanol (20 mL), and aqueous (NH_4_)_2_S (40–48 wt %, 6 equiv) were charged and added dropwise to the hot reaction mixture over a period of 30 min. The reaction mixture was heated to 65–70 °C for 2 h then cooled to room temperature with continued stirring overnight. The reaction mixture was acidified by 1 M HCl solution to adjust the pH to 0 to 1 and the filtrate was concentrated under reduced pressure. The aqueous solution was basified with ammonium hydroxide solution to maintain the pH to 10 and this was further extracted with DCM. The organic was collected, washed with distilled water, brine, dried over sodium sulfate, and concentrated under reduced pressure to afford dark red liquid which was purified by column chromatography with dichloromethane:0.1% NH_4_OH in methanol mobile phase to get orange liquid. ^1^H NMR (400 MHz, δ ppm, DMSO-*d*_6_) 7.53 (dd, *J =* 8.84 Hz, 2.65 Hz, 1H), 7.42 (d, *J =* 2.67 Hz, 1H), 6.50 (d, *J =* 8.91 Hz, 1H), 5.81 (t, *J =* 5.19 Hz, 1H), 5.07 (bs, 2H), 3.25 (q, *J =* 6.8 Hz, 2H), 2.63 (t, *J =* 13.75 Hz, 2H), 2.55–2.50 (m, 4H), 0.97 (t, *J =* 7.10 Hz, 6H). ^13^C NMR (100 MHz, DMSO-*d*_6_). 143.3, 137.0, 134.9, 116.5, 108.0, 107.4, 59.9, 51.5, 47.0, 41.6, 12.2, 8.7. HRMS (*m*/*z*): calc. 252.1586; obs. 253.1656 (M+H)^+^.

#### Procedure for the Synthesis of 2-(2-(4-Ethoxybenzyl)-5-nitro-1*H*-benzo­[*d*]­imidazol-1-yl)-*N*,*N*-diethylethanamine Hydrochloride (Etonitazene Hydrochloride)

Pale yellow powder. Yield – 82%. In a predried round-bottom flask fitted with nitrogen and molecular sieves was added 1-ethyl-3-(3-(dimethylamino)­propyl)­carbodiimide (EDCI, 1.5 equiv), hydroxybenzotriazole (HOBt, 1.5 equiv), 4-ethoxyphenylacetic acid (1.2 equiv), and anhydrous triethanolamine (4 equiv) in anhydrous dimethylformamide (DMF, 3 mL). This mixture was stirred for 2 h under an ice bath, followed by the addition of intermediate 2 predissolved in anhydrous DMF (2 mL), and stirring was continued at room temperature for 7 days. After the complete utilization of the starting material, the reaction mixture was evaporated to dryness under reduced pressure to obtain crude which was partitioned between basic water (pH 10 to 12) and DCM. The aqueous layer was extracted multiple times with DCM. The organic layer was combined, washed with distilled water and brine, dried over sodium sulfate, and concentrated to obtain a viscous liquid, which was purified by column chromatography (dichloromethane:0.1% NH_4_OH in methanol) to afford free base of compound 3. This intermediate (1 equiv) was dissolved in a minimum amount of methanol (0.1 mL) in a dry round-bottom flask and stirred under an ice bath. To this mixture was dropwise added HCl in methanol solution (4 equiv), and stirring was continued for 20 to 30 min. To this solution, diethyl ether was added and stirring was continued for 18 h to precipitate the solid, which was then filtered under vacuum and washed with cold diethyl ether to obtain etonitazene hydrochloride. ^1^H NMR (400 MHz, δ ppm, DMSO-*d*_6_) 10.90 (bs, 1H), 8.50 (d, *J =* 2.15 Hz, 1H), 8.20 (dd, *J =* 8.93 Hz, 2.17 Hz, 1H), 7.93 (d, *J =* 8.83 Hz, 1H), 7.28 (d, *J =* 8.33 Hz, 2H), 6.89 (d, *J =* 8.67 Hz, 2H), 4.78 (t, *J =* 6.96, 2H), 4.37 (s, 2H), 3.99 (q, *J =* 6.96 Hz, 2H), 3.24–3.11 (m, 6H), 1.30 (t, *J =* 6.97 Hz, 3H), 1.20 (t, *J =* 7.23 Hz, 6H). ^13^C NMR (100 MHz, DMSO-*d*_6_) 158.7, 158.0, 143.5, 141.6, 139.7, 130.5, 127.8, 118.4, 115.1, 115.1, 111.4, 63.4, 49.2, 46.6, 38.8, 32.4, 15.1, 8.7. HRMS (*m*/*z*): calc. 396.2161; obs. 397.2229 (M+H)^+^. HPLC data: purity – 98.35%, Retention time – 2.5 min.

#### General Procedure for the Synthesis of Intermediates **2a**–**2d**

Potassium carbonate (K_2_CO_3_, 2 equiv) was added in anhydrous ethanol in a predried round-bottom flask purged with nitrogen. To this mixture, the corresponding amines (1a-1d, 1.2 equiv) were added dropwise under stirring followed by 1-fluoronitrobenzene (1 equiv). The resulting mixture was stirred at reflux in an oil bath for 24 to 48 h and then cooled to room temperature. This solution was evaporated under reduced pressure and partitioned between basic water (pH 10 to 12) and DCM. The aqueous layer was extracted multiple times with DCM. Organic layer was combined together, washed with distilled water and brine, dried over sodium sulfate, and concentrated to obtain viscous liquid which was purified by column chromatography (hexane: ethyl acetate) to get the corresponding intermediates (2a-2d).

#### General Procedure for the Synthesis of Intermediates **3a**–**3d**

Step 1 intermediates 2a-2d (1 equiv) were dissolved in anhydrous ethanol (30 mL) in a predried flask fitted with nitrogen. Palladium on activated carbon (10 wt %, 0.1 equiv) was charged into the corresponding solution, and conc. HCl was added dropwise to maintain the pH at 1–2. The nitrogen atmosphere from this flask was evacuated by a vacuum pump and quickly replaced with hydrogen from the hydrogen cylinder, and this process was repeated twice more. This mixture was stirred under a hydrogen atmosphere (50–55 psi) at room temperature for 18 to 20 h. After complete consumption of the starting material, the reaction mixture was filtered through a pad of Celite to remove the insolubles and the filtrate was evaporated to dryness under reduced pressure to obtain the corresponding title intermediates used without any further purification.

#### General Procedure for the Synthesis of Intermediates **5a**–**8h**

In a predried round-bottom flask fitted with nitrogen and molecular sieves was added EDCI (1.5 equiv), HOBt (1.5 equiv), 4-substituted phenylacetic acid (4a-4h, 1.2 equiv), and anhydrous triethanolamine (4 equiv) in anhydrous DMF (3 mL). This mixture was stirred for 1 to 2 h under an ice bath, followed by the addition of the corresponding step 2 intermediate (3a-3d) predissolved in anhydrous DMF (2 mL), and stirring was continued at room temperature for 1 to 7 days. After the complete utilization of the starting material, the reaction mixture was evaporated to dryness under reduced pressure to obtain crude which was partitioned between basic water (pH 10 to 12) and DCM. The aqueous layer was extracted multiple times with DCM. The organic layer was combined, washed with distilled water and brine, dried over sodium sulfate, and concentrated to obtain a viscous liquid, which was purified by column chromatography (dichloromethane:0.1% NH_4_OH in methanol) to afford the corresponding title intermediates.

#### General Procedure for the Synthesis of Final Compounds **1**–**32**

Intermediates of step 3 (1 equiv) and phosphorus pentachloride (1.5 equiv) were dissolved in anhydrous chloroform (10 mL) in a round-bottom flask under nitrogen fitted with a condenser and magnetic stir bar. This solution was heated in an oil bath to reflux for 3 to 24 h. Upon completion, the reaction mixture was cooled and quenched with basic water (pH 9 to 10) under an ice bath. The aqueous layer was extracted multiple times with DCM. The organic layer was combined, washed with distilled water and brine, dried over sodium sulfate, and concentrated to afford liquid, which was purified by column chromatography (dichloromethane:0.1% NH_4_OH in methanol) to afford the respective free base. The free base (1 equiv) was dissolved in a minimum amount of methanol (0.1–0.2 mL) in a separate dry round-bottom flask and stirred under an ice bath. To this mixture HCl in methanol solution (4 equiv) was added dropwise and stirring was continued for 20 to 30 min. To this solution, diethyl ether was added, and stirring was continued for 3 to 18 h to precipitate the solid, which was then filtered under vacuum and washed with cold diethyl ether to afford the final compounds as hydrochloride salts.

#### 2-(2-(4-Fluorobenzyl)-1*H*-benzo­[*d*]­imidazol-1-yl)-*N*,*N*-dimethylethanamine Hydrochloride (**1**)

White powder. Yield -88%. ^1^H NMR (400 MHz, δ ppm, DMSO-*d*_6_) 11.21 (bs, 1H), 7.96 (s, 1H), 7.72–7.70 (m, 1H), 7.53–7.45 (m, 4H), 7.24 (t, *J =* 8.80 Hz, 2H), 4.84–4.83 (m, 2H), 4.60 (s, 2H), 3.41 (m, 2H), 2.86 (d, *J =* 4.35 Hz, 6H). ^13^C NMR (100 MHz, DMSO-*d*_6_) 163.3, 153.8, 132.0, 131.9, 125.3, 116.3, 116.1, 112.7, 53.5, 42.8, 39.6, 39.4, 31.1. HRMS (*m*/*z*): calc. 297.1641; obs. 298.1723 (M+H)^+^. HPLC data: purity – 100%, Retention time – 2.29 min.

#### *N*,*N*-Dimethyl-2-(2-(4-methylbenzyl)-1*H*-benzo­[*d*]­imidazol-1-yl)­ethanamine Hydrochloride (**2**)

White powder. Yield -79%. ^1^H NMR (400 MHz, δ ppm, DMSO-*d*_6_) 11.31 (s, 1H), 7.99 (s, 1H), 7.73 (d, *J =* 7.56 Hz, 1H), 7.53–7.48 (m, 2H), 7.36–7.34 (m, 2H), 7.22 (d, *J =* 7.86 Hz, 2H), 4.86–4.84 (m, 2H), 4.59 (s, 2H), 3.41 (m, 2H), 2.84 (d, *J =* 4.03 Hz, 6H), 2.31 (s, 3H). ^13^C NMR (100 MHz, DMSO-*d*_6_) 154.0, 137.4, 132.7, 130.9, 130.1, 129.7, 125.4, 115.9, 112.8, 53.4, 42.8, 31.4, 21.1. HRMS (*m*/*z*): calc. 293.1891; obs. 294.1954 (M+H)^+^. HPLC data: purity – 100%, Retention time – 2.28 min.

#### 2-(2-(4-Isopropylbenzyl)-1*H*-benzo­[*d*]­imidazol-1-yl)-*N*,*N*-dimethylethanamine Hydrochloride (**3**)

White powder. Yield -83%. ^1^H NMR (400 MHz, δ ppm, DMSO-*d*_6_) 11.04 (bs, 1H), 7.93 (s, 1H), 7.73–7.71 (m, 1H), 7.46–7.44 (m, 2H), 7.36–7.34 (m, 2H), 7.27–7.25 (m, 2H), 4.81 (s, 2H), 4.54 (s, 2H), 3.37 (m, 2H), 2.92–2.87 (m, 1H), 2.84 (d, *J =* 4.31 Hz, 6H), 1.19 (d, *J =* 6.91 Hz, 6H). ^13^C NMR (100 MHz, DMSO-*d*_6_) 153.9, 148.3, 129.7, 127.4, 53.5, 42.8, 33.5, 31.5, 24.2. HRMS (*m*/*z*): calc. 321.2204; obs. 322.2268 (M+H)^+^. HPLC data: purity – 99.56%, Retention time – 2.31 min.

#### *N*,*N*-Dimethyl-2-(2-(4-(trifluoromethyl)­benzyl)-1*H*-benzo­[*d*]­imidazol-1-yl)­ethanamine Hydrochloride (**4**)

White powder. Yield -70%. ^1^H NMR (400 MHz, δ ppm, DMSO-*d*_6_) 11.55 (bs, 1H), 8.04 (d, *J =* 7.91 Hz, 1H), 7.79–7.72 (m, 4H), 7.55–7.46 (m, 2H), 4.91 (t, *J =* 15.65 Hz, 2H), 4.78 (s, 2H), 3.5 (m, 2H), 2.86 (d, *J =* 4.07 Hz, 6H). ^13^C NMR (100 MHz, DMSO-*d*_6_) 153.1, 133.0, 130.8, 128.8, 126.2, 126.2, 125.4, 125.2, 112.6, 53.6, 42.7, 31.7. HRMS (*m*/*z*): calc. 347.1609; obs. 348.1694 (M+H)^+^. HPLC data: purity – 99.62%, Retention time – 2.34 min.

#### 2-(2-(4-Chlorobenzyl)-1*H*-benzo­[*d*]­imidazol-1-yl)-*N*,*N*-dimethylethanamine Hydrochloride (**5**)

Off white powder. Yield -84%. ^1^H NMR (400 MHz, δ ppm, DMSO-*d*_6_) 11.60 (bs, 1H), 8.06 (d, *J =* 7.90 Hz, 1H), 7.74–7.73 (m, 1H), 7.59–7.47 (m, 5H), 4.91 (t, *J =* 15.60 Hz, 2H), 4.69 (s, 2H), 3.41–3.35 (m, 2H), 2.86 (d, *J =* 4.00 Hz, 6H). ^13^C NMR (100 MHz, DMSO-*d*_6_) 153.5, 132.9, 132.8, 131.9, 129.4, 125.7, 125.4, 115.9, 112.8, 53.5, 42.7, 31.2. HRMS (*m*/*z*): calc. 313.1345; obs. 314.1414 (M+H)^+^. HPLC data: purity – 99.54%, Retention time – 2.31 min.

#### 4-((1-(2-(Dimethylamino)­ethyl)-1*H*-benzo­[*d*]­imidazol-2-yl)­methyl)­benzonitrile Hydrochloride (**6**)

Pale yellow powder. Yield -85%. ^1^H NMR (400 MHz, δ ppm, DMSO-*d*_6_) 11.87 (bs, 1H), 8.15–8.13 (m, 1H), 7.91–7.89 (m, 2H), 7.79–7.77 (m, 3H), 7.60–7.52 (m, 2H), 4.98 (t, *J =* 7.70 Hz, 2H), 4.88 (s, 2H), 3.53 (s, 2H), 2.88 (d, *J =* 4.20 Hz, 6H). ^13^C NMR (100 MHz, DMSO-*d*_6_) 152.7, 139.5, 133.3, 132.7, 132.5, 131.1, 126.1, 125.8, 119.1, 115.5, 113.1, 111.1, 65.3, 53.4, 42.7, 31.6, 15.6. HRMS (*m*/*z*): calc. 304.1687; obs. 305.1752 (M+H)^+^. HPLC data: purity – 99.52%, Retention time – 2.32 min.

#### 2-(2-(4-Ethylbenzyl)-1*H*-benzo­[*d*]­imidazol-1-yl)-*N*,*N*-dimethylethanamine Hydrochloride (**7)**

Pale yellow powder. Yield -65%. ^1^H NMR (400 MHz, δ ppm, DMSO-*d*_6_) 11.48 (bs, 1H), 8.05–8.03 (m, 1H), 7.76–7.74 (m, 1H), 7.56–7.49 (m, 2H), 7.40–7.38 (m, 2H), 7.26–7.22 (m, 2H), 4.90 (t, *J =* 15.66 Hz, 2H) 4.62 (s, 2H), 2.84 (d, *J =* 3.34 Hz, 6H), 2.61 (q, *J =* 7.56 Hz, 2H), 1.17 (t, *J =* 7.58 Hz, 3H). ^13^C NMR (100 MHz, DMSO-*d*_6_) 154.0, 129.7, 128.9, 53.5, 42.8, 31.5, 28.2, 15.9. HRMS (*m*/*z*): calc. 307.2048; obs. 308.2130 (M+H)^+^. HPLC data: purity – 99.12%, Retention time – 2.30 min.

#### 2-(2-(4-Ethoxybenzyl)-1*H*-benzo­[d]­imidazol-1-yl)-*N*,*N*-dimethylethan-1-amine Hydrochloride (**8**)

Off white powder. Yield -26%. ^1^H NMR (400 MHz, δ ppm, DMSO-*d*_6_) 11.79 (bs, 1H), 8.12–8.10 (m, 1H), 7.78–7.76 (m, 1H), 7.60–7.53 (m, 2H), (7.46–7.44 (m, 2H), 6.97–6.95 (m, 2H), 4.96 (t, *J =* 7.97 Hz, 2H), 4.64 (s, 2H), 4.05–4.00 (m, 2H), 3.45 (s, 2H), 2.85 (d, *J =* 1.83 Hz, 6H), 1.32 (t, *J =* 7.34 Hz, 3H). ^13^C NMR (100 MHz, DMSO-*d*_6_) 158.6, 154.2, 132.3, 131.1, 126.2, 125.8, 125.1, 115.4, 115.3, 113.1, 63.5, 53.3, 42.7, 30.7, 15.1. HRMS (*m*/*z*): calc. 323.1997; obs. 324.2069 (M+H)^+^. HPLC data: purity – 98.83%, Retention time – 2.28 min.

#### *N*,*N*-Diethyl-2-(2-(4-fluorobenzyl)-1*H*-benzo­[*d*]­imidazol-1-yl)­ethan-1-amine Hydrochloride (**9**)

Yellowish solid. Yield -78%. ^1^H NMR (400 MHz, δ ppm, DMSO-*d*_6_) 10.93 (bs, 1H), 7.91 (s, 1H), 7.69 (s, 1H), 7.48 (dd, *J* = 7.5, 4.6 Hz, 5H), 7.23 (s, 2H), 4.86 (s, 2H), 4.56 (s, 4H), 1.23 (s, 6H). ^13^C NMR (100 MHz, δ ppm, DMSO-*d*_6_) 162.7, 160.3, 153.9, 142.7, 135.6, 133.7, 133.7, 131.0, 131.0, 122.1, 121.7, 119.0, 115.7, 115.5, 110.5, 52.2, 47.2, 42.8, 32.7, 12.2. HRMS (*m*/*z*): calc. 325.1954; obs. 326.2026 (M+H)^+^. HPLC data: purity – 96.66%, Retention time – 2.31 min.

#### 2-(2-(4-Chlorobenzyl)-1*H*-benzo­[*d*]­imidazol-1-yl)-*N*,*N*-diethylethan-1-amine Hydrochloride (**10**)

Yellowish solid. Yield -74%. ^1^H NMR (400 MHz, δ ppm, DMSO-*d*_6_) 10.65 (bs, 1H), 7.85 (s, 1H), 7.68 (d, J = 7.8 Hz, 2H), 7.45 (d, J = 2.3 Hz, 4H), 7.42–7.37 (m, 2H), 4.80 (s, 2H), 4.52 (s, 2H), 1.22 (t, J = 7.2 Hz, 6H). ^13^C NMR (100 MHz, δ ppm, DMSO-*d*_6_) 153.7, 142.7, 136.6, 135.6, 131.7, 131.1, 128.9, 122.1, 121.7, 119.0, 110.5, 52.2, 47.2, 42.8, 32.8, 12.2. HRMS (*m*/*z*): calc. 341.1658; obs. 342.1719 (M+H)^+^. HPLC data: purity – 97.58%, Retention time – 2.33 min.

#### *N*,*N*-Diethyl-2-(2-(4-(trifluoromethyl)­benzyl)-1*H*-benzo­[*d*]­imidazol-1-yl)­ethan-1-amine Hydrochloride (**11**)

Yellowish solid. Yield -100% ^1^H NMR (400 MHz, δ ppm, DMSO-*d*_6_) 11.68 (s, 1H), 8.07 (m, 1H), 7.89–7.62 (m, 5H), 7.59–7.46 (m, 2H), 5.01 (t, *J =* 8.1 Hz, 2H), 4.81 (s, 2H), 3.46–3.34 (m, 2H), 3.31–3.12 (m, 4H), 1.25 (t, *J =* 7.2 Hz, 6H). ^13^C NMR (100 MHz, δ ppm, DMSO-*d*_6_) 153.7, 142.7, 136.6, 135.6, 131.7, 131.1, 128.9, 122.1, 121.7, 119.0, 110.5, 52.2, 47.2, 42.8, 32.8, 12.2. HRMS (*m*/*z*): calc. 375.1922; obs. 376.2005 (M+H)^+^. HPLC data: purity – 98.87%, Retention time – 2.35 min.

#### 4-((1-(2-(Diethylamino)­ethyl)-1*H*-benzo­[*d*]­imidazol-2-yl)­methyl)­benzonitrile Hydrochloride (**12**)

Pinkish solid. Yield -80%. ^1^H NMR (400 MHz, δ ppm, DMSO-*d*_6_) 11.11 (s, 1H), 7.96–7.76 (m, 3H), 7.67 (dd, *J* = 15.3, 7.9 Hz, 3H), 7.42 (m, 2H), 4.88 (t, *J* = 8.1 Hz, 2H), 4.68 (s, 2H), 3.27–3.14 (m, 6H), 1.32–1.07 (m, 6H). ^13^C NMR (100 MHz, δ ppm, DMSO-*d*_6_) 171.6, 153.1, 143.6, 142.7, 135.5, 132.8, 132.4, 130.7, 130.5, 122.2, 121.8, 119.3, 119.1, 110.5, 109.9, 52.2, 47.3, 42.8, 42.5, 33.5, 12.1. HRMS (*m*/*z*): calc. 332.2000; obs. 333.2060 (M+H)^+^. HPLC data: purity – 96.47%, Retention time – 2.33 min.

#### *N*,*N*-Diethyl-2-(2-(4-methylbenzyl)-1*H*-benzo­[*d*]­imidazol-1-yl)­ethan-1-amine Hydrochloride (**13**)

Pinkish solid. Yield -86%. ^1^H NMR (400 MHz, δ ppm, DMSO-*d*_6_) 11.56 (bs, 1H), 8.09 (d, *J* = 6.6 Hz, 1H), 7.80–7.73 (m, 1H), 7.55 (m, 2H), 7.37 (dd, *J* = 7.9, 1.8 Hz, 2H), 7.23 (d, *J* = 7.9 Hz, 2H), 4.99 (t, *J* = 7.9 Hz, 2H), 4.64 (s, 2H), 3.33 (s, 2H), 3.22 (m, 4H), 2.31 (s, 3H), 1.23 (t, *J* = 7.2 Hz, 6H). ^13^C NMR (100 MHz, δ ppm, DMSO-*d*_6_) 170.1, 135.8, 133.6, 129.4, 129.2, 127.2, 126.8, 124.0, 116.2, 111.5, 51.6, 46.8, 42.6, 21.1. HRMS (*m*/*z*): calc. 321.2204; obs. 322.2286 (M+H)^+^. HPLC data: purity – 99.25%, Retention time – 2.30 min.

#### *N*,*N*-Diethyl-2-(2-(4-isopropylbenzyl)-1*H*-benzo­[*d*]­imidazol-1-yl)­ethan-1-amine Hydrochloride (**14**)

White solid. Yield -82%. ^1^H NMR (400 MHz, δ ppm, DMSO-*d*_6_) 11.83 (bs, 1H), 8.21–8.14 (m, 1H), 7.85–7.77 (m, 1H), 7.66–7.54 (m, 2H), 7.48–7.39 (m, 2H), 7.32–7.20 (m, 2H), 5.12–5.04 (m, 2H), 4.72 (s, 2H), 3.35 (dd, *J* = 7.8, 2.7 Hz, 2H), 3.29–3.18 (m, 4H), 2.96–2.82 (m, 1H), 1.23 (t, *J* = 7.2 Hz, 6H), 1.19 (d, *J* = 6.9 Hz, 6H). ^13^C NMR (100 MHz, δ ppm, DMSO-*d*_6_) 153.8, 148.5, 132.2, 130.9, 130.1, 129.7, 127.5, 126.6, 126.1, 115.1, 113.4, 49.0, 48.5, 46.6, 33.5, 31.0, 24.2, 8.7. HRMS (*m*/*z*): calc. 349.2517; obs. 350.2581 (M+H)^+^. HPLC data: purity – 96.46%, Retention time – 2.34 min.

#### *N*,*N*-Diethyl-2-(2-(4-ethylbenzyl)-1*H*-benzo­[*d*]­imidazol-1-yl)­ethan-1-amine Hydrochloride (**15**)

White solid. Yield -90%. ^1^H NMR (400 MHz, δ ppm, DMSO-*d*_6_) 11.38 (bs, 1H), 7.51 (m, 2H), 7.37 (d, *J* = 8.1 Hz, 2H), 7.24 (d, *J* = 8.0 Hz, 2H), 4.95 (t, *J* = 8.1 Hz, 2H), 4.60 (s, 2H), 3.27 (d, *J* = 5.5 Hz, 2H), 3.24–3.14 (m, 4H), 2.60 (q, *J* = 7.6 Hz, 2H), 1.26–1.19 (m, 6H), 1.16 (d, *J* = 7.6 Hz, 3H). ^13^C NMR (100 MHz, δ ppm, DMSO-*d*_6_) 153.5, 142.9, 142.7, 135.3, 133.7, 128.4, 128.3, 122.1, 121.8, 119.6, 109.2, 51.9, 47.6, 43.1, 34.2, 28.4, 15.5, 11.9. HRMS (*m*/*z*): calc. 335.2361; obs. 336.2446 (M+H)^+^. HPLC data: purity – 99.54%, Retention time – 2.33 min.

#### 2-(2-(4-Ethoxybenzyl)-1*H*-benzo­[d]­imidazol-1-yl)-*N*,*N*-diethylethan-1-amine Hydrochloride (**16**)

Off white powder. Yield -26%. ^1^H NMR (400 MHz, δ ppm, DMSO-*d*_6_) 11.84 (bs, 1H), 8.14–8.13 (m, 1H), 7.79–7.77 (m, 1H), 7.60–7.75 (m, 2H), 7.74–7.44 (m, 2H), 6.96–6.94 (m, 2H), 5.04 (t, *J =* 8.70 Hz, 2H), 4.64 (s, 2H), 4.04–3.99 (m, 2H), 3.35–3.22 (m, 2H), 3.21–3.12 (m, 2H), 1.31 (t, *J =* 7.05 Hz, 3H), 1.24 (t, *J*= 7.22 Hz, 6H). ^13^C NMR (100 MHz, DMSO-*d*_6_) 158.6, 154.1, 132.4, 131.0, 126., 125.8, 125.3, 115.4, 113.1, 63.5, 48.5, 46.6, 30.7, 15.0, 8.7. HRMS (*m*/*z*): calc. 351.2310; obs. 352.2387 (M+H)^+^. HPLC data: purity – 98.52%, Retention time – 2.28 min.

#### 2-(4-Chlorobenzyl)-1-(2-(pyrrolidin-1-yl)­ethyl)-1*H*-benzo­[*d*]­imidazole Hydrochloride (**17**)

Pale solid. Yield -60%. ^1^H NMR (400 MHz, δ ppm, DMSO-*d*_6_) 11.53 (bs, 1H), 8.00–7.99 (d, 1H), 7.72–7.70 (d, 1H), 7.52–7.44 (m, 6H), 4.84 (t, J= 7.71 Hz, 2H), 4.64 (s, 2H), 3.57–3.52 (m, 3H), 3.11–3.06 (m, 3H), 2.03–2.01 (m, 2H), 1.92–1.89 (m, 2H). ^13^C NMR (100 MHz, δ ppm, DMSO-*d*_6_) 153.5, 132.9, 131.8, 129.4, 125.3, 116.0, 112.8, 53.4, 51.0, 45.9, 39.4, 31.3, 23.3, 8.9. HRMS (*m*/*z*): calc. 339.1502; obs. 340.1577 (M+H)^+^. HPLC data: purity – 99.05%, Retention time – 2.31 min.

#### 1-(2-(Pyrrolidin-1-yl)­ethyl)-2-(4-(trifluoromethyl)­benzyl)-1*H*-benzo­[*d*]­imidazole Hydrochloride (**18**)

Pale solid. Yield -63%. ^1^H NMR (400 MHz, δ ppm, DMSO-*d*_6_) 11.78 (bs, 1H), 8.07–8.05 (d, 1H), 7.80–7.78 (d, 2H), 7.74–7.72 (d, 3H), 7.55–7.47 (m, 2H), 4.90 (t, *J*= 7.56 Hz, 2H), 4.80 (s, 2H), 3.60–3.41 (m, 3H), 3.12–3.08 (m, 3H), 2.05–2.01 (m, 2H), 1.93–1.90 (m, 2H). ^13^C NMR (100 MHz, δ ppm, DMSO-*d*_6_) 153.1, 130.9, 126.2, 126.2, 125.7, 125.4, 112.9, 53.4, 51.0, 39.4, 31.7, 23.3. HRMS (*m*/*z*): calc. 373.1765; obs. 374.1825 (M+H)^+^. HPLC data: purity – 98.04%, Retention time – 2.34 min.

#### 2-(4-Fluorobenzyl)-1-(2-(pyrrolidin-1-yl)­ethyl)-1*H*-benzo­[*d*]­imidazole Hydrochloride (**19**)

Pale solid. Yield -40%. ^1^H NMR (400 MHz, δ ppm, DMSO-*d*_6_) 11.85 (bs, 1H), 8.09–8.07 (d, 1H), 7.76–7.74 (m, 1H), 7.59–7.49 (m, 4H), 7.28–7.23 (m, 2H), 4.91 (t, *J*= 7.62 Hz, 2H), 4.69 (s, 2H), 3.60–3.47 (m, 3H), 3.12–3.08 (m, 3H), 2.05–2.00 (m, 2H), 1.93–1.90 (m, 2H). ^13^C NMR (100 MHz, δ ppm, DMSO-*d*_6_) 163.4, 161.0, 153.7, 132.6, 132.1, 132.0, 130.0, 126.0, 125.6, 116.4, 116.2, 115.6, 113.0, 53.4, 50.9, 31.0, 23.3. HRMS (*m*/*z*): calc. 323.1797; obs. 324.1876 (M+H)^+^. HPLC data: purity – 99.57%, Retention time – 2.31 min.

#### 2-(4-Ethylbenzyl)-1-(2-(pyrrolidin-1-yl)­ethyl)-1*H*-benzo­[*d*]­imidazole Hydrochloride (**20**)

Pale solid. Yield -49%. ^1^H NMR (400 MHz, δ ppm, DMSO-*d*_6_) 11.81 (bs, 1H), 8.08–8.06 (d, 1H), 7.77–7.75 (d, 1H), 7.56–7.52 (m, 2H), 7.42–7.40 (m, 2H), 7.26–7.24 (m, 2H), 4.90 (t, *J*= 7.47 Hz, 2H), 4.65 (s, 2H), 3.57–3.49 (m, 3H), 3.09–3.04 (m, 3H), 2.62 (q, *J*= 7.45 Hz, 2H), 2.02–2.00 (m, 2H), 1.92–1.91 (m, 2H), 1.20–1.16 (t, *J*= 7.32 Hz, 3H). ^13^C NMR (100 MHz, δ ppm, DMSO-*d*_6_) 154.1, 142.8, 142.5, 135.6, 134.7, 129.1, 128.4, 122.2, 121.8, 119.1, 110.5, 55.4, 54.7, 54.2, 49.1, 43.0, 33.2, 28.3, 23.6, 16.6, 16.2. HRMS (*m*/*z*): calc. 333.2204; obs. 334.2292 (M+H)^+^. HPLC data: purity – 99.28%, Retention time – 2.32 min.

#### 2-(4-Methylbenzyl)-1-(2-(pyrrolidin-1-yl)­ethyl)-1*H*-benzo­[*d*]­imidazole Hydrochloride (**21**)

Pale solid. Yield -17%. ^1^H NMR (400 MHz, δ ppm, DMSO-*d*_6_) 11.78 (bs, 1H), 8.07–8.05 (d, 1H), 7.76–7.74 (d, 1H), 7.57–7.51 (m, 2H), 7.39–7.37 (d, 2H), 7.23–7.21 (m, 2H), 4.88 (t, *J*= 7.68 Hz, 2H), 4.64 (s, 2H), 3.57–3.50 (m, 3H), 3.09–3.05 (m, 3H), 2.31 (s, 3H), 2.02–2.00 (m, 2H), 1.92–1.89 (m, 2H). ^13^C NMR (100 MHz, δ ppm, DMSO-*d*_6_) 154.1, 142.8, 136.1, 135.6, 134.4, 129.5, 129.0, 122.2, 121.7, 119.1, 110.5, 55.4, 54.8, 54.3, 49.1, 43.0, 33.2, 23.6, 21.1, 16.6. HRMS (*m*/*z*): calc. 319.2048; obs. 320.2107 (M+H)^+^. HPLC data: purity – 99.08%, Retention time – 2.29 min.

#### 2-(4-Isopropylbenzyl)-1-(2-(pyrrolidin-1-yl)­ethyl)-1*H*-benzo­[*d*]­imidazole Hydrochloride (**22**)

Pale solid. Yield -66%. ^1^H NMR (400 MHz, δ ppm, DMSO-*d*_6_) 11.88 (bs, 1H), 8.09–8.08 (d, 1H), 7.78–7.77 (d, 1H), 7.58–7.51 (m, 2H), 7.44–7.42 (d, 2H), 7.29–7.27 (d, 2H), 4.92 (t, *J*= 6.85 Hz, 2H), 4.66 (s, 2H), 3.57–3.52 (m, 4H), 3.08–3.06 (m, 2H), 2.93–2.86 (m, 1H), 2.01–1.99 (m, 2H), 1.95–1.89 (m, 2H), 1.20–1.19 (d, 6H). ^13^C NMR (100 MHz, δ ppm, DMSO-*d*_6_) 153.8, 148.4, 132.3, 131.1, 129.7, 127.4, 126.2, 125.8, 115.4, 113.2, 53.3, 50.8, 49.0, 33.5, 31.2, 24.2, 23.3. HRMS (*m*/*z*): calc. 347.2361; obs. 348.2451 (M+H)^+^. HPLC data: purity – 99.14%, Retention time – 2.33 min.

#### 4-((1-(2-(Pyrrolidin-1-yl)­ethyl)-1*H*-benzo­[*d*]­imidazol-2-yl)­methyl)­benzonitrile Hydrochloride (**23**)

Pale solid. Yield -33%. ^1^H NMR (400 MHz, δ ppm, DMSO-*d*_6_) 11.88 (bs, 1H), 8.09–8.07 (d, 1H), 7.91–7.89 (d, 2H), 7.75–7.72 (m, 3H), 7.56–7.48 (m, 2H), 4.91 (t, *J*= 8.11 Hz, 2H), 4.82 (s, 2H), 3.59–3.41 (m, 4H), 3.12–3.08 (m, 2H), 2.03–2.00 (m, 2H), 1.92–1.89 (m, 2H). ^13^C NMR (100 MHz, δ ppm, DMSO-*d*_6_) 152.8, 133.3, 131.1, 125.5, 119.1, 115.9, 113.0, 111.1, 55.3, 53.3, 50.9, 39.3, 31.8, 23.3. HRMS (*m*/*z*): calc. 330.1844; obs. 331.1927 (M+H)^+^. HPLC data: purity – 99.74%, Retention time – 2.33 min.

#### 2-(4-Ethoxybenzyl)-1-(2-(pyrrolidin-1-yl)­ethyl)-1*H*-benzo­[d]­imidazole Hydrochloride (**24**)

Off white powder. Yield -33%. ^1^H NMR (400 MHz, δ ppm, DMSO-*d*_6_) 11.87 (bs, 1H), 8.09–8.07 (m, 1H), 7.76–7.74 (m, 1H), 7.58–7.50 (m, 2H), 7.44–7.42 (m, 2H), 6.96–6.94 (m, 2H), 4.90 (t, *J =* 6.40 Hz, 2H), 4.82 (s, 2H), 4.05–3.99 (m, 2H), 3.56–3.53 (m, 4H), 3.12–3.06 (m, 2H), 2.02–1.92 (m, 2H), 1.91–1.89 (m, 2H), 1.32 (t, *J =* 7.92 Hz, 3H). ^13^C NMR (100 MHz, DMSO-*d*_6_) 158.6, 154.2, 132.4, 131.1, 126.2, 125.8, 125.1, 115.4, 113.1, 63.5, 53.5, 50.8. HRMS (*m*/*z*): calc. 349.2154; obs. 350.2244 (M+H)^+^. HPLC data: purity – 98.37%, Retention time – 2.28 min.

#### 2-(4-Chlorobenzyl)-1-(2-(piperidin-1-yl)­ethyl)-1*H*-benzo­[*d*]­imidazole Hydrochloride (**25**)

White powder. Yield – 69%. ^1^H NMR (400 MHz, δ ppm, DMSO-*d*_6_) 11.57 (bs, 1H), 8.10 (d, *J* = 7.9 Hz, 1H), 7.74 (dd, *J* = 7.2, 1.5 Hz, 1H), 7.59–7.45 (m, 6H), 5.06–4.94 (m, 2H), 4.70 (s, 2H), 2.97 (d, *J* = 7.9 Hz, 3H), 1.85 (m, 4H), 1.75 (d, *J* = 13.6 Hz, 1H). ^13^C NMR (100 MHz, δ ppm, DMSO-*d*_6_) 153.7, 142.7, 136.6, 135.6, 131.7, 131.2, 128.8, 122.2, 121.7, 119.0, 110.6, 57.9, 54.8, 41.6, 32.7, 26.0, 24.3. HRMS (*m*/*z*): calc. 353.1658; obs. 354.1720 (M+H)^+^. HPLC data: purity – 99.01%, Retention time – 2.31 min.

#### 1-(2-(Piperidin-1-yl)­ethyl)-2-(4-(trifluoromethyl)­benzyl)-1*H*-benzo­[*d*]­imidazole Hydrochloride (**26**)

White powder. Yield – 63%. ^1^H NMR (400 MHz, δ ppm, DMSO-*d*_6_) 11.63 (bs, 1H), 8.12 (d, *J* = 7.9 Hz, 1H), 7.77 (q, *J* = 8.1 Hz, 5H), 7.62–7.46 (m, 2H), 5.10–4.97 (m, 2H), 4.83 (s, 2H), 3.46 (s, 4H), 2.97 (d, *J* = 10.8 Hz, 2H), 1.93–1.80 (m, 4H), 1.75 (d, *J* = 13.3 Hz, 1H), 1.40 (q, *J* = 12.6 Hz, 1H). ^13^C NMR (100 MHz, δ ppm, DMSO-*d*_6_) 153.4, 142.7, 135.6, 130.2, 125.8, 125.7, 122.2, 121.8, 119.0, 110.6, 57.9, 54.8, 41.7, 33.2, 26.0, 24.3. HRMS (*m*/*z*): calc. 387.1922; obs. 388.1978 (M+H)^+^. HPLC data: purity – 98.32%, Retention time – 2.34 min.

#### 2-(4-Fluorobenzyl)-1-(2-(piperidin-1-yl)­ethyl)-1*H*-benzo­[*d*]­imidazole Hydrochloride (**27**)

White powder. Yield – 66%. ^1^H NMR (400 MHz, δ ppm, DMSO-*d*_6_) 11.62 (bs, 1H), 8.14 (d, *J* = 7.9 Hz, 1H), 7.77 (dd, *J* = 7.2, 1.6 Hz, 1H), 7.57 (m, 4H), 7.31–7.22 (m, 2H), 5.03 (t, *J* = 8.1 Hz, 2H), 4.72 (s, 2H), 2.97 (m, 3H), 1.90–1.80 (m, 4H), 1.75 (d, *J* = 13.7 Hz, 1H), 1.47–1.34 (m, 1H). ^13^C NMR (100 MHz, δ ppm, DMSO-*d*_6_) 153.9, 142.7, 135.6, 133.7, 133.6, 131.1, 131.1, 122.1, 121.7, 119.0, 115.7, 115.5, 110.5, 57.9, 54.8, 41.6, 32.6, 26.0, 24.3. HRMS (*m*/*z*): calc. 337.1954; obs. 338.2022 (M+H)^+^. HPLC data: purity – 99.01%, Retention time – 2.31 min.

#### 2-(4-Ethylbenzyl)-1-(2-(piperidin-1-yl)­ethyl)-1*H*-benzo­[*d*]­imidazole Hydrochloride (**28**)

White powder. Yield -37%. ^1^H NMR (400 MHz, δ ppm, DMSO-*d*_6_) 11.56 (bs, 1H), 8.09 (d, *J* = 7.8 Hz, 1H), 7.79–7.73 (m, 1H), 7.59–7.49 (m, 2H), 7.41 (d, *J* = 7.9 Hz, 2H), 7.25 (d, *J* = 7.8 Hz, 2H), 4.99 (t, *J* = 8.1 Hz, 2H), 4.64 (s, 2H), 3.49 (s, 2H), 2.90 (dd, *J* = 13.1, 8.4 Hz, 3H), 2.61 (q, *J* = 7.6 Hz, 3H), 1.84 (tt, *J* = 9.7, 3.8 Hz, 4H), 1.75 (d, *J* = 14.5 Hz, 1H), 1.40 (s, 1H), 1.18 (t, *J* = 7.6 Hz, 3H). ^13^C NMR (100 MHz, δ ppm, DMSO-*d*_6_) 154.2, 142.7, 142.5, 134.6, 129.1, 128.3, 122.1, 121.7, 119.0, 110.5, 54.7, 33.2, 31.1, 28.2, 16.1. HRMS (*m*/*z*): calc. 347.2361; obs. 348.2439 (M+H)^+^. HPLC data: purity – 99.27%, Retention time – 2.33 min.

#### 2-(4-Ethoxybenzyl)-1-(2-(piperidin-1-yl)­ethyl)-1*H*-benzo­[d]­imidazole Hydrochloride (**29**)

Off white powder. Yield -63%. ^1^H NMR (400 MHz, δ ppm, DMSO-*d*_6_) 11.85 (bs, 1H), 8.17–8.15 (m, 1H), 7.79–7.77 (m, 1H), 7.60–7.53 (m, 2H), 7.49–7.47 (m, 2H), 6.97–6.94 (m, 2H), 5.05 (t, *J =* 7.43 Hz, 2H), 4.66 (s, 2H), 4.04–3.99 (m, 2H), 3.53–3.50 (m, 2H), 3.40–3.35 (m, 2H), 2.99–2.92 (m, 2H), 1.93–1.73 (m, 5H), 1.47–1.38 (m, 1H), 1.36–1.30 (m, 3H). ^13^C NMR (100 MHz, DMSO-*d*_6_) 158.6, 154.1, 132.2, 131.8, 131.1, 126.3, 125.9, 125.1, 115.4, 115.2, 113.2, 65.3, 63.5, 52.7, 52.3, 30.6, 22.7, 21.7, 15.6, 15.0. HRMS (*m*/*z*): calc. 363.2310; obs. 364.2383 (M+H)^+^. HPLC data: purity – 99.46%, Retention time – 2.29 min.

#### 2-(4-Isopropylbenzyl)-1-(2-(piperidin-1-yl)­ethyl)-1*H*-benzo­[*d*]­imidazole Hydrochloride (**30**)

White powder. Yield – 24%. ^1^H NMR (400 MHz, δ ppm, DMSO-*d*_6_) 11.84 (bs, 1H), 8.20–8.15 (m, 1H), 7.84–7.77 (m, 1H), 7.64–7.54 (m, 2H), 7.50–7.44 (m, 2H), 7.32–7.26 (m, 2H), 5.06 (t, *J* = 8.2 Hz, 2H), 4.71 (s, 2H), 3.41–3.30 (m, 4H), 2.92 (m, 4H), 1.92–1.79 (m, 4H), 1.47–1.34 (m, 1H), 1.20 (d, *J* = 6.9 Hz, 6H). ^13^C NMR (100 MHz, δ ppm, DMSO-*d*_6_) 153.7, 148.5, 132.2, 131.0, 129.7, 127.5, 126.4, 126.0, 115.2, 113.3, 52.7, 52.3, 33.5, 31.0, 24.2, 22.7, 21.7. HRMS (*m*/*z*): calc. 361.2517; obs. 362.2596 (M+H)^+^. HPLC data: purity – 97.78%, Retention time – 2.32 min.

#### 2-(4-Methylbenzyl)-1-(2-(piperidin-1-yl)­ethyl)-1*H*-benzo­[*d*]­imidazole Hydrochloride (**31**)

White powder. Yield – 27%. ^1^H NMR (400 MHz, δ ppm, DMSO-*d*_6_) 11.75 (bs, 1H), 8.20–8.13 (m, 1H), 7.79 (dd, *J* = 6.9, 2.0 Hz, 1H), 7.58 (m, 2H), 7.43 (d, *J* = 8.0 Hz, 2H), 7.24 (d, *J* = 7.8 Hz, 2H), 5.09–4.98 (m, 2H), 4.69 (s, 2H), 3.41 (t, *J* = 5.2 Hz, 4H), 2.95 (dt, *J* = 14.0, 9.3 Hz, 3H), 2.32 (s, 3H), 1.86 (dd, *J* = 14.1, 8.4 Hz, 4H), 1.41 (m, 1H). ^13^C NMR (100 MHz, δ ppm, DMSO-*d*_6_) 153.9, 137.6, 132.2, 130.4, 130.1, 129.8, 126.4, 125.9, 115.2, 113.3, 52.7, 52.4, 31.1, 22.7, 21.7, 21.1. HRMS (*m*/*z*): calc. 333.2204; obs. 334.2287 (M+H)^+^. HPLC data: purity – 99.33%, Retention time – 2.31 min.

#### 4-((1-(2-(Piperidin-1-yl)­ethyl)-1*H*-benzo­[d]­imidazol-2-yl)­methyl)­benzonitrile Hydrochloride (**32**)

White powder. Yield – 27%. ^1^H NMR (400 MHz, δ ppm, DMSO-*d*_6_) 11.57 (bs, 1H), 8.07 (d, *J* = 7.9 Hz, 1H), 7.92–7.87 (m, 2H), 7.73 (dd, *J* = 8.1, 6.2 Hz, 3H), 7.57–7.45 (m, 2H), 4.98 (t, *J* = 8.1 Hz, 2H), 4.79 (s, 2H), 3.07 (m, 2H), 2.97 (q, *J* = 13.4, 11.0 Hz, 3H), 1.92–1.79 (m, 4H), 1.79–1.69 (m, 1H), 1.42 (dd, *J* = 12.3, 6.9 Hz, 1H). ^13^C NMR (100 MHz, δ ppm, DMSO-*d*_6_) 152.7, 133.2, 131.0, 125.6, 119.1, 112.8, 111.0, 52.7, 45.9, 31.8, 22.7, 21.7, 8.9. HRMS (*m*/*z*): calc. 344.2000; obs. 345.2067 (M+H)^+^. HPLC data: purity – 99.23%, Retention time – 2.32 min.

#### Biological Evaluation Drugs

Morphine (morphine sulfate pentahydrate salt) and fentanyl was purchased from Mallinckrodt (St. Louis, MO) or provided by the National Institute on Drug Abuse (NIDA). All drugs and test compounds were dissolved in pyrogen-free isotonic saline (Baxter Healthcare, Deerfield, IL) or sterile-filtered distilled/deionized water. All other reagents were purchased from either Sigma-Aldrich or Thermo Fisher.

#### Calcium Mobilization Assay

This assay was performed following previously reported procedure.^74^ Chinese Hamster Ovary cells expressing the mouse MOR were used to perform this assay. In brief, the mMOR-CHO cells were cultured with Dulbecco’s Modified Eagle Medium F12 (DMEM/F-12) media with 10% Fetal Bovine Serum (FBS) at 37 °C and 5% CO_2_. For the transfection, cells were transfected with Gqi4 cDNA using lipofectamine 2000 [ratio 1:2 (w/v)] in OptiMEM reduced-serum media. The cells were incubated at 37 °C with 5% CO_2_ for 20–24 h. Post transfection the cells were plated in a black 96-well plate with clear bottoms at 20,000 cells/well for 44–48 h. Assay buffer was prepared by using Hanks’ Balanced Salt Solution (HBSS), 4-(2-hydroxyethyl)­piperazine-1-ethane-sulfonic acid (HEPES), probenecid, 1 mM CaCl_2_, 1 mM MgCl_2_. Loading buffer was prepared by mixing assay buffer, probenecid, and Fluo-4 AM solution (used as fluorescent reagent).

For agonism assay, post incubation of the 96-well plate (assay plate), the culture media was aspirated and 50 μL of dye-loading buffer (Fluo-4 AM dye and assay buffer) was added to the plate, followed by 1 h incubation. In the meantime, a source plate (a separate 96-well plate) was prepared which contained different concentrations of the test compounds. After preincubation with dye-loading buffer, 80 μL assay buffer was added to the assay plate containing the MOR-CHO cells and later 20 μL of test compound solution was autotransferred from the source plate. The assay plate was read on a FlexStation3 microplate reader at ex494/em516 at 37 °C. Upon MOR activation (via Gqi4-mediated signaling), an increase in fluorescence intensity corresponds to an increase in intracellular calcium concentration. The fluorescence signal was monitored and captured, and the peak height was obtained by using SoftMaxPro software. For the antagonism assay, the cells were incubated with dye-loading buffer for 1 h and then it was aspirated. After that, 60 μL of assay buffer followed by 20 μL of the test compound (different concentrations) was added to the assay plate and incubated at 37 °C with 5% CO_2_ for 15 min. In the meantime, the source plate was prepared by adding the positive control (DAMGO, fentanyl, or etonitazene) and blank (assay buffer), and later 20 μL was auto transferred to the assay plate while reading. All experiments were performed at least three times, and each concentration were tested in triplicates. The nonlinear regression curves were generated by using GraphPad Prism 10.3 and the corresponding IC_50_ values were determined.

#### Animals

Male Swiss-Webster mice (25–35 g, 7–8 weeks, Envigo Laboratories, Indianapolis, IN) were housed five to a cage in animal care quarters maintained at 22 °C on a 12 h light/dark cycle with food and water available ad libitum. Protocols and procedures (Animal Welfare Assurance Number D16–00180) were approved by the Institutional Animal Care and Use Committee (IACUC) at the Virginia Commonwealth University Medical Center and complied with the recommendations of the IASP (International Association for the Study of Pain).

#### Warm-Water Tail Immersion Assay

This assay was performed to determine the antinociceptive potential of the synthesized compounds by using Swiss Webster male mice as reported previously.^75^ The mice were brought to the laboratory (22 ± 2 °C, 12 h light–dark cycle) and allowed 18 h to recover from the transport. The tail-flick test was performed using a water bath with the temperature maintained at 56 ± 0.1 °C. Each mouse was acclimatized to the lab environment and handling 24 h prior to the experiments and was gently wrapped in a soft cloth allowing secure handling with only the tail exposed. Baseline latency was measured before s.c. injection of the compounds. The distal one-third of the tail was immersed perpendicularly in water, and the mouse rapidly flicked his tail from the bath at the first sign of discomfort. The duration of time the tail remained in the water bath was counted as the baseline latency. Untreated mice with baseline latency reaction times ranging from 2 to 4 s were used. Test latency was obtained 20 min after the agonist injection. A 10 s maximum cutoff latency was used to prevent any tissue damage. Antinociception was quantified as the percentage of maximal possible effect (% MPE), which was calculated as % MPE = [(test latency – control latency)/(10 – control latency)] × 100. The % MPE value was calculated for each mouse using six mice per compound. If the compound was evaluated for its antagonizing effects against morphine, fentanyl or etonitazene, the compound was s.c. injected 5 min prior to the agonist administration.

#### Statistical Analysis

One-way ANOVA followed by the posthoc Dunnett test were performed to assess the significance using GraphPad Prism software (San Diego, CA).

#### In Vitro Hepatic Metabolism S9 Fraction Incubation

The assay was performed following previously reported procedures.^76^ Briefly, 1 μM of compound **26** or reference compounds were tested in 0.3 mg/mL human liver S9 plus 1 mM UDPGA (Uridine-5′-diphospho-α-d-glucuronic acid) or CD-1 mouse liver S9 plus 1 mM UDPGA, respectively. At time 0, 15, 30, 45, and 60 min of incubation, the concentration of each compound was determined using LC-MS/MS. After the experiment, metabolic stability, expressed as percent of the parent compound remaining, was calculated by comparing the peak area of the compound at the time point relative to that at time 0. The half-life (*t*_1/2_) was estimated from the slope of the initial linear range of the logarithmic curve of compound remaining (%) vs time, assuming the first-order kinetics. The apparent intrinsic clearance (CL_int_, in μL/min/mg) was calculated according to the following formula: CL_int_= 0.693/*t*_1/2_*­(mg protein/μL)

#### Caco-2 Cell Permeability^77^

Human epithelial colorectal adenocarcinoma (Caco-2) cells (HTB-37) were cultured in T75 flasks using complete DMEM containing 10% FBS, 1% glutamine, 1% penicillin and 1% streptomycin, at 37 °C in a 5% CO_2_ atmosphere. Cells were passaged at 80–90% confluency using 0.05% trypsin-EDTA and the medium was changed every other day. Following this, the cells were trypsinized, suspended in medium and applied to a Millipore 96-well plate where they were cultured as monolayers at a density of 25,000 cells/well. The cells were incubated in a 37 °C/5% CO_2_ incubator to allow cell attachment and proliferation. Media was changed every 2–3 days for 21 days when cells reached 100% confluency. For Apical→ Basolateral (A→B) permeability, 10 μM compound **26** or controls in the presence or absence of inhibitors were added to the apical (A) side and the amount of permeation determined on the basolateral (B) side; for Basolateral→ Apical (B→A) permeability, 10 μM compound **26** or controls in the presence or absence of inhibitors were added to the B-side and the amount of permeation was determined on the A side. The A-side buffer contained 100 μM lucifer yellow dye, in Transport Buffer (1.98 g/L glucose in 10 mM HEPES, 1x HBSS) pH 7.4, and the B-side buffer used was the Transport Buffer at pH 7.4. Caco-2 cells were incubated with compounds in these buffers for 1 h. At the end of the assay, donor and receiver side solution samples were collected, quenched by 100% methanol containing an internal standard and centrifuged at 5000 rpm for 10 min at 4 °C. Following centrifugation, the supernatant for donor and receiver side samples was analyzed by HPLC-MS/MS to determine peak area ratios. Fluorescein assessment for Permeability assays: Fluorescein was used as the cell monolayer integrity marker. Fluorescein permeability assessment (in the A-B direction at pH 7.4 on both sides) was performed after the permeability assay for the test compound. The cell monolayer that had a fluorescein permeability of less than 1.5 × 10^–6^ cm/s for Caco-2 was considered intact, and the permeability result of the test compound from intact cell monolayer is reported.

The apparent permeability coefficient (Papp) of the test compound was calculated as follows:

$$\mathrm{Papp}\left(\mathrm{cm}/s\right)={\frac{{\mathrm{VR}}^{*}\mathrm{CR},\mathrm{end}}{\Delta T}}^{*}\frac{1}{A^{*}\left(\mathrm{CD},\mathrm{mid}-\mathrm{CR},\mathrm{mid}\right)}$$

where VR is the volume of the receiver chamber. CR,end is the concentration of the test compound in the receiver chamber at the end time point, Δt is the incubation time and A is the surface area of the cell monolayer. CD,mid is the calculated midpoint concentration of the test compound in the donor side, which is the mean value of the donor concentration at time 0 min and the donor concentration at the end time point. CR,mid is the midpoint concentration of the test compound in the receiver side, which is one-half of the receiver concentration at the end time point. Concentrations of the test compound were expressed as peak areas of the test compound.

The Efflux Ratio (R_E_) was calculated as

$$R_{E}=\frac{Papp\left(B\to A\right)}{Papp\left(A\to B\right)}$$

#### Molecular Docking Studies

Etonitazene and compound **26** were drawn using Sybylx2.1, assigned Gasteiger–Huckel charges, and energy minimized with the Tripos force field. Etonitazene was docked in the active conformation (PDB ID 8EF5)^66^ while compound **26** was docked in the active and inactive conformation (PDB ID 9BJK)^78^ of the MOR. Protein structures were prepared for docking by adding hydrogen atoms, deleting water molecules and bound ligands inside the binding pocket. GOLD 2020 ^79^ a genetic algorithm docking program was used to dock the ligands, and the binding site was defined to include all atoms within 10 Å of cocrystallized ligands. A distance constraint of 4 Å between the 19N of the compounds and D147 carboxylate group in the MOR was applied. The molecules were docked into the proteins with a total of 100 iterations. To optimize the structural models for the ligand–protein complexes, docking was followed by energy minimization under Tripos force field in Sybylx2.1. CHEMPLP score, which has been optimized for modeling steric complementarity between ligand and protein along with distance and angle-dependent hydrogen bonding, was used to obtain plausible docking poses. Optimal docking poses for each ligand–protein complex were chosen based on highest ChemPLP. Figures are generated using PyMOL version 1.7.4.

## Supplementary Material













## Notes

Calcium mobilization single concentration agonism assay results of etonitazene and nitazene analogs at the MOR; calcium mobilization assay concentration response curves in agonist and antagonist-mode of identified hits; spectra data for target compounds (^1^H NMR, ^13^C NMR, and MS); purity data of target compounds (HPLC chromatograms) (PDF)
Highest scored docking pose 1 of etonitazene at the active MOR (PDB)
Highest scored docking pose 2 of etonitazene at the active MOR (PDB)
Highest scored docking pose of compound **26** at the active MOR (PDB)
Highest scored docking pose of compound **26** at the inactive MOR (PDB)
Molecular formula strings and some data (CSV)

## Abbreviations Used

AD_50_: anti-antinociceptive dose
BBB: blood brain barrier
CNS: central nervous system
CHO: Chinese hamster ovary
DAMGO: d-Ala2-MePhe4-Gly­(ol)­5]­enkephalin
DCM: dichloromethane
DEA: Drug Enforcement Administration
ECL: extracellular loop
FDA: Food and Drug Administration
GPCRs: G protein-coupled receptors
HBSS: Hanks’ balanced salt solution
HEPES: 4-(2-hydroxyethyl)­piperazine-1-ethane-sulfonic acid
HPLC: high-performance liquid chromatography
IC_50_: inhibitory concentration
MPE: maximum potential effect
MOR: mu-opioid receptor
NTX: naltrexone
NSOs: new synthetic opioids
NMR: nuclear magnetic resonance
OUD: opioid use disorder
PLC: phospho lipase C
PDB: Protein Data Bank
RMSD: root mean square deviation
SAR: structure–activity relationship
TM: transmembrane
WWTI: warm-water tail immersion
