# Supplementary material for: Landscaping the Nitazene Scaffold to Identify Novel Mu-Opioid Receptor Modulators: From Molecular Design and Chemical Synthesis to Pharmacological Profiling
Source: J Med Chem. 2026 Jul 6;69(14):16658–76. doi: 10.1021/acs.jmedchem.6c00283 (PMC13403233; doi:10.1021/acs.jmedchem.6c00283)
Supplement: Supplementary file 6 [file jm6c00283_si_006.pdf]

**Supporting Information**

# Landscaping the Nitazene Scaffold to Identify Novel Mu-Opioid Receptor Modulators: From Molecular Design, Chemical Synthesis to Pharmacological Profiling

Neha Upadhyay [a], Logan T. Neel [a], Ennian Li [a], Abeje A. Silte [a], Rui Lyu [a], Dana E. Selley [b], William L. Dewey [b], Yan Zhang [a,b,c*], and Piyusha P. Pagare [a,c*]

[a] *Department of Medicinal Chemistry, School of Pharmacy, Virginia Commonwealth University, Richmond, Virginia 23298, United States*

[b] *Department of Pharmacology and Toxicology, Virginia Commonwealth University, Richmond, Virginia 23298, United States*

[c] *Center for Drug Discovery, Virginia Commonwealth University, Richmond, Virginia 23298, United States*

* E-mail: pagarepp@vcu.edu (P. Pagare) and yzhang2@vcu.edu (Y. Zhang)

**Table of Contents**

1. Table **S1**. Calcium mobilization single concentration agonism assay results of etonitazene and nitazene analogs at the MOR. (S3)
2. Figure **S1**. Calcium mobilization agonism assay concentration response curves of DAMGO and identified hits. (S4)
3. Figure **S2**. Calcium mobilization antagonism assay concentration response curves of identified hits against DAMGO, fentanyl and etonitazene. (S5)
4. $^{1}$H NMR, $^{13}$C NMR and MS spectra of final compounds. (S6 to S55)
5. Table **S2**. Purity data of final compounds. (S56 to S57)
6. HPLC chromatograms of final compounds. (S57 to S73)

## 1. Calcium Mobilization Single Concentration Agonism Assay

**Table S1.** Calcium mobilization agonism assay results of nitazene analogs at 10 μM at the MOR. Data are presented as mean RFU values (% of the value of 10 μM DAMGO) ± SEM; n = 3.

| **Compounds** | **Mean RFU Values (% 10 μM DAMGO) ± SEM** | **Compounds** | **Mean RFU Values (% 10 μM DAMGO) ± SEM** |
|---|---|---|---|
| Etonitazene | 81.86 ± 8.08 | **17** | 50.31 ± 14.52 |
| **1** | 12.11 ± 4.53 | **18** | 67.57 ± 2.96 |
| **2** | 30.97 ± 0.36 | **19** | 53.09 ± 3.15 |
| **3** | 67.59 ± 5.92 | **20** | 79.34 ± 6.28 |
| **4** | 53.09 ± 3.76 | **21** | 55.20 ± 5.33 |
| **5** | 17.47 ± 2.61 | **22** | 79.34 ± 3.16 |
| **6** | 5.22 ± 1.11 | **23** | 67.93 ± 4.19 |
| **7** | 68.09 ± 10.27 | **24** | 78.81 ± 3.44 |
| **8** | 81.30 ± 8.59 | **25** | 21.10 ± 1.34 |
| **9** | 27.81 ± 6.22 | **26** | 36.73 ± 2.04 |
| **10** | 61.25 ± 0.99 | **27** | 40.91 ± 4.39 |
| **11** | 71.02 ± 6.46 | **28** | 74.68 ± 1.84 |
| **12** | 75.12 ± 8.47 | **29** | 72.37 ± 7.77 |
| **13** | 55.09 ± 4.29 | **30** | 72.98 ± 1.32 |
| **14** | 73.34 ± 1.60 | **31** | 47.75 ± 2.11 |
| **15** | 63.77 ± 4.91 | **32** | 66.21 ± 7.66 |
| **16** | 71.66 ± 5.25 | | |

## 2. Calcium Mobilization Agonism Assay Concentration Response Curves of DAMGO and Identified Hits

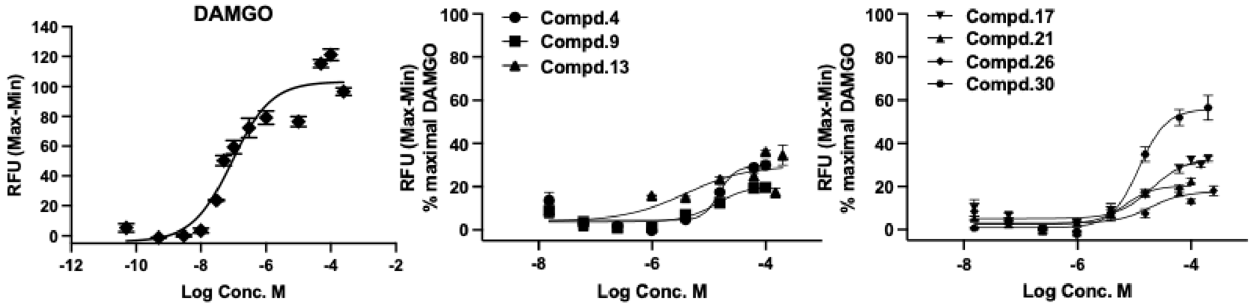


| Compounds | $EC_{50}$ (μM) | $E_{max}$ [% maximal DAMGO] | Compounds | $EC_{50}$ (μM) | $E_{max}$ [% maximal DAMGO] |
|---|---|---|---|---|---|
| **4** | 15.29 ± 0.27 | 29.61 ± 2.38 | **17** | 20.46 ± 15.46 | 33.27 ± 4.39 |
| **9** | 13.73 ± 1.49 | 20.54 ± 4.48 | **21** | 6.92 ± 0.58 | 20.37 ± 0.27 |
| **13** | 3.91 ± 1.39 | 29.81 ± 0.09 | **26** | 19.10 ± 4.39 | 17.72 ± 1.36 |
| | | | **30** | 12.07 ± 0.19 | 55.88 ± 3.56 |

**Figure S1.** Concentration response curves of DAMGO and identified hits. All experiments were performed in at least three replicates, and each concentration was tested in triplicate. Data are presented as mean values ± SEM.

# 3. Calcium Mobilization Antagonism Assay Concentration Response Curves of Naltrexone (NTX) and Identified Hits

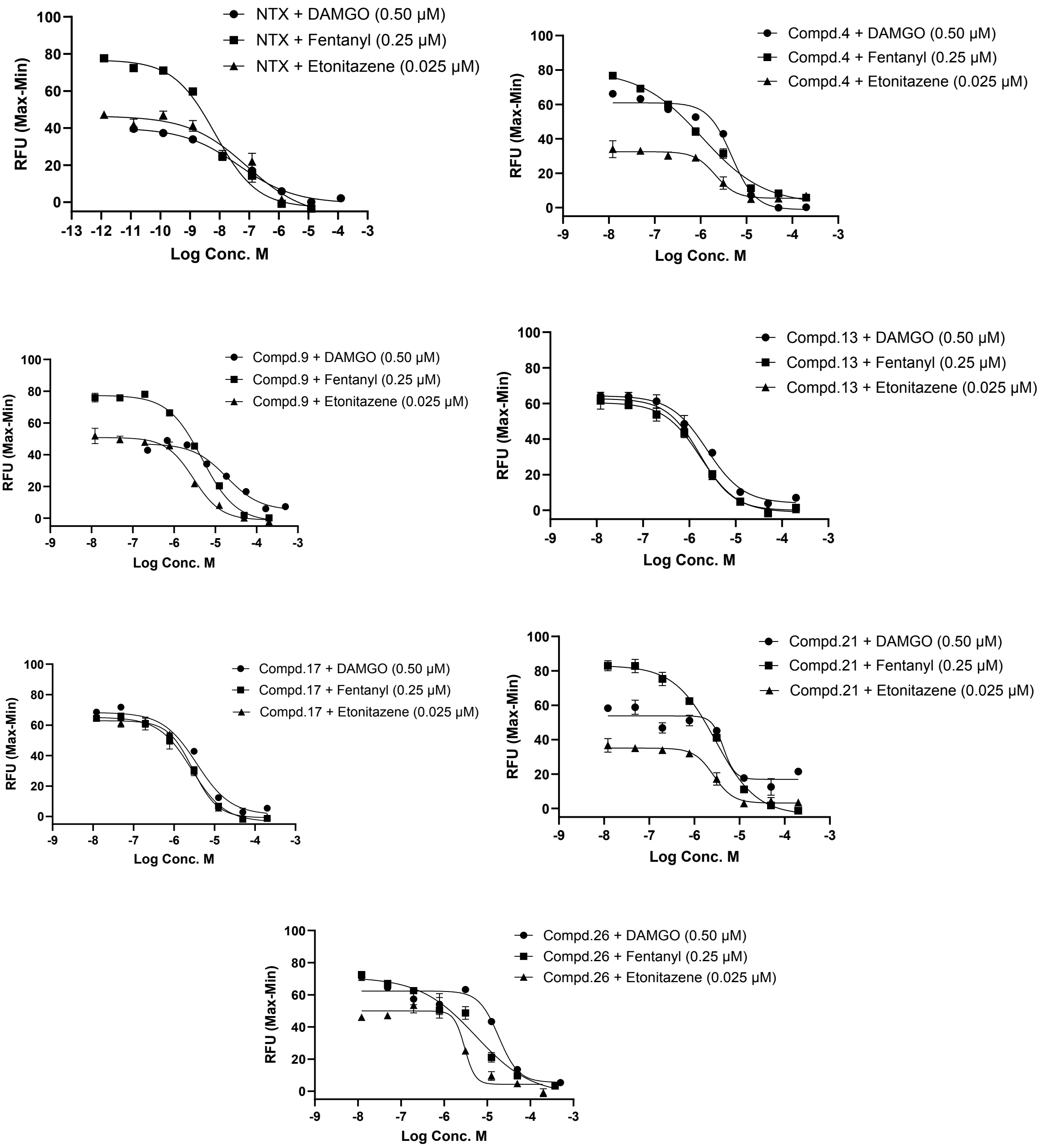


**Figure S2.** Concentration response antagonism curves of identified hits against DAMGO (0.50 μM), Fentanyl (0.25 μM), and Etonitazene (0.025 μM). All experiments were performed at least three times, and each concentration was tested in triplicate. Data are presented as mean values ± SEM; n = 3.

## 4. $^{1}$H NMR, $^{13}$C NMR and MS Spectra of Final Compounds.

**2-(2-(4-ethoxybenzyl)-5-nitro-1*H*-benzo[*d*]imidazol-1-yl)-N,N-diethylethanamine hydrochloride (Etonitazene hydrochloride).**

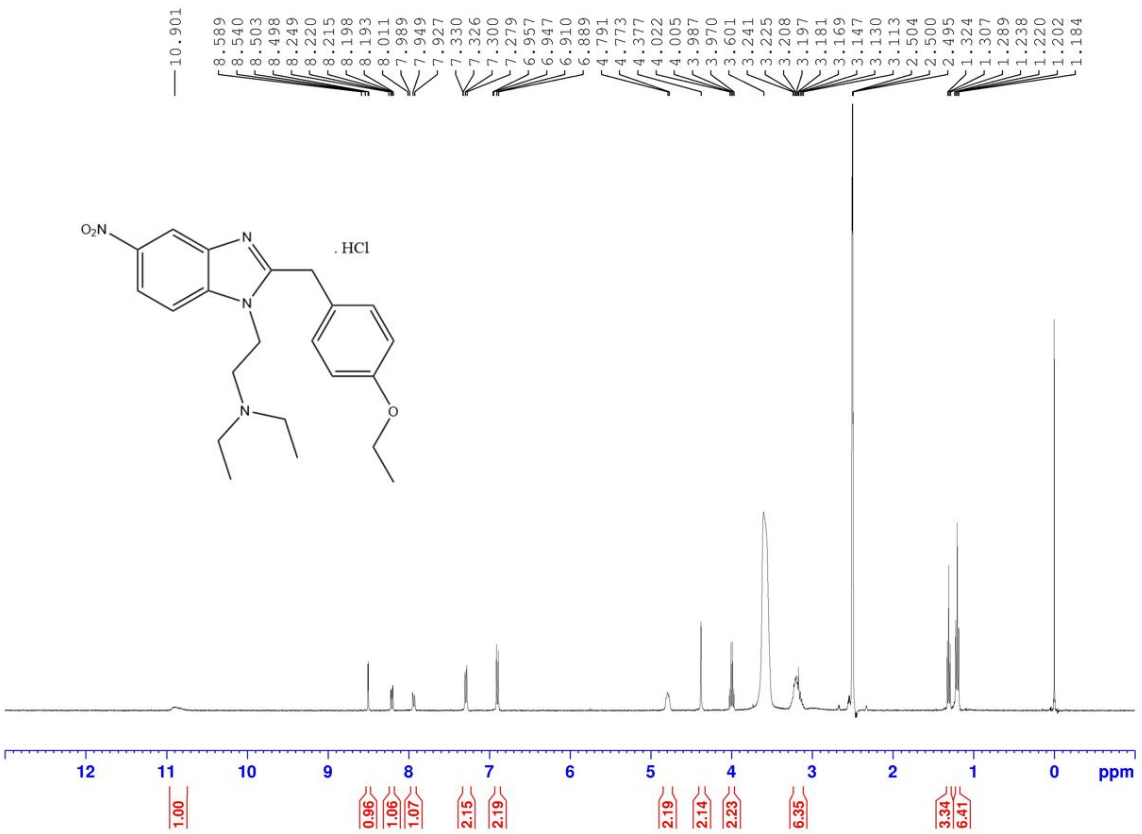


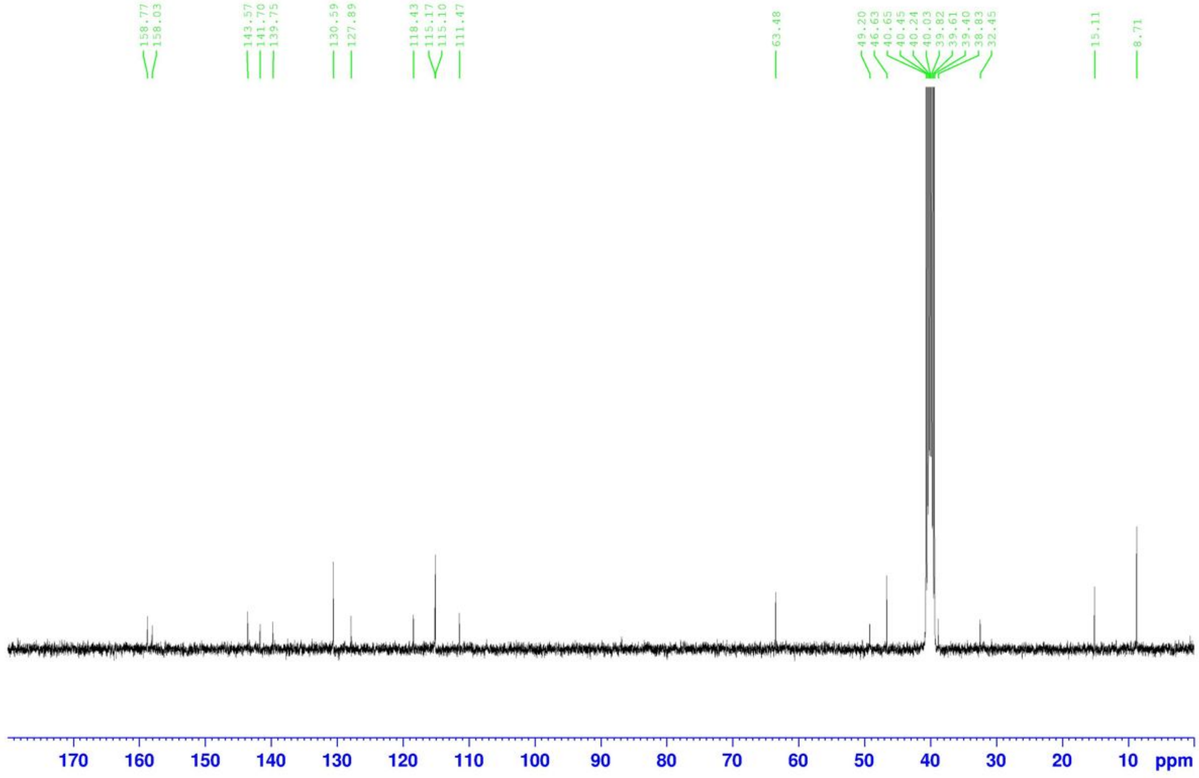

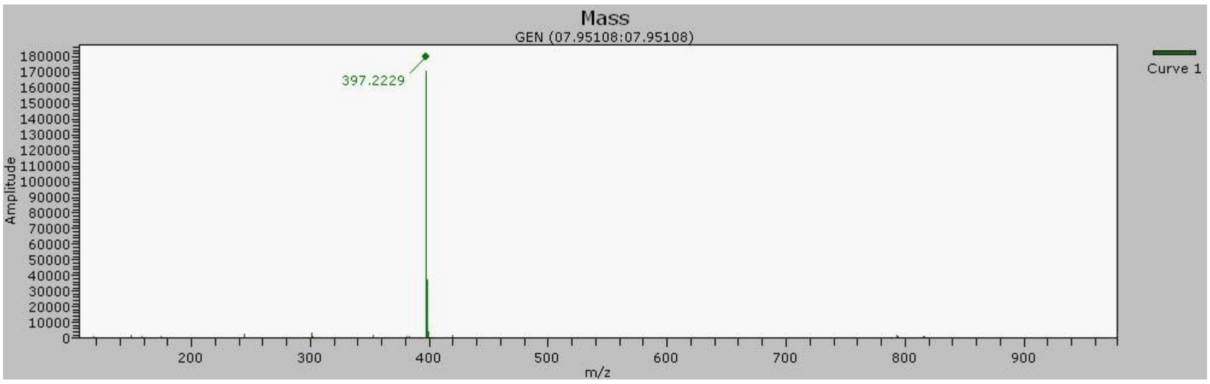


**2-(2-(4-fluorobenzyl)-1*H*-benzo[*d*]imidazol-1-yl)-N,N-dimethylethanamine hydrochloride (1).**

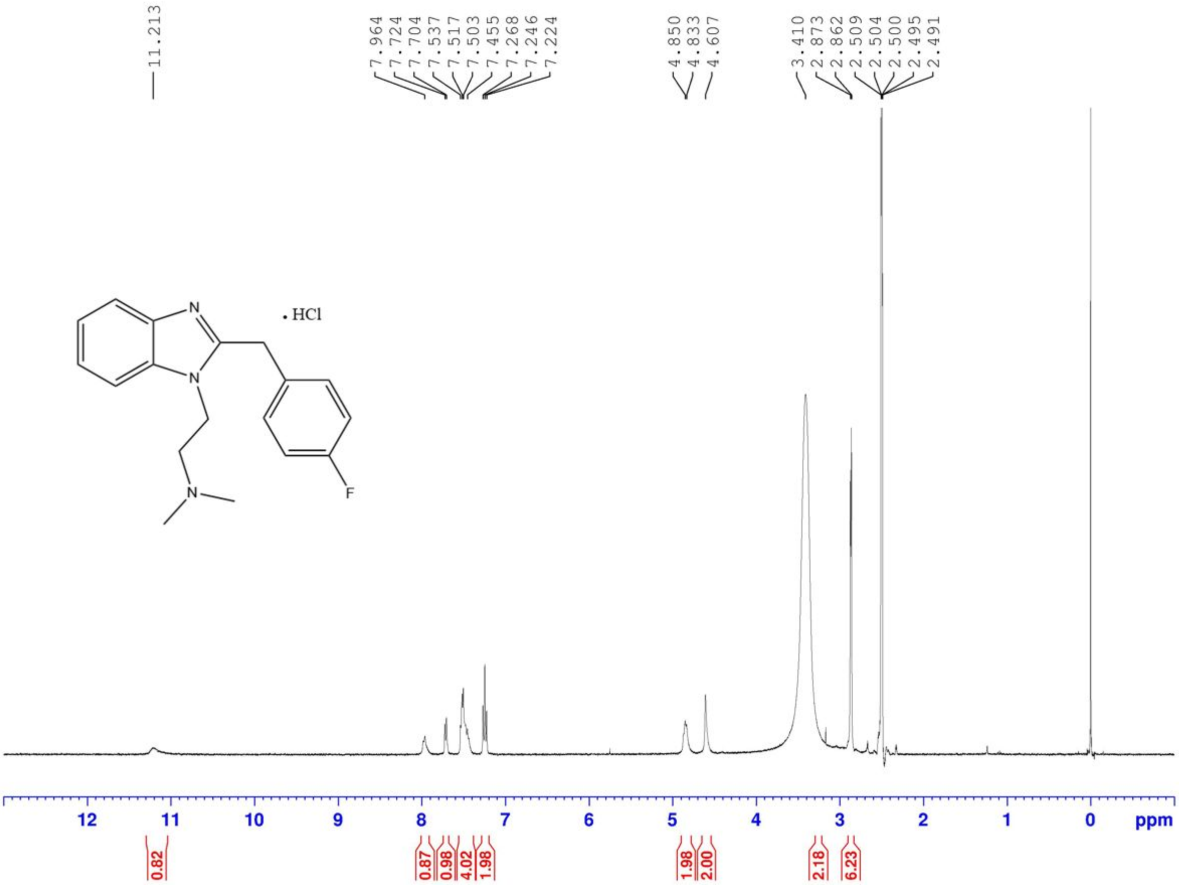

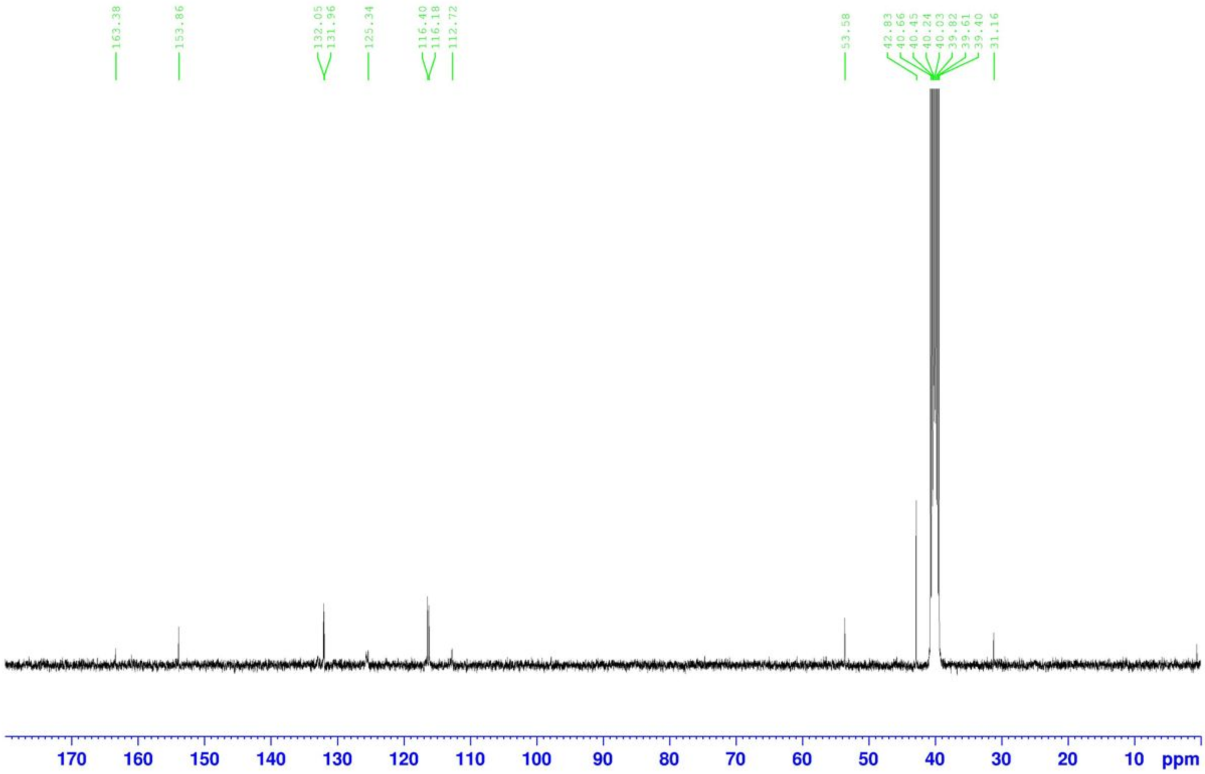
163.38
153.86
132.05
131.96
125.34
116.40
116.18
112.72
53.58
42.83
40.66
40.45
40.24
40.03
39.82
39.61
39.40
31.16
170 160 150 140 130 120 110 100 90 80 70 60 50 40 30 20 10 ppm

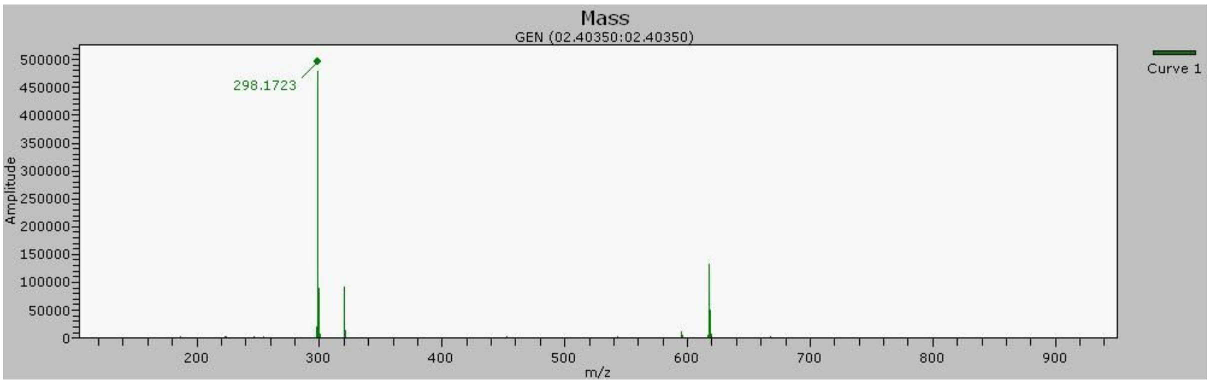
Mass
GEN (02.40350:02.40350)
298.1723
Curve 1
Amplitude
m/z

**N,N-dimethyl-2-(2-(4-methylbenzyl)-1*H*-benzo[*d*]imidazol-1-yl)ethanamine hydrochloride (2).**

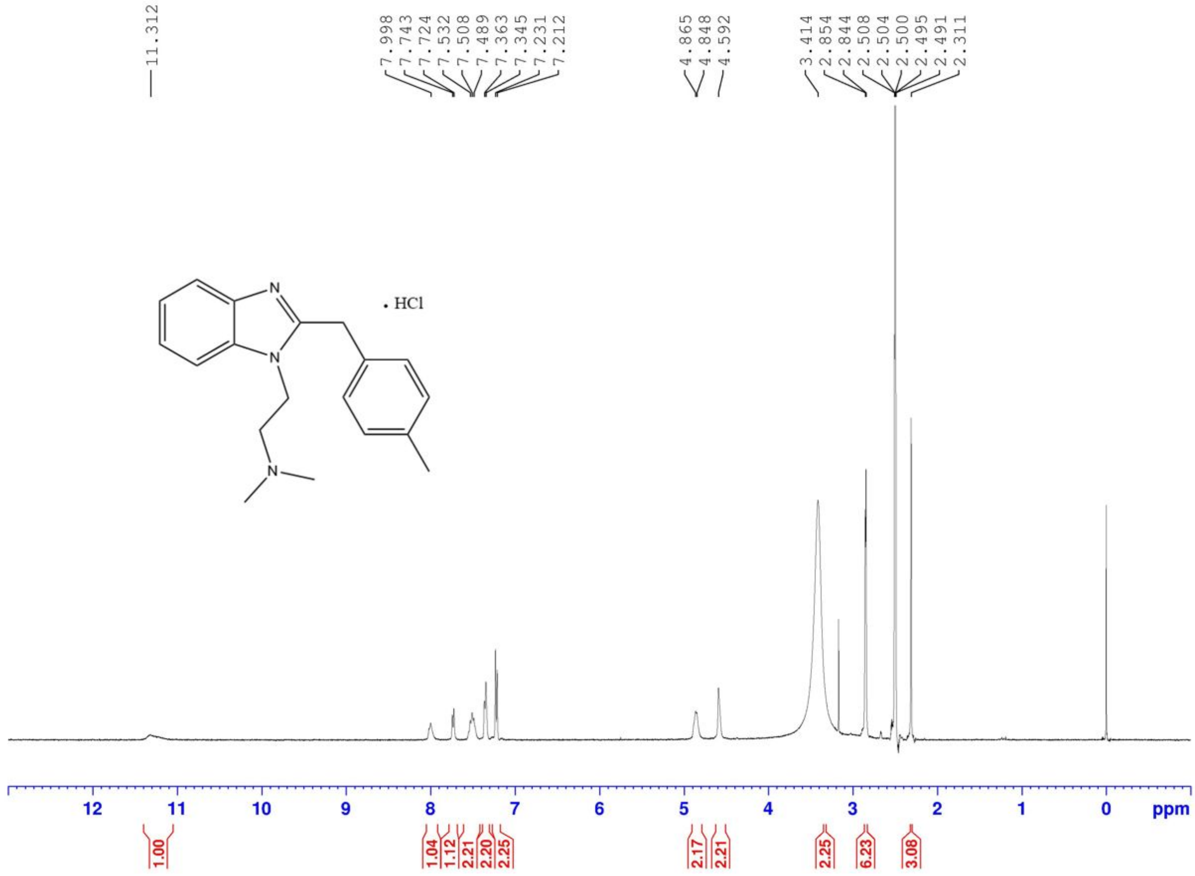


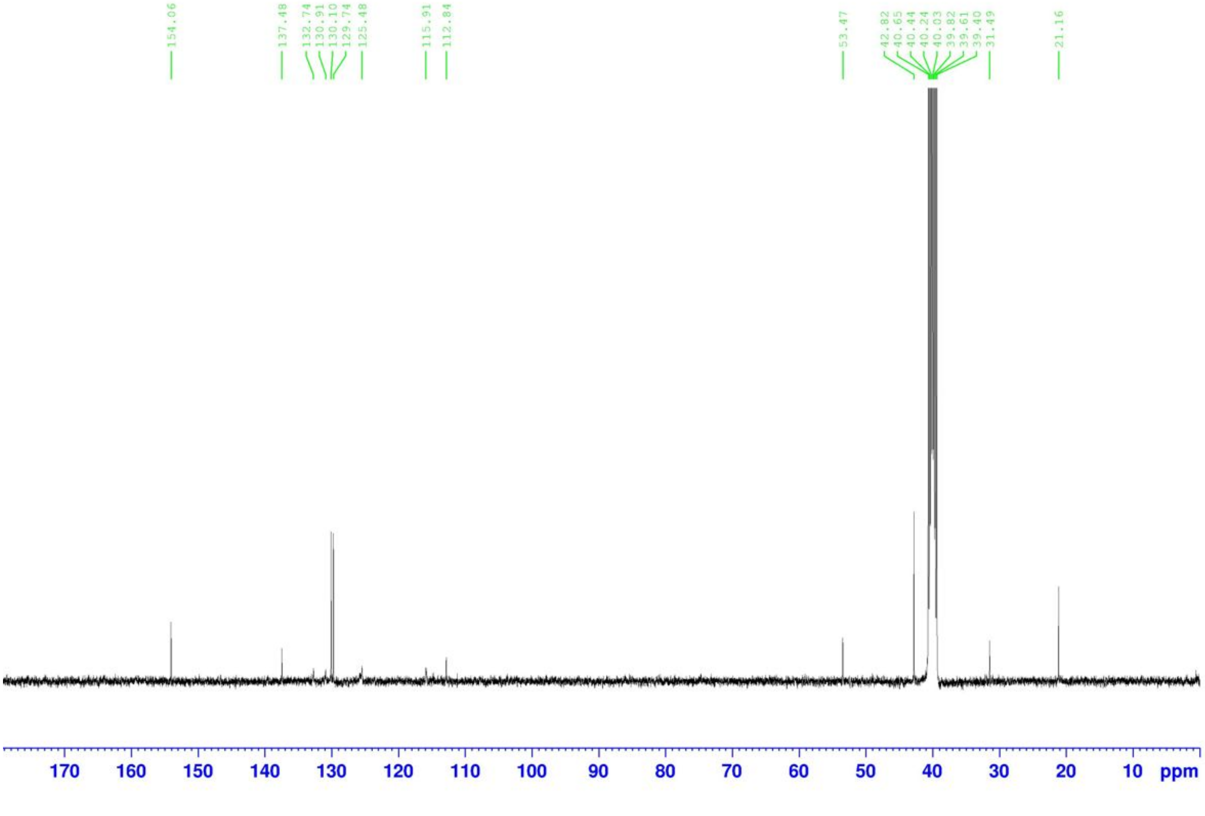

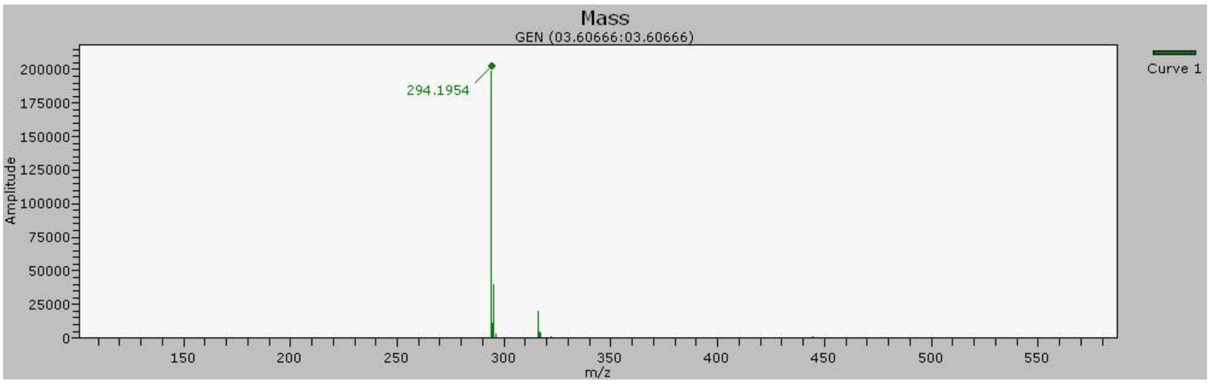


**2-(2-(4-isopropylbenzyl)-1*H*-benzo[*d*]imidazol-1-yl)-N,N-dimethylethanamine hydrochloride (3).**

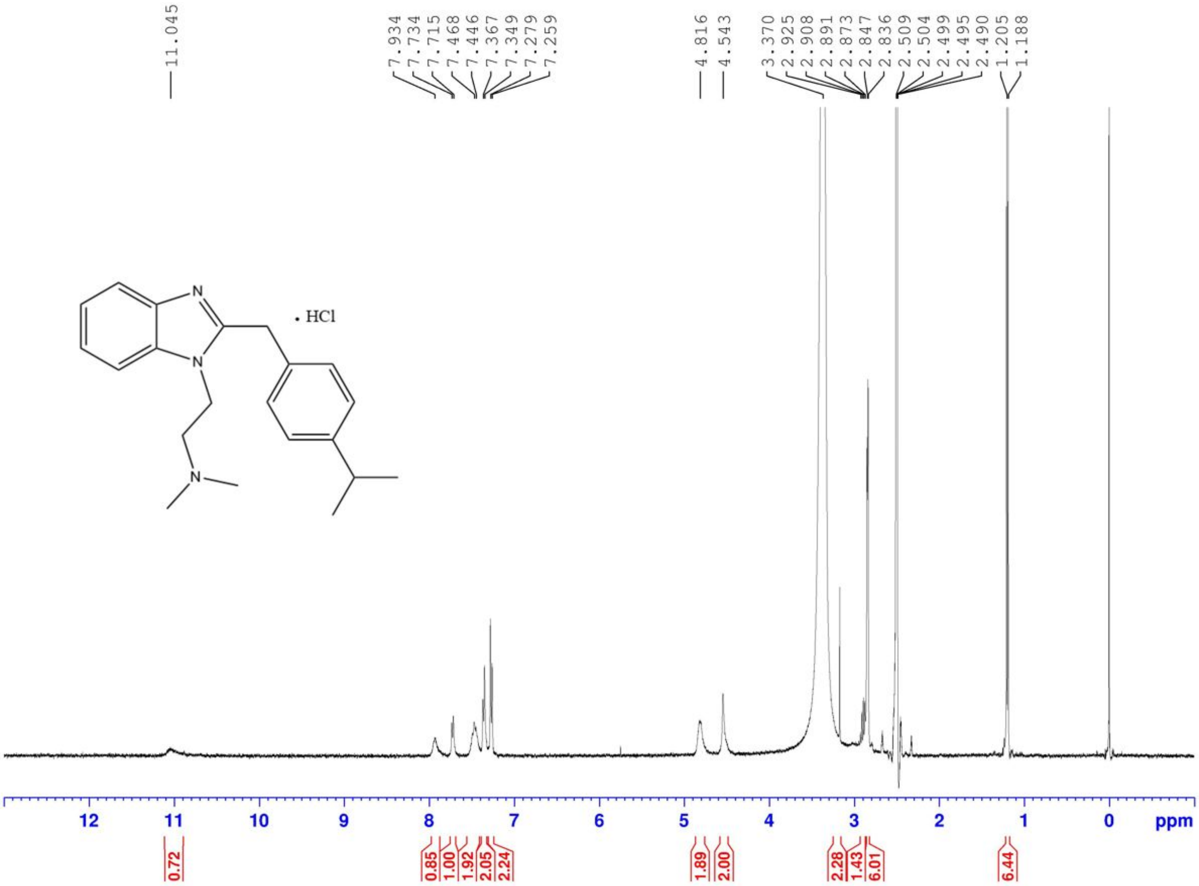

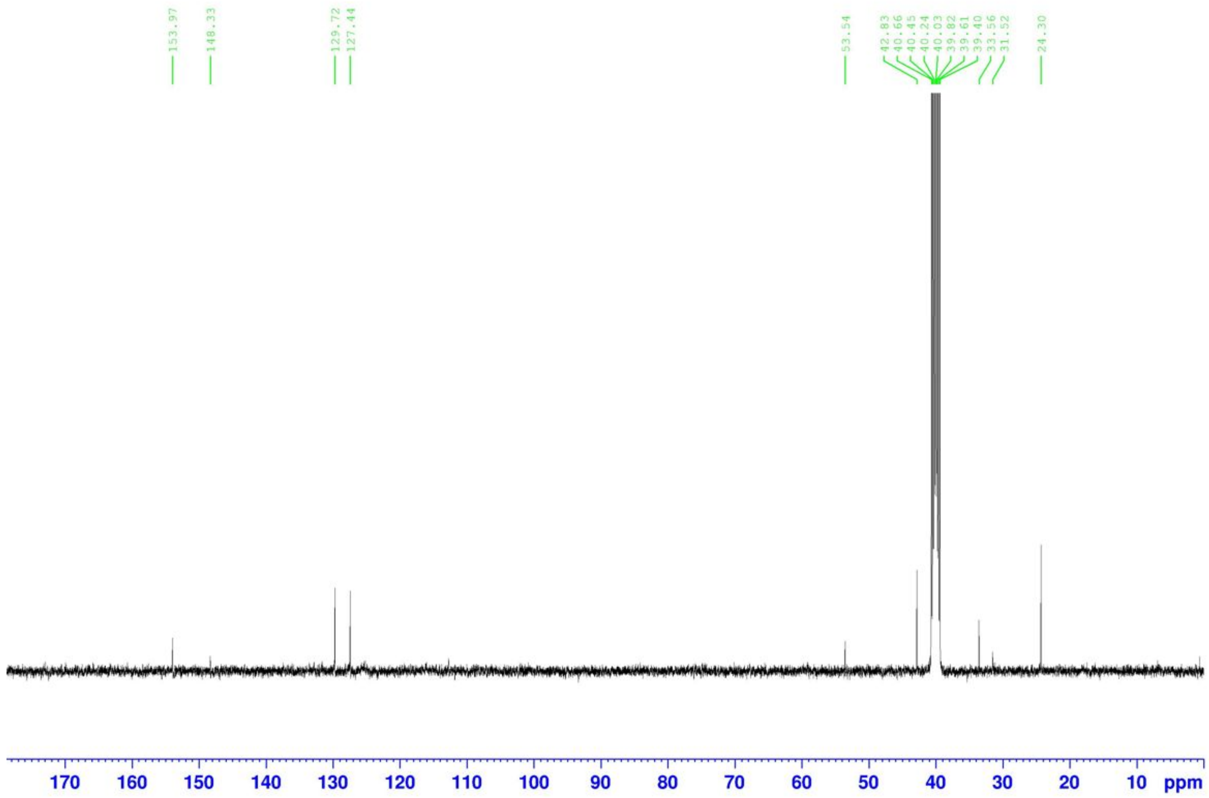
153.97
148.33
129.72
127.44
53.54
42.83
40.66
40.45
40.24
40.03
39.82
39.61
39.40
33.56
31.52
24.30
170 160 150 140 130 120 110 100 90 80 70 60 50 40 30 20 10 ppm

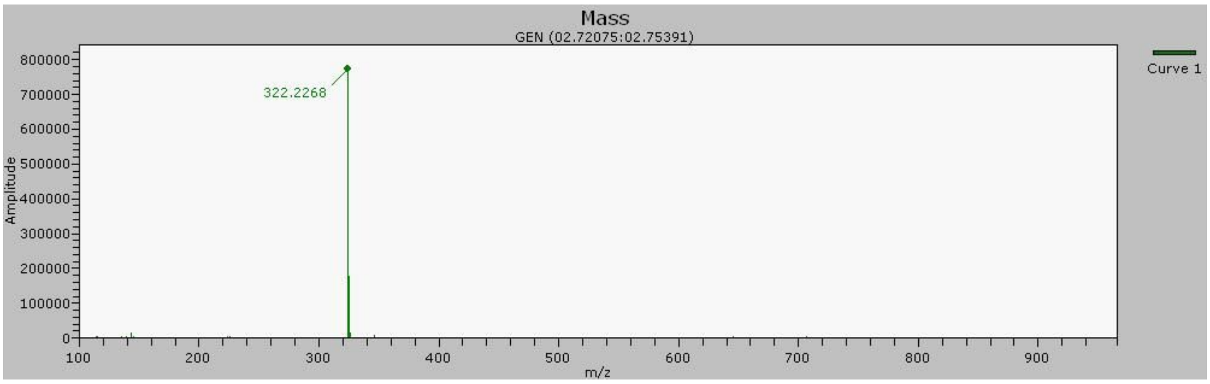
Mass
GEN (02.72075:02.75391)
Curve 1
322.2268
Amplitude
800000
700000
600000
500000
400000
300000
200000
100000
0
100 200 300 400 500 600 700 800 900
m/z

**N,N-dimethyl-2-(2-(4-(trifluoromethyl)benzyl)-1*H*-benzo[*d*]imidazol-1-yl)ethanamine hydrochloride (4).**

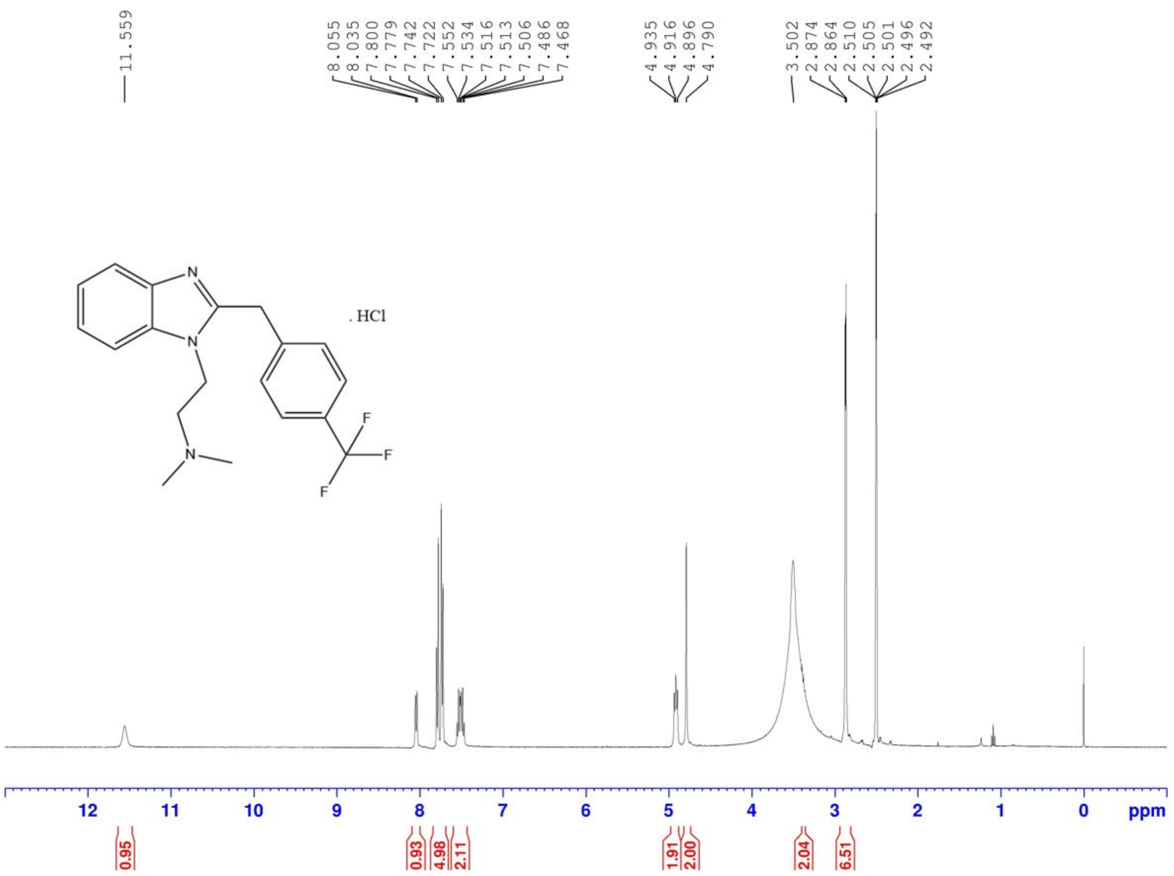


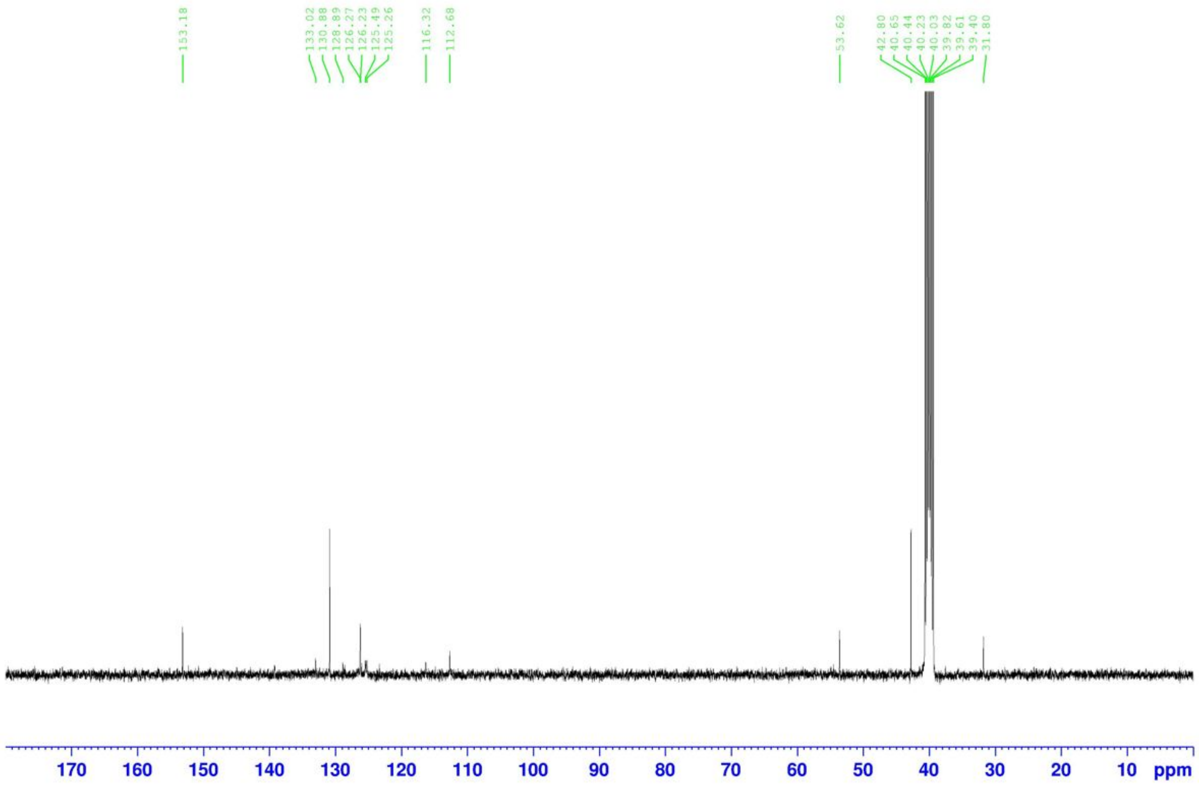

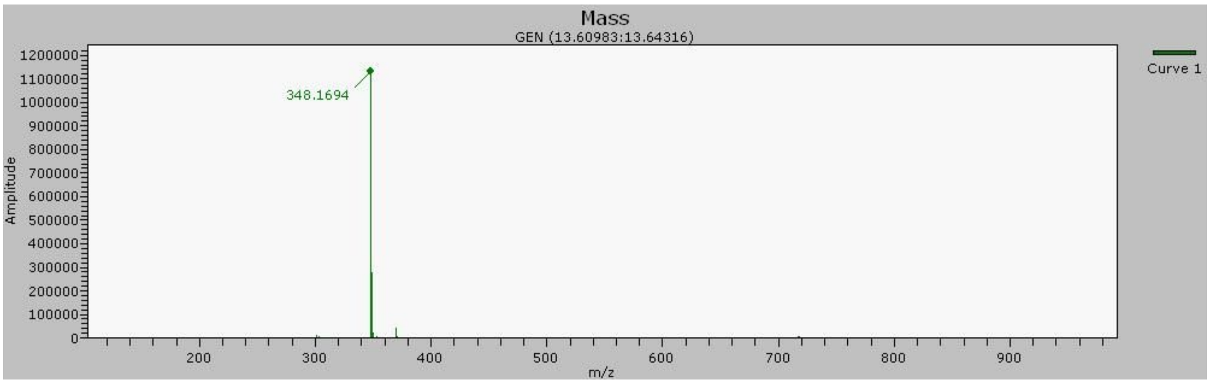


**2-(2-(4-chlorobenzyl)-1*H*-benzo[*d*]imidazol-1-yl)-N,N-dimethylethanamine hydrochloride (5).**

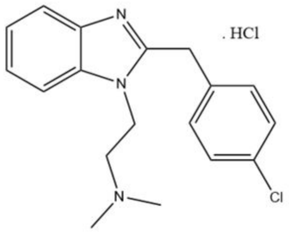


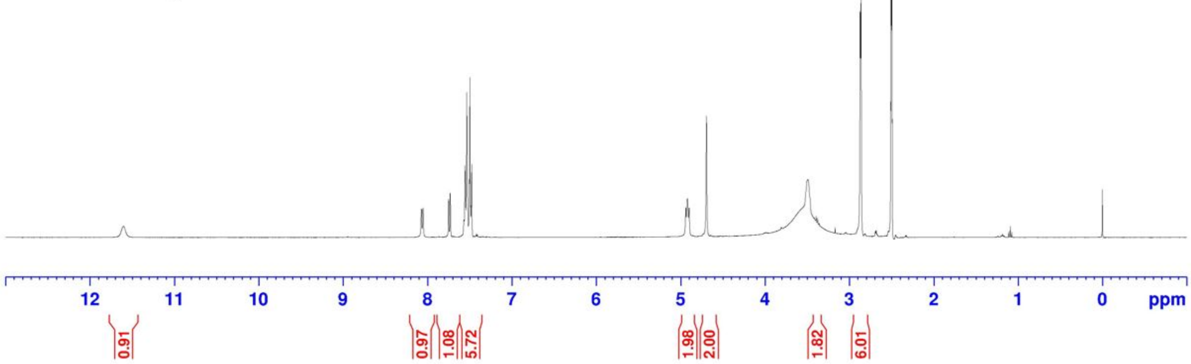

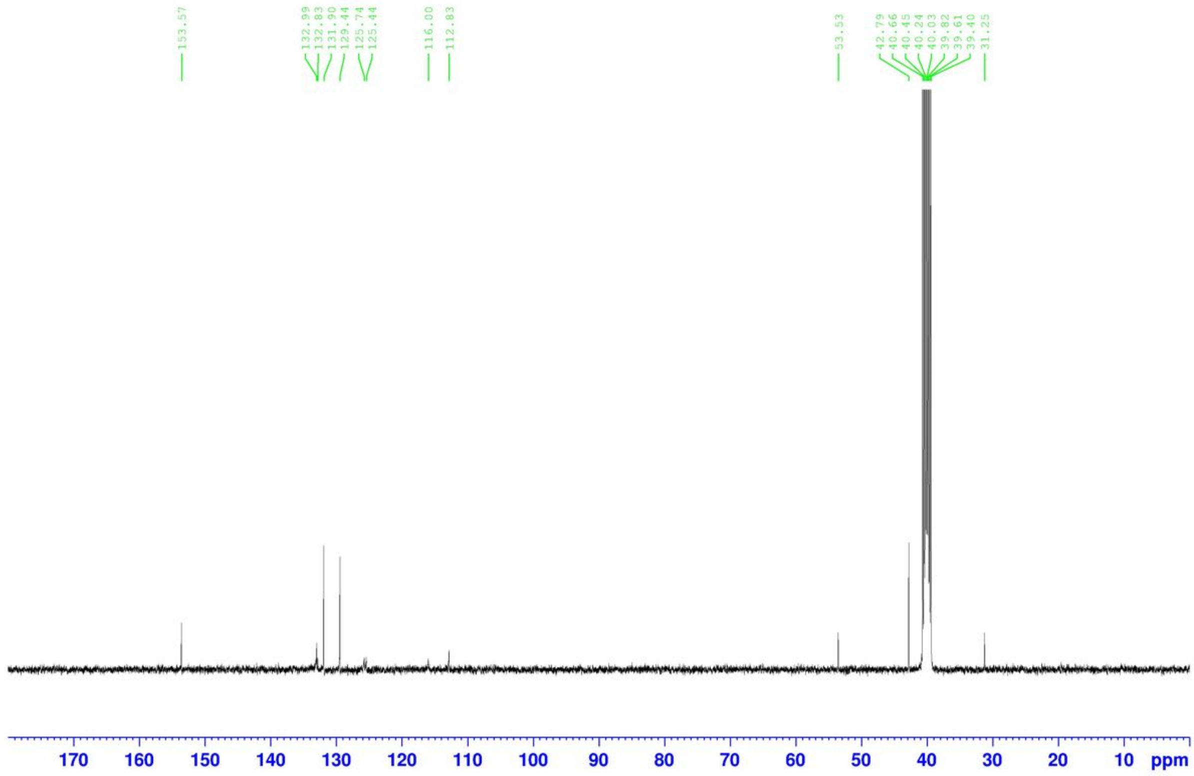
153.57
132.99
132.83
131.90
129.44
125.74
125.44
116.00
112.83
53.53
42.79
40.66
40.45
40.24
40.03
39.82
39.61
39.40
31.25
170 160 150 140 130 120 110 100 90 80 70 60 50 40 30 20 10 ppm

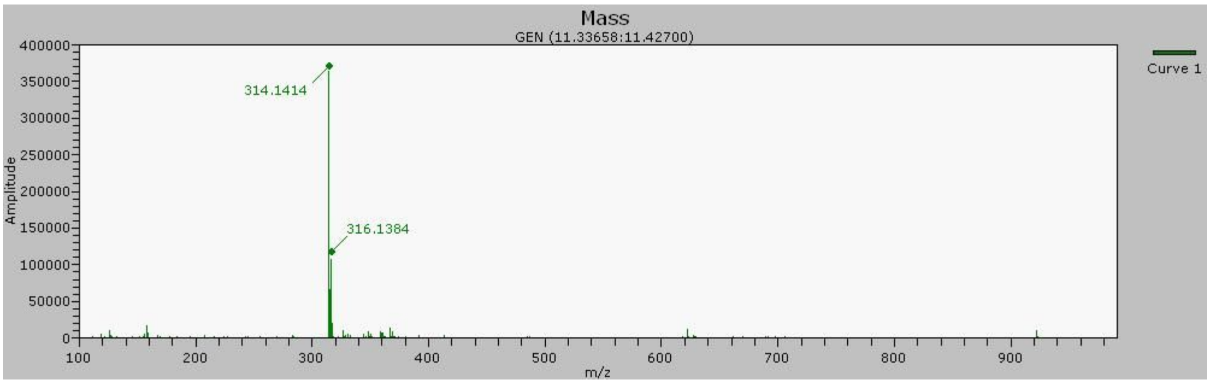
Mass
GEN (11.33658:11.42700)
Curve 1
Amplitude
m/z
314.1414
316.1384

**4-((1-(2-(dimethylamino)ethyl)-1*H*-benzo[*d*]imidazol-2-yl)methyl)benzonitrile hydrochloride (6).**

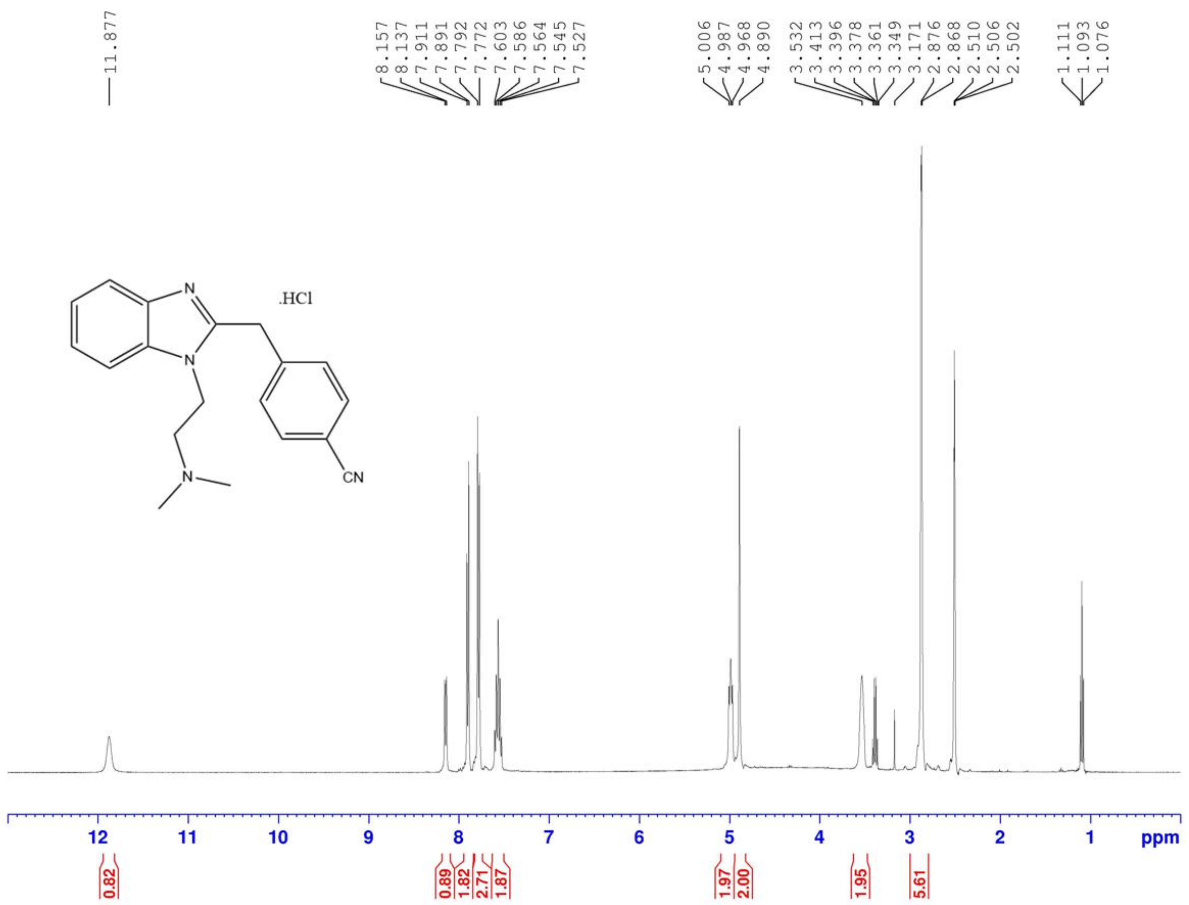

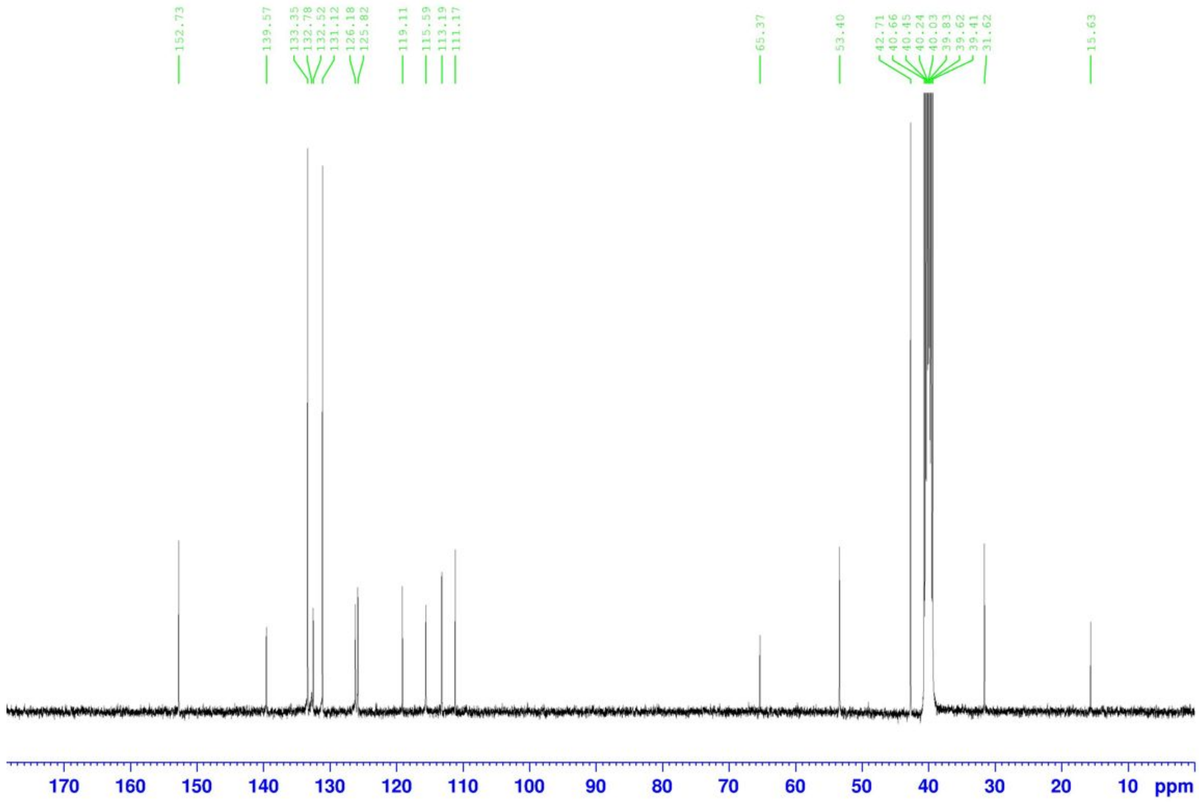
152.73
139.57
133.35
132.78
132.52
131.12
126.18
125.82
119.11
115.59
113.19
111.17
65.37
53.40
42.71
40.66
40.45
40.24
40.03
39.83
39.62
39.41
31.62
15.63
170 160 150 140 130 120 110 100 90 80 70 60 50 40 30 20 10 ppm

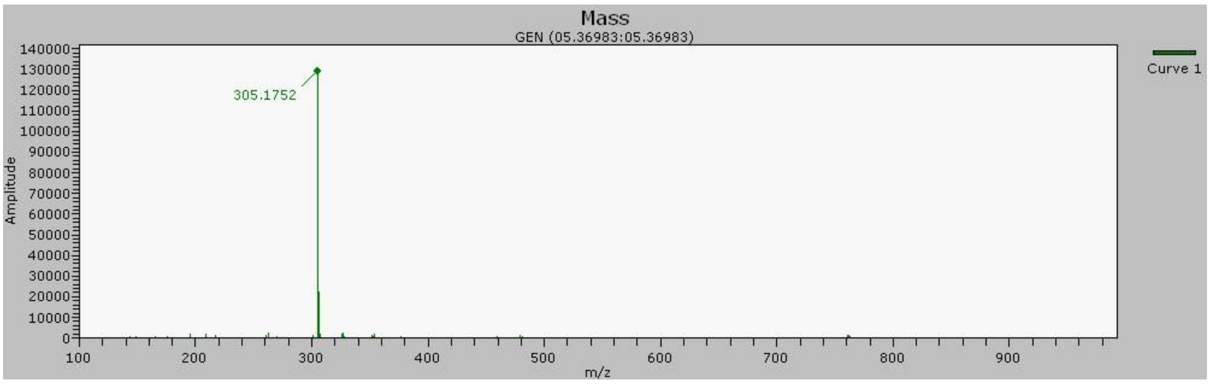
Mass
GEN (05.36983:05.36983)
Curve 1
305.1752
Amplitude
m/z

**2-(2-(4-ethylbenzyl)-1*H*-benzo[*d*]imidazol-1-yl)-*N*,*N*-dimethylethanamine hydrochloride (7).**

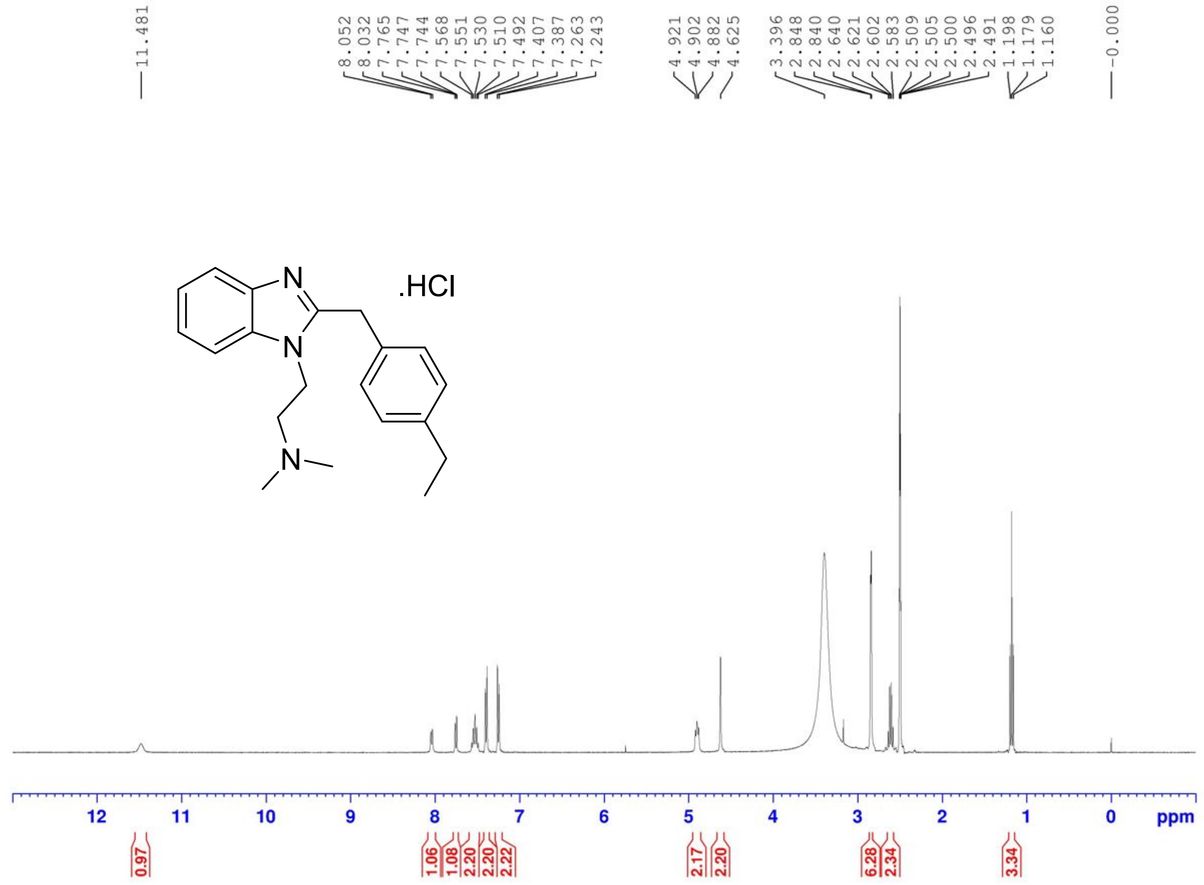


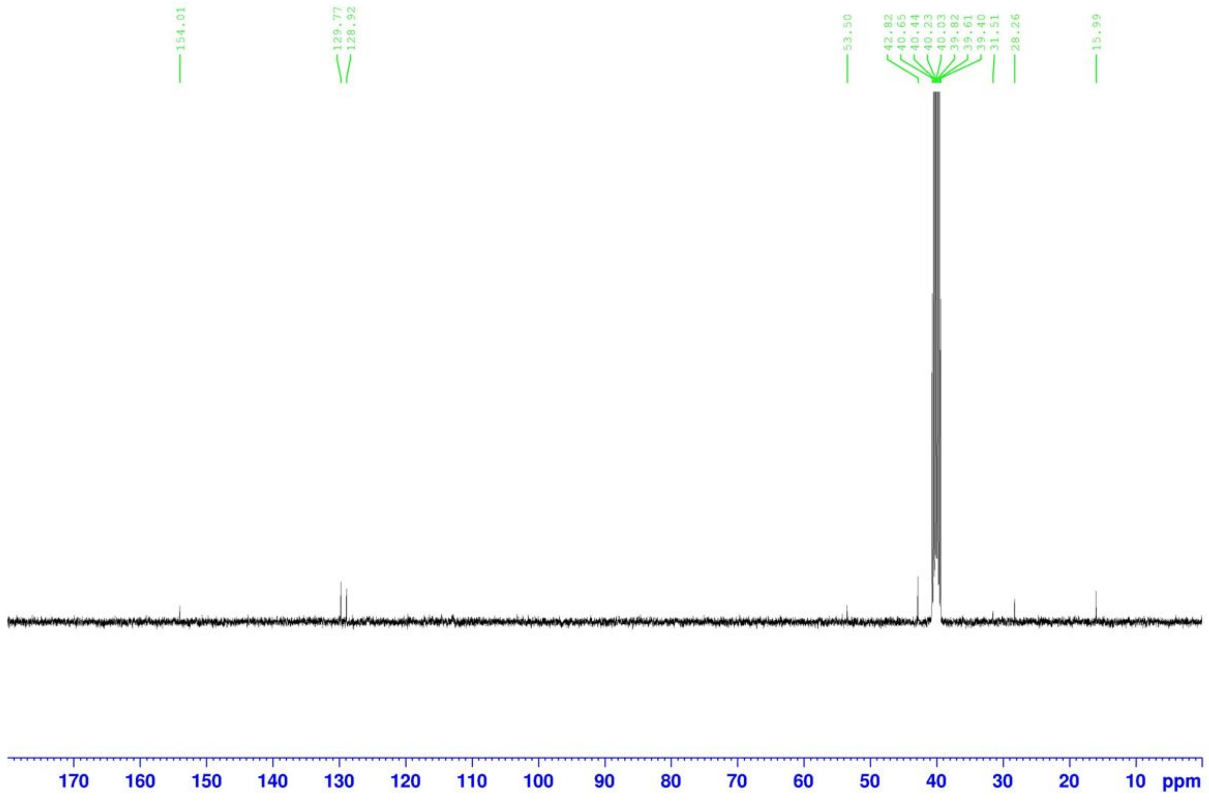

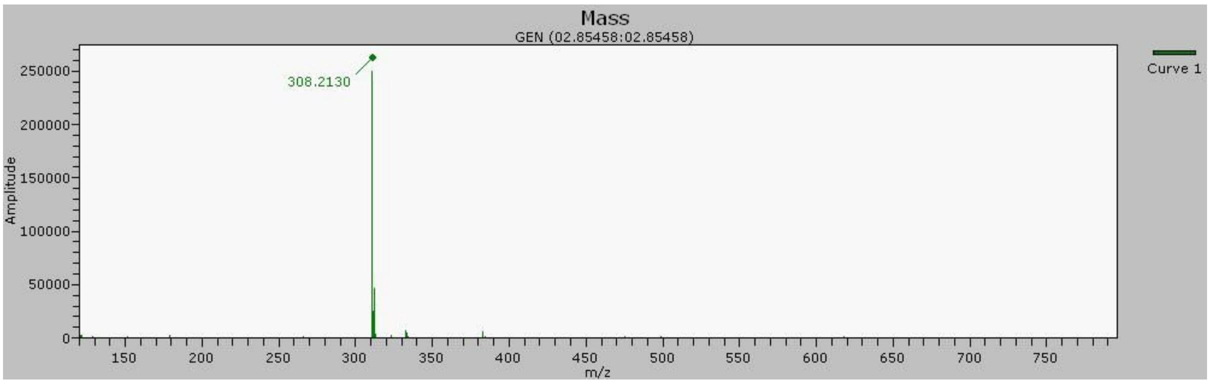


**2-(2-(4-ethoxybenzyl)-1H-benzo[d]imidazol-1-yl)-N,N-dimethylethan-1-amine hydrochloride (8).**

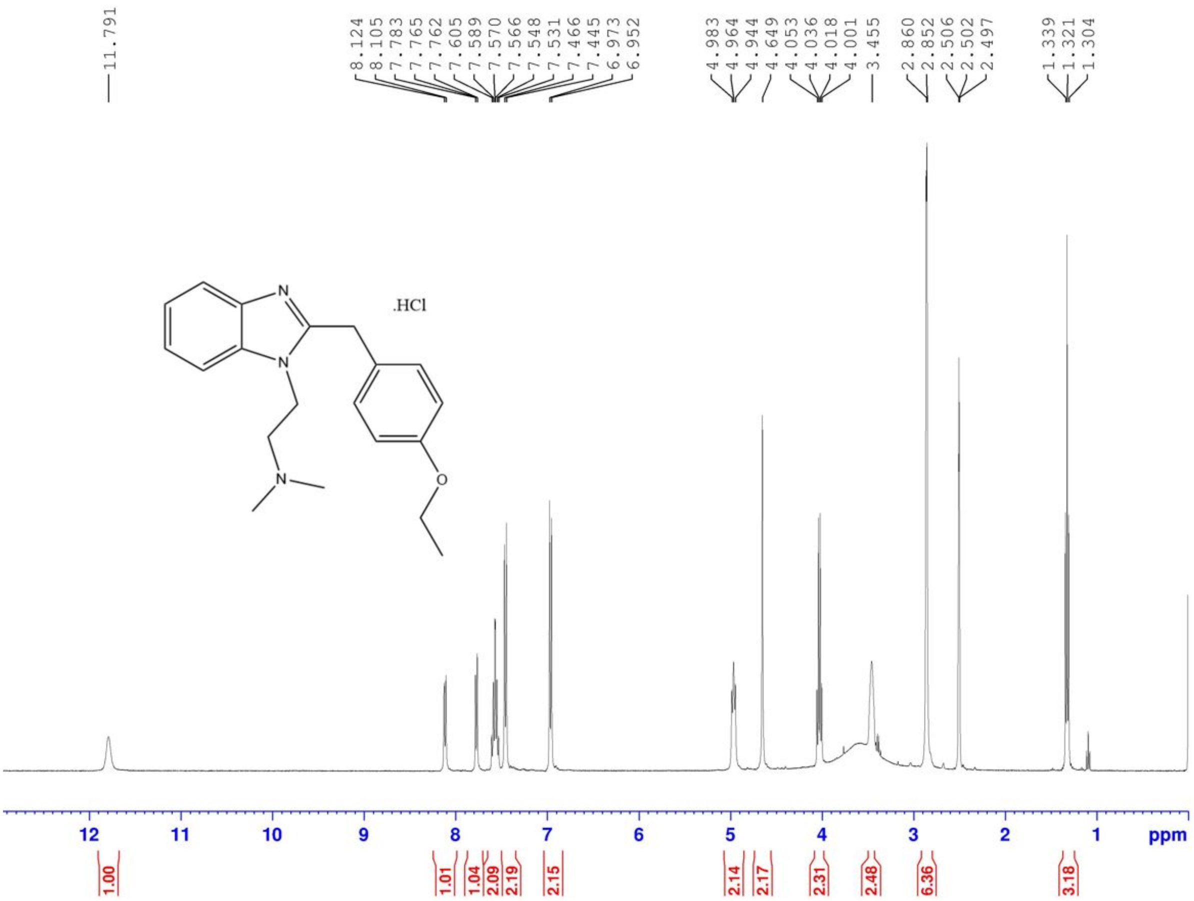

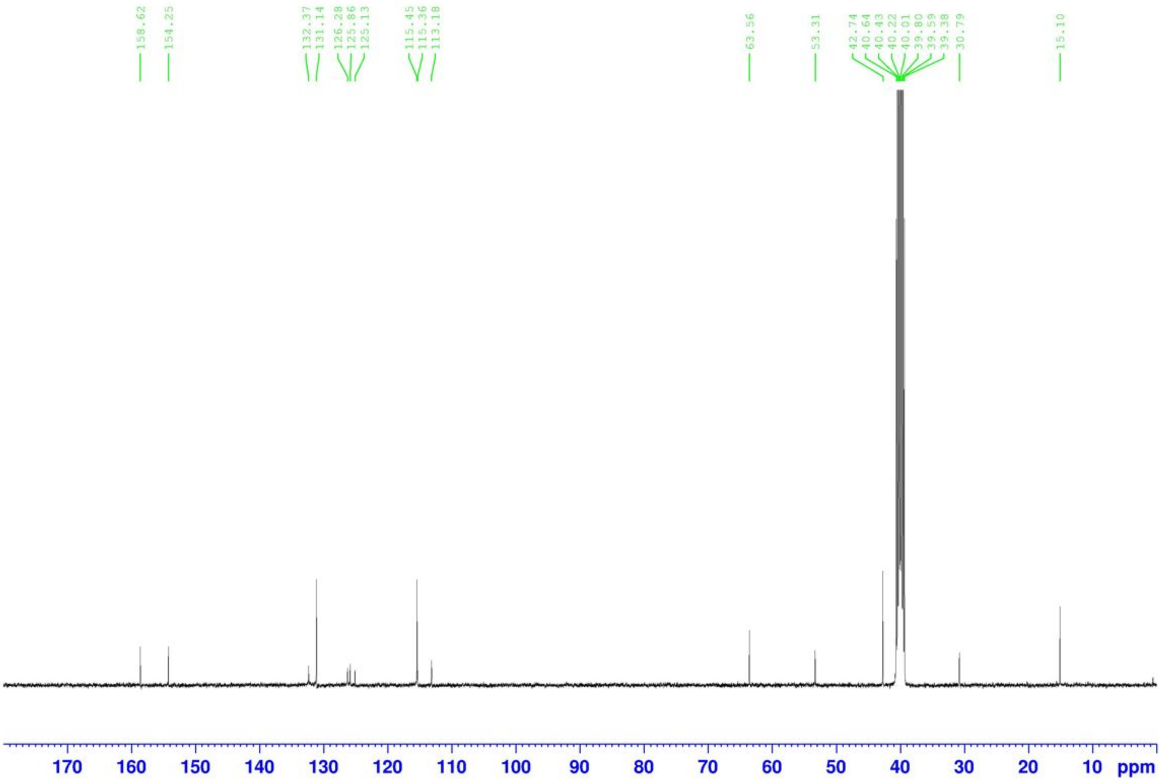
158.62
154.25
132.37
131.14
126.28
125.86
125.13
115.45
115.36
113.18
63.56
53.31
42.74
40.64
40.43
40.22
40.01
39.80
39.59
39.38
30.79
15.10
170 160 150 140 130 120 110 100 90 80 70 60 50 40 30 20 10 ppm

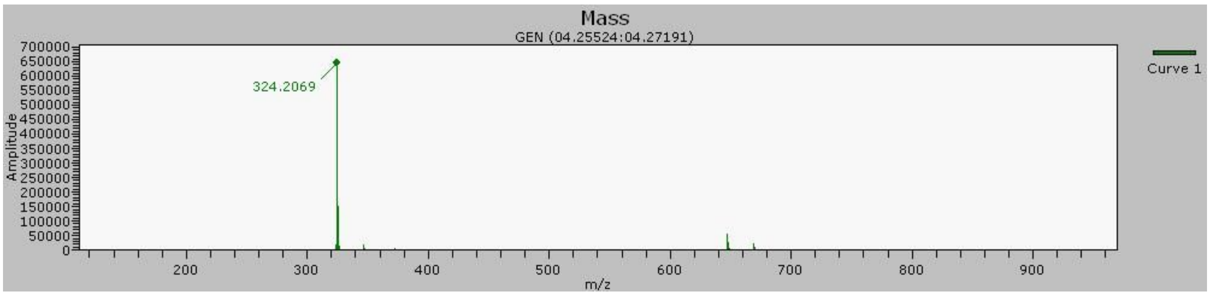
Mass
GEN (04.25524:04.27191)
Curve 1
324.2069
Amplitude
m/z

**N,N-diethyl-2-(2-(4-fluorobenzyl)-1*H*-benzo[*d*]imidazol-1-yl)ethan-1-amine hydrochloride (9).**

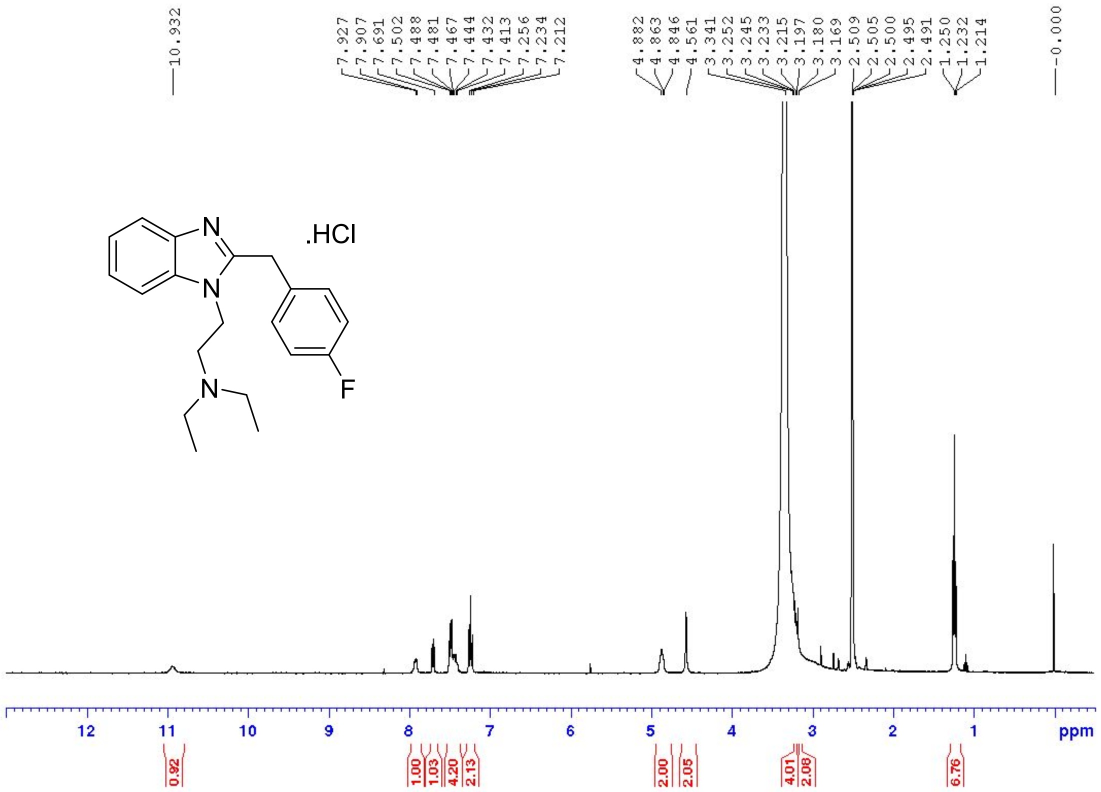


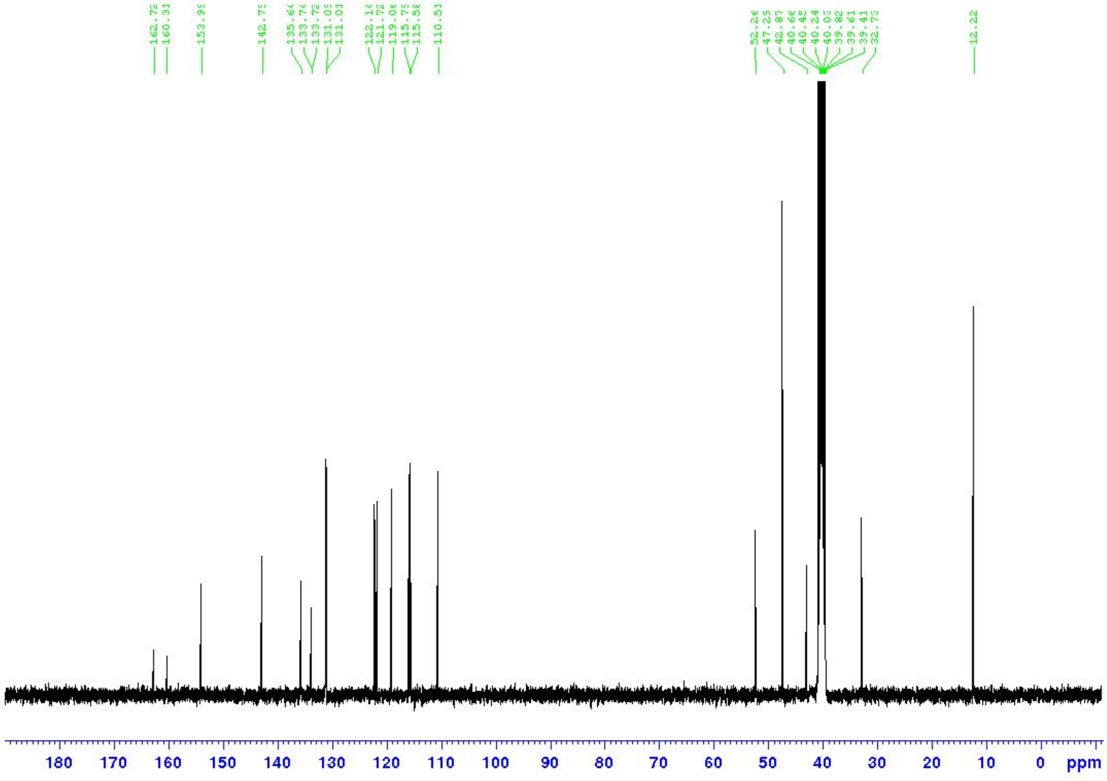

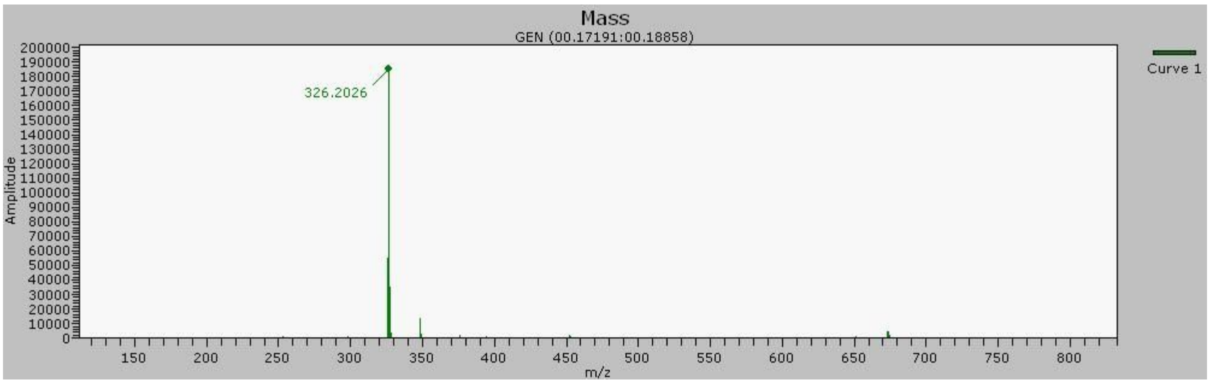


**2-(2-(4-chlorobenzyl)-1*H*-benzo[*d*]imidazol-1-yl)-N,N-diethylethan-1-amine hydrochloride (10).**

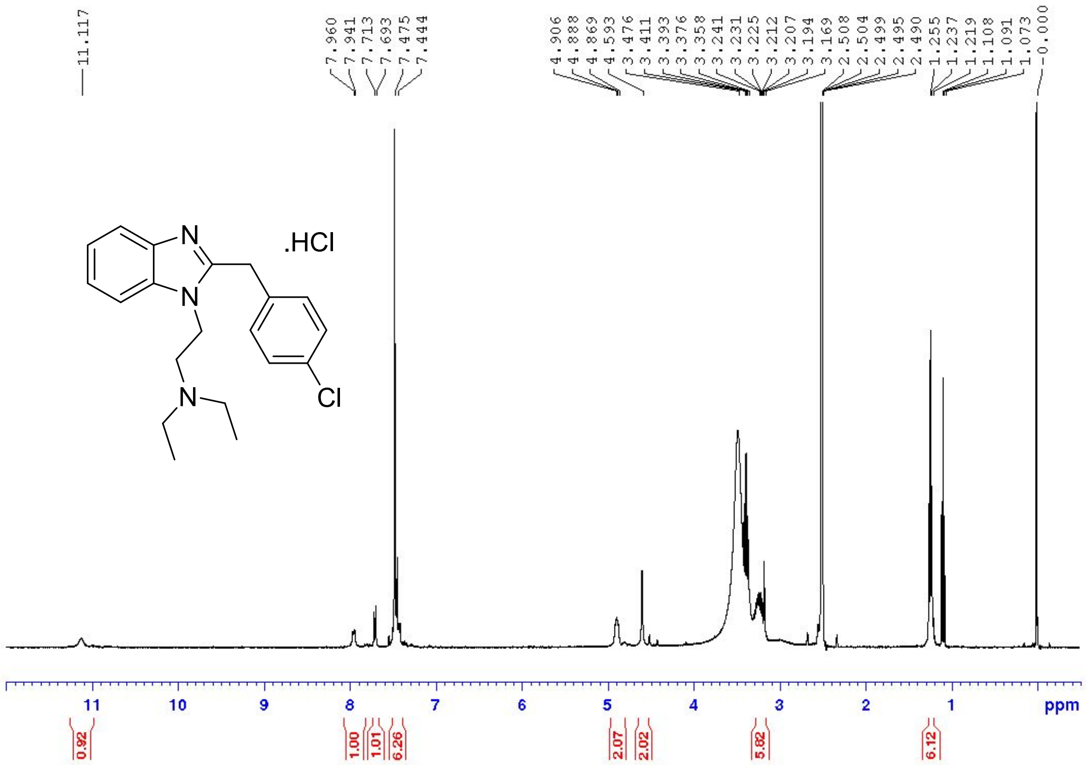

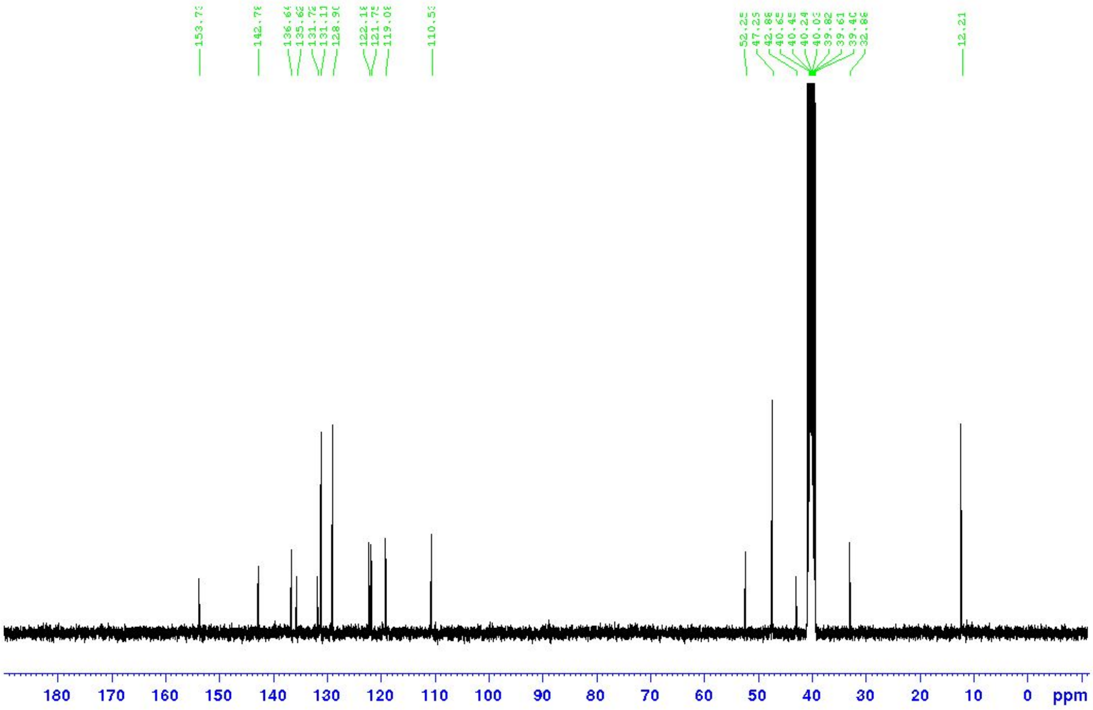

12.21
180
170
160
150
140
130
120
110
100
90
80
70
60
50
40
30
20
10
0
ppm


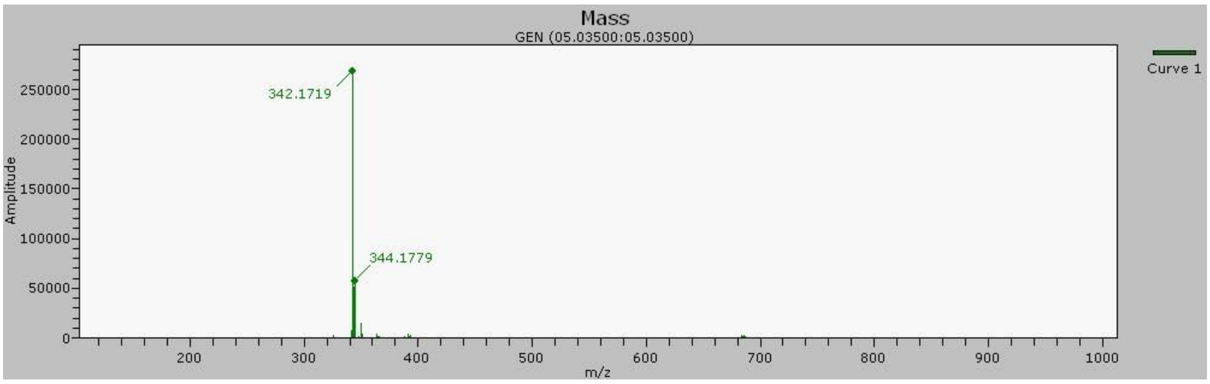

Mass
GEN (05.03500:05.03500)
Curve 1
342.1719
344.1779
Amplitude
250000
200000
150000
100000
50000
0
200
300
400
500
600
700
800
900
1000
m/z

**N, N-diethyl-2-(2-(4-(trifluoromethyl)benzyl)-1*H*-benzo[*d*]imidazol-1-yl)ethan-1-amine hydrochloride (11).**

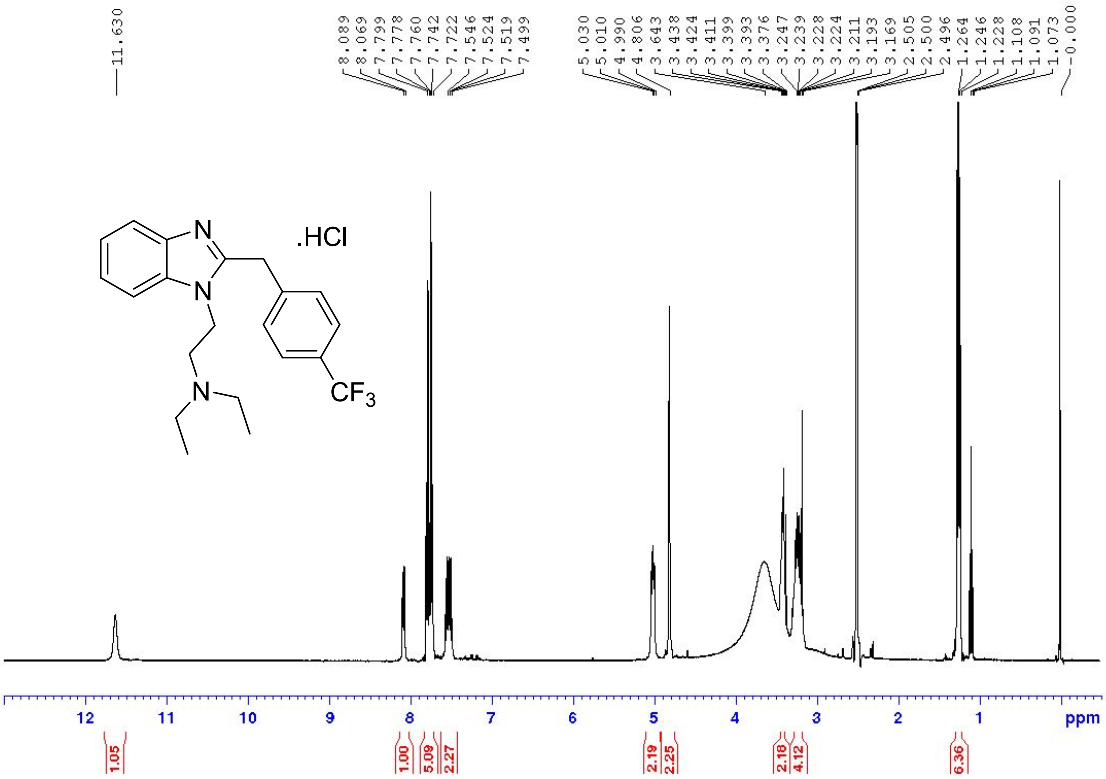


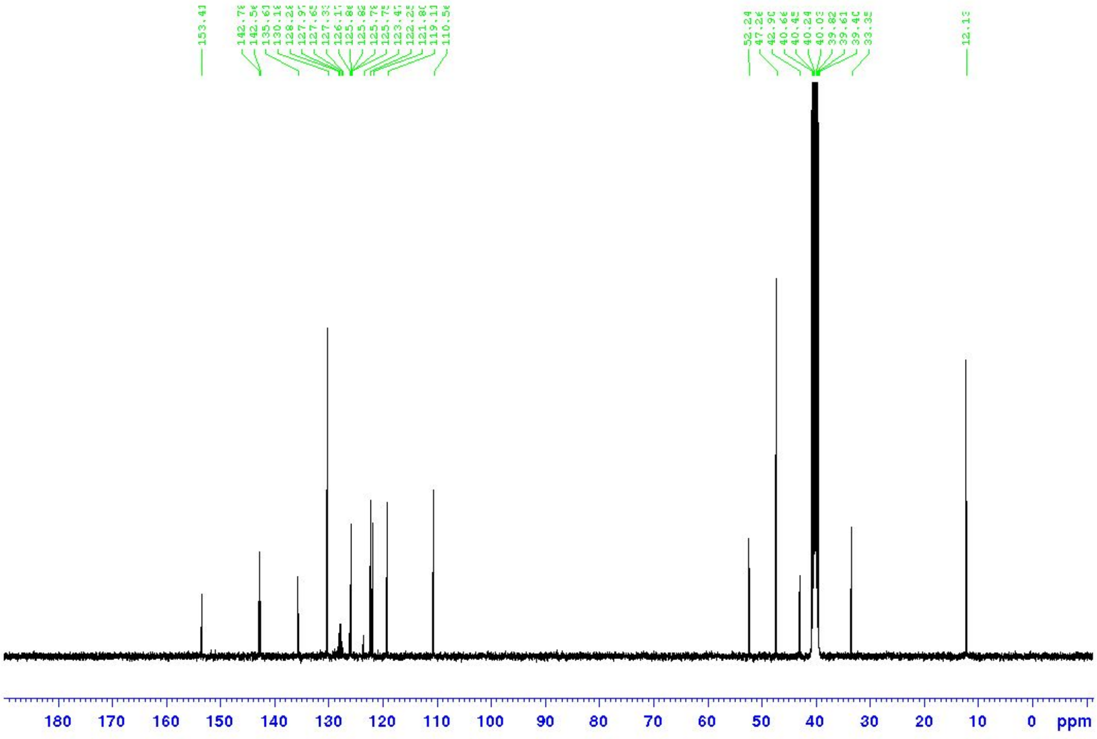

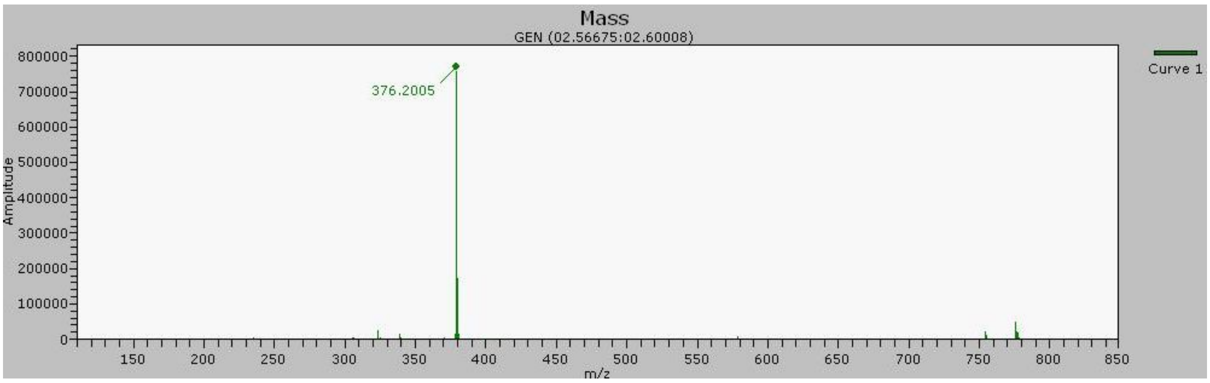


**4-((1-(2-(diethylamino)ethyl)-1*H*-benzo[*d*]imidazol-2-yl)methyl)benzonitrile hydrochloride (12).**

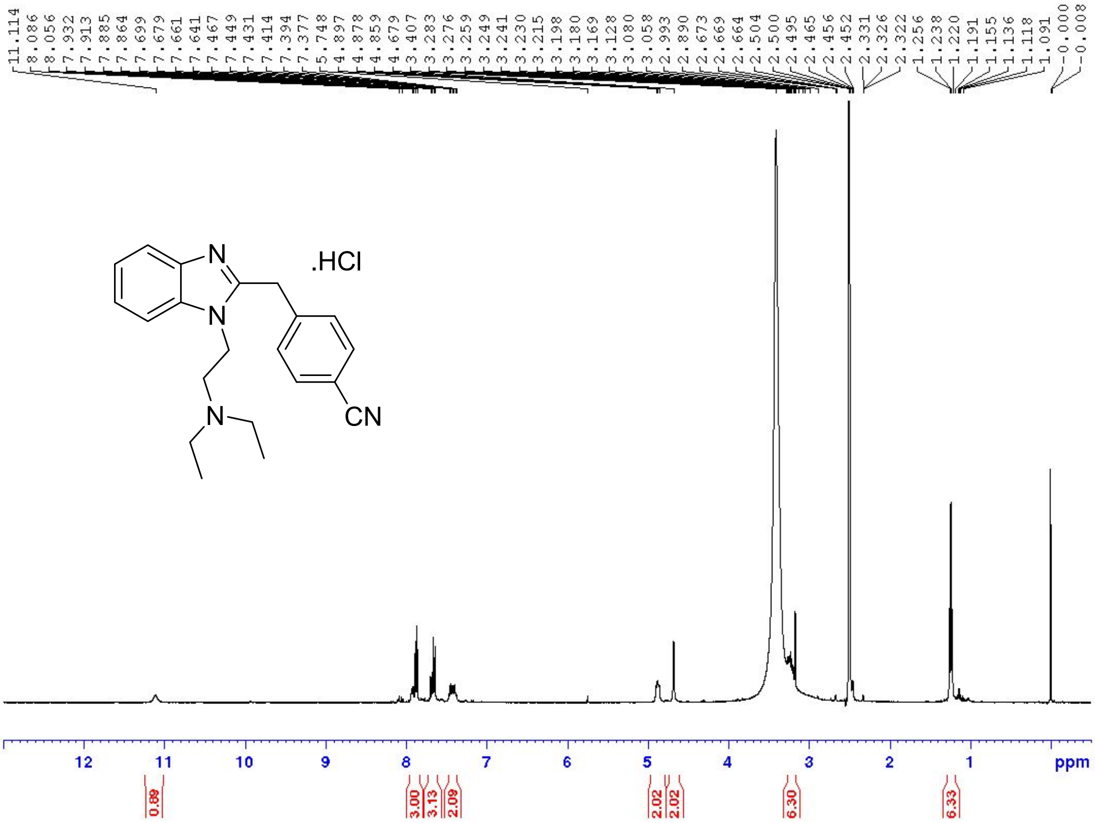

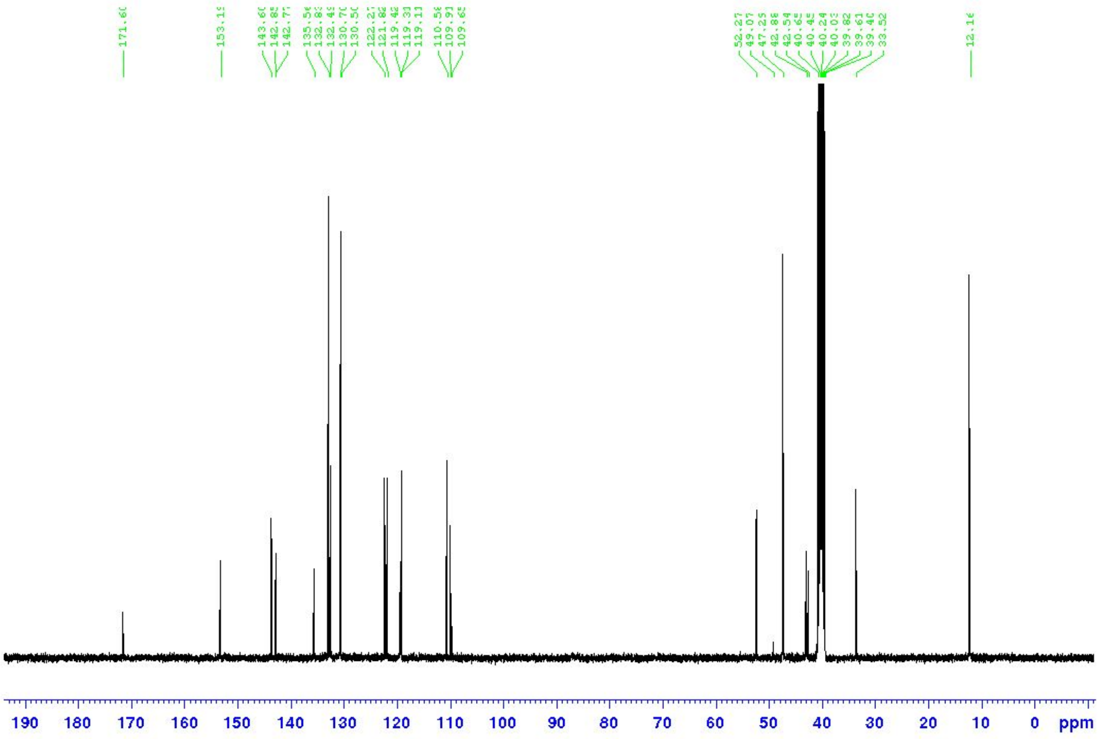
171.60
153.19
143.60
142.85
142.77
135.56
132.83
132.49
130.70
130.50
122.27
121.82
119.42
119.31
119.13
110.56
109.91
109.62
52.27
49.07
47.25
42.88
42.54
40.45
40.24
40.03
39.82
39.61
39.40
33.52
12.16
190 180 170 160 150 140 130 120 110 100 90 80 70 60 50 40 30 20 10 0 ppm

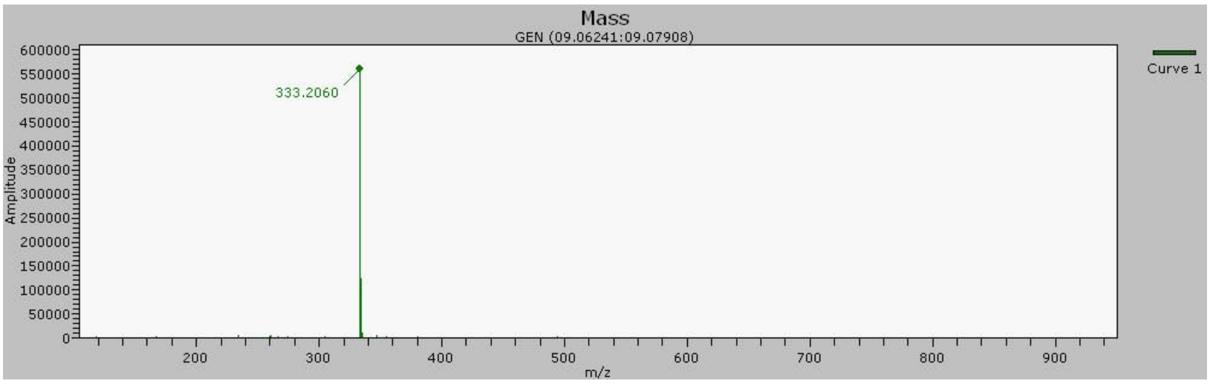
Mass
GEN (09.06241:09.07908)
Curve 1
333.2060
Amplitude
m/z

**N,N-diethyl-2-(2-(4-methylbenzyl)-1*H*-benzo[*d*]imidazol-1-yl)ethan-1-amine hydrochloride (13).**

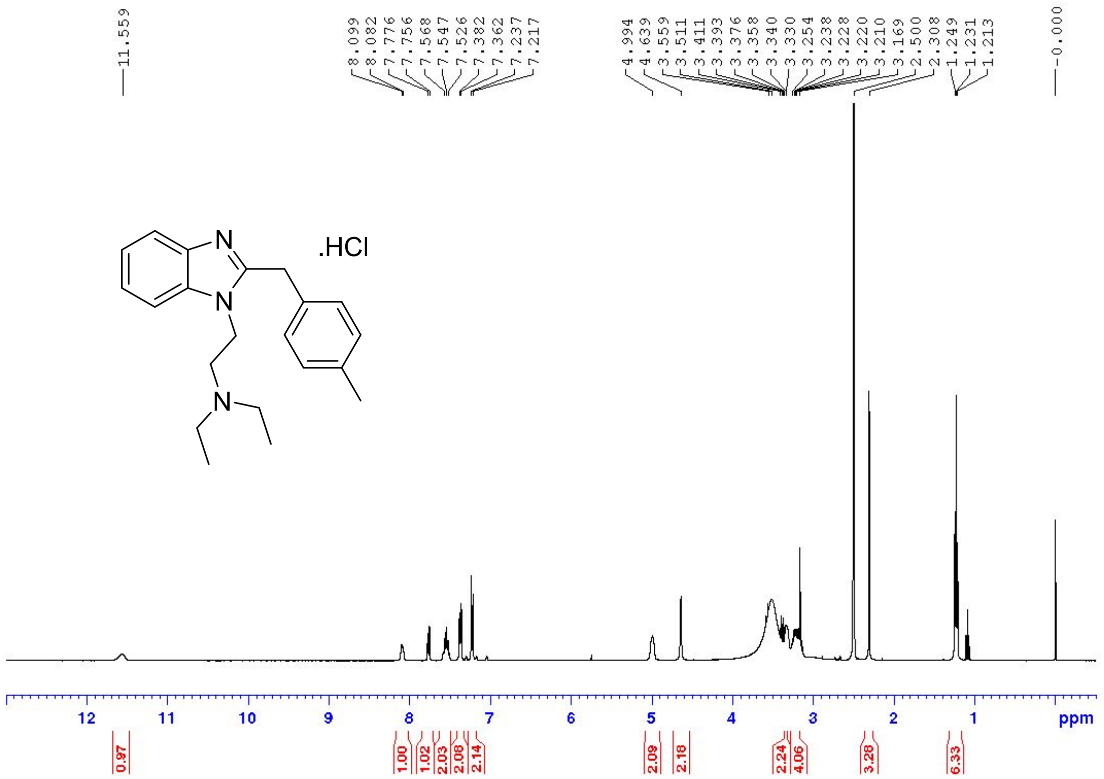


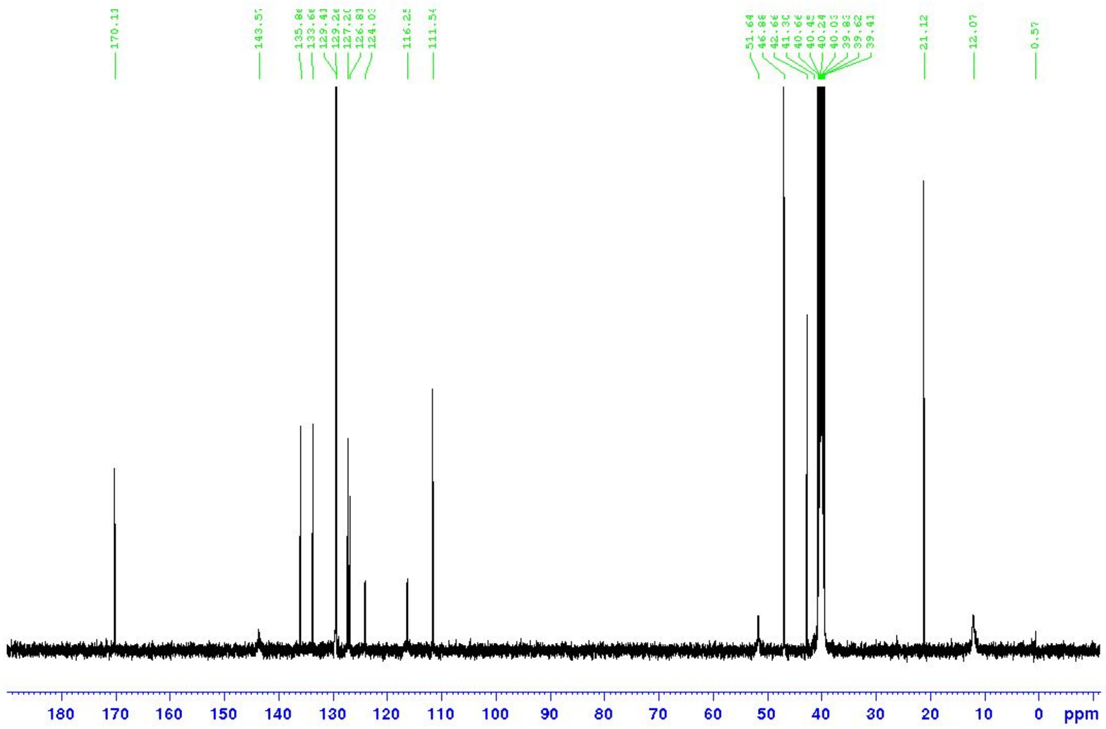

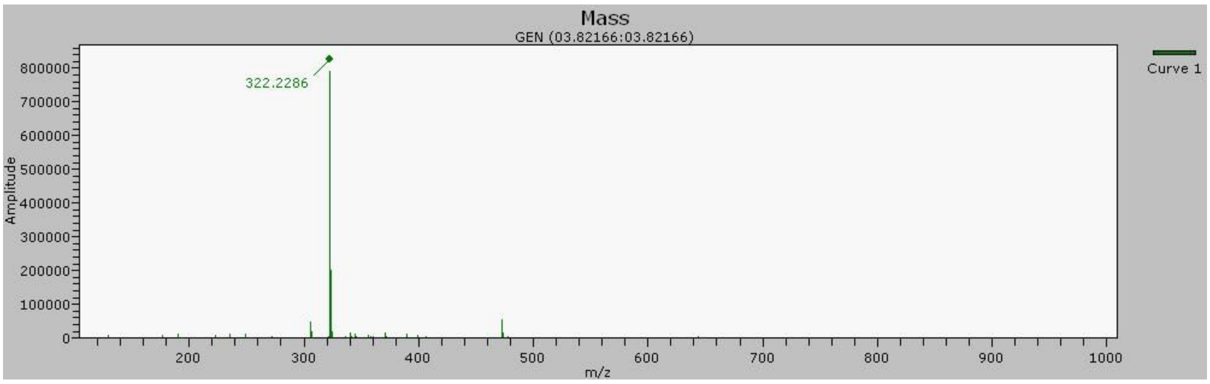


**N,N-diethyl-2-(2-(4-isopropylbenzyl)-1*H*-benzo[*d*]imidazol-1-yl)ethan-1-amine hydrochloride (14).**

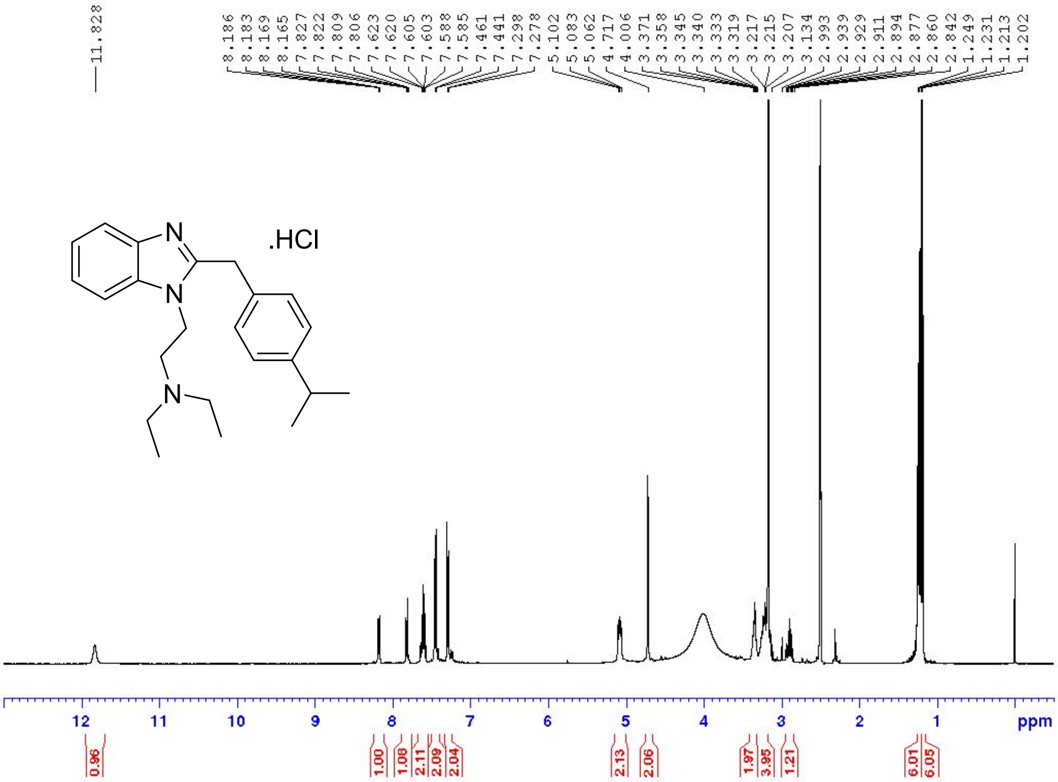

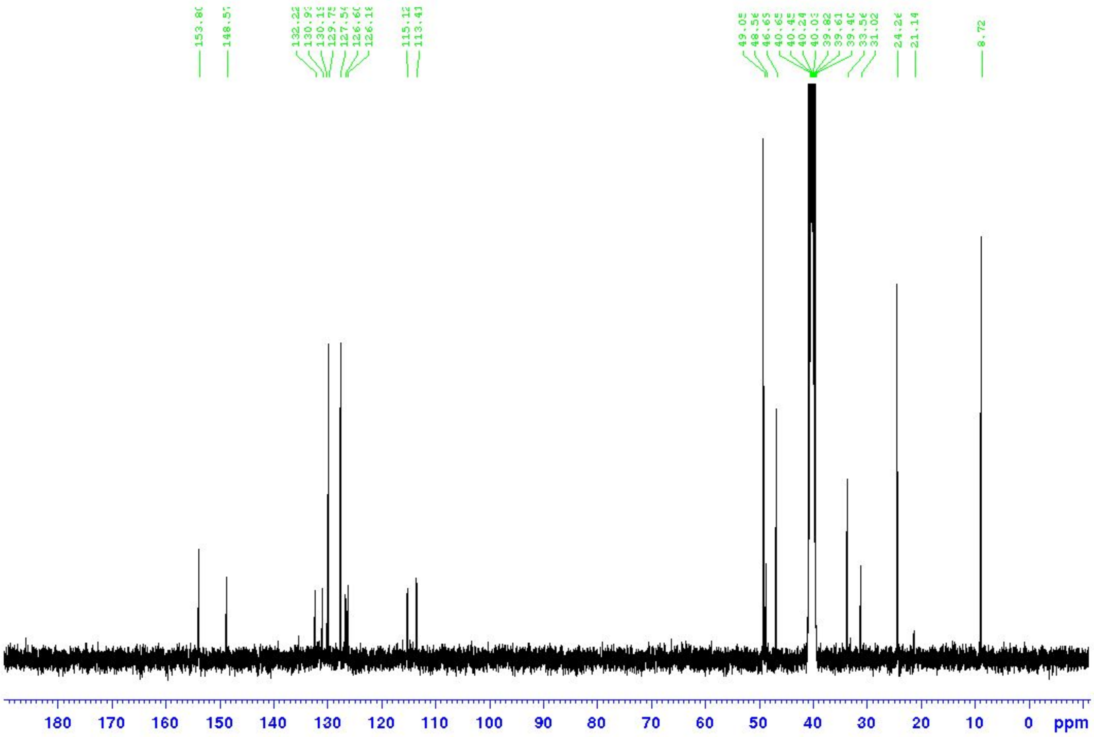

180 170 160 150 140 130 120 110 100 90 80 70 60 50 40 30 20 10 0 ppm


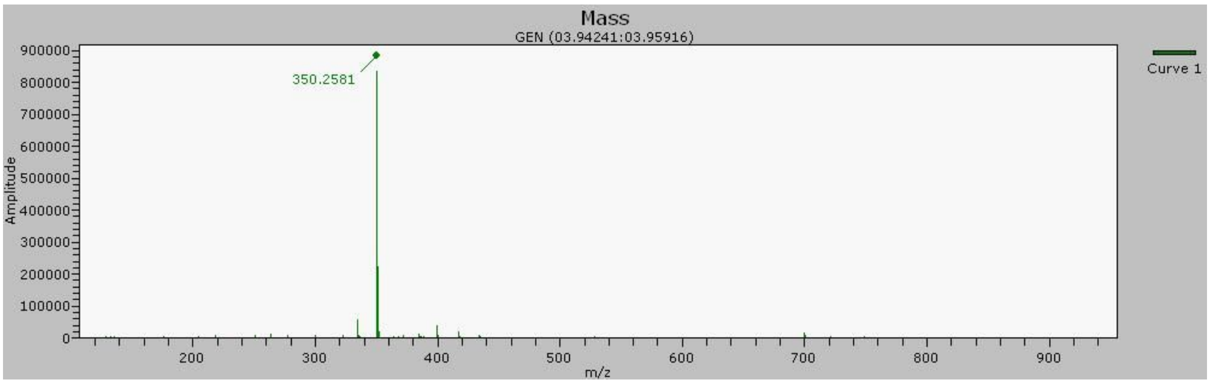

Mass
GEN (03.94241:03.95916)
350.2581
Curve 1
Amplitude
900000
800000
700000
600000
500000
400000
300000
200000
100000
0
200
300
400
500
600
700
800
900
m/z

**N,N-diethyl-2-(2-(4-ethylbenzyl)-1*H*-benzo[*d*]imidazol-1-yl)ethan-1-amine hydrochloride (15).**

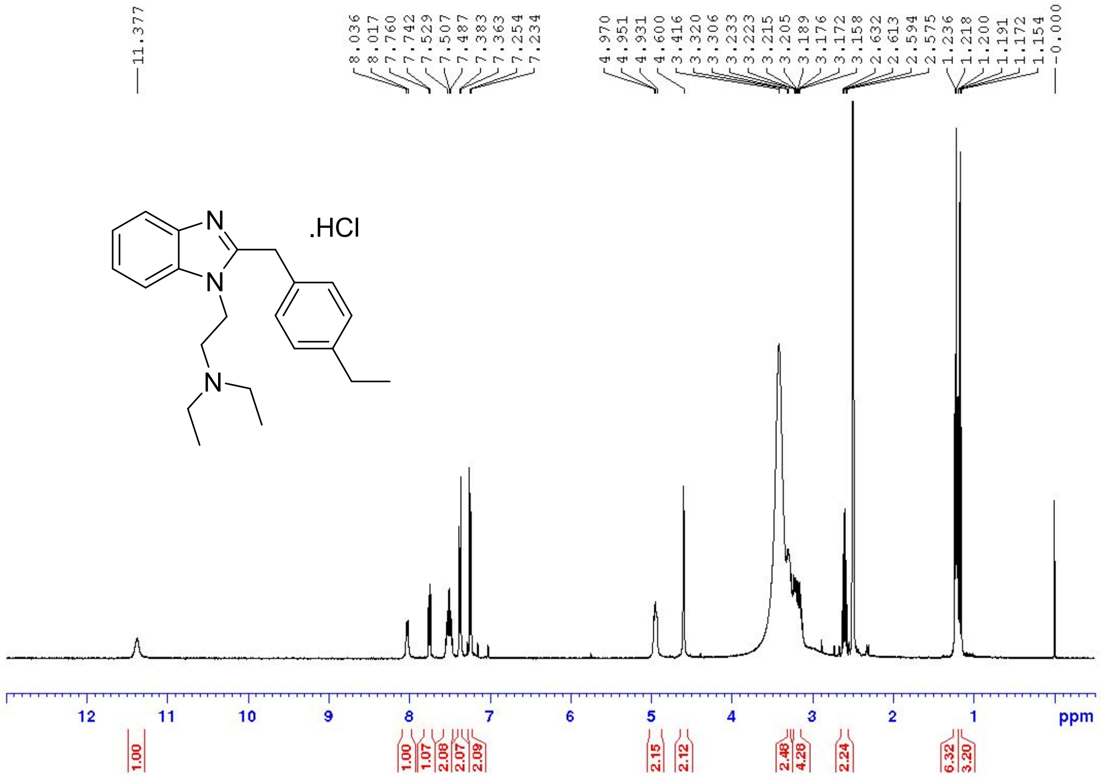

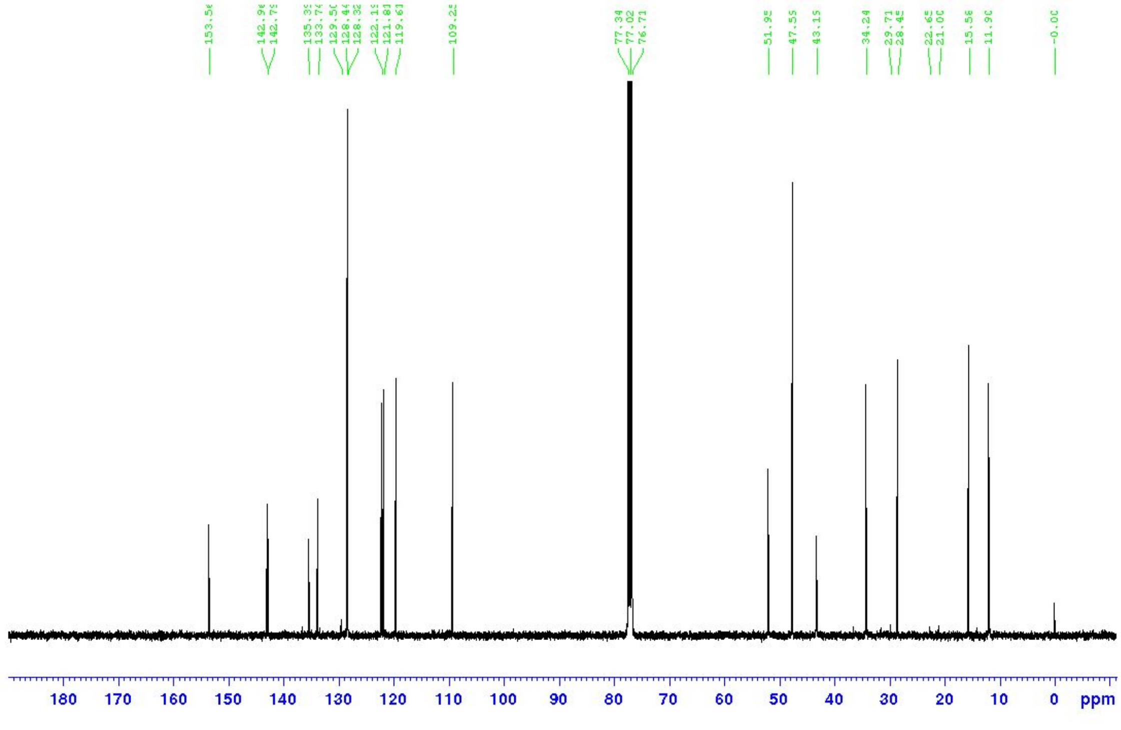

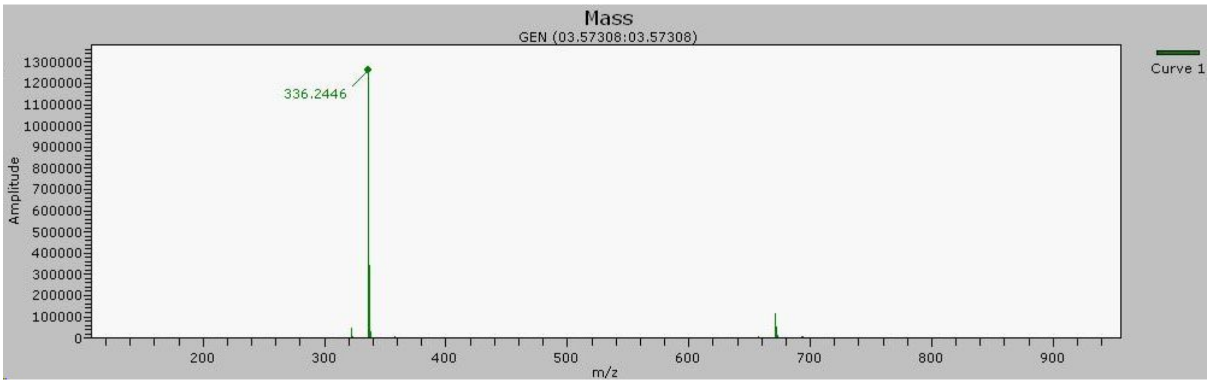


**2-(2-(4-ethoxybenzyl)-1H-benzo[d]imidazol-1-yl)-N,N-diethylethan-1-amine hydrochloride (16)**.

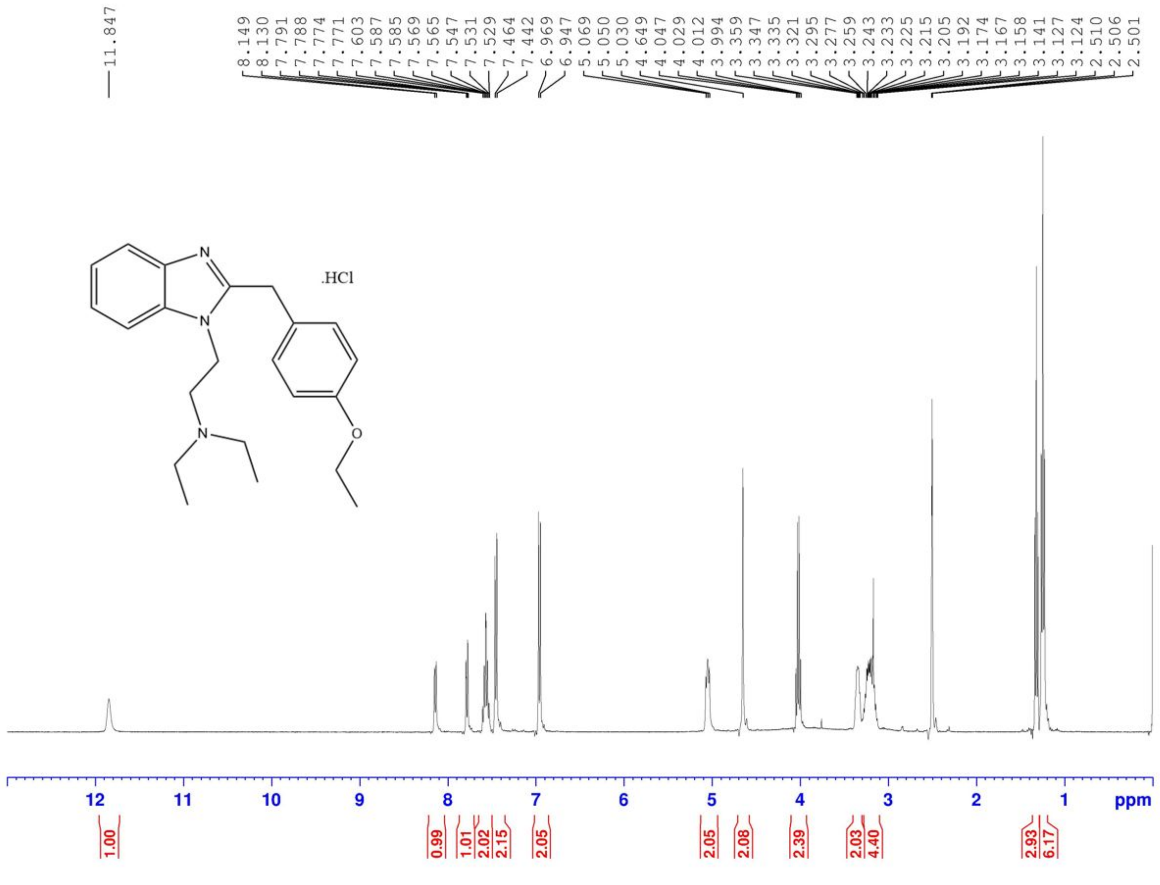

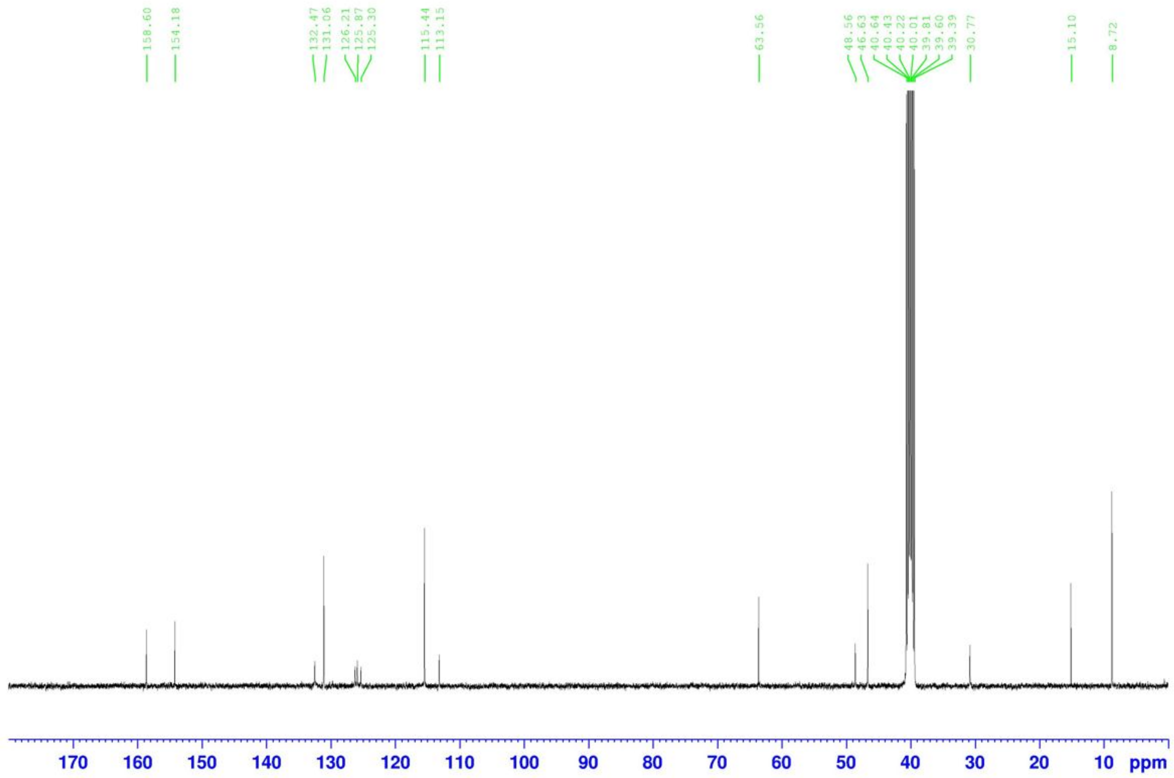

158.60
154.18
132.47
131.06
126.21
125.87
125.30
115.44
113.15
63.56
48.56
46.63
40.64
40.43
40.22
40.01
39.81
39.60
39.39
30.77
15.10
8.72
170
160
150
140
130
120
110
100
90
80
70
60
50
40
30
20
10
ppm


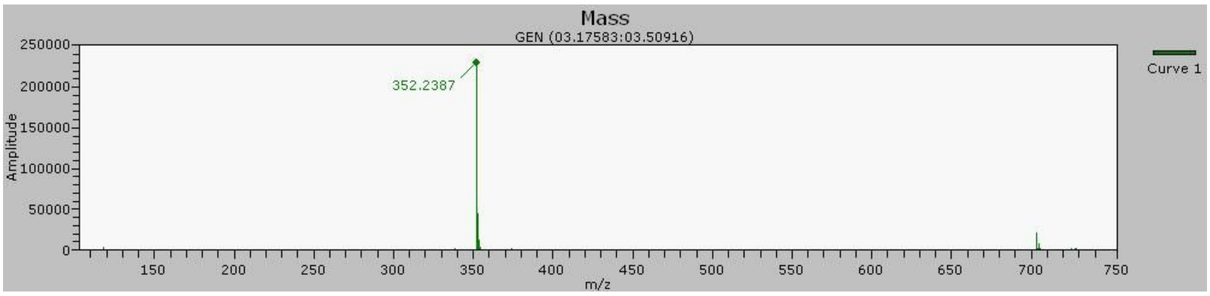

Mass
GEN (03.17583:03.50916)
Curve 1
352.2387
Amplitude
m/z

**2-(4-chlorobenzyl)-1-(2-(pyrrolidin-1-yl)ethyl)-1*H*-benzo[*d*]imidazole hydrochloride (17).**

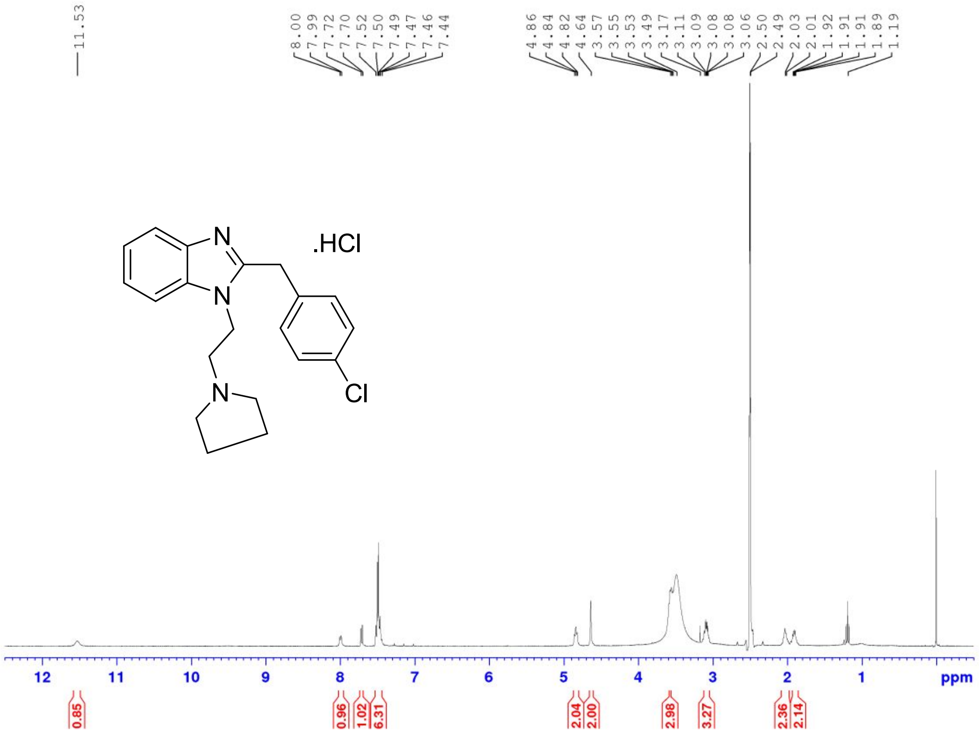

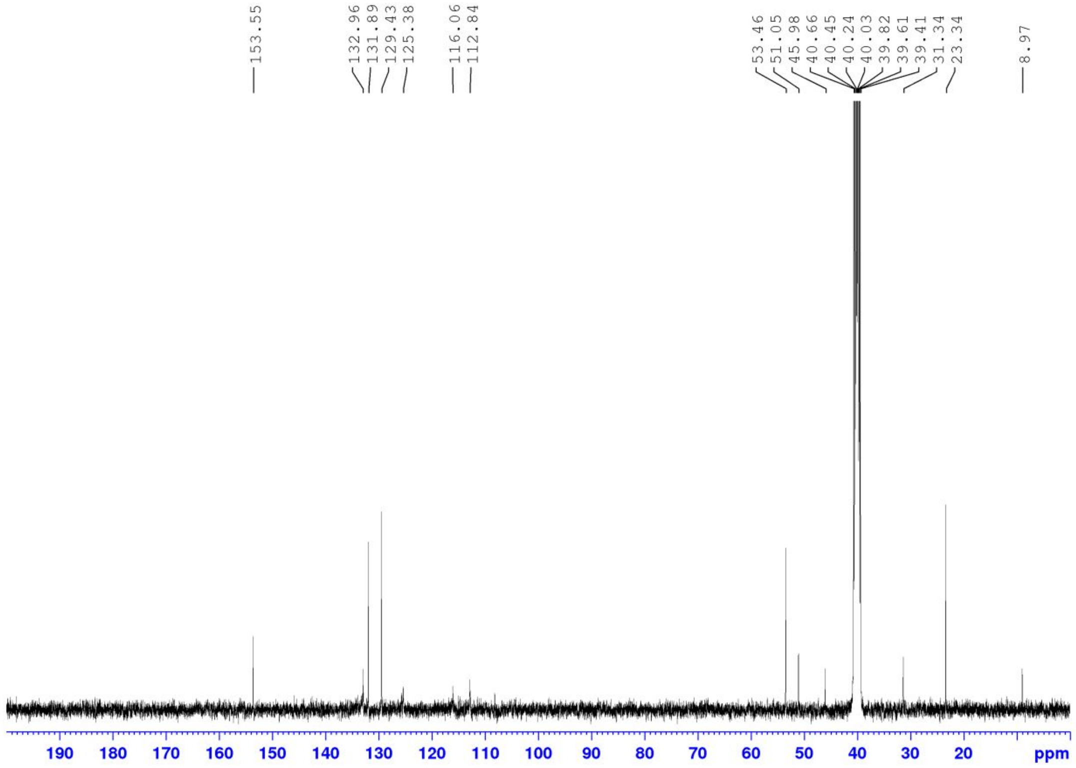

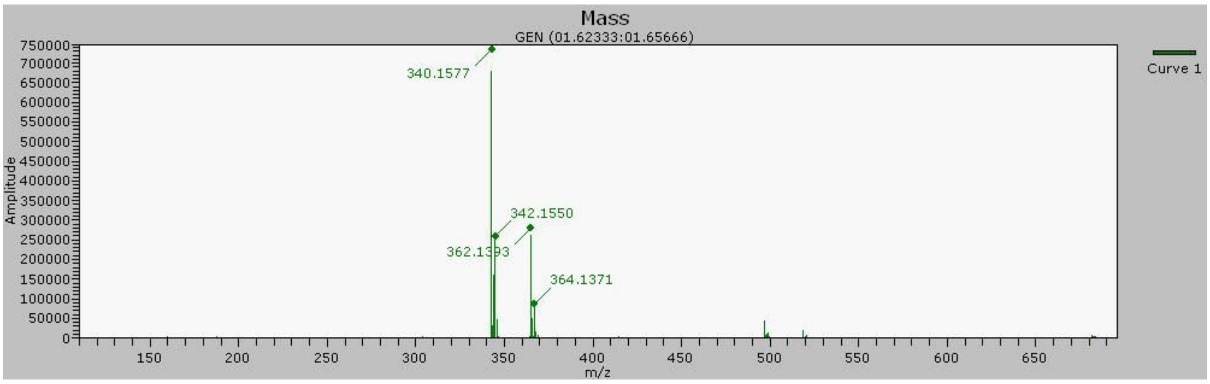


**1-(2-(pyrrolidin-1-yl)ethyl)-2-(4-(trifluoromethyl)benzyl)-1*H*-benzo[*d*]imidazole hydrochloride (18).**

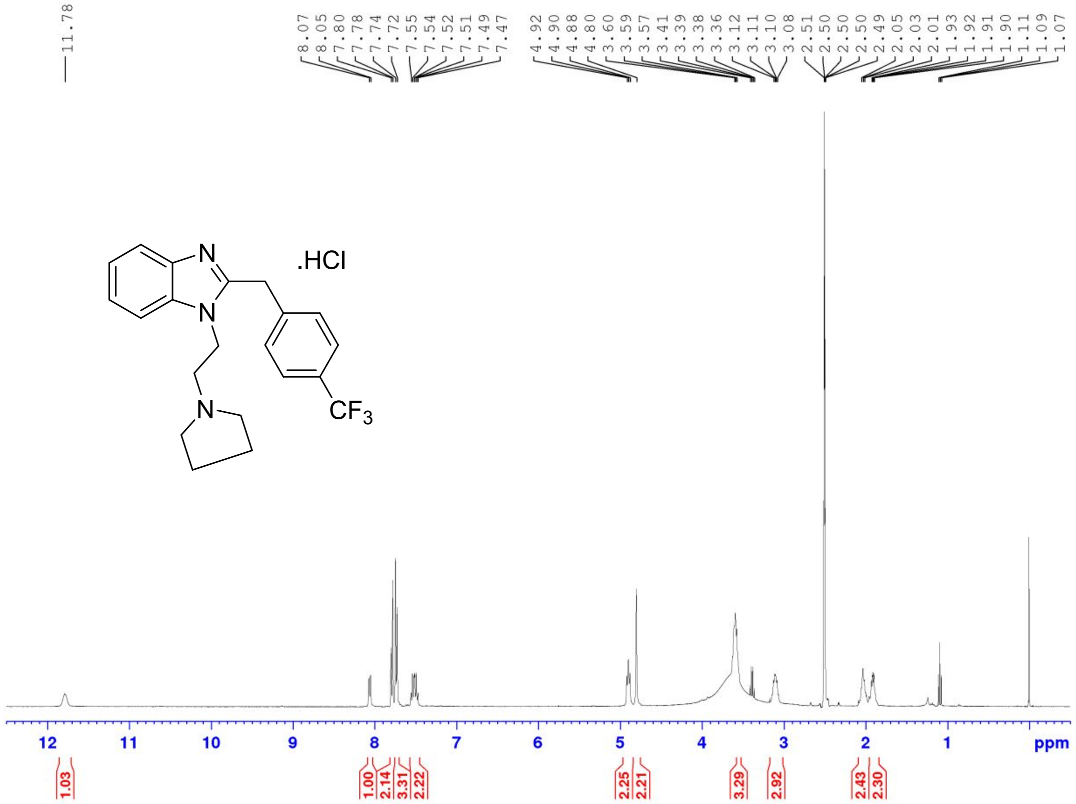

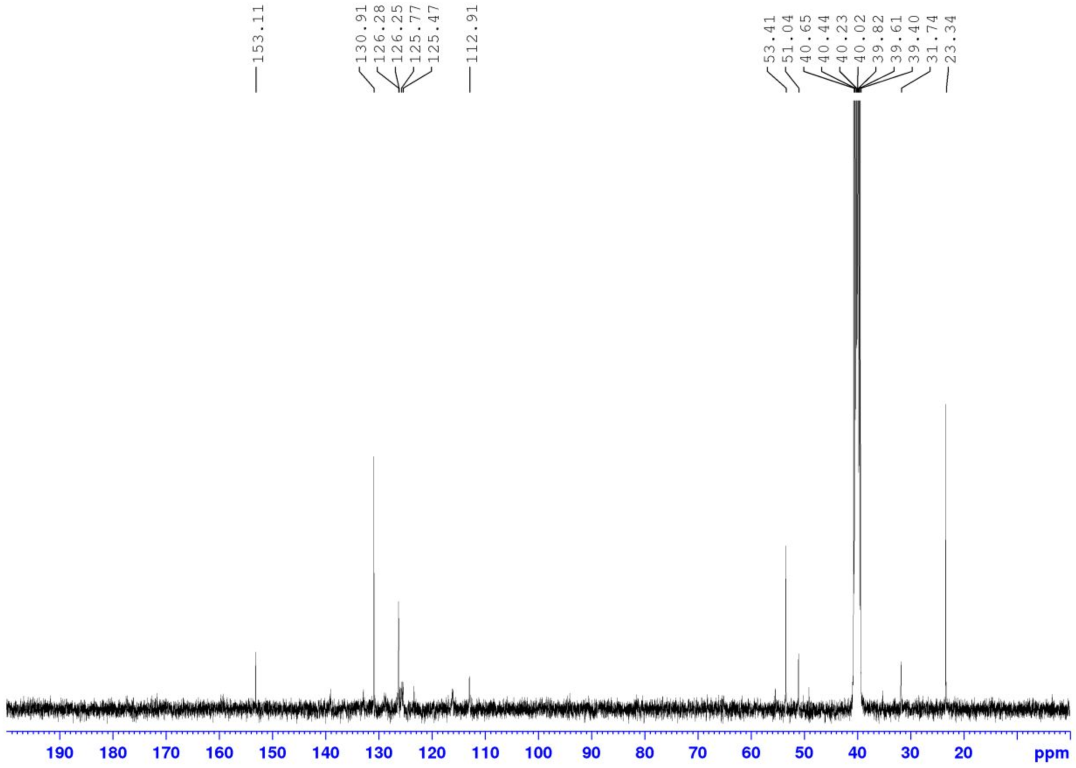
153.11
130.91
126.28
126.25
125.77
125.47
112.91
53.41
51.04
40.65
40.44
40.23
40.02
39.82
39.61
39.40
31.74
23.34
190 180 170 160 150 140 130 120 110 100 90 80 70 60 50 40 30 20 ppm

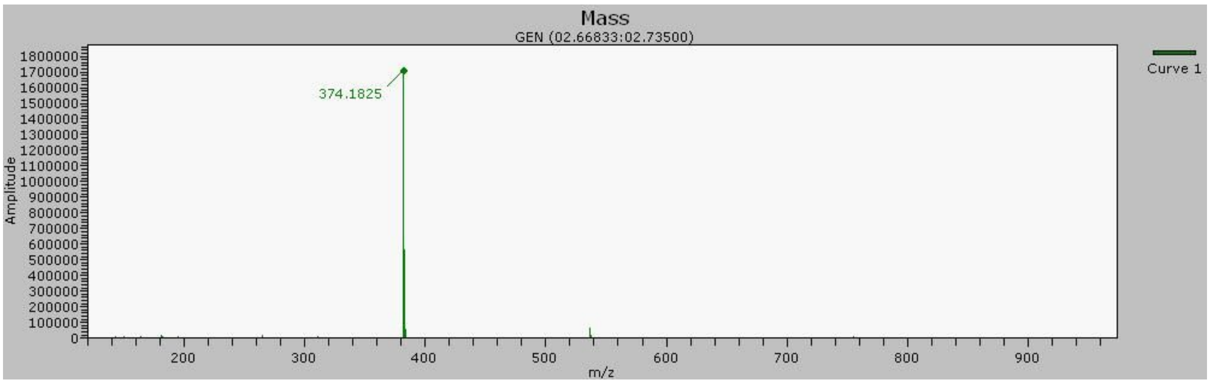
Mass
GEN (02.66833:02.73500)
Curve 1
374.1825
Amplitude
m/z

**2-(4-fluorobenzyl)-1-(2-(pyrrolidin-1-yl)ethyl)-1*H*-benzo[*d*]imidazole hydrochloride (19).**

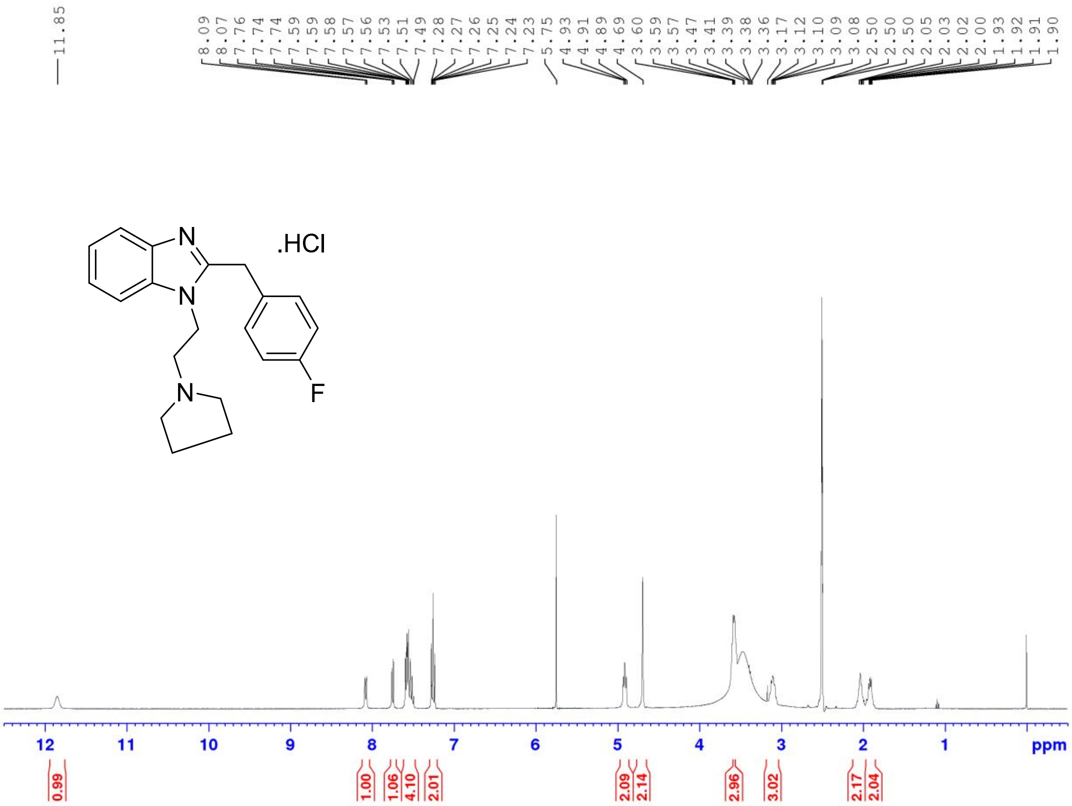


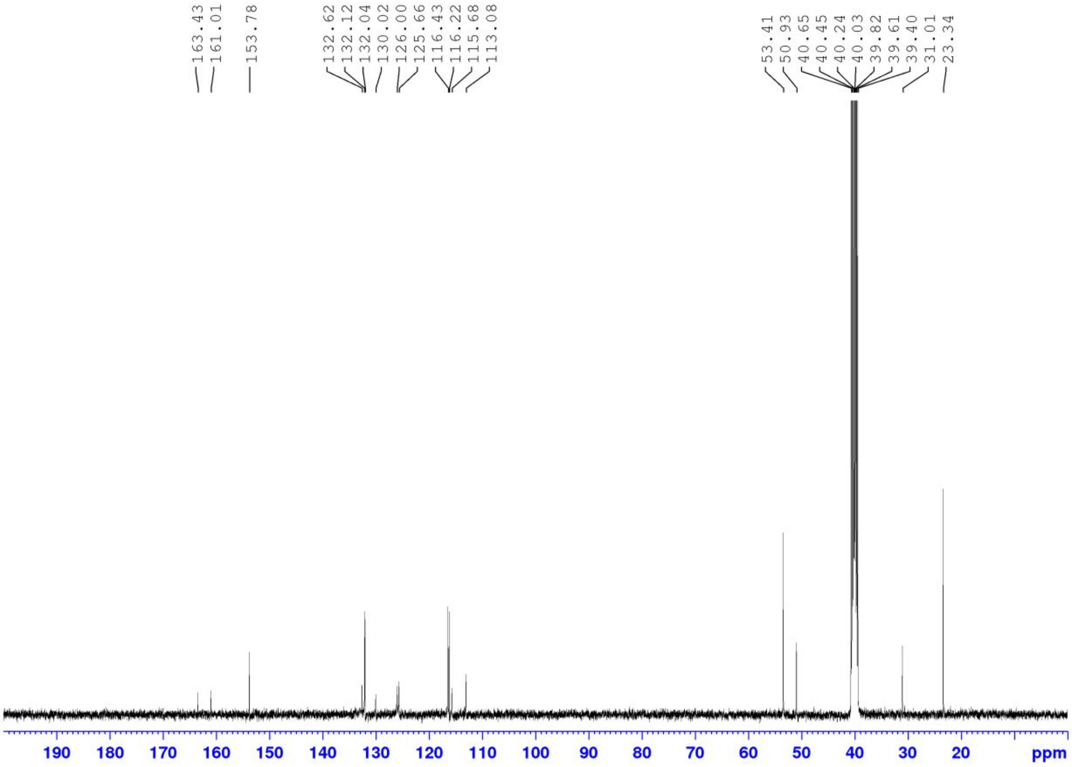

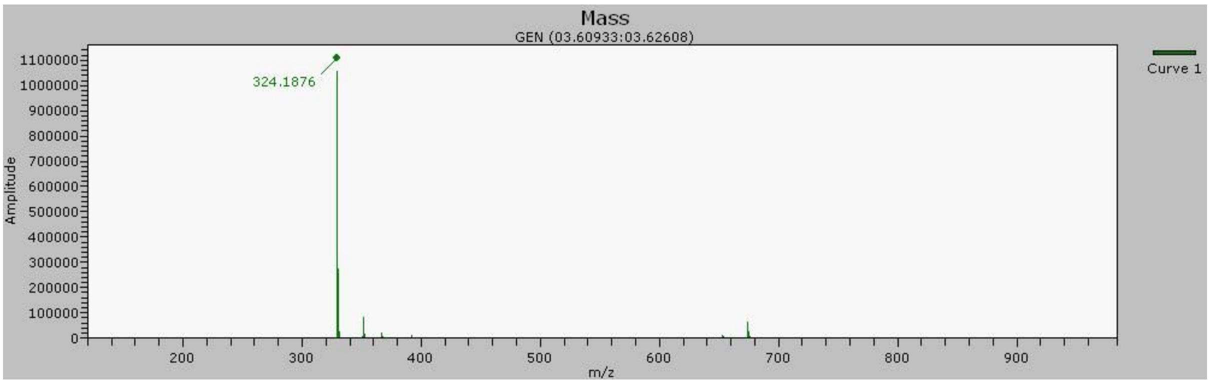


**2-(4-ethylbenzyl)-1-(2-(pyrrolidin-1-yl)ethyl)-1*H*-benzo[*d*]imidazole hydrochloride (20).**

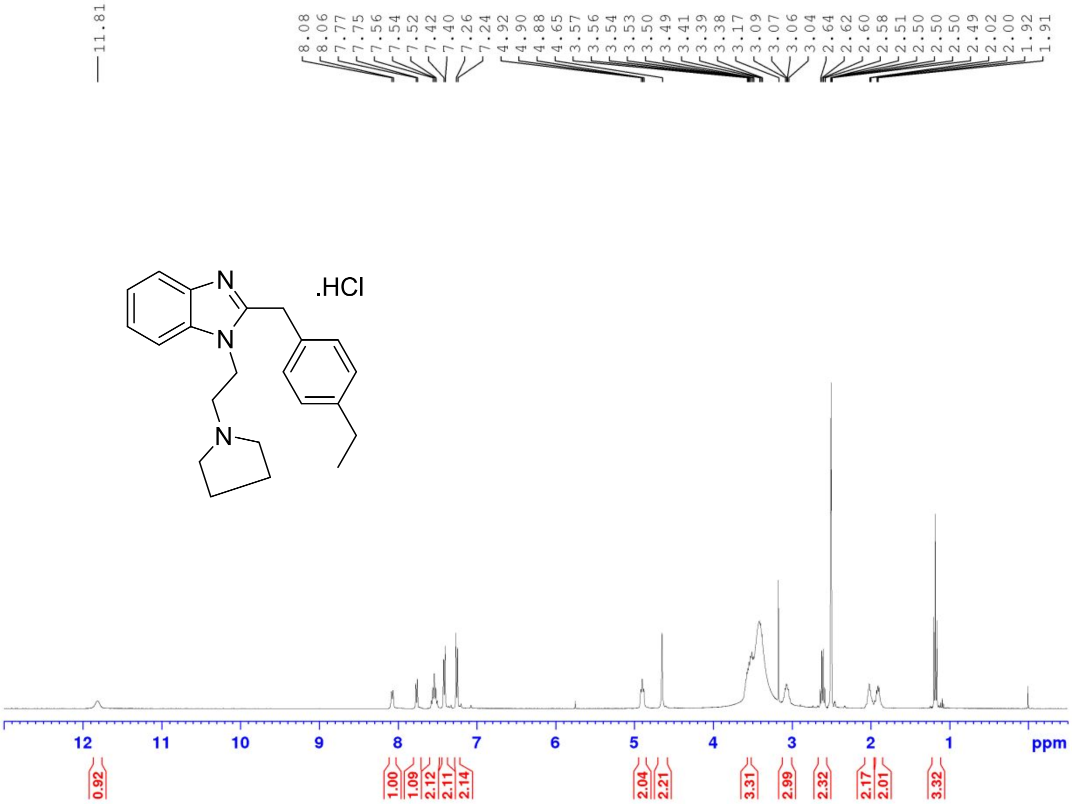

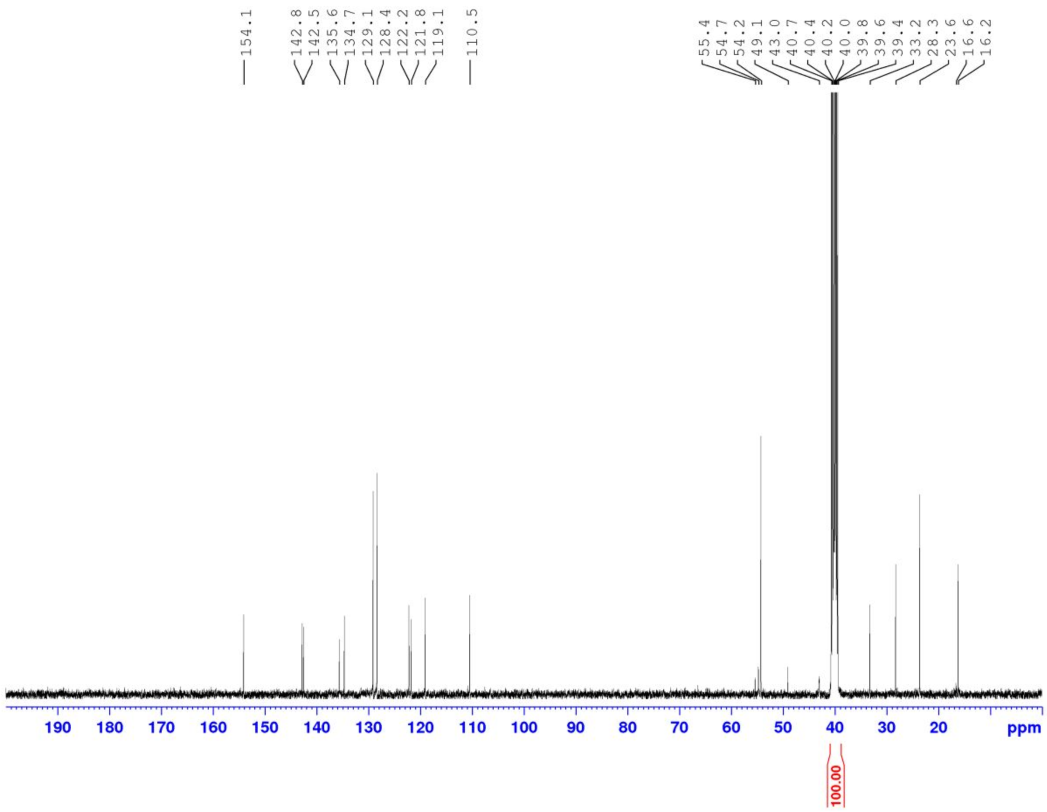
154.1
142.8
142.5
135.6
134.7
129.1
128.4
122.2
121.8
119.1
110.5
55.4
54.7
54.2
49.1
43.0
40.7
40.4
40.2
40.0
39.8
39.6
39.4
33.2
28.3
23.6
16.6
16.2
190 180 170 160 150 140 130 120 110 100 90 80 70 60 50 40 30 20 ppm
100.00

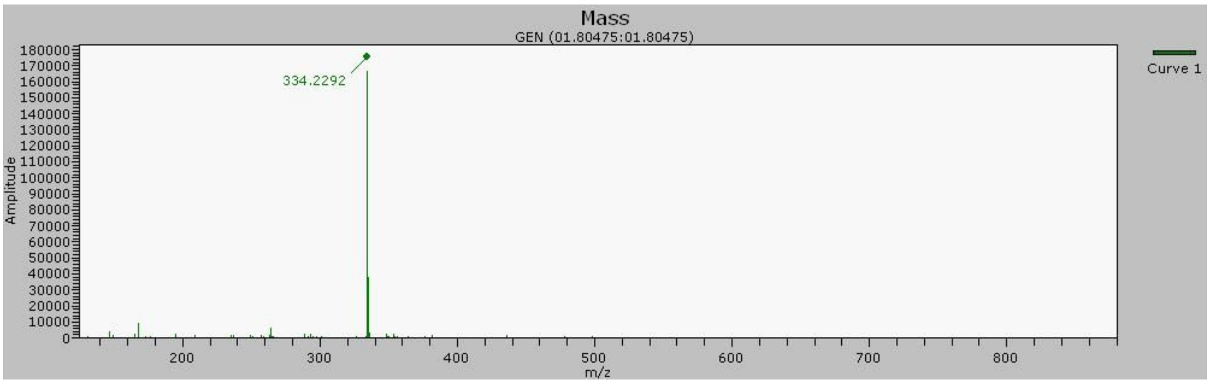
Mass
GEN (01.80475:01.80475)
Curve 1
334.2292
Amplitude
180000 170000 160000 150000 140000 130000 120000 110000 100000 90000 80000 70000 60000 50000 40000 30000 20000 10000 0
200 300 400 500 600 700 800
m/z

**2-(4-methylbenzyl)-1-(2-(pyrrolidin-1-yl)ethyl)-1*H*-benzo[*d*]imidazole hydrochloride (21).**

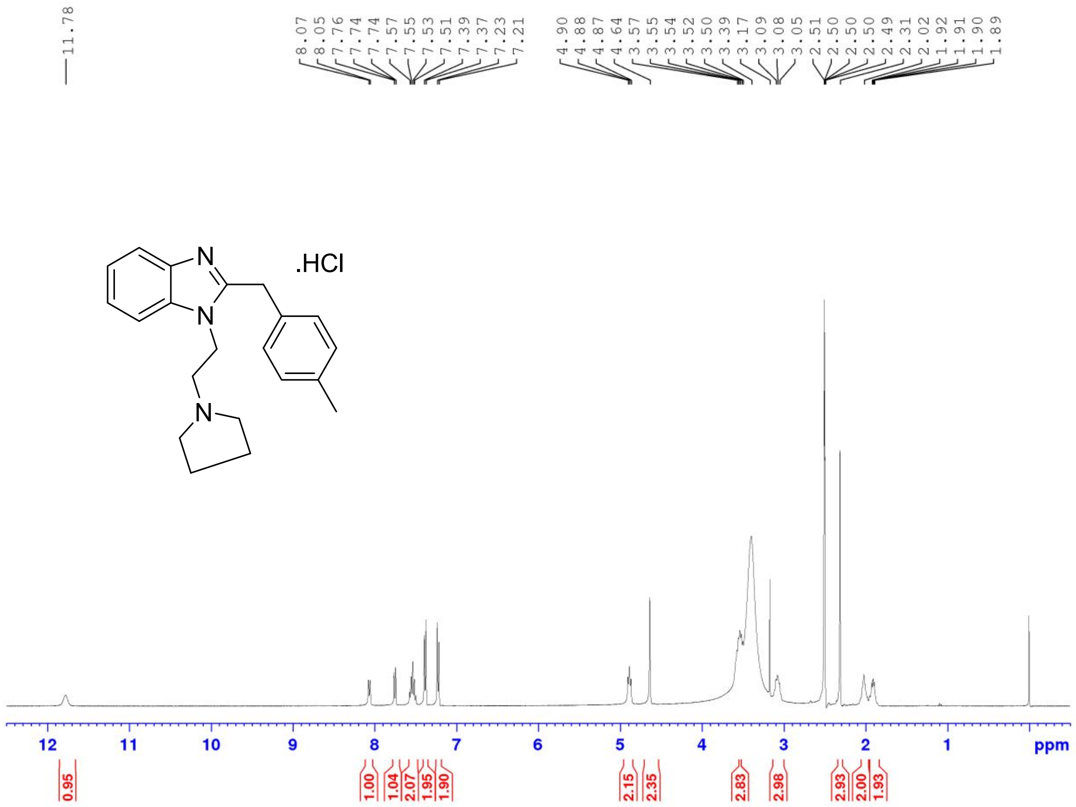


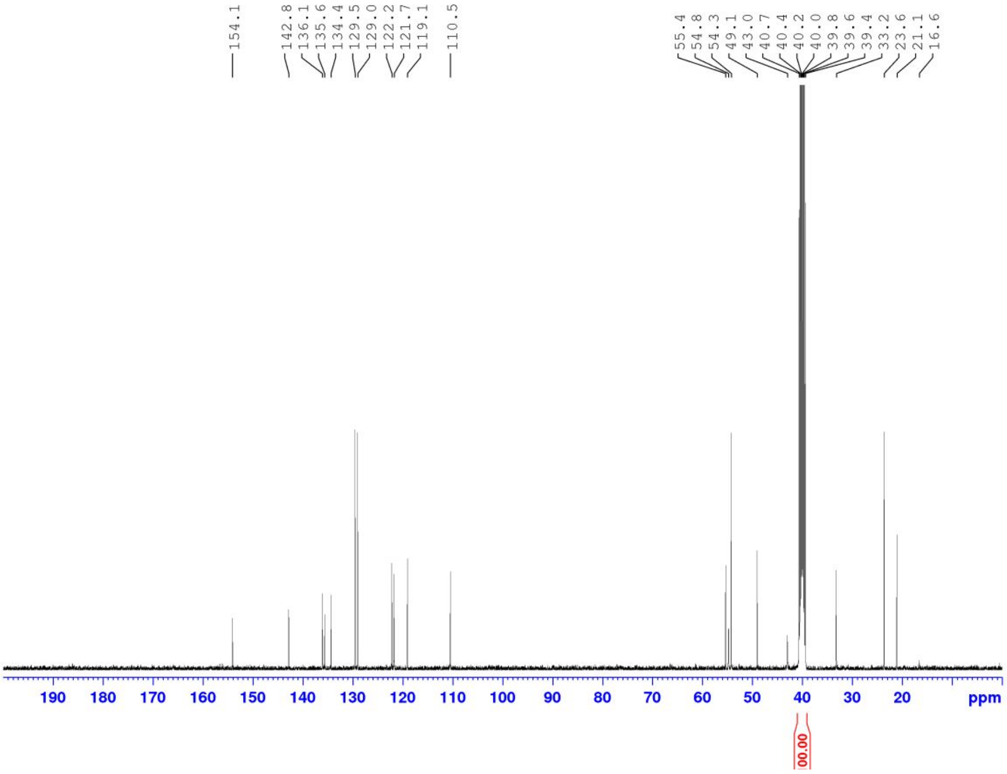

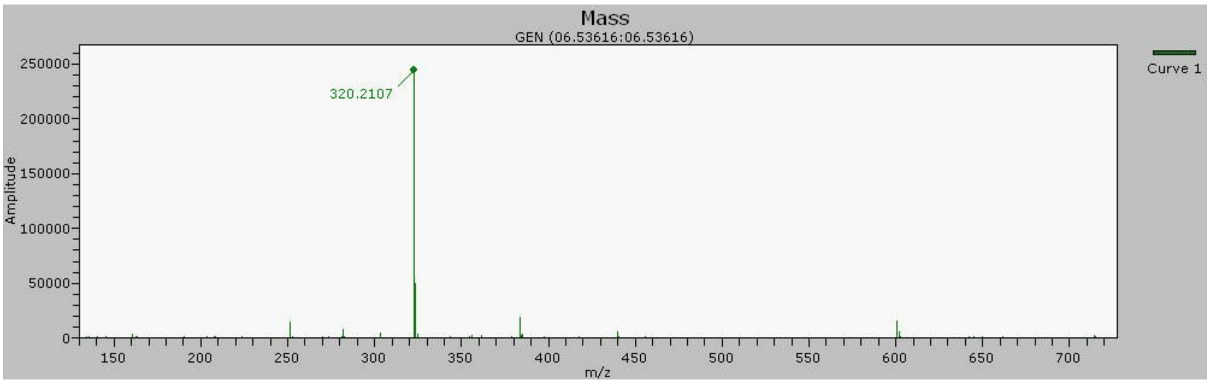


**2-(4-isopropylbenzyl)-1-(2-(pyrrolidin-1-yl)ethyl)-1*H*-benzo[*d*]imidazole hydrochloride (22).**

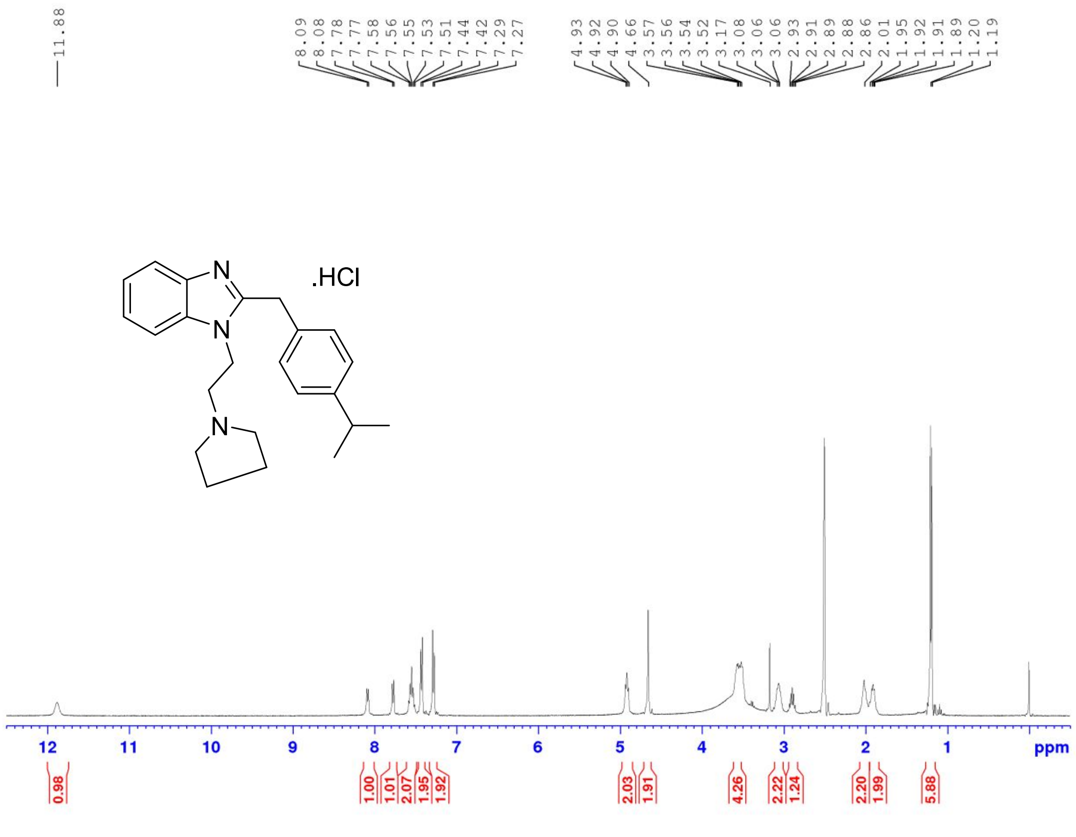

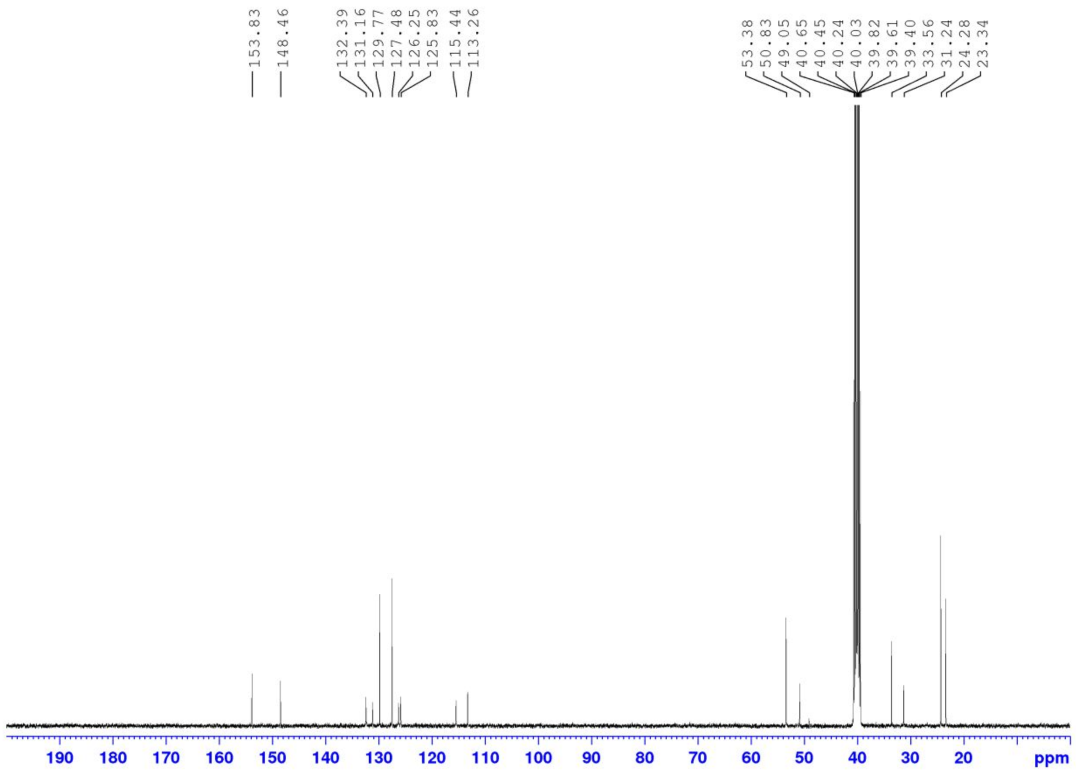
153.83
148.46
132.39
131.16
129.77
127.48
126.25
125.83
115.44
113.26
53.38
50.83
49.05
40.65
40.45
40.24
40.03
39.82
39.61
39.40
33.56
31.24
24.28
23.34
190
180
170
160
150
140
130
120
110
100
90
80
70
60
50
40
30
20
ppm

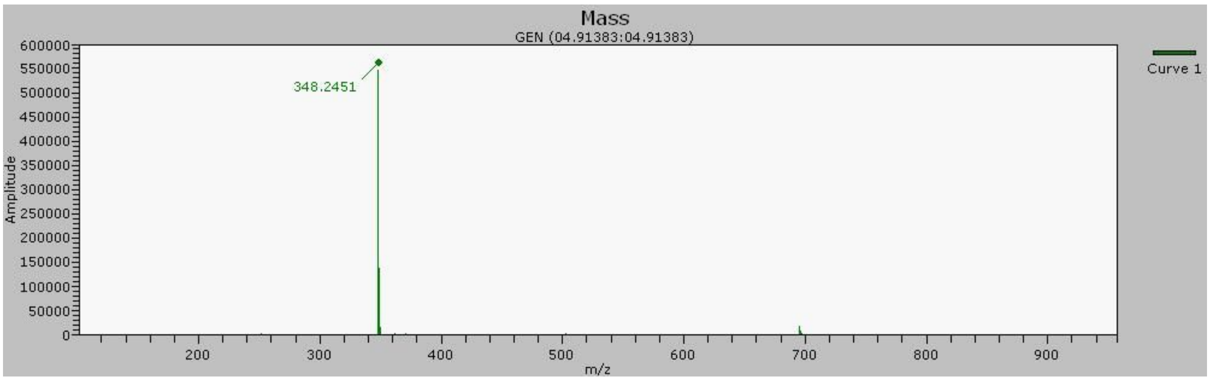
Mass
GEN (04.91383:04.91383)
348.2451
Curve 1
Amplitude
m/z

**4-((1-(2-(pyrrolidin-1-yl)ethyl)-1*H*-benzo[*d*]imidazol-2-yl)methyl)benzonitrile hydrochloride (23).**

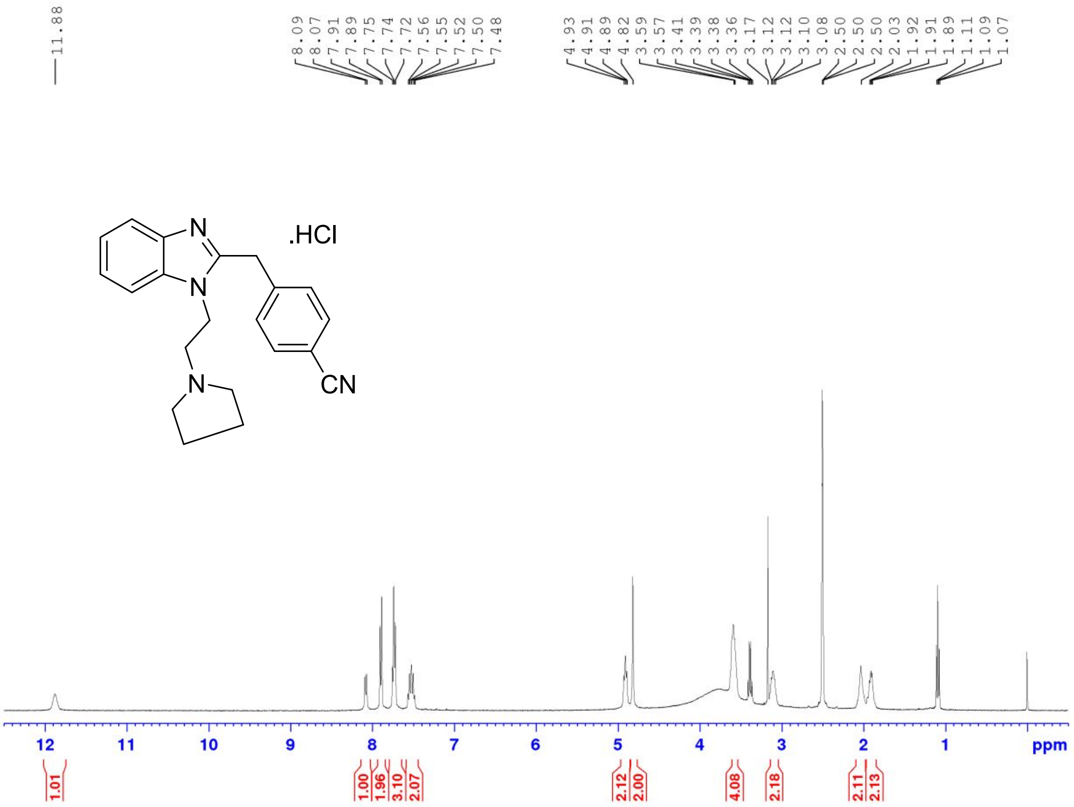


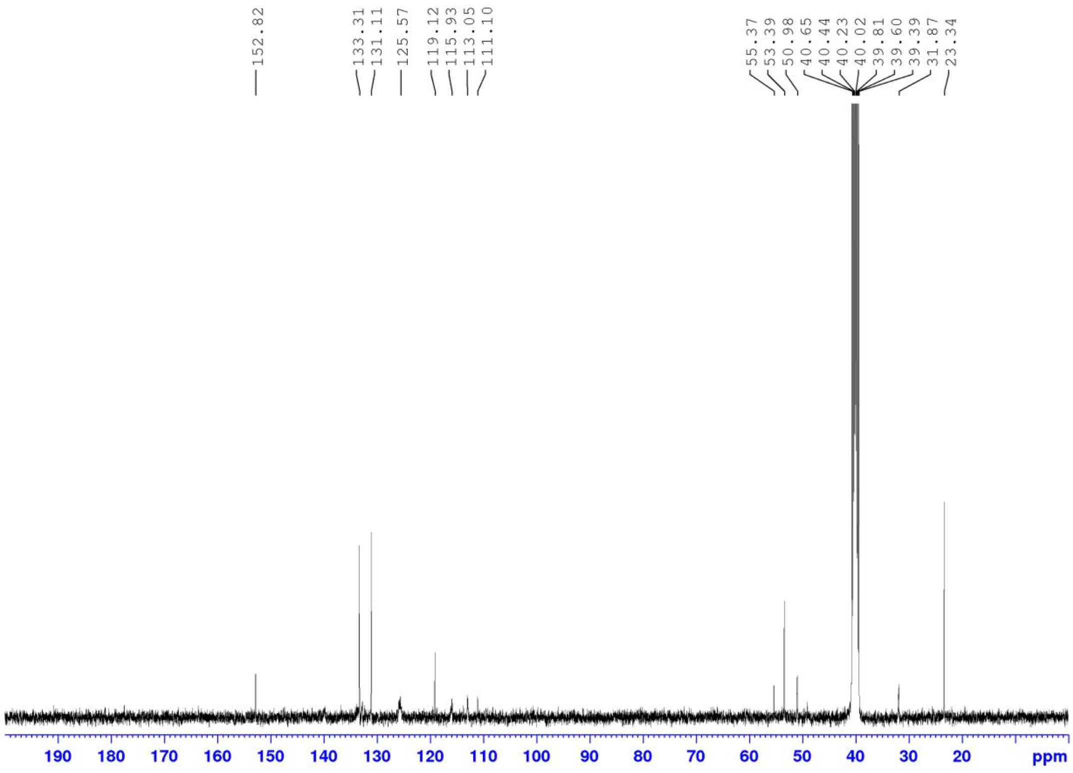

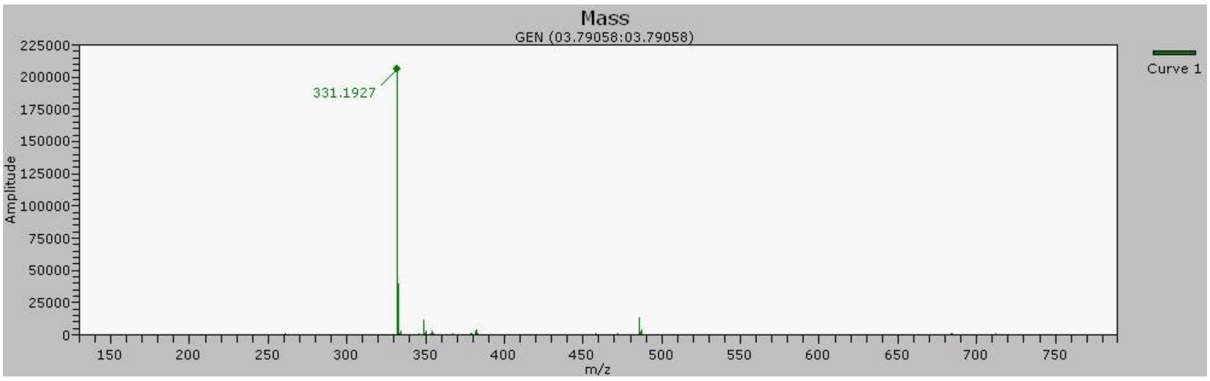


**2-(4-ethoxybenzyl)-1-(2-(pyrrolidin-1-yl)ethyl)-1H-benzo[d]imidazole hydrochloride (24).**

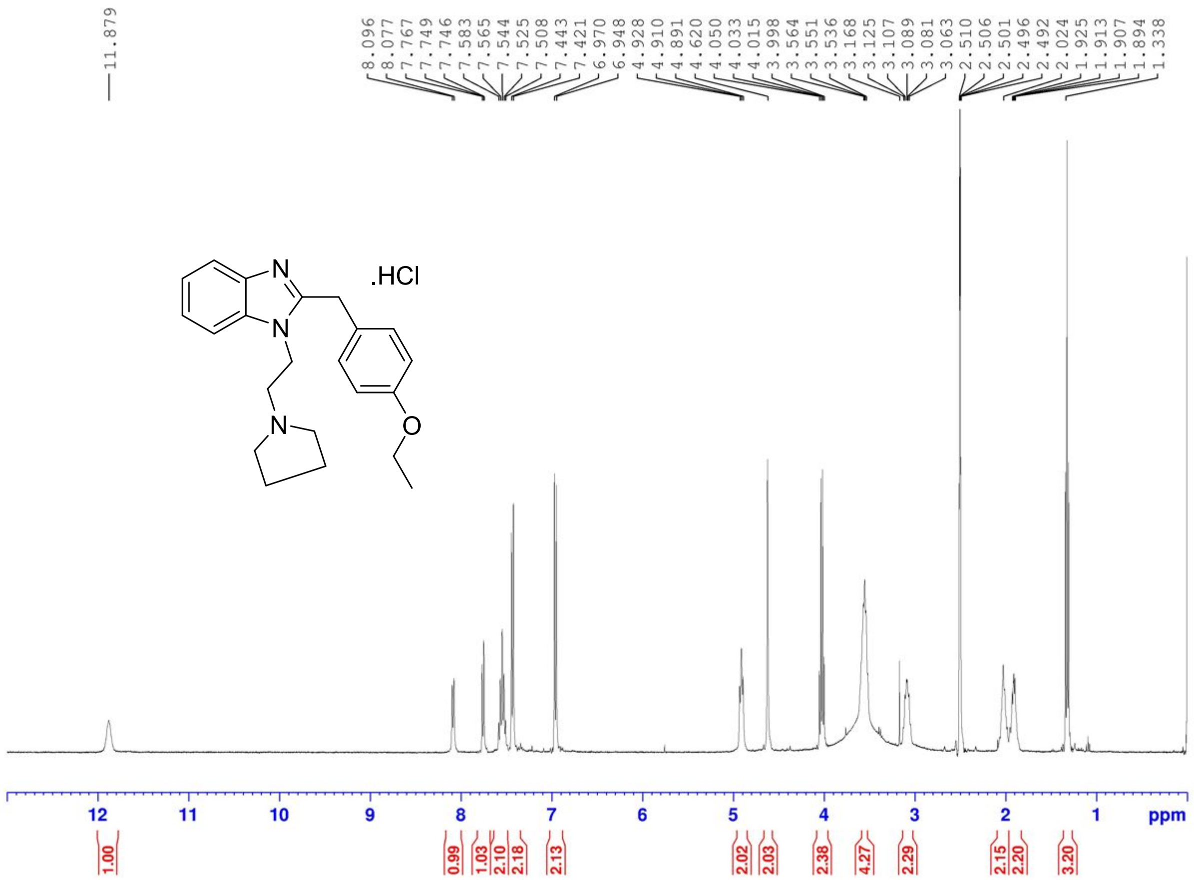

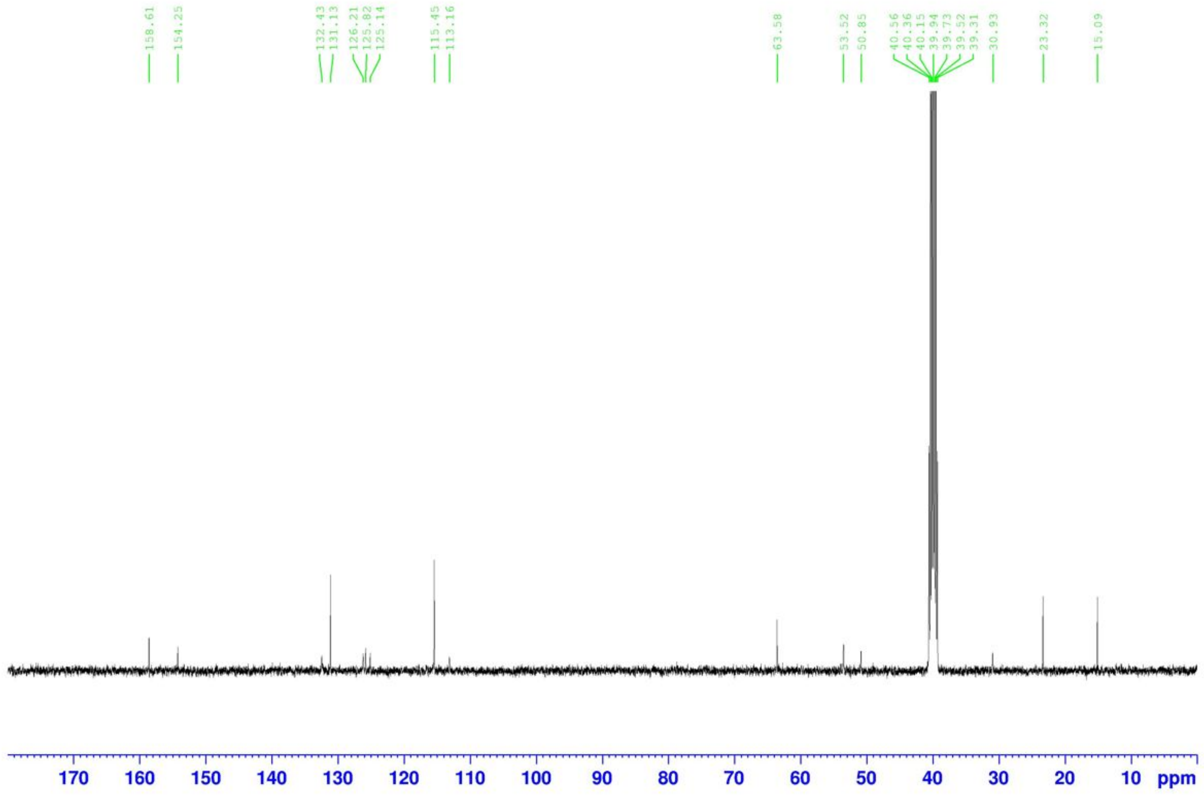
158.61
154.25
132.43
131.13
126.21
125.82
125.14
115.45
113.16
63.58
53.52
50.85
40.56
40.36
40.15
39.94
39.73
39.52
39.31
30.93
23.32
15.09
170 160 150 140 130 120 110 100 90 80 70 60 50 40 30 20 10 ppm

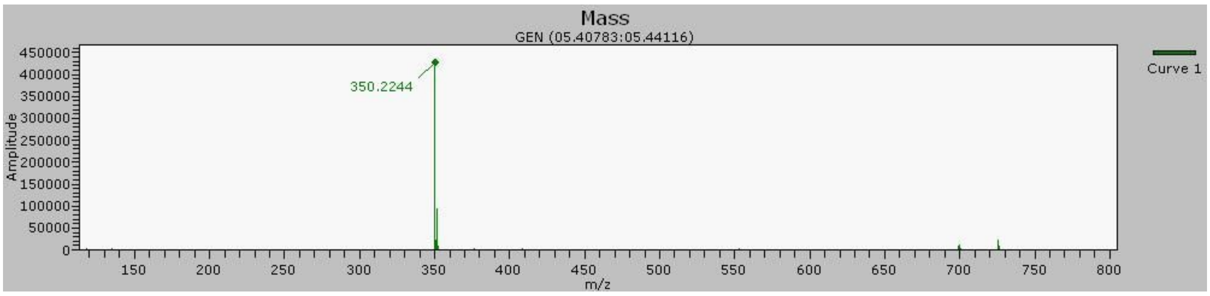
Mass
GEN (05.40783:05.44116)
Curve 1
350.2244
Amplitude
m/z

**2-(4-chlorobenzyl)-1-(2-(piperidin-1-yl)ethyl)-1*H*-benzo[*d*]imidazole hydrochloride (25).**

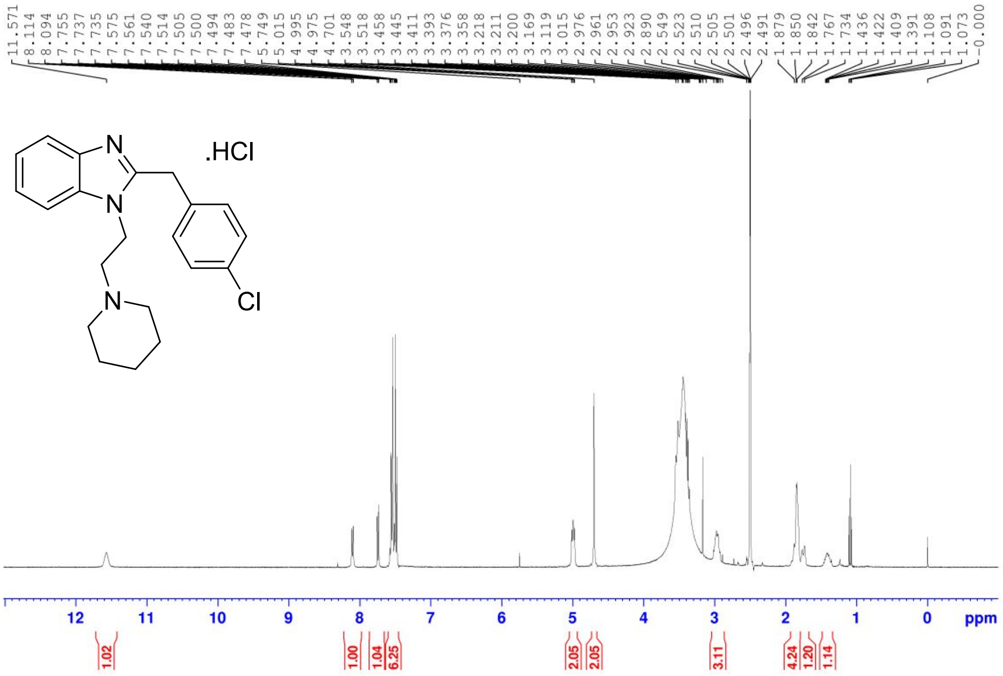


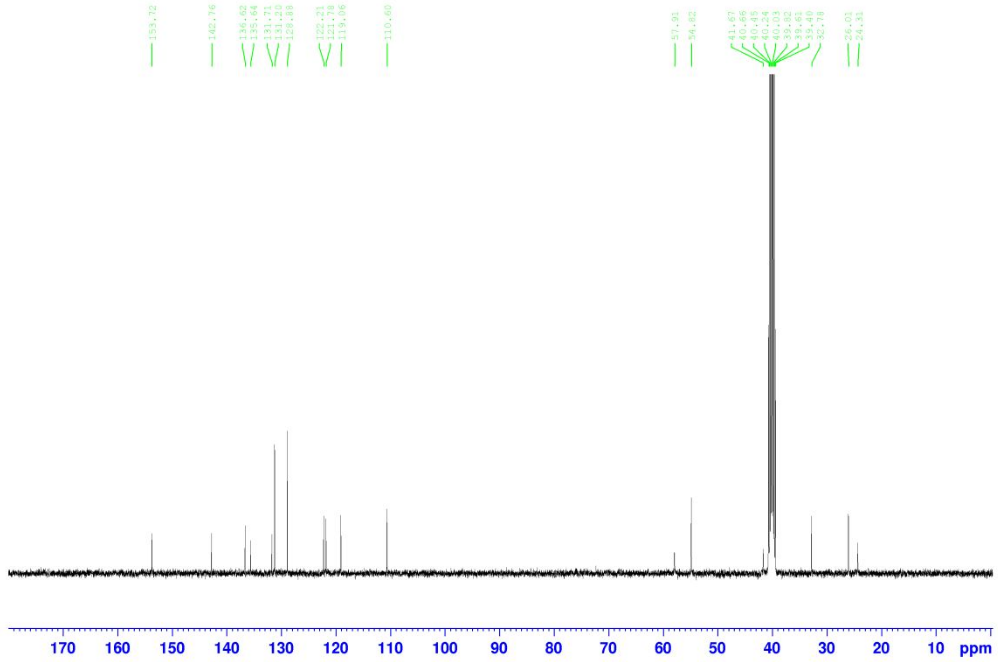

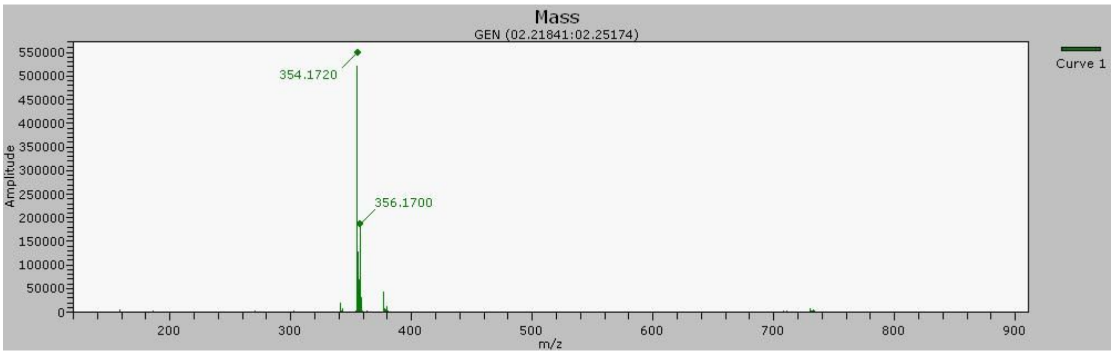


**1-(2-(piperidin-1-yl)ethyl)-2-(4-(trifluoromethyl)benzyl)-1*H*-benzo[*d*]imidazole hydrochloride (26)**.

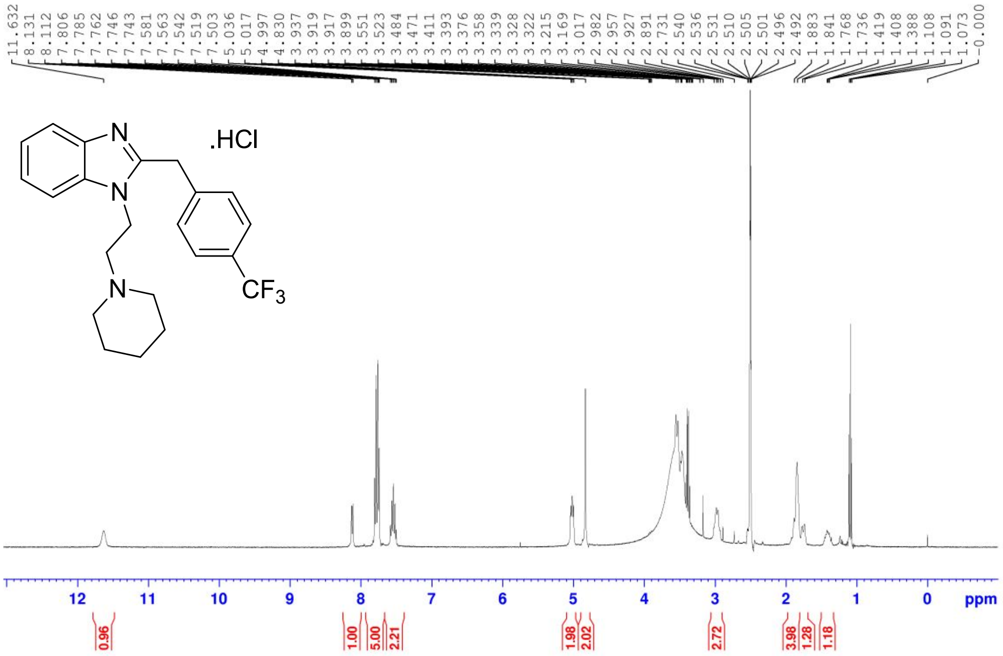

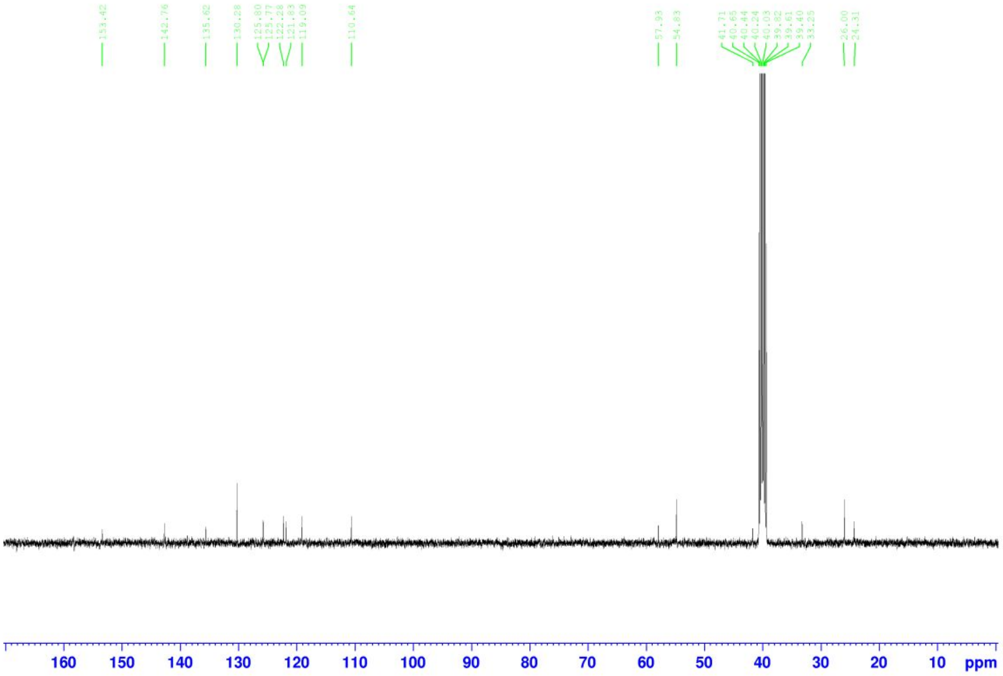
153.42
142.76
135.62
130.28
125.80
125.77
122.28
121.83
119.09
110.64
57.93
54.83
41.71
40.65
40.44
40.24
40.03
39.82
39.61
39.40
33.25
26.00
24.31
160
150
140
130
120
110
100
90
80
70
60
50
40
30
20
10
ppm

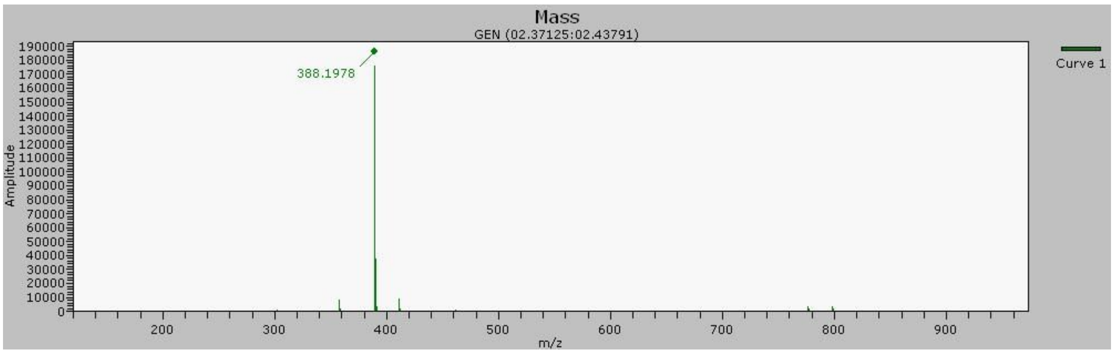
Mass
GEN (02.37125:02.43791)
Curve 1
388.1978
Amplitude
190000
180000
170000
160000
150000
140000
130000
120000
110000
100000
90000
80000
70000
60000
50000
40000
30000
20000
10000
0
200
300
400
500
600
700
800
900
m/z

**2-(4-fluorobenzyl)-1-(2-(piperidin-1-yl)ethyl)-1*H*-benzo[*d*]imidazole hydrochloride (27).**

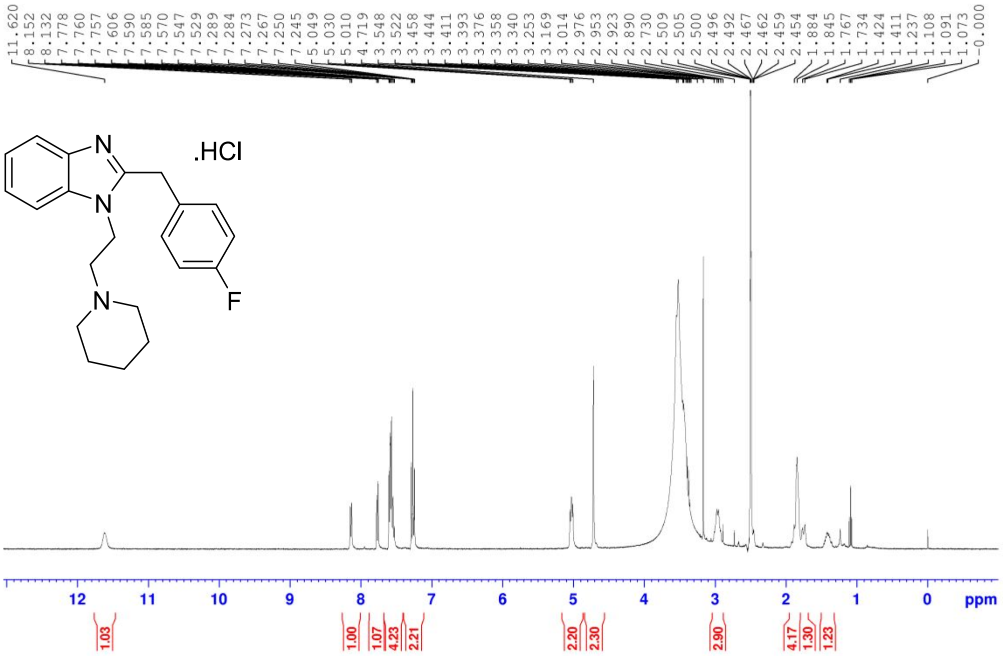


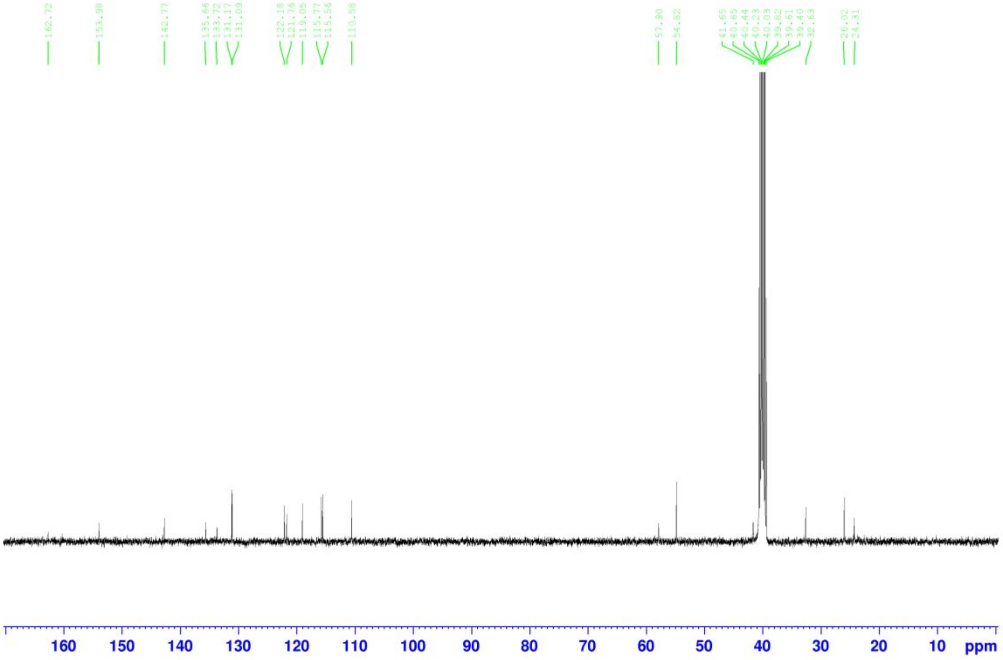

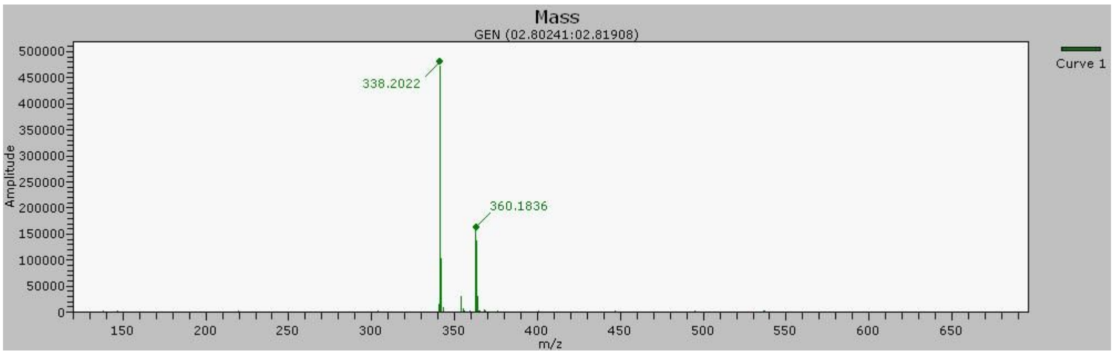


**2-(4-ethylbenzyl)-1-(2-(piperidin-1-yl)ethyl)-1*H*-benzo[*d*]imidazole hydrochloride (28)**.

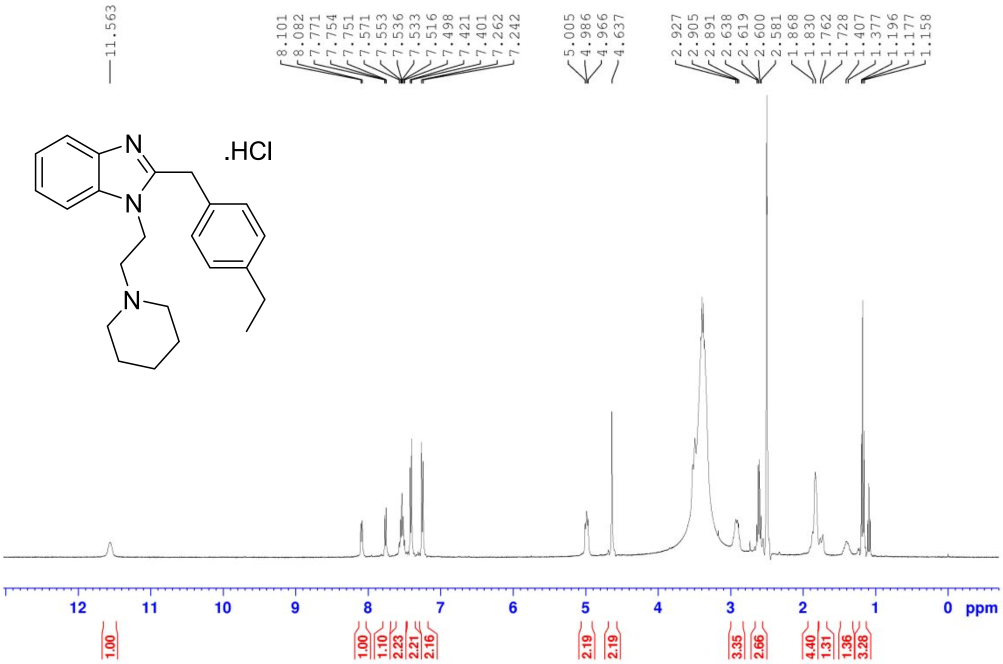

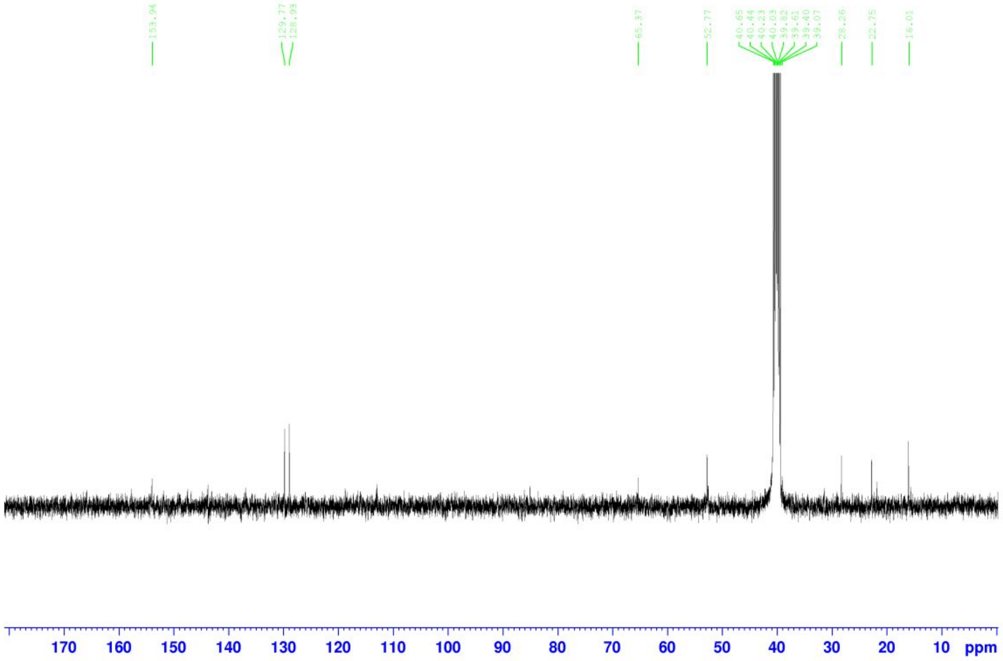
153.94
129.77
128.93
65.37
52.77
40.65
40.44
40.23
40.03
39.82
39.61
39.40
39.07
28.26
22.75
16.01
170
160
150
140
130
120
110
100
90
80
70
60
50
40
30
20
10
ppm

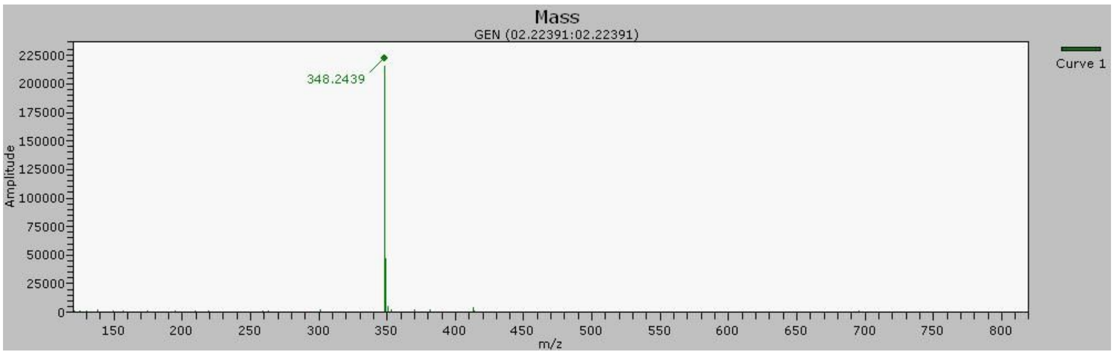
Mass
GEN (02.22391:02.22391)
Curve 1
348.2439
Amplitude
225000
200000
175000
150000
125000
100000
75000
50000
25000
0
150
200
250
300
350
400
450
500
550
600
650
700
750
800
m/z

**2-(4-ethoxybenzyl)-1-(2-(piperidin-1-yl)ethyl)-1H-benzo[d]imidazole hydrochloride (29).**

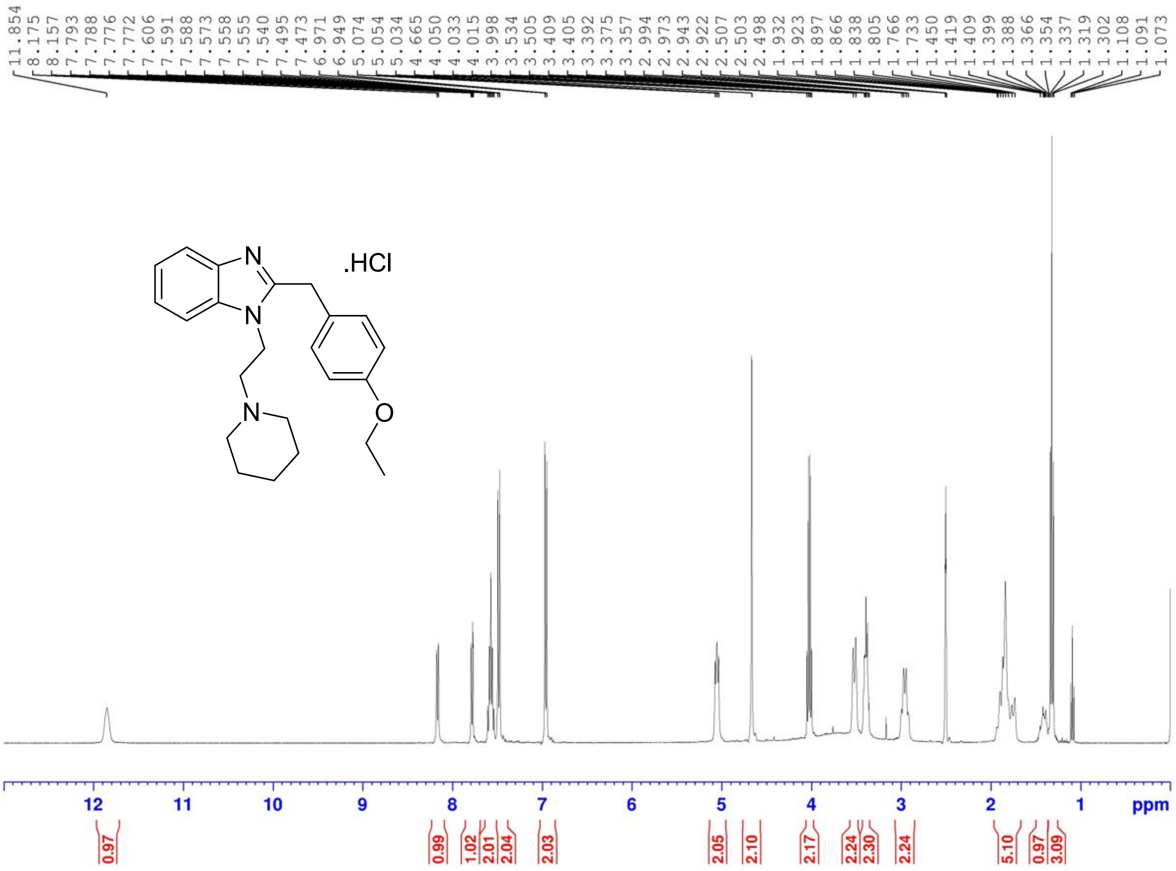


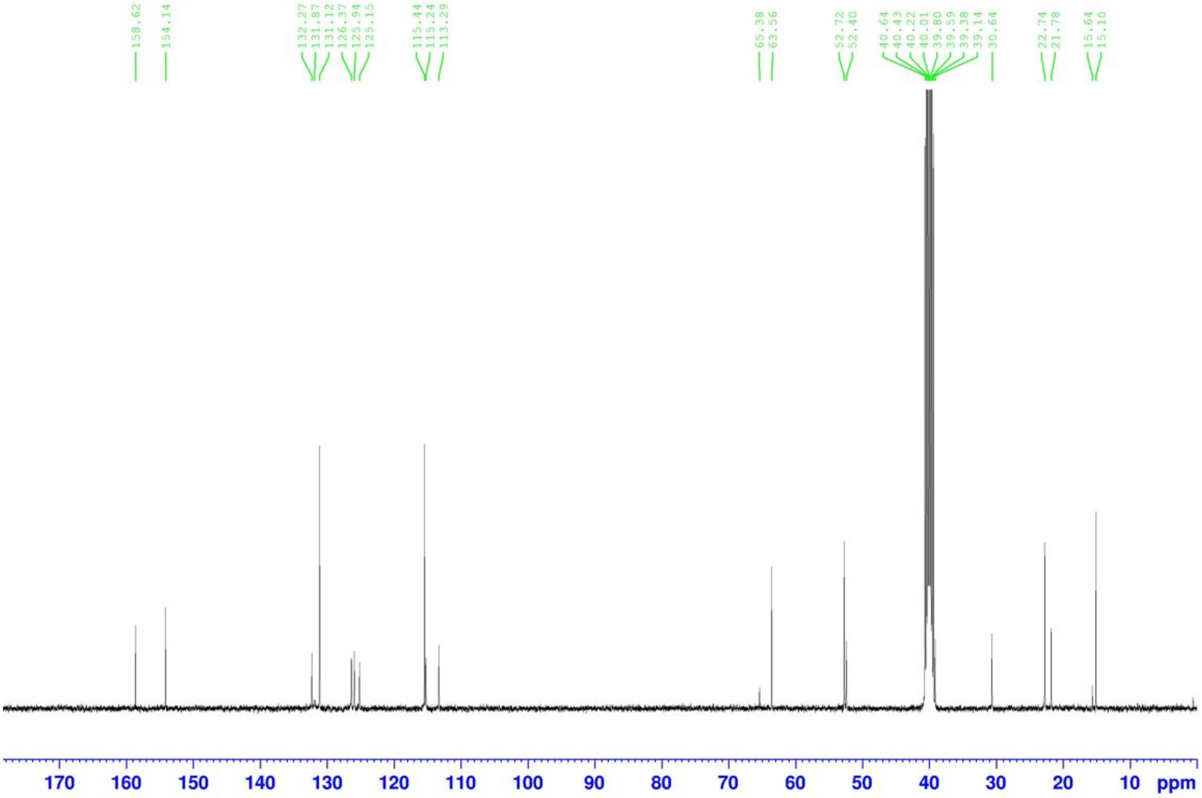

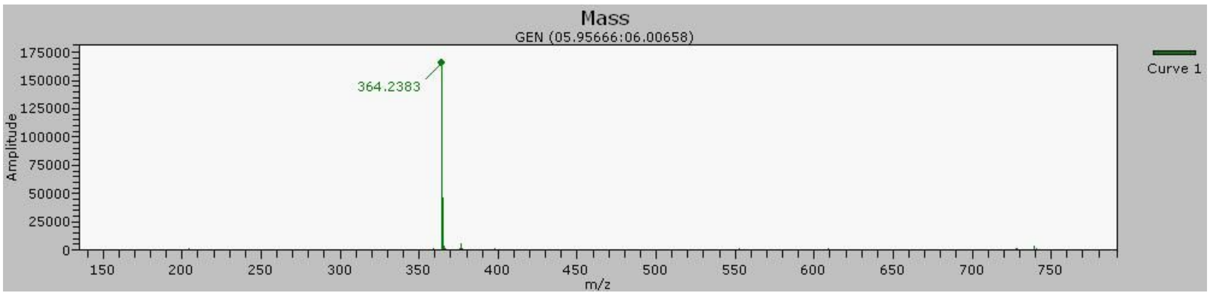


**2-(4-isopropylbenzyl)-1-(2-(piperidin-1-yl)ethyl)-1*H*-benzo[*d*]imidazole hydrochloride (30).**

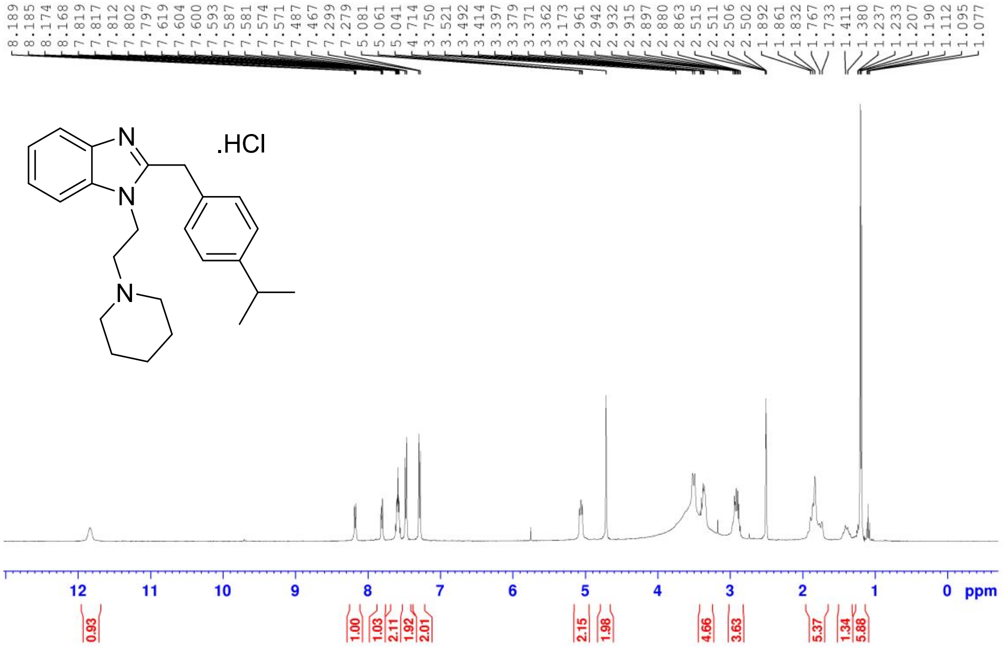

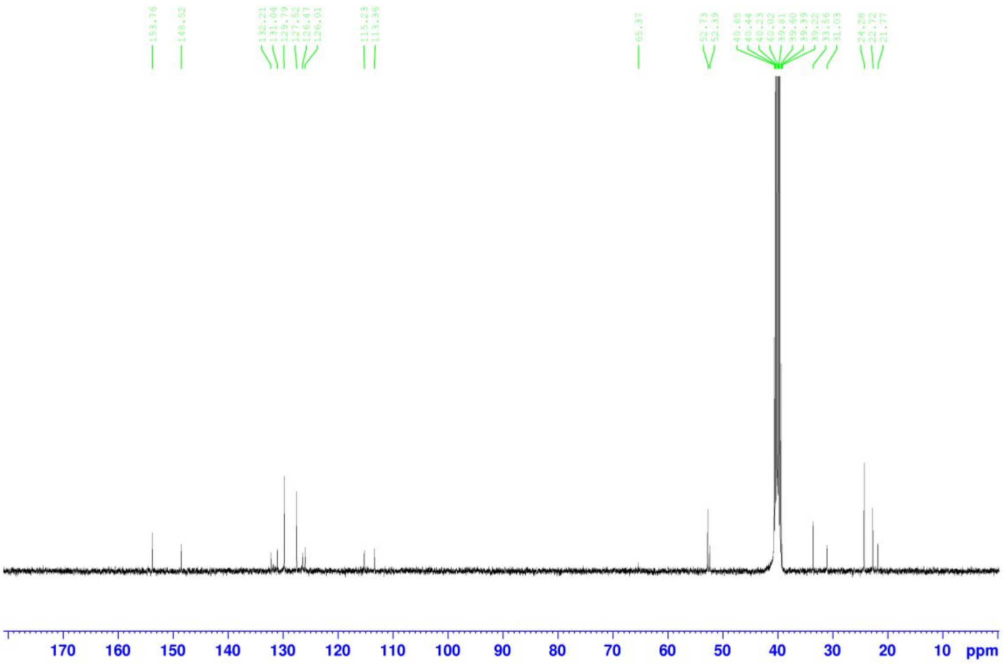
153.76
148.52
132.21
131.04
129.79
127.52
126.47
126.01
115.23
113.36
65.37
52.73
52.39
40.65
40.44
40.23
40.02
39.81
39.60
39.39
39.22
33.56
31.03
24.28
22.72
21.77
170 160 150 140 130 120 110 100 90 80 70 60 50 40 30 20 10 ppm

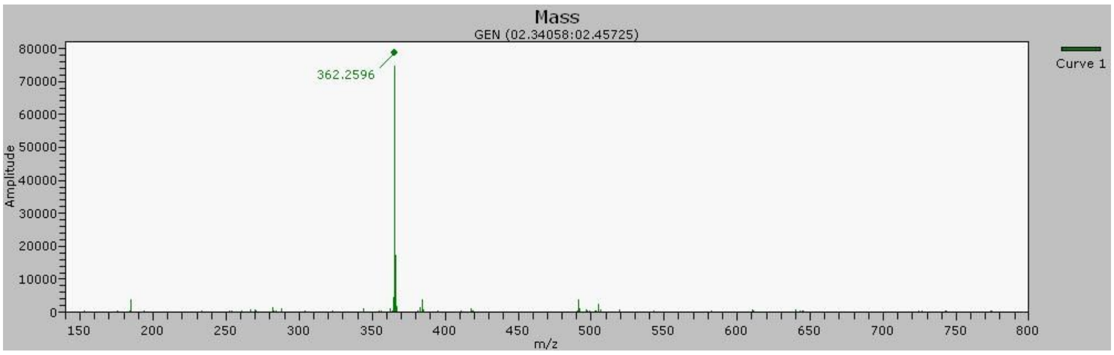
Mass
GEN (02.34058:02.45725)
362.2596
Curve 1
Amplitude
80000 70000 60000 50000 40000 30000 20000 10000 0
150 200 250 300 350 400 450 500 550 600 650 700 750 800
m/z

**2-(4-methylbenzyl)-1-(2-(piperidin-1-yl)ethyl)-1*H*-benzo[*d*]imidazole hydrochloride (31).**

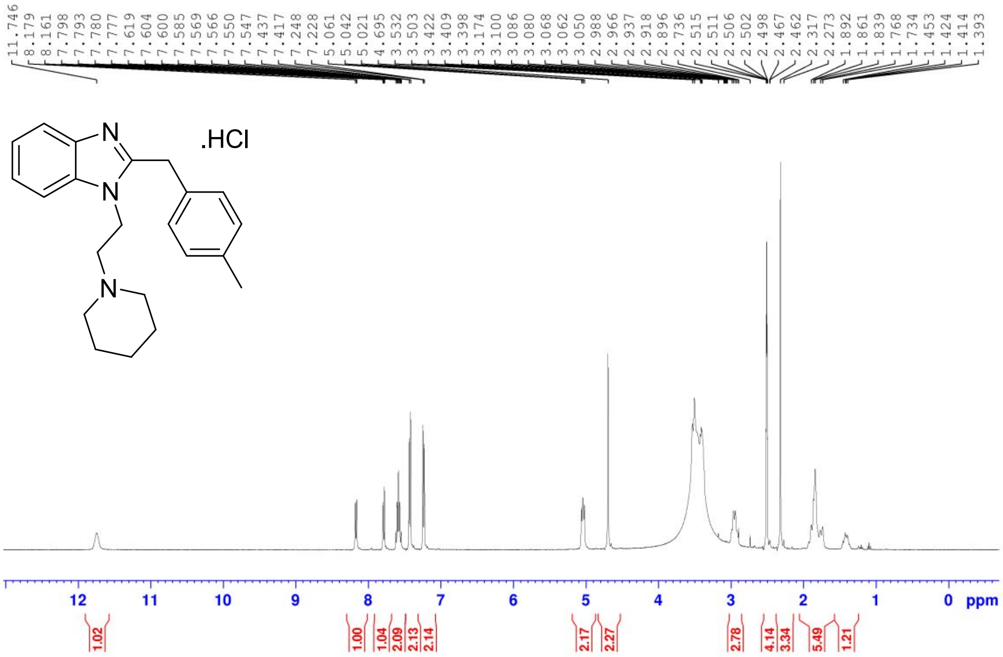


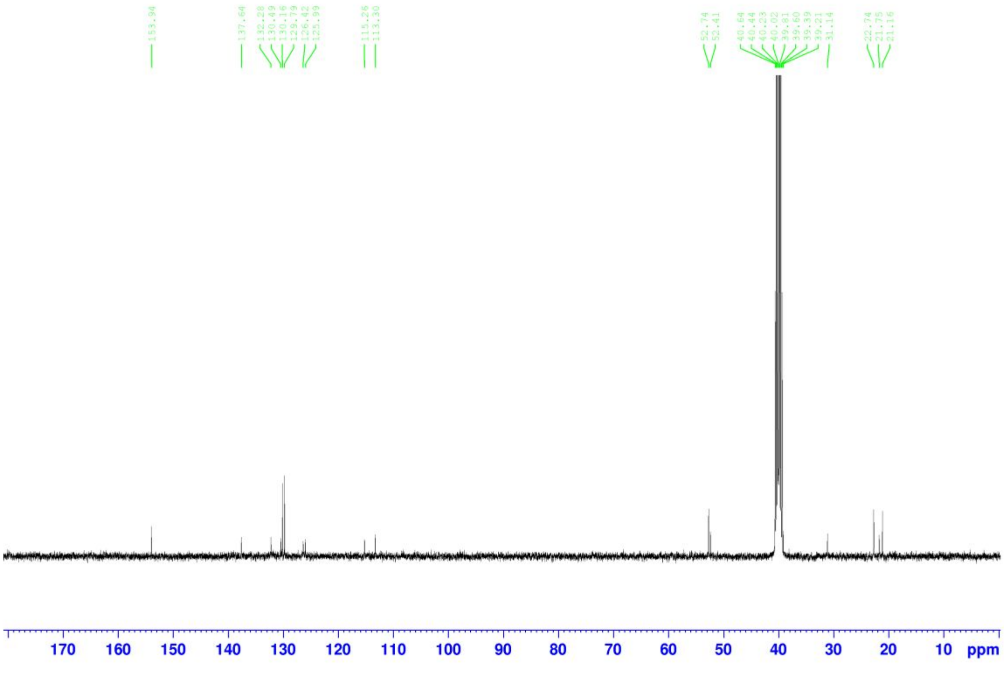

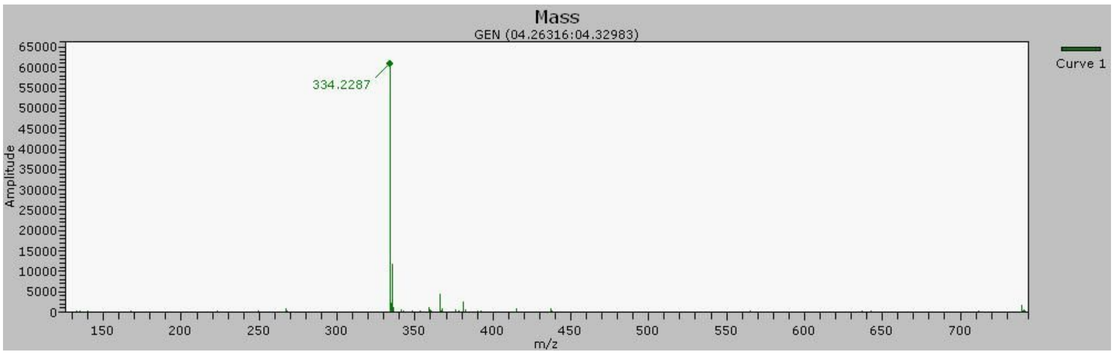


**4-((1-(2-(piperidin-1-yl)ethyl)-1*H*-benzo[d]imidazol-2-yl)methyl)benzonitrile hydrochloride (32).**

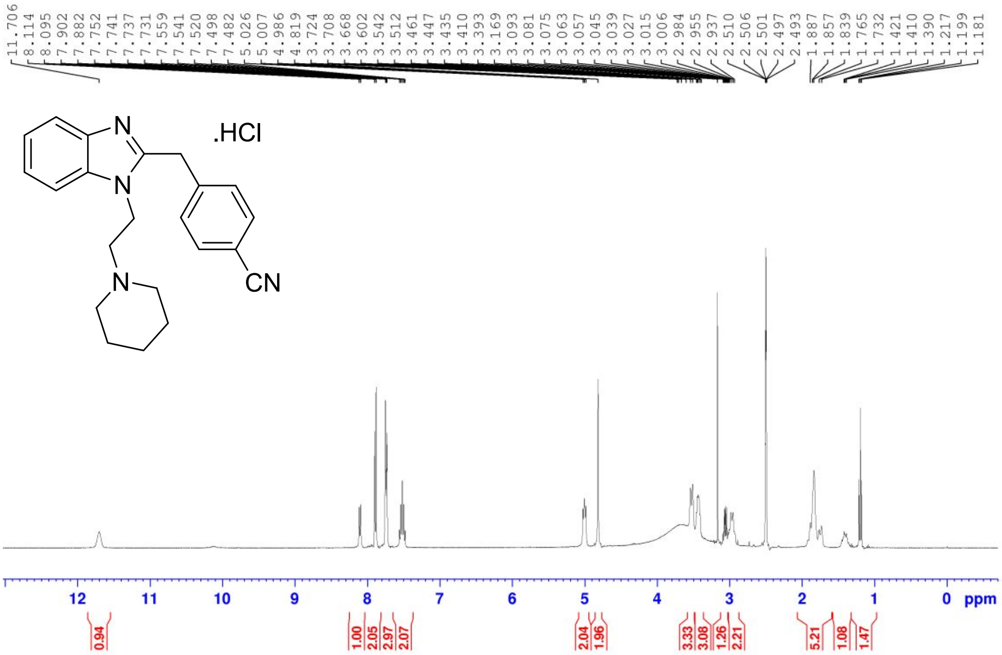

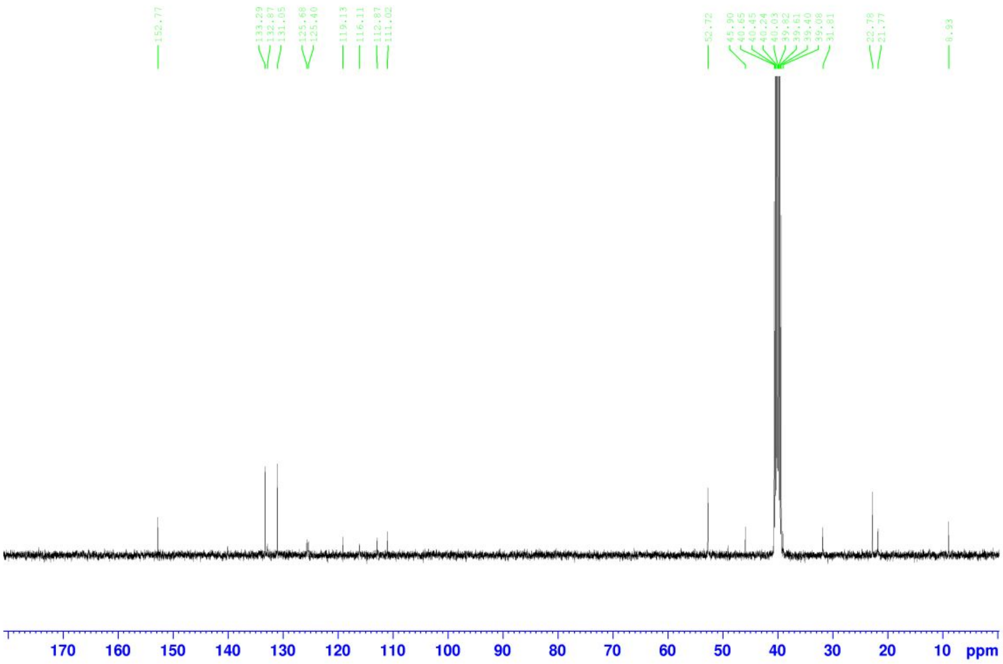
152.77
133.29
132.87
131.05
125.68
125.40
119.13
116.11
112.87
111.02
52.72
45.90
40.65
40.45
40.24
40.03
39.82
39.61
39.40
39.08
31.81
22.78
21.77
8.93
170 160 150 140 130 120 110 100 90 80 70 60 50 40 30 20 10 ppm

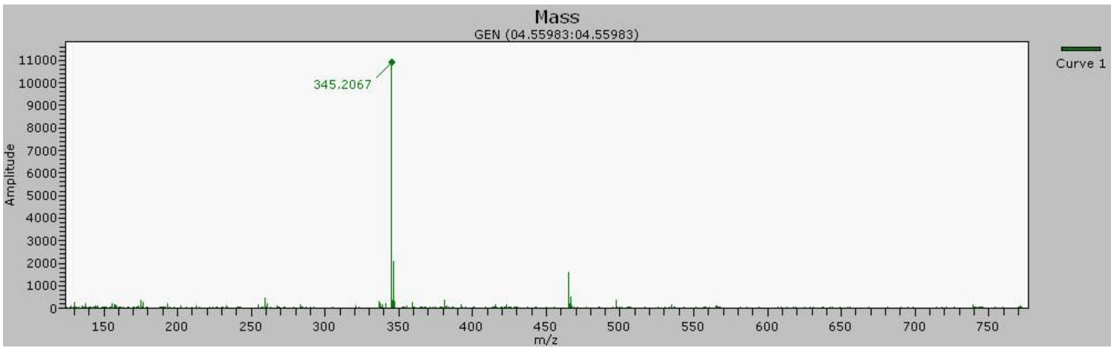
Mass
GEN (04.55983:04.55983)
Curve 1
345.2067
Amplitude
m/z
0 1000 2000 3000 4000 5000 6000 7000 8000 9000 10000 11000
150 200 250 300 350 400 450 500 550 600 650 700 750

## 5. Purity Data of Final Compounds.

HPLC System: Waters Arc HPLC

Column: XBridge™ $C_{18}$ 3.5 µm (4.6 x 50 mm)

Injection Volume: 5 µL

Mobile Phase: water/acetonitrile (30/70) with 0.01% trifluoroacetic acid

Flow Rate: 0.2 ml/min

Sample Concentration: 0.125 mg/mL

Single Wavelength: 210 nm

**Table S2.** HPLC Analysis of Target Compounds.

| Compound | Retention Time (min) | Purity (%) |
|---|---|---|
| **Etonitazene** | 2.50 | 98.35 |
| **1** | 2.29 | 100.00 |
| **2** | 2.28 | 100.00 |
| **3** | 2.31 | 99.56 |
| **4** | 2.34 | 99.62 |
| **5** | 2.31 | 99.54 |
| **6** | 2.32 | 99.52 |
| **7** | 2.30 | 99.12 |
| **8** | 2.31 | 98.83 |
| **9** | 2.50 | 98.35 |
| **10** | 2.31 | 96.66 |
| **12** | 2.33 | 97.58 |
| **13** | 2.35 | 98.57 |
| **14** | 2.33 | 96.47 |
| **15** | 2.30 | 99.25 |
| **16** | 2.28 | 98.52 |
| **17** | 2.34 | 96.46 |
| **18** | 2.33 | 99.54 |
| **19** | 2.31 | 99.05 |
| **20** | 2.34 | 98.04 |
| **21** | 2.31 | 99.57 |
| **22** | 2.32 | 99.28 |

| | | |
|---|---|---|
| **23** | 2.29 | 99.08 |
| **24** | 2.28 | 98.37 |
| **25** | 2.31 | 99.01 |
| **26** | 2.31 | 99.33 |
| **27** | 2.32 | 97.78 |
| **28** | 2.34 | 98.32 |
| **29** | 2.31 | 99.01 |
| **30** | 2.32 | 99.23 |
| **31** | 2.33 | 99.27 |
| **32** | 2.29 | 99.46 |

## 6. HPLC Chromatograms of Final Compounds

### Etonitazene hydrochloride

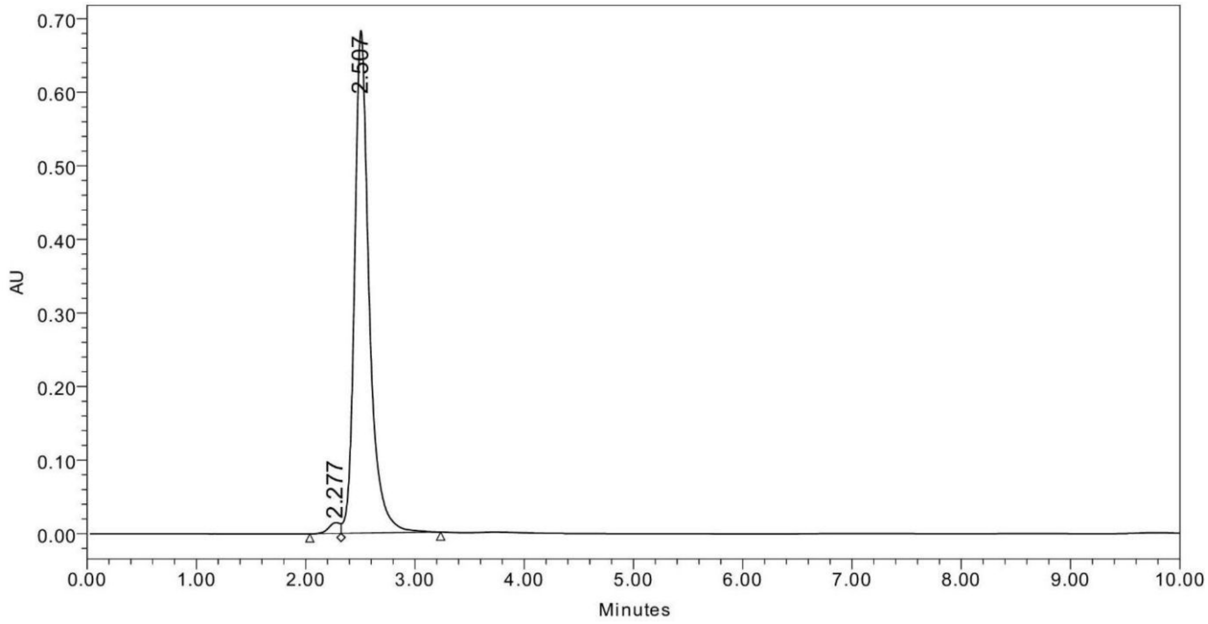

**1**

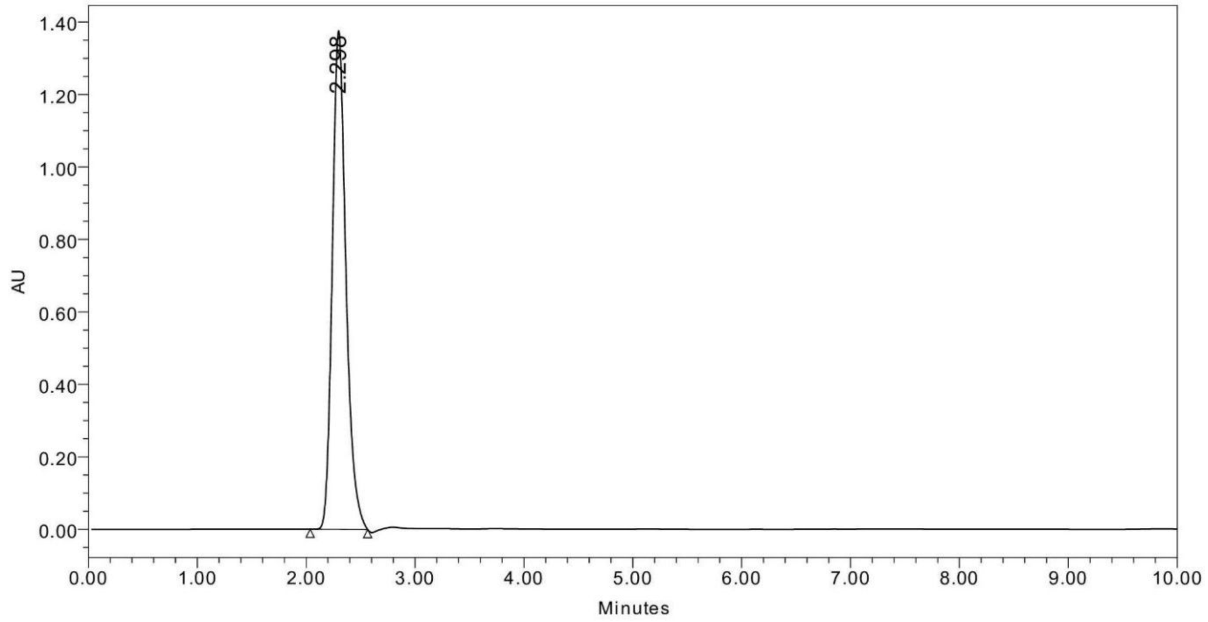

2.298
AU
1.40
1.20
1.00
0.80
0.60
0.40
0.20
0.00
0.00
1.00
2.00
3.00
4.00
5.00
6.00
7.00
8.00
9.00
10.00
Minutes


**2**

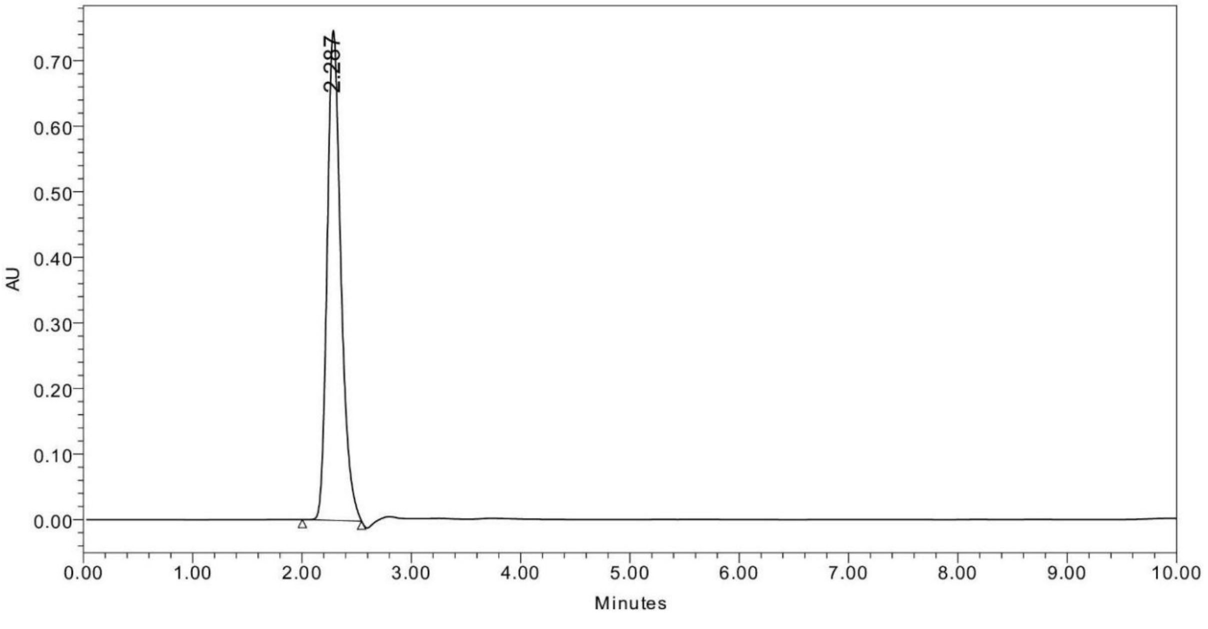

2.287
AU
0.70
0.60
0.50
0.40
0.30
0.20
0.10
0.00
0.00
1.00
2.00
3.00
4.00
5.00
6.00
7.00
8.00
9.00
10.00
Minutes

**3**

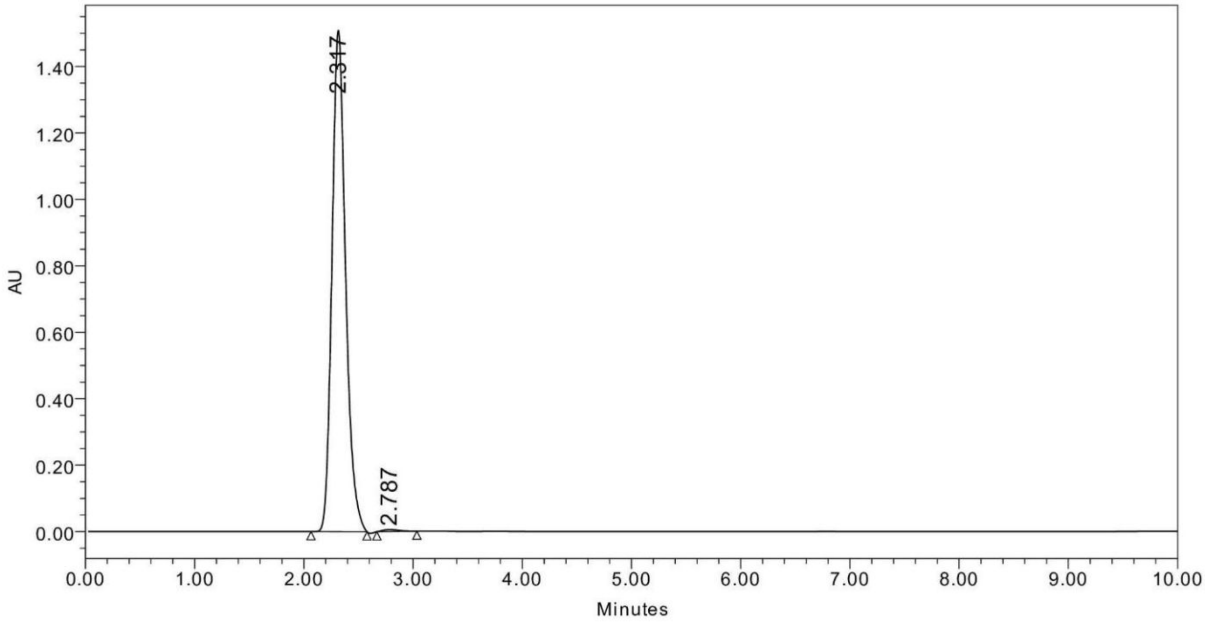


**4**

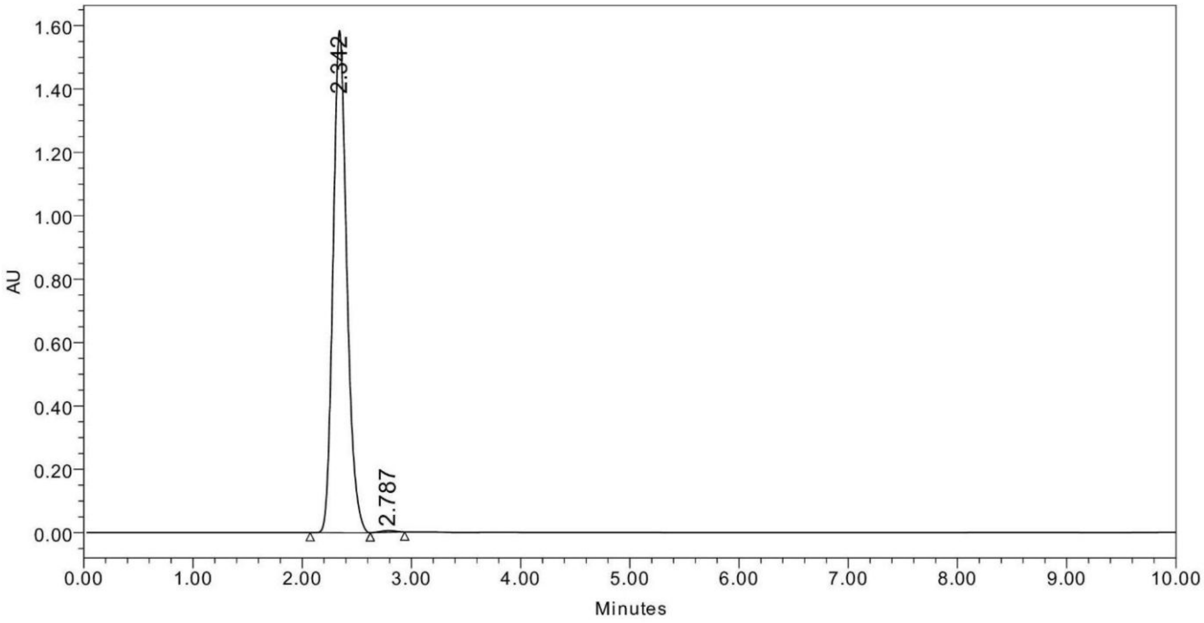

**5**

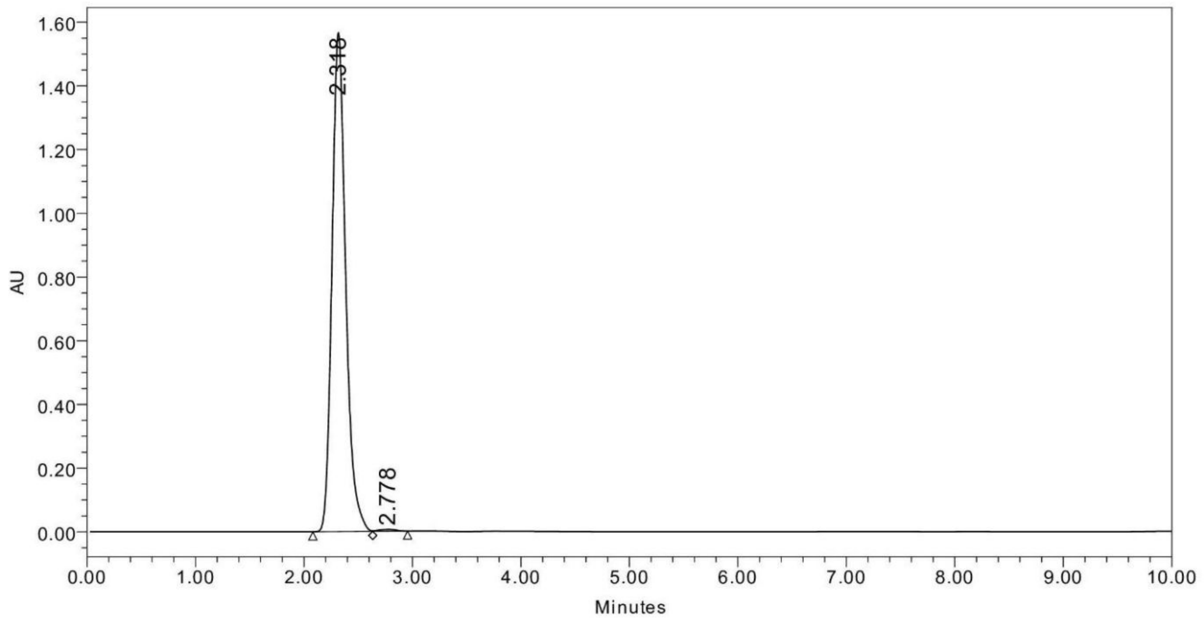


**6**

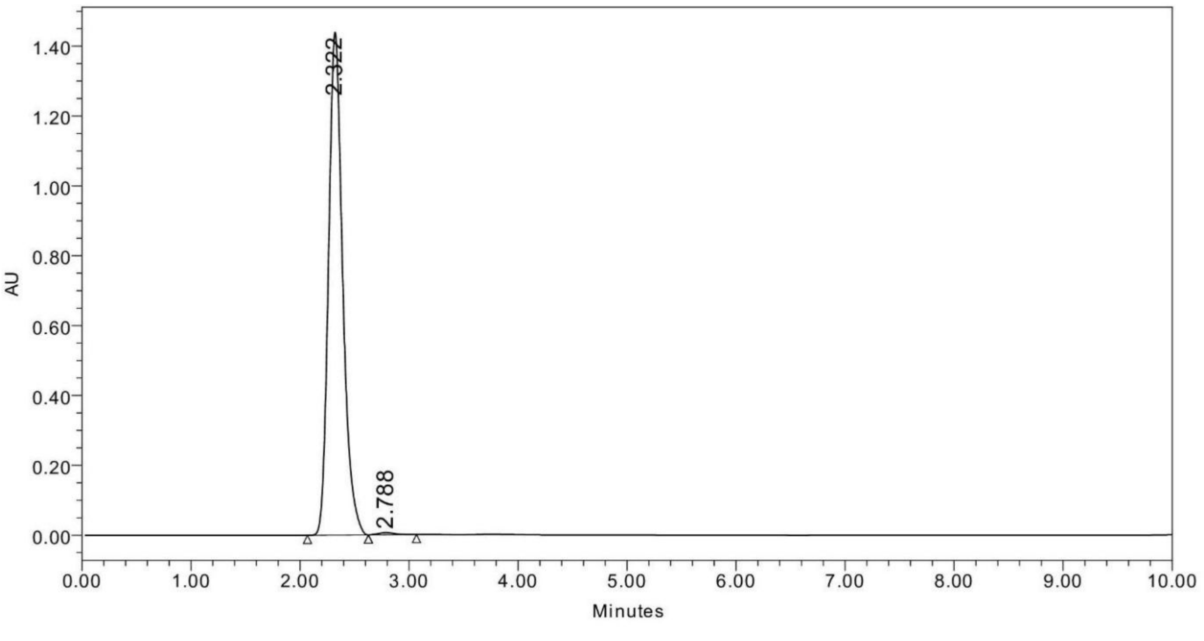

**7**

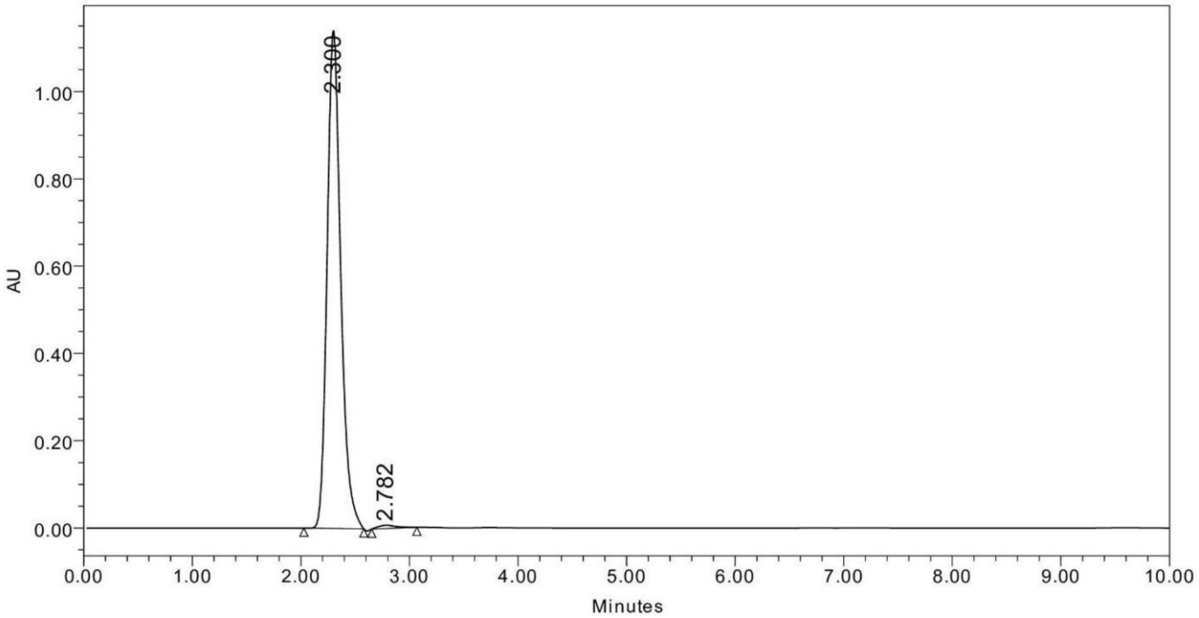

2.300
2.782
1.00
0.80
0.60
0.40
0.20
0.00
AU
0.00 1.00 2.00 3.00 4.00 5.00 6.00 7.00 8.00 9.00 10.00
Minutes


**8**

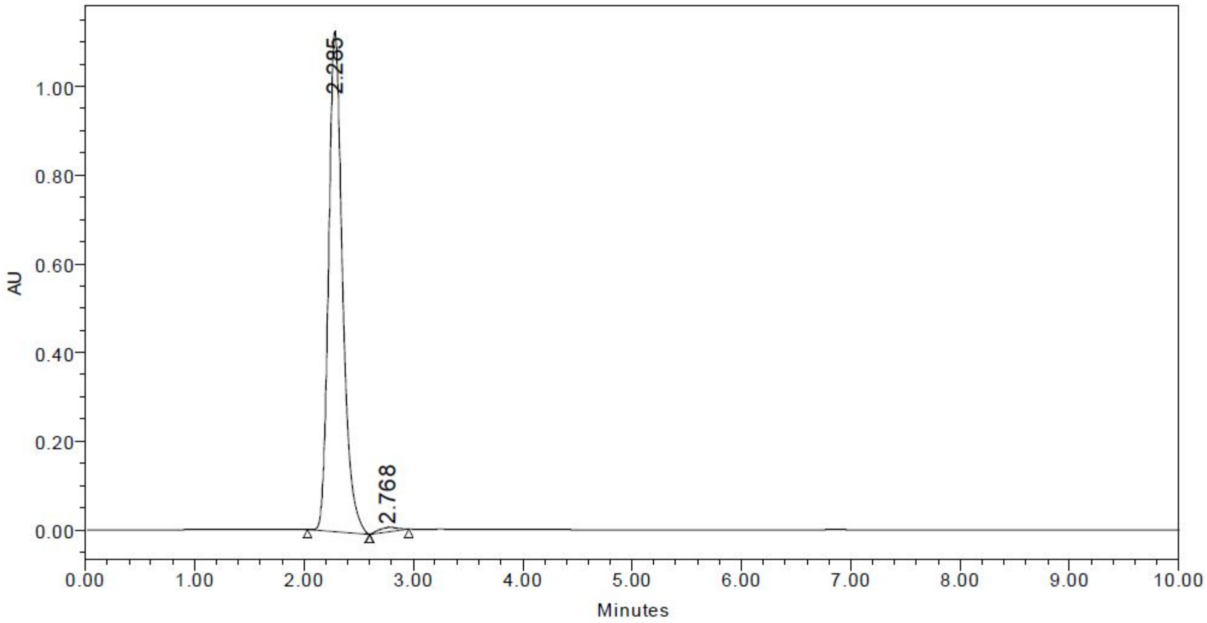

2.285
2.768
1.00
0.80
0.60
0.40
0.20
0.00
AU
0.00 1.00 2.00 3.00 4.00 5.00 6.00 7.00 8.00 9.00 10.00
Minutes

**9**

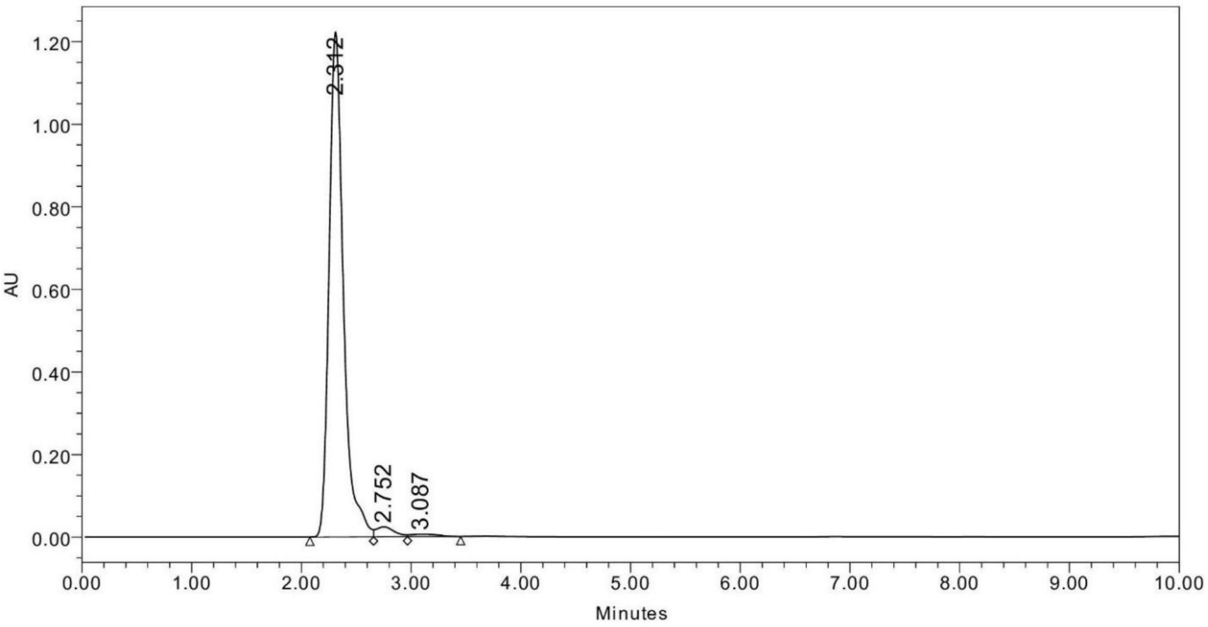
2.312
2.752
3.087
AU
Minutes
1.20
1.00
0.80
0.60
0.40
0.20
0.00
0.00
1.00
2.00
3.00
4.00
5.00
6.00
7.00
8.00
9.00
10.00

**10**

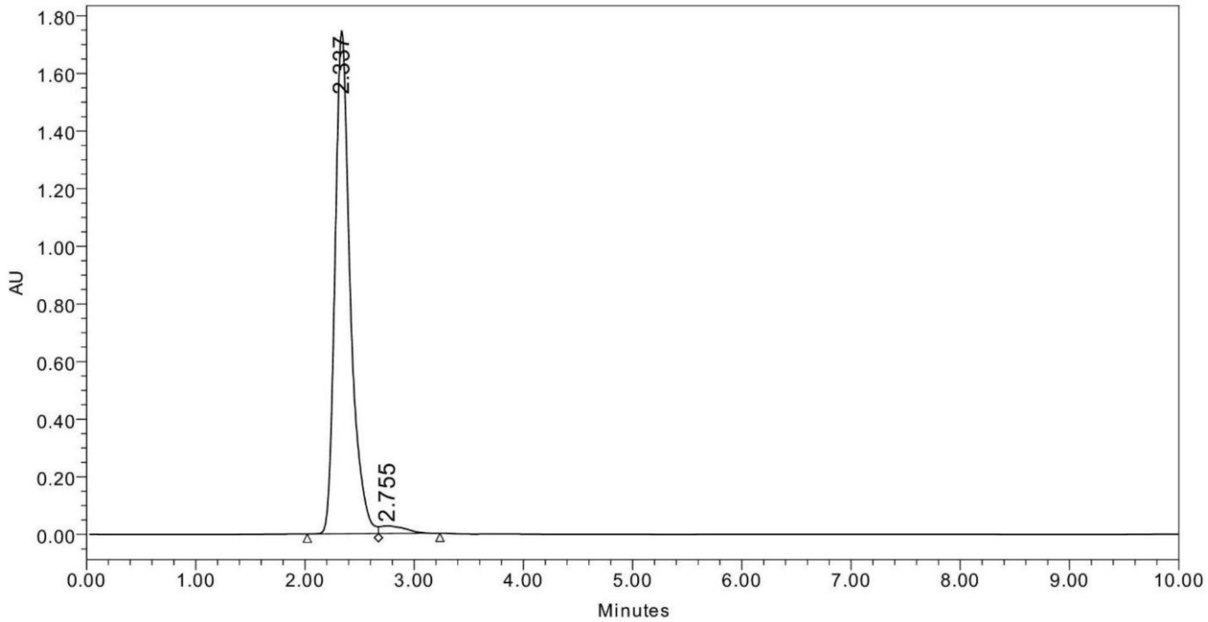
2.337
2.755
AU
Minutes
1.80
1.60
1.40
1.20
1.00
0.80
0.60
0.40
0.20
0.00
0.00
1.00
2.00
3.00
4.00
5.00
6.00
7.00
8.00
9.00
10.00

**11**

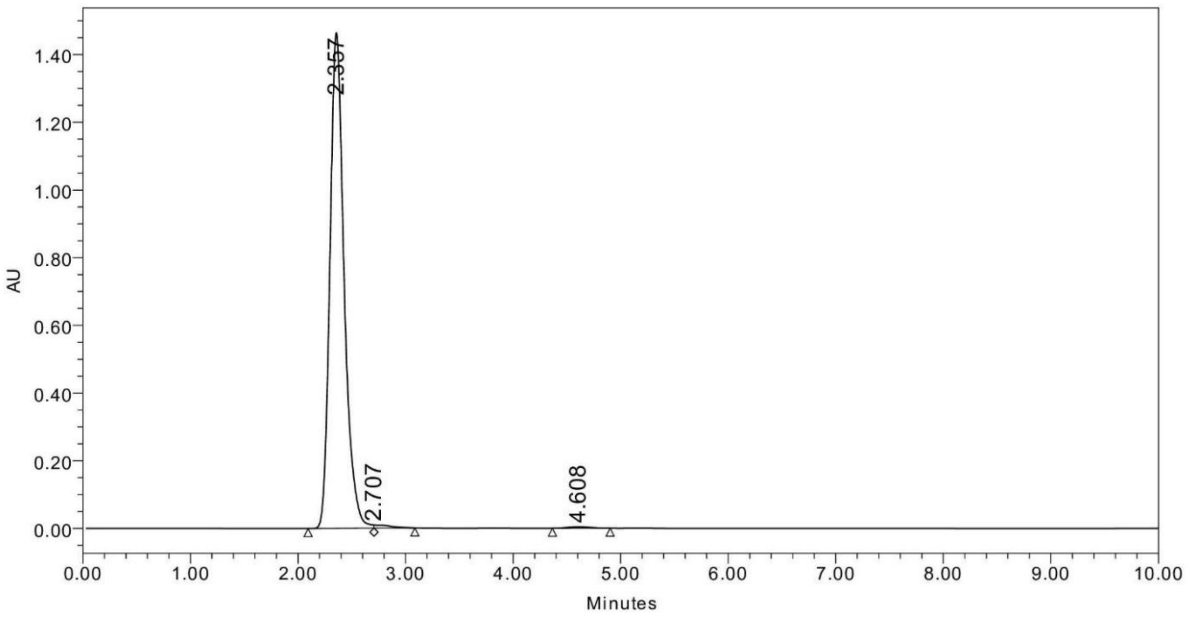


**12**

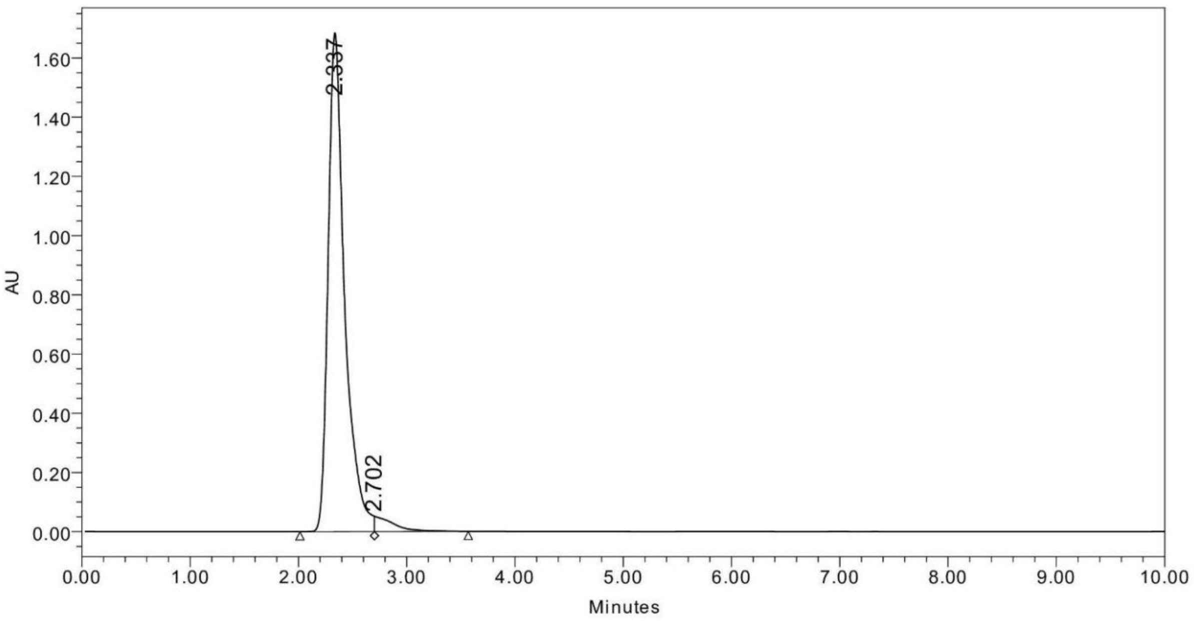

**13**

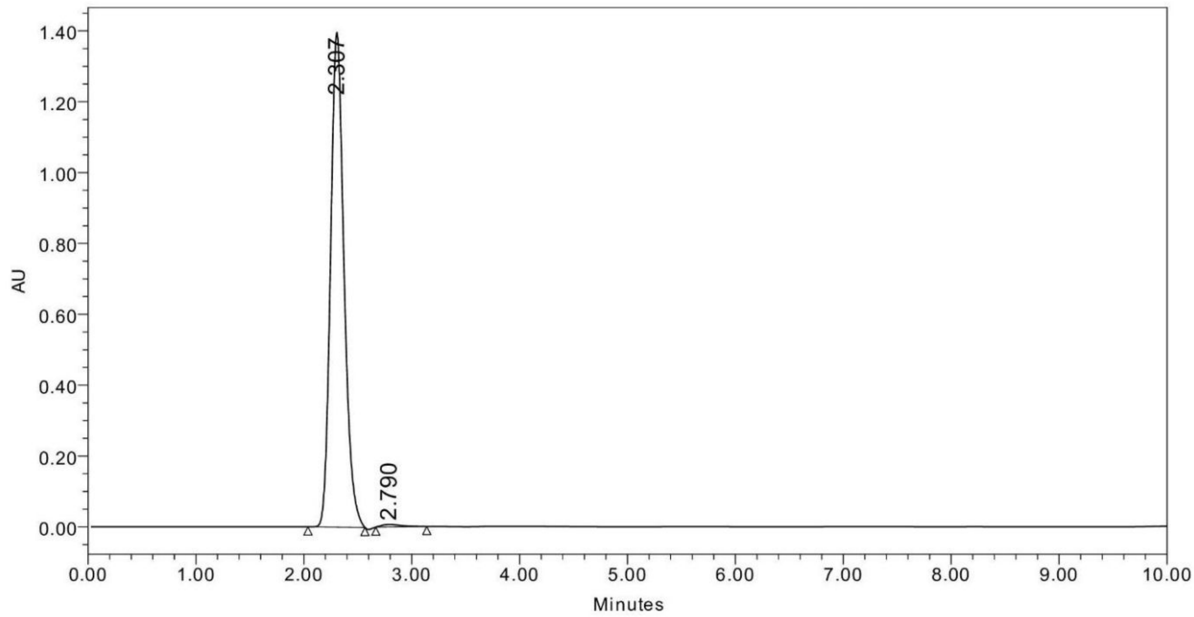

2.307
2.790
AU
1.40
1.20
1.00
0.80
0.60
0.40
0.20
0.00
0.00
1.00
2.00
3.00
4.00
5.00
6.00
7.00
8.00
9.00
10.00
Minutes


**14**

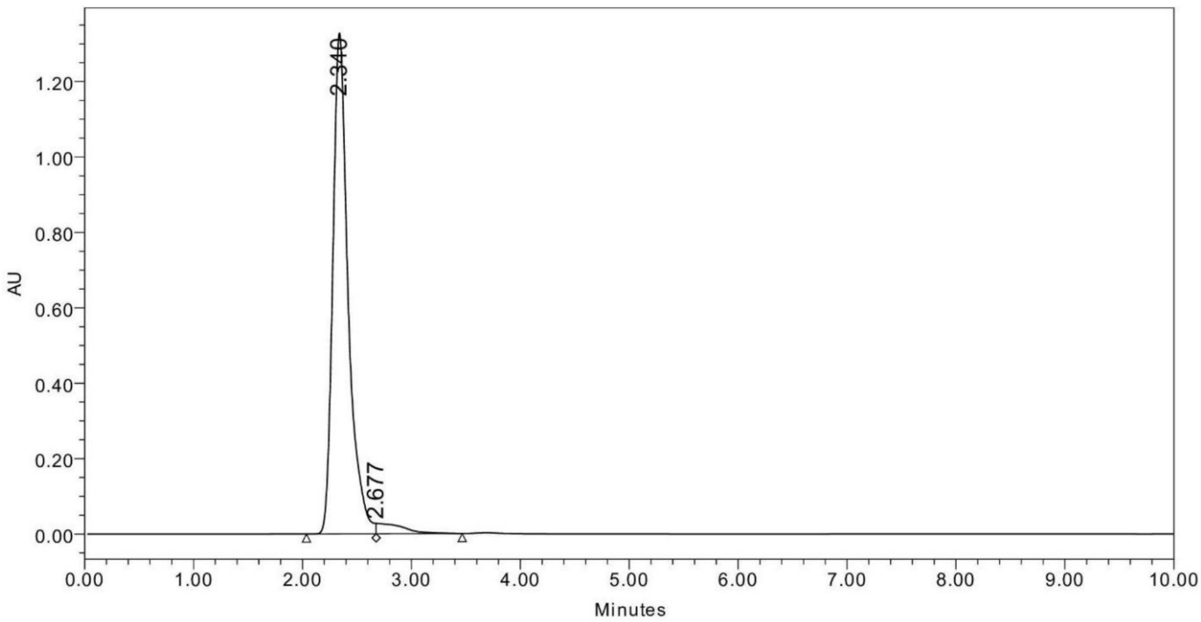

2.340
2.677
AU
1.20
1.00
0.80
0.60
0.40
0.20
0.00
0.00
1.00
2.00
3.00
4.00
5.00
6.00
7.00
8.00
9.00
10.00
Minutes

**15**

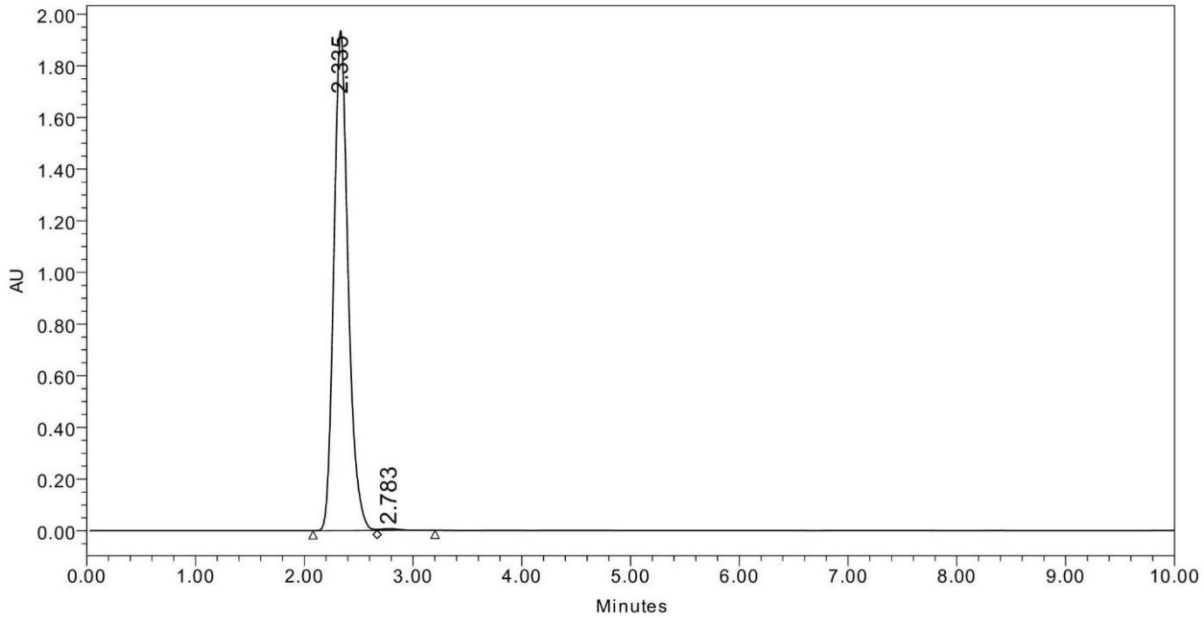


**16**

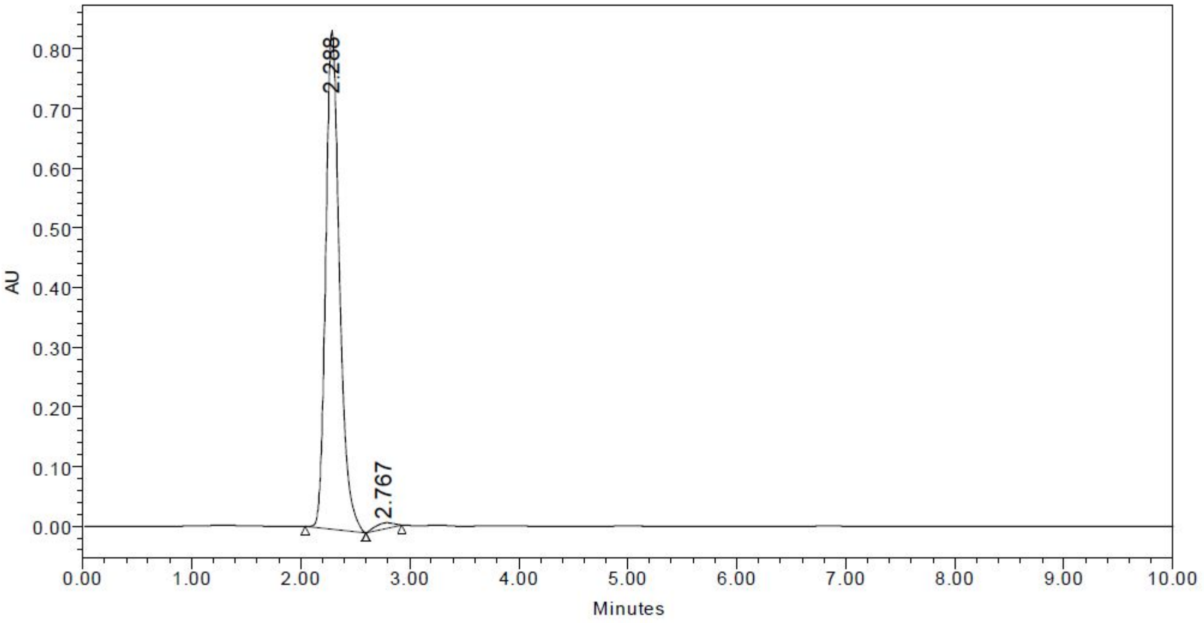

**17**

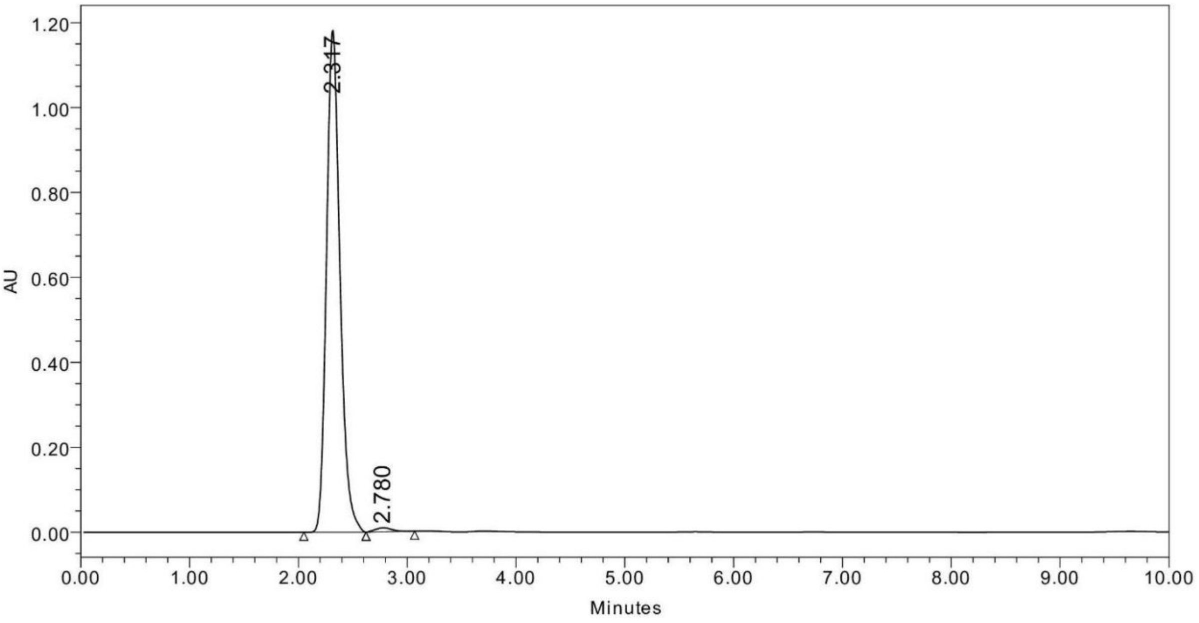


**18**

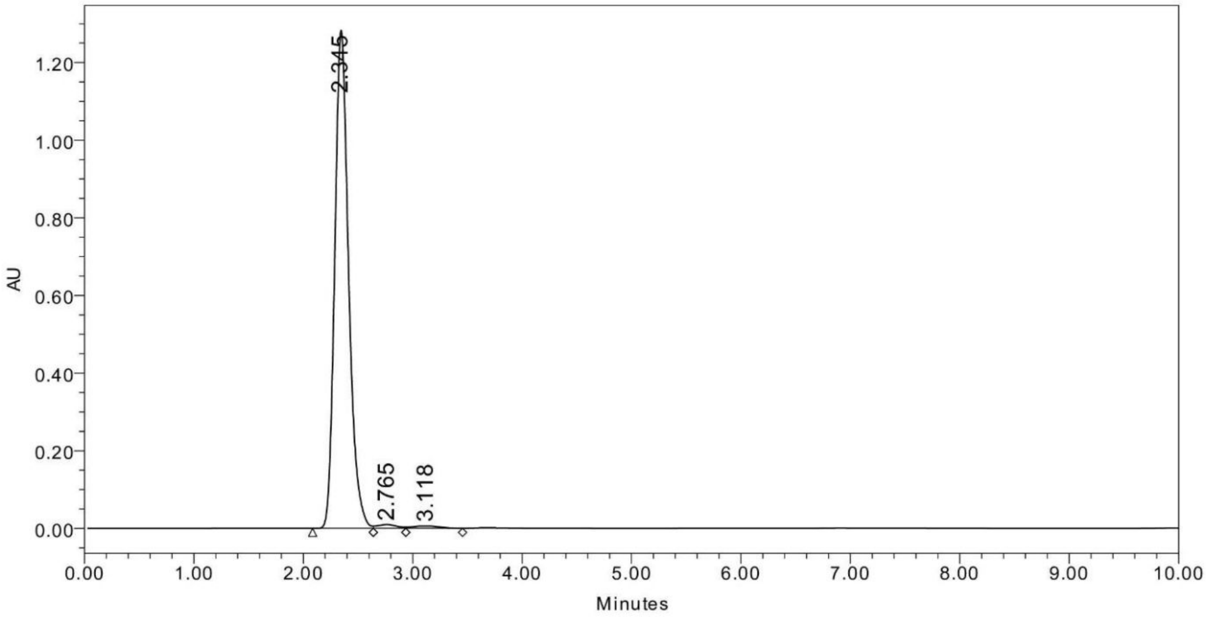

**19**

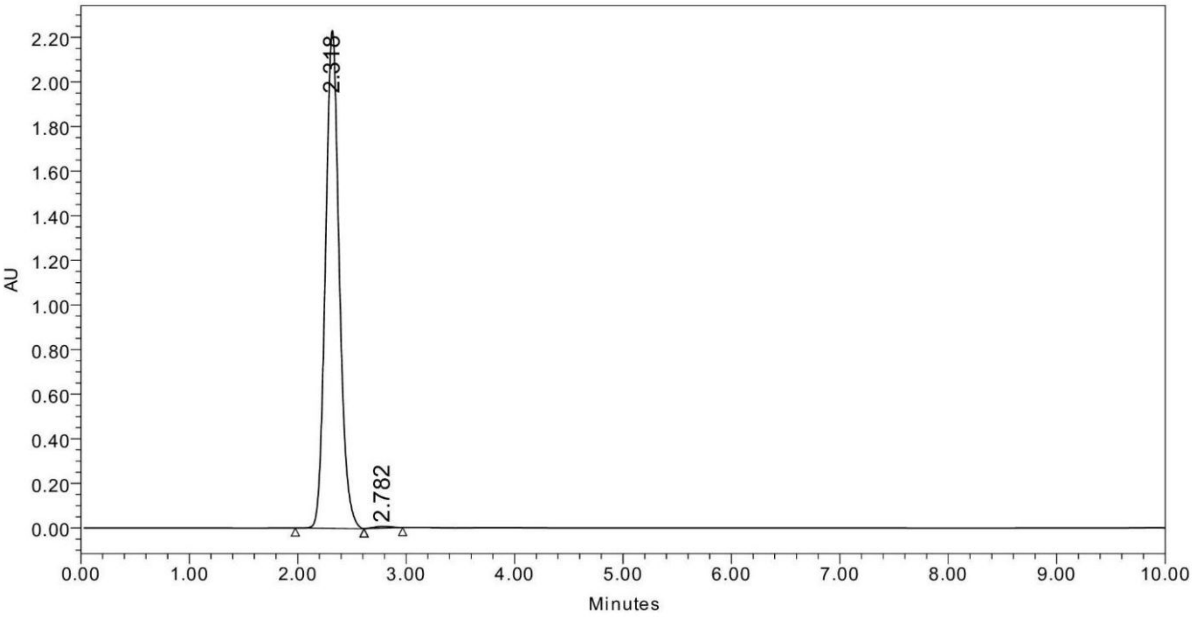


**20**

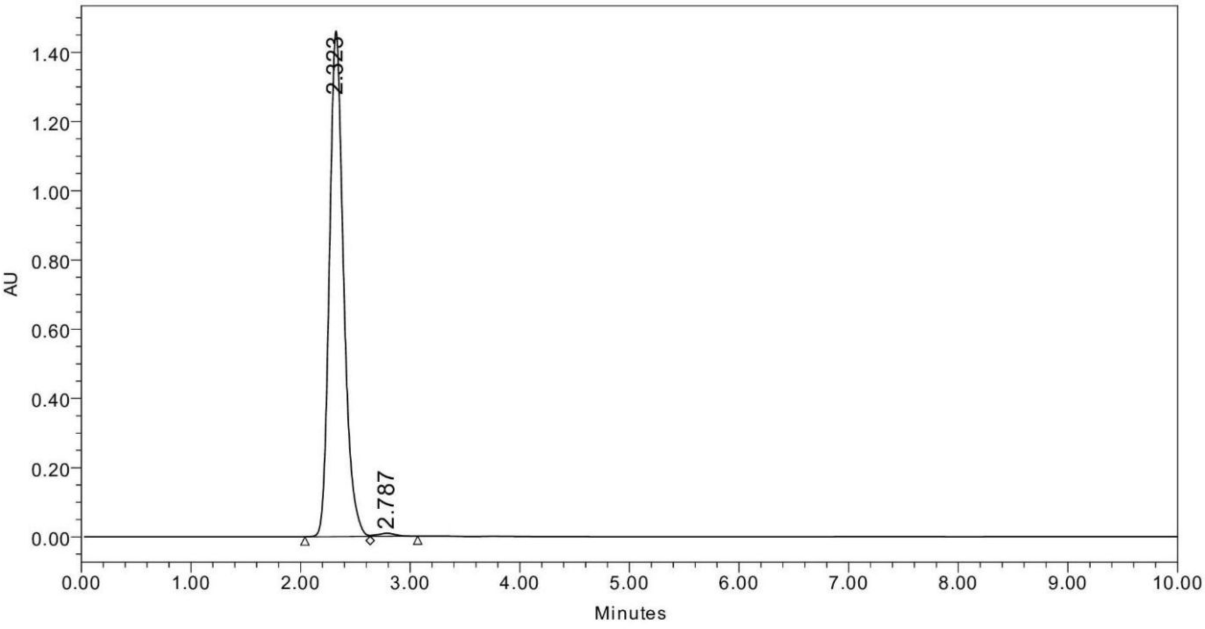

**21**

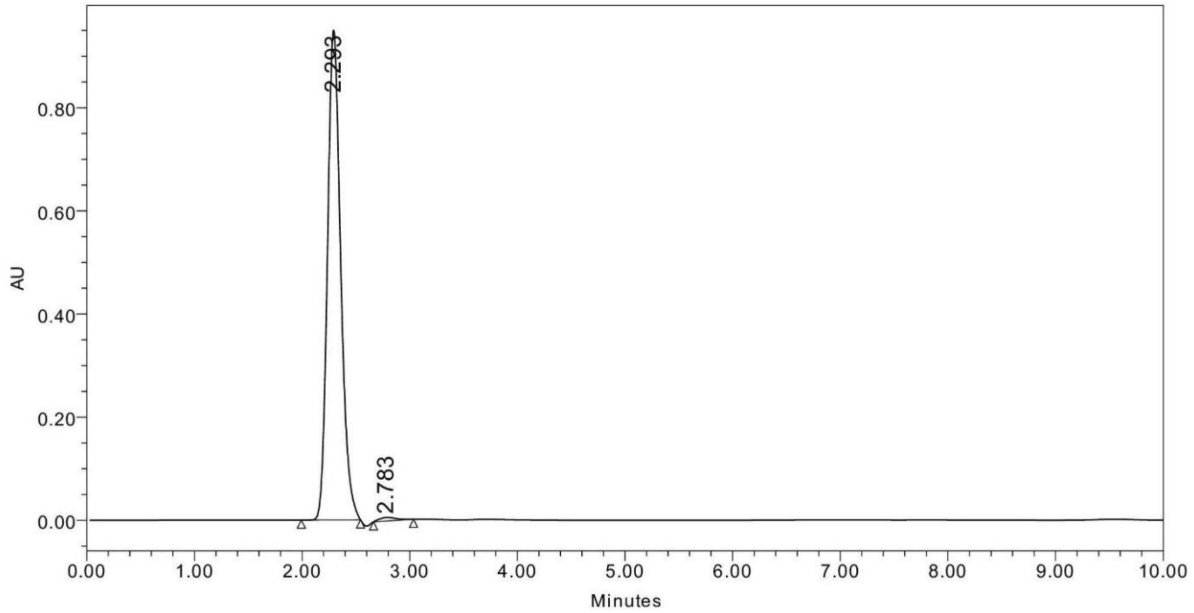
2.293
2.783
0.80
0.60
0.40
0.20
0.00
AU
0.00
1.00
2.00
3.00
4.00
5.00
6.00
7.00
8.00
9.00
10.00
Minutes

**22**

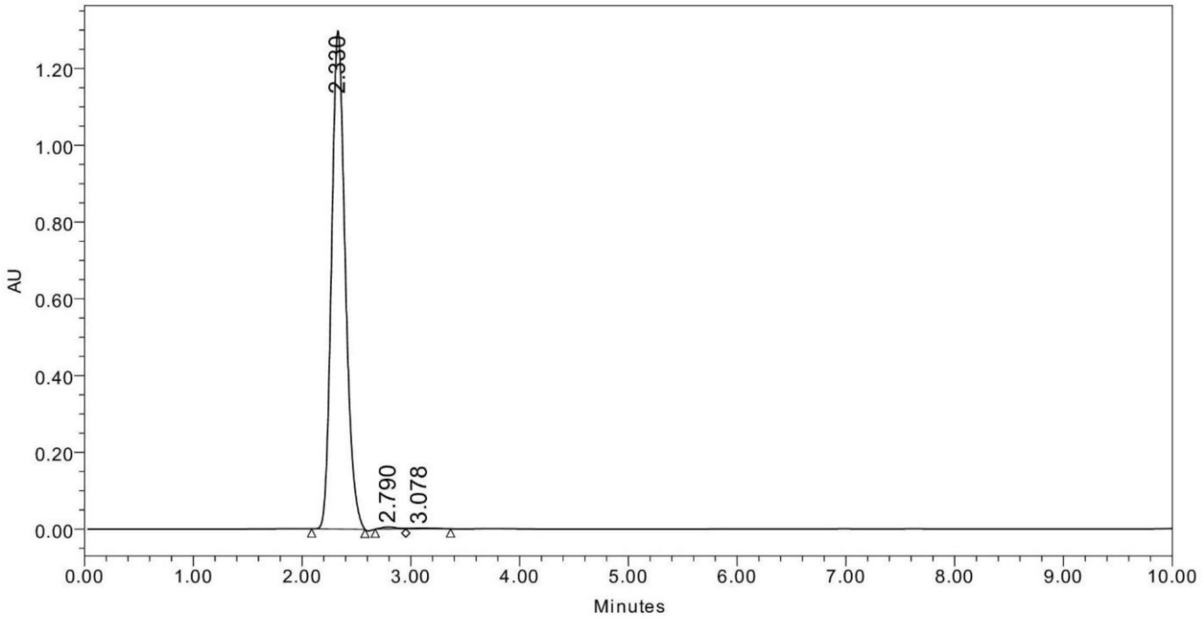
2.330
2.790
3.078
1.20
1.00
0.80
0.60
0.40
0.20
0.00
AU
0.00
1.00
2.00
3.00
4.00
5.00
6.00
7.00
8.00
9.00
10.00
Minutes

**23**

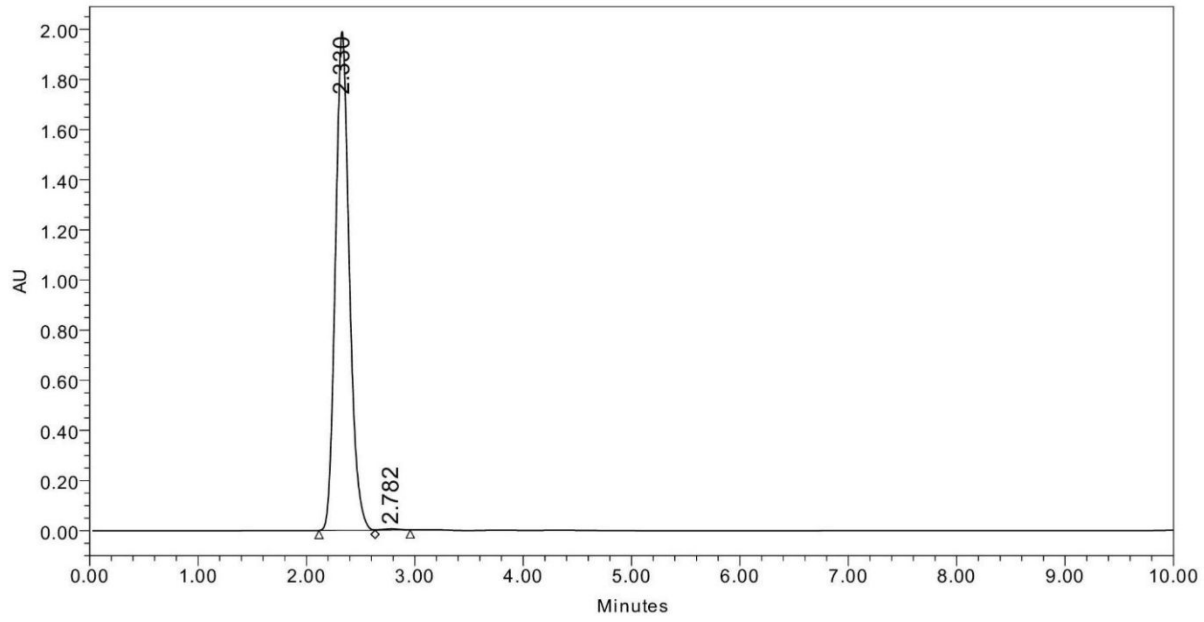


**24**

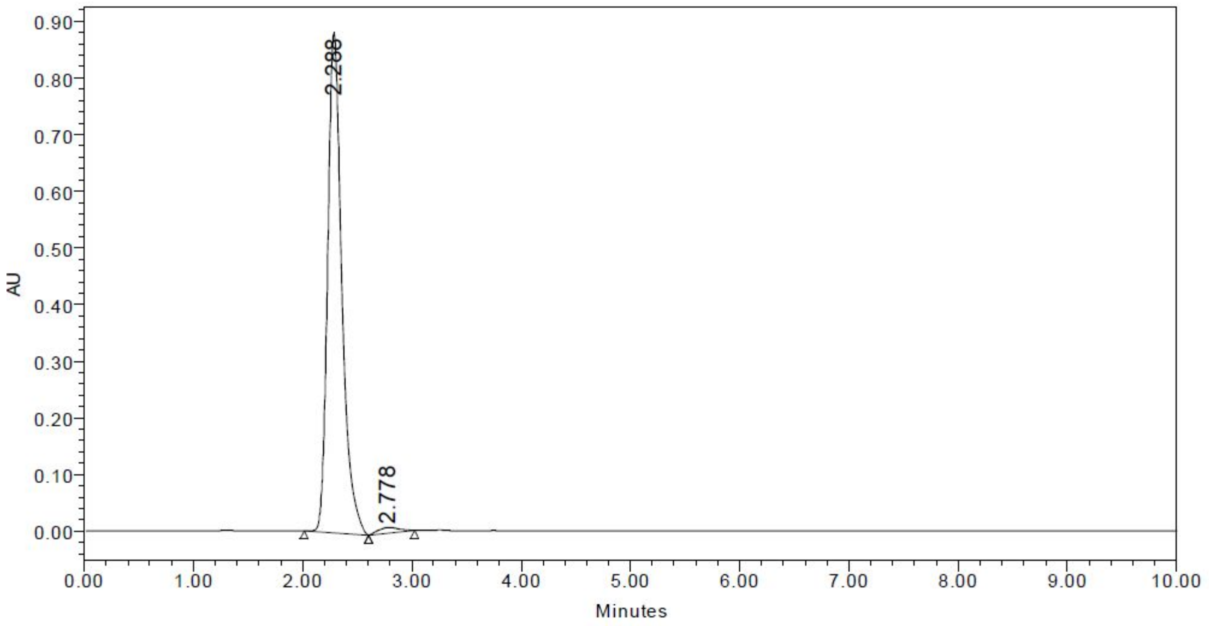

**25**

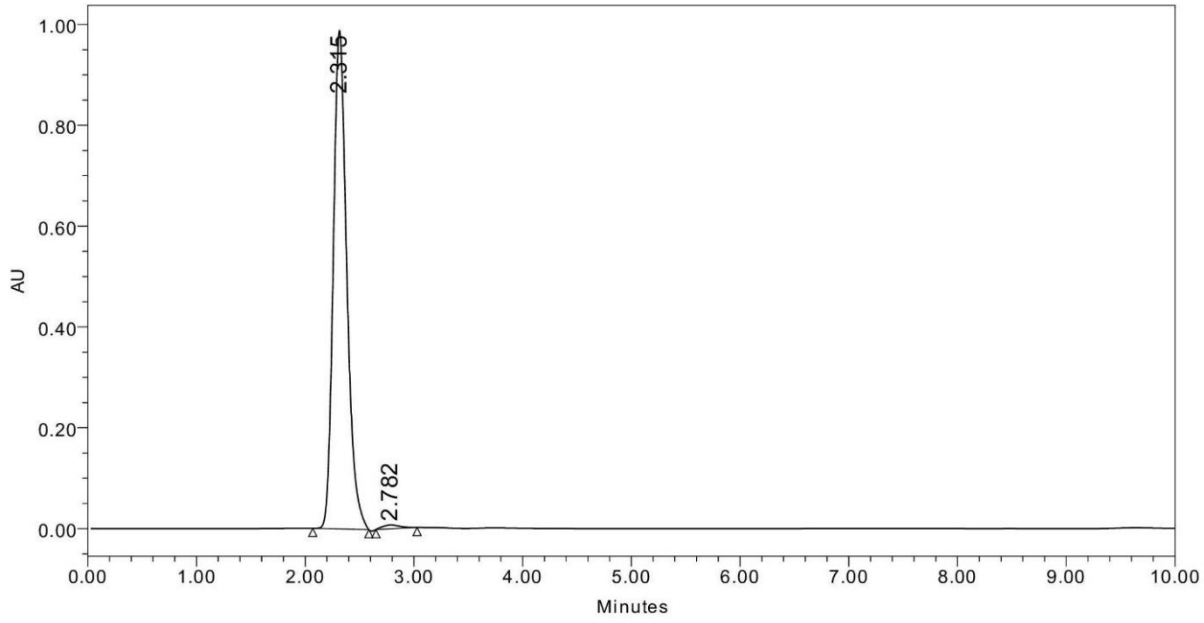
2.345
2.782
1.00
0.80
0.60
0.40
0.20
0.00
AU
0.00 1.00 2.00 3.00 4.00 5.00 6.00 7.00 8.00 9.00 10.00
Minutes

**26**

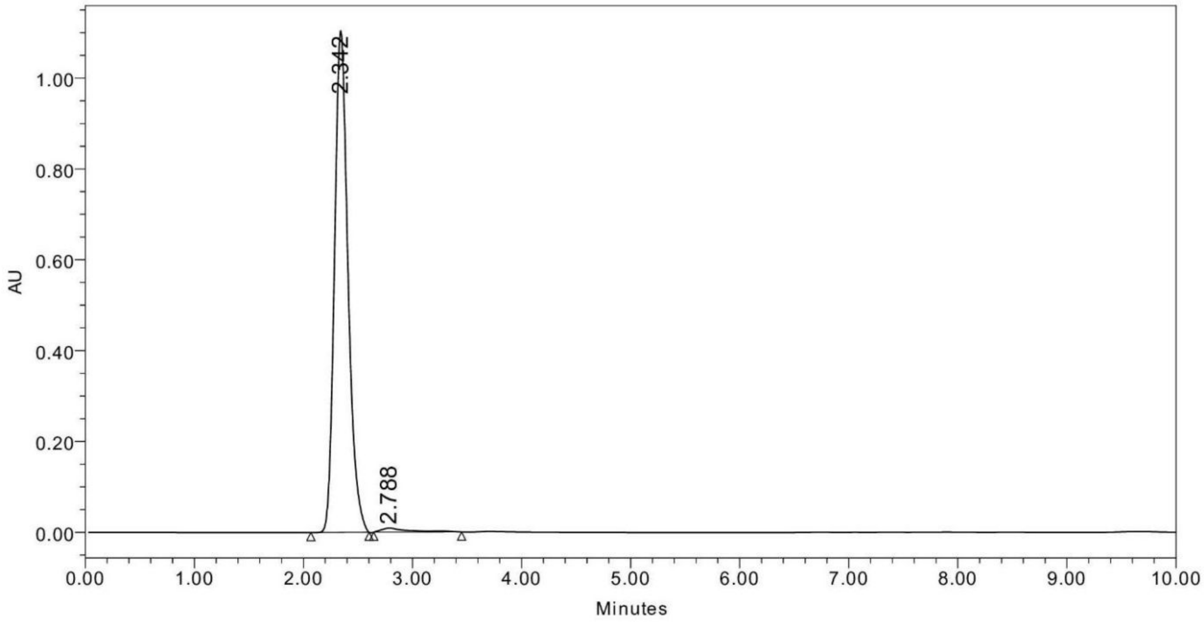
2.342
2.788
1.00
0.80
0.60
0.40
0.20
0.00
AU
0.00 1.00 2.00 3.00 4.00 5.00 6.00 7.00 8.00 9.00 10.00
Minutes

**27**

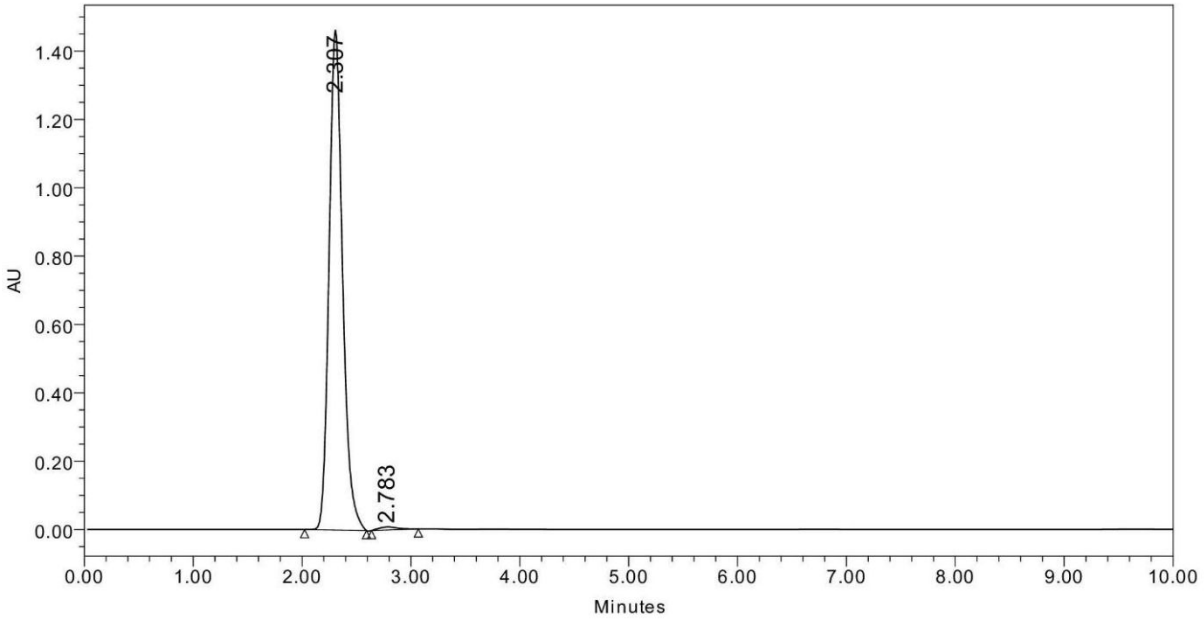
2.307
2.783
AU
1.40
1.20
1.00
0.80
0.60
0.40
0.20
0.00
0.00
1.00
2.00
3.00
4.00
5.00
6.00
7.00
8.00
9.00
10.00
Minutes

**28**

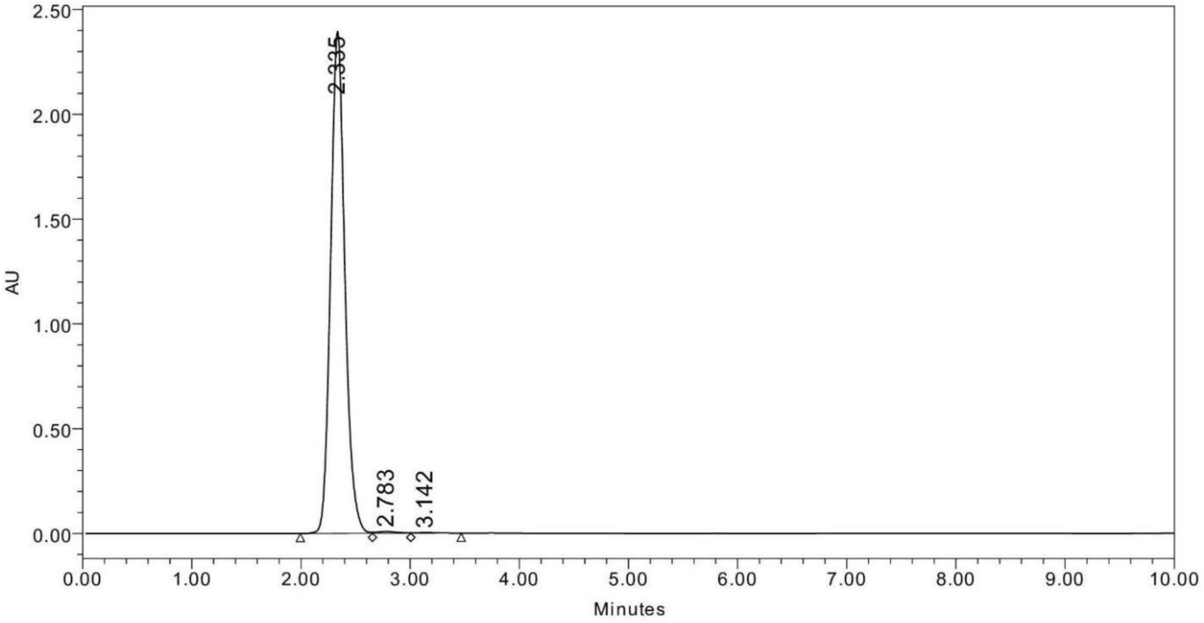
2.335
2.783
3.142
AU
2.50
2.00
1.50
1.00
0.50
0.00
0.00
1.00
2.00
3.00
4.00
5.00
6.00
7.00
8.00
9.00
10.00
Minutes

**29**

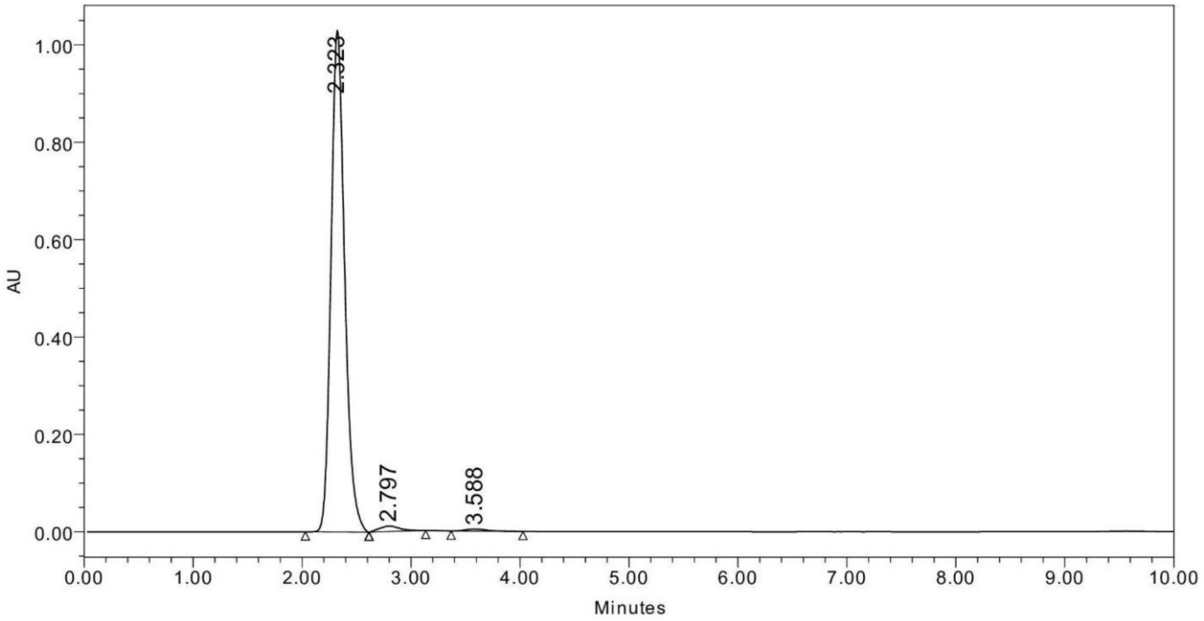
2.323
2.797
3.588
AU
1.00
0.80
0.60
0.40
0.20
0.00
0.00 1.00 2.00 3.00 4.00 5.00 6.00 7.00 8.00 9.00 10.00
Minutes

**30**

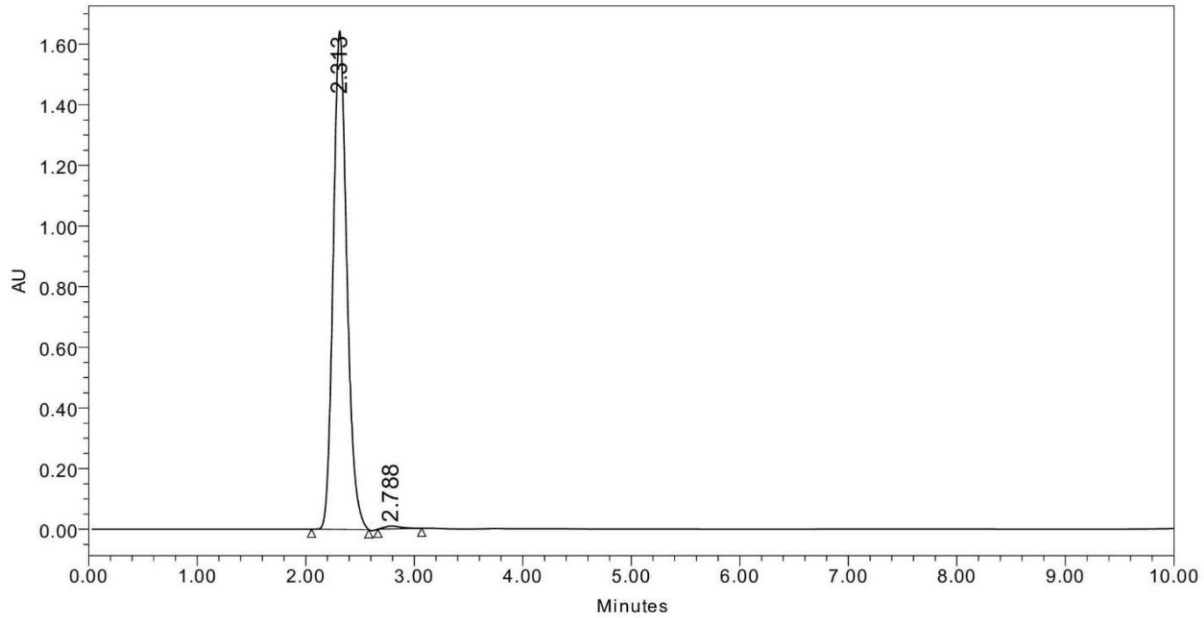
2.313
2.788
AU
1.60
1.40
1.20
1.00
0.80
0.60
0.40
0.20
0.00
0.00 1.00 2.00 3.00 4.00 5.00 6.00 7.00 8.00 9.00 10.00
Minutes

**31**

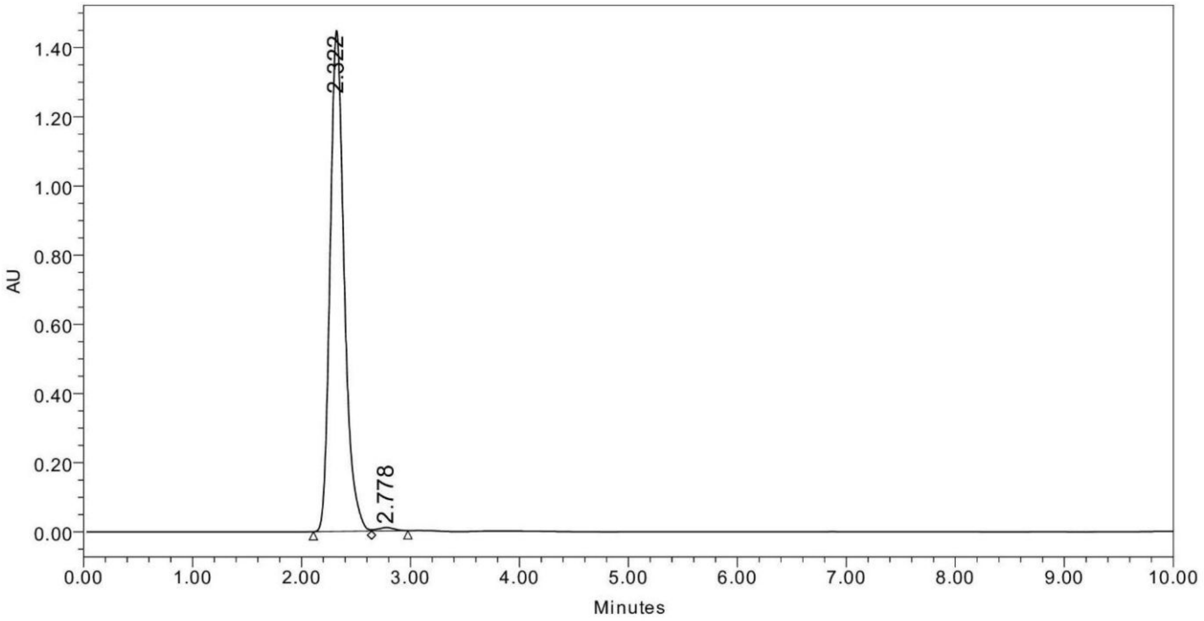

2.322
2.778
AU
1.40
1.20
1.00
0.80
0.60
0.40
0.20
0.00
0.00
1.00
2.00
3.00
4.00
5.00
6.00
7.00
8.00
9.00
10.00
Minutes


**32**

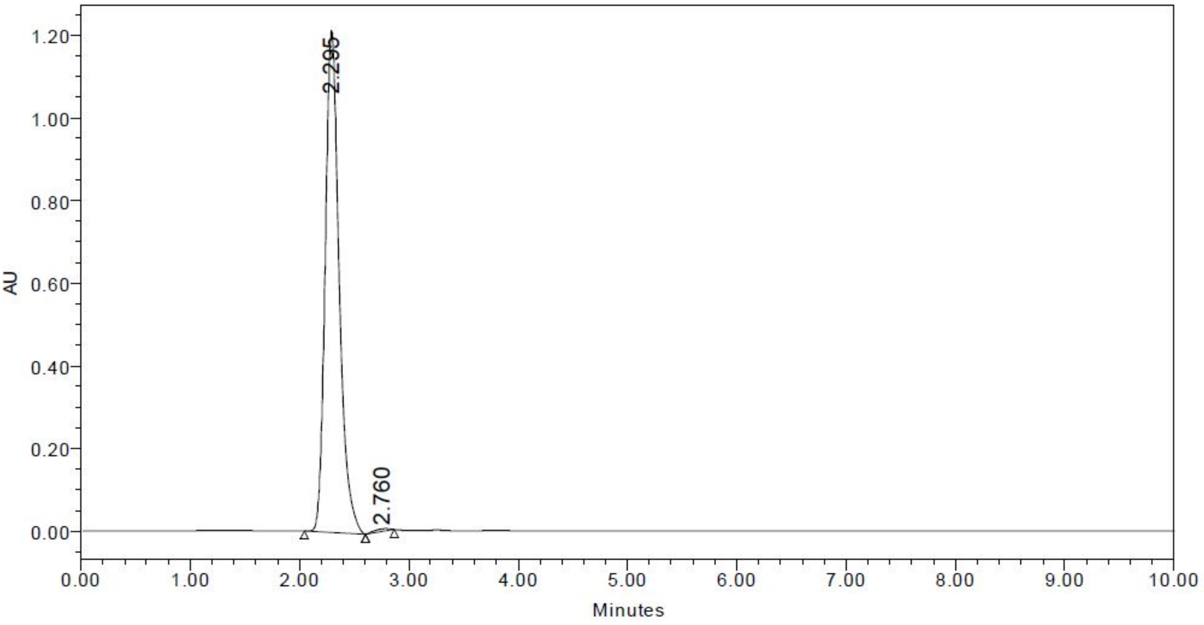

2.295
2.760
AU
1.20
1.00
0.80
0.60
0.40
0.20
0.00
0.00
1.00
2.00
3.00
4.00
5.00
6.00
7.00
8.00
9.00
10.00
Minutes
